# Machine-learning surrogates enable rapid prediction of 3-D substrate gradients in industrial bioreactors

**DOI:** 10.1038/s41467-026-78273-7

**Published:** 2026-10-15

**Authors:** Víctor Puig-I-Laborda, Jorge Carrasco Muriel, Andrea Castañeda Tena, Teddy Groves, Johan le Nepvou de Carfort, Ulrich Krühne, Krist V. Gernaey, Cees Haringa, Ignasi Puig-de-Dou, Lars Keld Nielsen

**Affiliations:** 1 Novo Nordisk Foundation Center for Biosustainability, Technical University of Denmark (The centre was succeeded at DTU by the Novo Nordisk Foundation Biotechnology Research Institute for the Green Transition, BRIGHT, in 2026), Kongens Lyngby, Denmark; 2 Department of Chemical and Biochemical Engineering, Technical University of Denmark, Kongens Lyngby, Denmark; 3 Department of Biotechnology, Faculty of Applied Sciences, Delft University of Technology, Delft, The Netherlands; 4 Escola Tècnica Superior d’Enginyeria Industrial, Departament d’Estadística i Investigació Operativa, Universitat Politècnica de Catalunya, Barcelona, Spain; 5 Australian Institute for Bioengineering and Nanotechnology (AIBN), The University of Queensland, St. Lucia, Brisbane, Australia

**Keywords:** Scientific data, Biotechnology, Fluid dynamics, Applied mathematics

## Abstract

High-fidelity computational fluid dynamics (CFD) provide detailed substrate gradient predictions in industrial stirred-tank bioreactors but are computationally intensive, limiting their routine application. We developed surrogate models trained on 760 CFD simulations to rapidly predict fully 3-D substrate concentration fields from global operating conditions (e.g., stirring speed, feeding strategy, feeding point location, fluid density and viscosity, liquid height, bio-kinetic parameters, biomass concentration), and local spatial descriptors (e.g., distance to the feeding point). Among various methods, attention-based neural networks achieved the highest accuracy (test ${{{\rm{R}}}}^{2}\approx 0.91$), capturing essential mixing features and enabling millisecond-scale inference—up to a million-fold speed-up compared to CFD. These surrogates facilitate rapid exploration of operating spaces, supporting real-time optimization and digital-twin frameworks in bioprocessing. Our results demonstrate that data-driven models can effectively couple hydrodynamics and biokinetics to provide fast, accurate spatial predictions, advancing capabilities for industrial bioreactor design and control.

## Abstract (web summary)

High-fidelity CFD simulations can accurately predict substrate gradients in industrial bioreactors but are computationally too intensive for routine use, limiting real-time process optimization. This study developed machine learning surrogate models trained on CFD data that can predict fully 3D substrate concentration fields in milliseconds.

## Introduction

Bio-based manufacturing—including the production of pharmaceuticals, cosmetics, food additives, fuels and bioplastics—relies on bioreactors to cultivate cells and enzymes at scale^1–3^. Achieving commercial success requires precise control over fluid flow, mass transfer, shear, and cell physiology, an interplay that becomes increasingly complex during scale-up^4^. The stirred-tank reactor (STR) remains the industry standard^1^, but limitations in power input can lead to the development of macroscopic concentration and shear gradients^5^, which negatively impact process efficiency and product yield^6^.

High-fidelity computational fluid dynamics (CFD) can predict these gradients^7^, but even with modern hardware such as GPUs, cloud clusters, and lattice-Boltzmann solvers^8,9^, a single turbulent simulation may require hours to complete—making it impractical for routine optimization or digital-twin control. Compartment models (CMs) offer a viable alternative; however, they typically rely on a prior CFD simulation to define the flow field and are mainly used to accelerate the simulation of transport of diluted species within the compartmentalized fluid flow^10–12^. Machine learning (ML) surrogates trained on CFD-generated data can reduce evaluation times by several orders of magnitude^13–15^. This approach has matured to the point where major CFD vendors now provide ML/AI emulators^16^.

Nevertheless, in bioprocessing, most ML applications remain limited to 2-D tasks such as fouling detection^17^, basic control^18^, or 0-D, physics-informed kinetics^19,20^. Existing CFD-ML hybrids rarely couple hydrodynamics with cellular behavior^21^, leaving the spatial variation in cell physiological states (spatial physiology) largely unmodeled, even though errors in substrate flux across different regions of the reactor can significantly affect cell growth and product yield^22–25^.

Physics-informed neural networks (PINNs) provide an alternative scientific-ML route in which governing equations, such as the Navier-Stokes equations, are incorporated directly into the training objective through physics-based residual losses. PINNs have been demonstrated for forward and inverse problems involving non-linear partial differential equations (PDEs) and have been increasingly explored in fluid mechanics^26,27^. However, PINNs do not remove the computational difficulty of fluid-flow modeling: training can be numerically demanding, sensitive to loss weighting and boundary-condition specification, and challenging for high-Reynolds-number turbulent flows or large parametric design spaces^27^. In contrast, fully supervised CFD-trained surrogates require an upfront simulation database, but once trained, they provide extremely fast interpolation across the sampled operating space.

Here, we develop supervised-learning surrogate models trained on CFD data to predict fully 3-D substrate concentration fields in stirred-tank bioreactors across operating conditions and fermentation stages. The models provide millisecond, spatially resolved gradient predictions, enabling rapid design-space exploration and supporting real-time optimization and digital-twin workflows without repeated CFD.

## Results

**Fig. 1:**
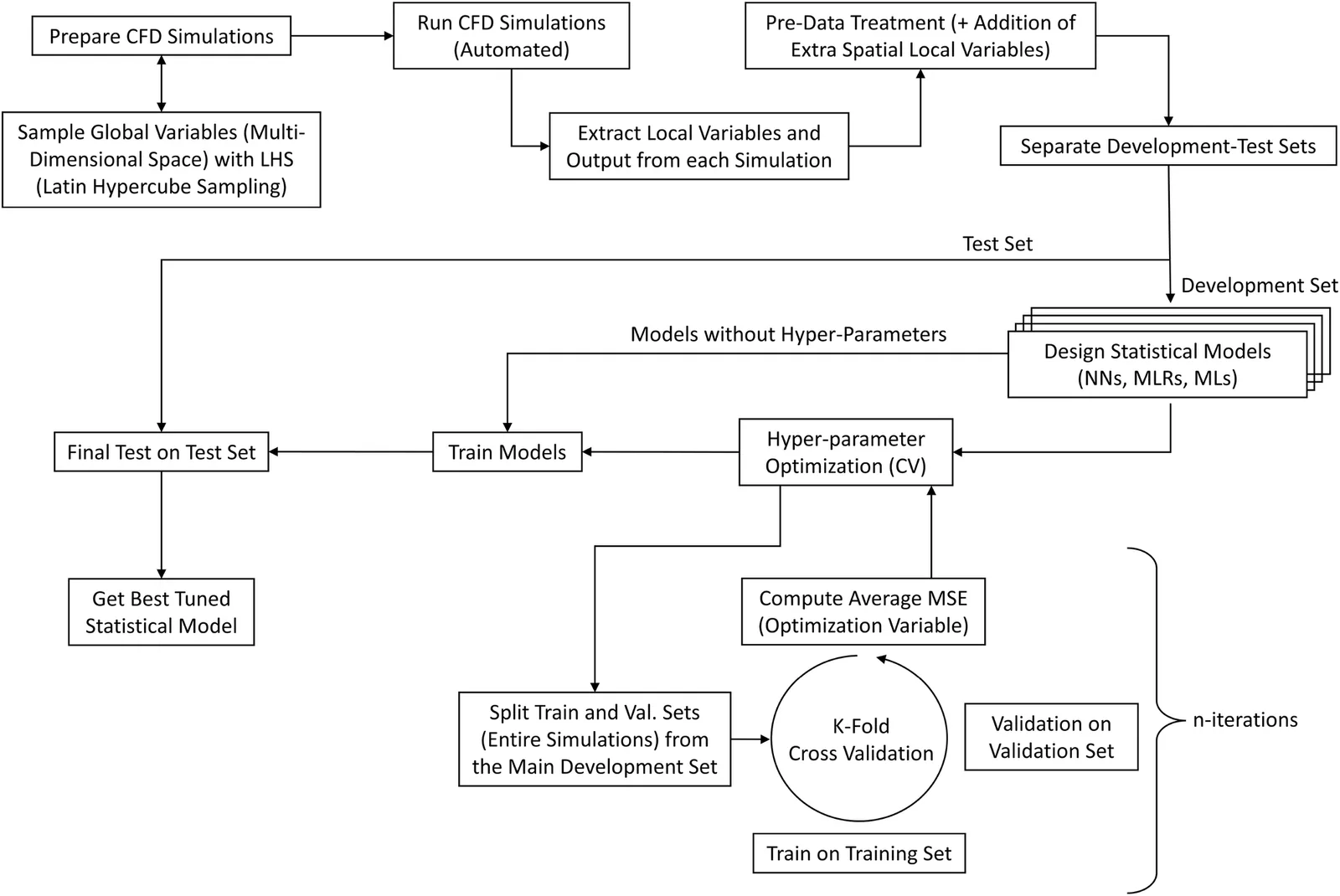
End-to-end workflow for the development of CFD-driven surrogate models. The pipeline integrates automated data generation with rigorous statistical benchmarking. Top: Global operating conditions are selected via Latin hypercube sampling (LHS) to drive high-fidelity CFD simulations, generating a dataset of 3-D substrate concentration fields. Middle: Local flow variables are extracted and enriched with spatial descriptors before being partitioned into development and strictly held-out test sets. Bottom: Candidate models—ranging from linear regression to neural networks (NN)—undergo hyperparameter optimization via ${{\boldsymbol{k}}}$-fold cross-validation (minimizing MSE) prior to final evaluation on the test set. ${{\bf{MSE}}}$, mean squared error. MLR, multiple linear regression. MLs machine learning models. CV cross-validation.

To overcome the prohibitive computational cost of CFD in bioreactor simulation, we developed an end-to-end framework for training data-driven surrogates that predict 3-D substrate concentration fields faster than real time. The workflow, summarized in Fig. 1, integrates automated CFD data generation with rigorous statistical benchmarking to identify model architectures that balance accuracy, data efficiency, and physical interpretability.

The framework was deployed on an industry-standard 4-Rushton-impeller stirred-tank bioreactor^1^. To ensure the surrogates captured a wide range of process dynamics, we utilized Latin Hypercube Sampling^28^ to select global operating conditions—including stirring speed, feeding point location, feed rate, fluid properties and biokinetic parameters—which were then simulated to generate reference 3-D concentration fields. The learning task was formulated to map these global parameters, combined with local spatial descriptors (e.g., Cartesian coordinates, distance to the feeding point), directly to local concentration values.

We assessed the performance of candidate models ranging in complexity from linear baselines to complex attention-based neural networks^29^. All models were implemented using open-source software^30,31^. Additional statistical background is provided in Supplementary Note 1.

The benchmarking suite included multiple linear regression (MLR) and its variants (Lasso, Ridge, elastic net, Huber, polynomial + Lasso), k-nearest neighbors (k-NN), decision trees (DT), ensemble methods such as random forest regression (RFR) and gradient boosting regression (GBR), as well as support vector regression (SVR) and neural networks (NNs). The suitability of these surrogates was evaluated across five key dimensions: (i) representation—impact of target variable transformation on learning stability; (ii) accuracy—generalization performance on a strictly held-out test set; (iii) efficiency—data requirements quantified via learning-curve analysis; (iv) fidelity—capability to reconstruct complex 3-D spatial gradients relative to CFD reference; and (v) speed—inference latency compared to full-physics simulations.

Additional results, such as hyperparameter tuning for all models and best subset analysis for MLR, which complement the main objective of the paper, are presented in the supplementary material.

### Target representation and predictor analysis

To identify the optimal target for surrogate modeling, we first analyzed the statistical properties of the CFD-generated training set. The volume-weighted distribution of raw substrate concentration (${C}_{S}$) was heavily right-skewed (Supplementary Fig. 1). This distribution is a direct consequence of the transport-reaction balance along streamlines, ${{\bf{U}}}\cdot \nabla {C}_{S}\approx -r\left({C}_{S}\right)$, which yields an approximately exponential decay from the feed point when Monod kinetics operate in a near first-order regime $\left({C}_{S}\ll {K}_{S}\right)$. Consequently, a small fraction of the vessel volume retains high ${C}_{S}$ while the majority experience significantly lower levels.

To stabilize learning across these sharp gradients, we evaluated four target representations: raw ${C}_{S}$, ${\log }_{10}{C}_{S}$, the biologically bounded cell performance ${q}_{S}/{q}_{S,\max }$ and a rank-based quantile transform ${{\rm{qt}}}\left({C}_{S}\right)$. Across model families ${q}_{S}/{q}_{S,\max }$ and ${\log }_{10}{C}_{S}$ yielded the highest predictive accuracy. While models trained on ${\log }_{10}{C}_{S}$ achieved high scores in log-space, back-transformation into concentration units amplified errors multiplicatively, leading to wide uncertainty bands at high concentrations. By contrast, the ${q}_{S}/{q}_{S,\max }$ ratio offered nearly identical accuracy while mapping predictions to a physically meaningful $\left[{\mathrm{0,1}}\right]$ scale without requiring error-prone inverse mappings. We therefore adopted ${q}_{S}/{q}_{S,\max }$ as the primary target for subsequent analysis. For transparency, complete benchmarking results, selected hyperparameters and best-subset analyses for all target representations are provided in Supplementary Figs. 5–11 and Supplementary Tables 1–4 and 7–9.

We quantified linear associations among predictors and between predictors and each target transformation. Pairwise Pearson coefficients were generally close to zero, with a single structured block along the axial direction where the axial coordinates ($y,\,{y}_{{{\rm{feed}}}}$) and liquid height showed moderate correlation because of geometric constraints imposed by the free surface (Supplementary Fig. 2). Principal component analysis confirmed the absence of a low-dimensional manifold: explained variance increased nearly linearly with the number of components, and only the first component was dominated by the axially constrained variables (Supplementary Figs. 3 and 4).

Across targets (${C}_{S}$, ${\log }_{10}{C}_{S}$, ${{\rm{qt}}}\left({C}_{S}\right)$, ${q}_{S}/{q}_{S,\max }$), the strongest correlations arose with a compact set of predictors: global set points (${F}_{V}$, ${C}_{X}$, ${K}_{S}$, ${q}_{S,\max }$) and the local distance to the feeding point. This pattern indicates that global operating conditions set the mean substrate level, whereas distance to the feeding point captures deviations from homogeneity. The design of experiments used LHS, which provided uniform coverage of the 11-D space despite weak pairwise correlations (Supplementary Fig. 2)^32^. For model design, we selected compact predictor sets for linear baselines (MLR) using information criteria (e.g., ${{\rm{AIC}}}$) in a best-subset analysis, whereas flexible learners (e.g., NNs or ML-models) retained all features and relied on regularization or attention to down-weight weak predictors.

### Benchmarking on test set

Prior to full-scale benchmarking, we performed a best-subset screening for the linear baseline (MLR) to identify the most informative predictors. Both the Akaike Information Criterion (${{\rm{AIC}}}$) and adjusted ${{{\rm{R}}}}^{2}$ (Methods) consistently selected compact feature sets dominated by global operating conditions and the local distance to the feeding point. Raw spatial coordinates and fluid properties (density and viscosity) were rarely selected, likely due to the narrow sampling ranges inherent to standard bioreactor operation (Table 1, Methods). Notably, stirring speed was frequently excluded for transformed targets, a redundancy attributed to the dominance of feed-distance effects in the high-Reynolds-number mixing regime. Full subset diagnostics are provided in Supplementary Tables 1–4. This pruning step was exclusive to linear baselines; flexible architectures (NNs, ensembles) were trained on the full predictor set, relying on internal regularization and attention to control overfitting and suppress noise.

**Table 1:** Operational parameter intervals for the global parameters in the CFD simulations

Global variable	Interval	Unit
${{\bf{liquid\; height}}}$	3 − 12.5	$\left[{{\rm{m}}}\right]$
${{\bf{stirring\; speed}}}$	5 − 700	$\left[{{\rm{rpm}}}\right]$
${{{\boldsymbol{F}}}}_{{{\boldsymbol{V}}}}$	$1\times {10}^{-4}$ − $5\times {10}^{-3}$	$\left[{{\rm{kg}}}{{{\rm{m}}}}^{-3}{{{\rm{s}}}}^{-1}\right]$
${{\boldsymbol{\mu }}}$	0.001 − 0.02	$\left[{{\rm{kg}}}{{{\rm{m}}}}^{-1}{{{\rm{s}}}}^{-1}\right]$
${{\boldsymbol{\rho }}}$	950 − 1250	$\left[{{\rm{kg}}}{{{\rm{m}}}}^{-3}\right]$
${{{\boldsymbol{C}}}}_{{{\boldsymbol{X}}}}$	2 − 75	$\left[{{\rm{kg}}}{{{\rm{m}}}}^{-3}\right]$
${{{\boldsymbol{K}}}}_{{{\boldsymbol{S}}}}$ (mass fraction)	$1\times {10}^{-5}$ − $2\times {10}^{-4}$	$\left[{{{\rm{kg}}}}_{{{\rm{S}}}}\,{{{\rm{kg}}}}^{-1}\right]$
${{{\boldsymbol{q}}}}_{{{\boldsymbol{S}}}},{{\max }}$	$6.95\times {10}^{-5}$ − $4.86\times {10}^{-4}$	$\left[{{{\rm{kg}}}}_{{{\rm{S}}}}\,{{{{\rm{kg}}}}_{{{\rm{X}}}}}^{-1}{{{\rm{s}}}}^{-1}\right]$
${{{\boldsymbol{r}}}}_{{{\bf{feed}}}}$	0 − 2.1875	$\left[{{\rm{m}}}\right]$
${{{\boldsymbol{\theta }}}}_{{{\bf{feed}}}}$	0 − 2π	−
${{{\boldsymbol{y}}}}_{{{\bf{feed}}}}$	0 − 12.5	$\left[{{\rm{m}}}\right]$

**Fig. 2:**
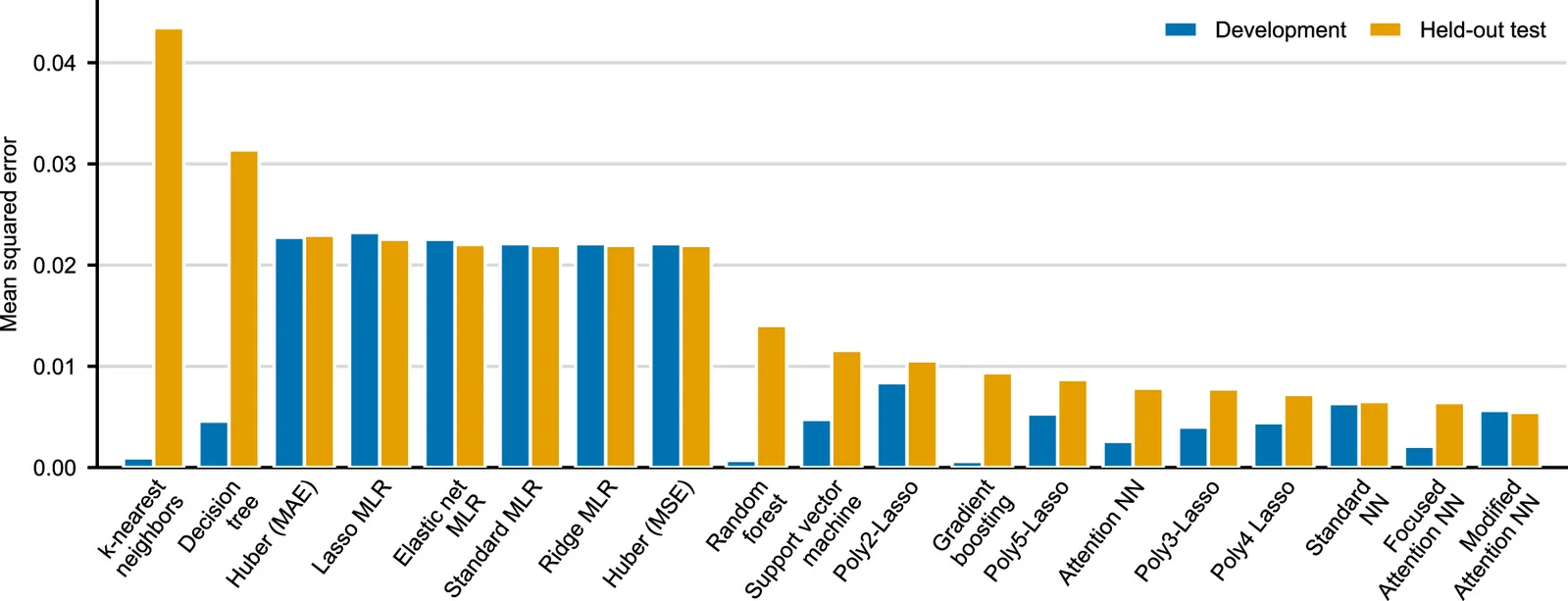
Generalization performance of candidate surrogate models on the primary target ${q}_{s}/{q}_{s,\max }$. Bars represent the MSE on the development set (blue) and the strictly held-out test set (orange). Models are ranked by test-set accuracy (left to right: worst to best). (Left) Non-parametric methods like ${{\boldsymbol{k}}}$-NN and single DTs suffer from high variance and poor generalization. (Middle) Linear baselines (MLR, Lasso, elastic net) offer stable but limited capacity, while tree ensembles (random forest) exhibit significant overfitting gaps. (Right) The Modified-Attention NN achieves the lowest test error (MSE = 0.0054) by effectively capturing non-linear interactions between global operating conditions and local spatial features.

We then trained the models on the development set of 560 CFD simulations using the protocol described in Fig. 1, with the optimal hyperparameters for each model (Supplementary Table 9), and evaluated them on an independent test set of 200 previously unseen simulations. Development and test performance for the primary target ${q}_{S}/{q}_{S,\max }$ is summarized in Fig. 2, with complementary ${{{\rm{R}}}}^{2}$ results reported in Supplementary Fig. 5.

The Modified-Attention NN achieved the lowest ${{\rm{MSE}}}$ (0.0054), and a test ${{{\rm{R}}}}^{2}$ of 0.91. Its superior performance is consistent with its architecture, which separates global set points from local spatial descriptors and uses attention mechanisms to let operating conditions modulate the local representation (Methods). Although Supplementary Figs. 2–4 show weak pairwise linear correlations among the raw inputs, these analyses do not exclude higher-order, non-linear interactions between process conditions and spatial features.

Polynomial–Lasso regressors at intermediate degree (3rd−4th order) emerged as the next best class. These models were flexible enough to capture dominant nonlinearities, while ${{{\mathcal{l}}}}_{1}$ regularization effectively pruned the interaction space to prevent overfitting, a limitation observed in pure 5th-order polynomials. However, their larger development–test gap compared with the NNs indicates some overfitting. Thus, Poly-Lasso reduced the bias compared to most models but did not provide the same generalization robustness as the best-performing NNs.

Tree-based ensembles (random forest, gradient boosting) were competitive during training but exhibited significant development–test gaps. This generalization gap suggests that row-level bootstrapping was insufficient to counteract the strong influence of global predictors, leading trees to memorize specific simulation contexts rather than learning invariant mixing rules. Support Vector Regression performed respectably but at a higher computational cost. Classical linear regressors (OLS, Ridge, Lasso, Elastic Net) formed a stable, fast baseline with small generalization gaps yet higher test MSE, consistent with limited capacity to represent mixing–uptake curvature.

The weakest results came from k-NN and single decision trees (DT), consistent with the curse of dimensionality for distance-based methods and the high variance of unregularized trees. Their errors were poor in two ways. (i) Limited accuracy: with ${q}_{S}/{q}_{S,\max }\in \left[{\mathrm{0,1}}\right]$, test MSEs around 0.045 (${{\rm{RMSE}}}\approx 0.21$) and low coefficients of determination (e.g., ${{{\rm{R}}}}^{2}\approx 0.2$ for k-NN) indicate limited explanatory power. (ii) Poor generalization: Large gaps between training and test MSE, particularly pronounced for decision trees, suggest overfitting (memorizing patterns of the training data rather than learning the global trends).

Residual analysis further distinguished the model tiers: Weaker learners exhibited a characteristic bias—overpredicting at low saturation and underpredicting at higher saturation—along with strong residual–predictor correlations. In contrast, the top performers showed predicted values concentrated around the 1:1 line, residuals centered close to zero with near-constant spread, and minimal correlation with either ${y}_{{{\rm{true}}}}$ or the inputs, confirming that systematic mixing structures and reactions were successfully captured. Residual analysis figures and extensive discussion are compiled in Supplementary Discussion 4 and Supplementary Figs. 12–30.

To further assess interpolation within the sampled operating domain, we performed one-dimensional operating sweeps in ${q}_{S,\max }$, ${y}_{{{\rm{feed}}}}$ and ${{\rm{stirring\; speed}}}$, showing smooth surrogate responses that generally followed CFD trends at fixed monitoring points (Supplementary Discussion 5, Supplementary Table 5 and Supplementary Fig. 31).

### Data efficiency and learning dynamics

To quantify the training data requirements for accurate surrogate modeling, we performed a learning-curve analysis via simulation-level subsampling. From the 560-run development set we iteratively drew random subsets of increasing size (from 10 to 560 simulations, 5 simulations per step), split them 80%/20% into training/validation (entire simulations), and evaluated performance using optimum fixed hyperparameters (Supplementary Table 9).

**Fig. 3:**
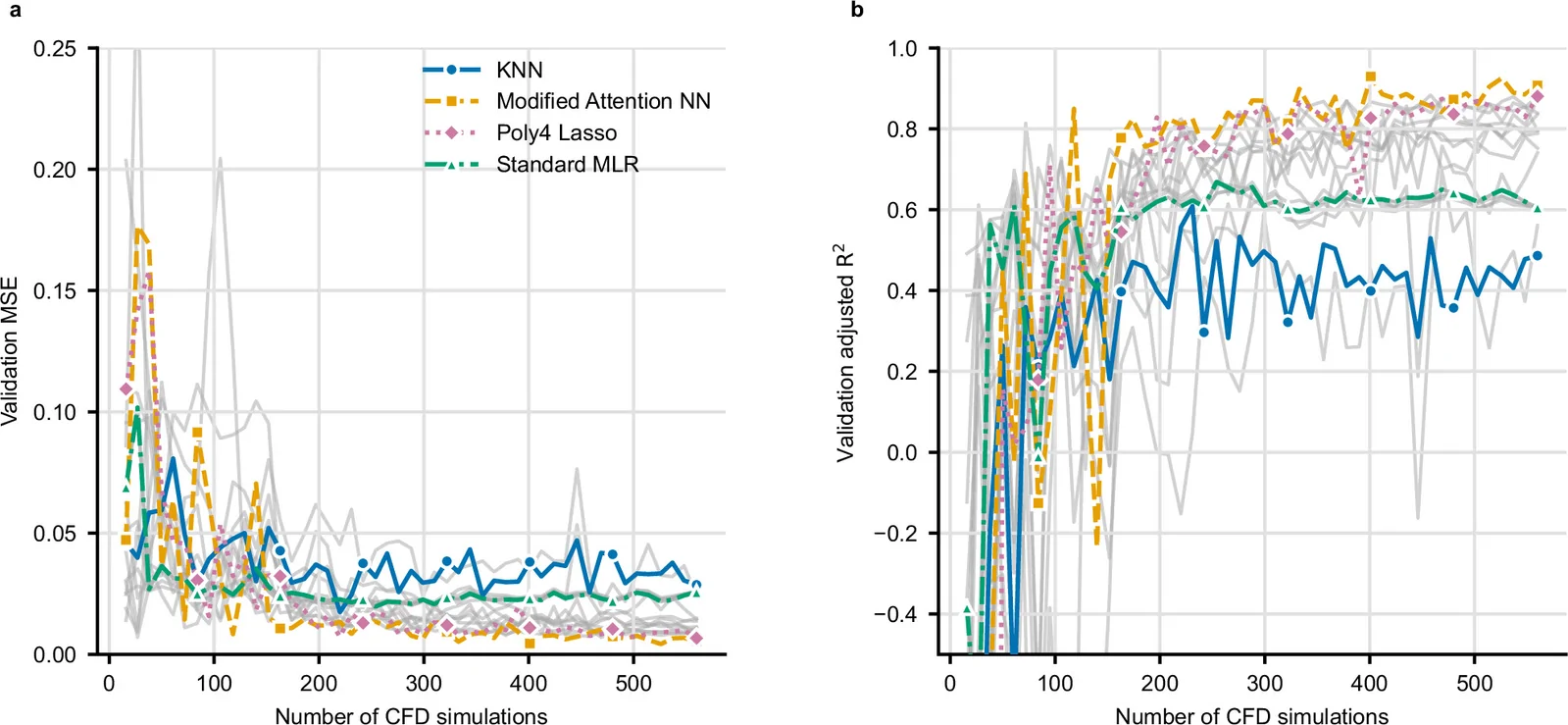
Impact of training set size on surrogate generalization. Validation performance metrics—**a** mean squared error (MSE), and **b** adjusted coefficient of determination $\left({{{\bf{R}}}}_{{{\bf{adj}}}}^{{{\boldsymbol{2}}}}\right)$—are plotted as functions of the number of unique CFD simulations used for model development. Colored lines highlight four representative architectures, while gray lines denote the remaining benchmarked models. Low-capacity models, such as MLR and ${{\boldsymbol{k}}}$-NN, reach an early performance plateau, whereas higher-capacity models continue to benefit from additional simulations.

Two regimes emerged (Fig. 3): Low-capacity models (e.g., MLR, k-NN) rapidly saturated, achieving their minimum error floor after ~200 simulations. This plateau indicates a bias-limited regime, where additional data cannot overcome the model’s inability to represent complex mixing-reaction dynamics.

Conversely, high-capacity architectures (specifically the Modified-Attention NN and 4th-order Poly-Lasso) continued to reduce error well beyond 400 simulations. These models exhibit variance-limited behavior in early training, allowing them to extract subtle non-linear dependencies as more data becomes available.

**Fig. 4:**
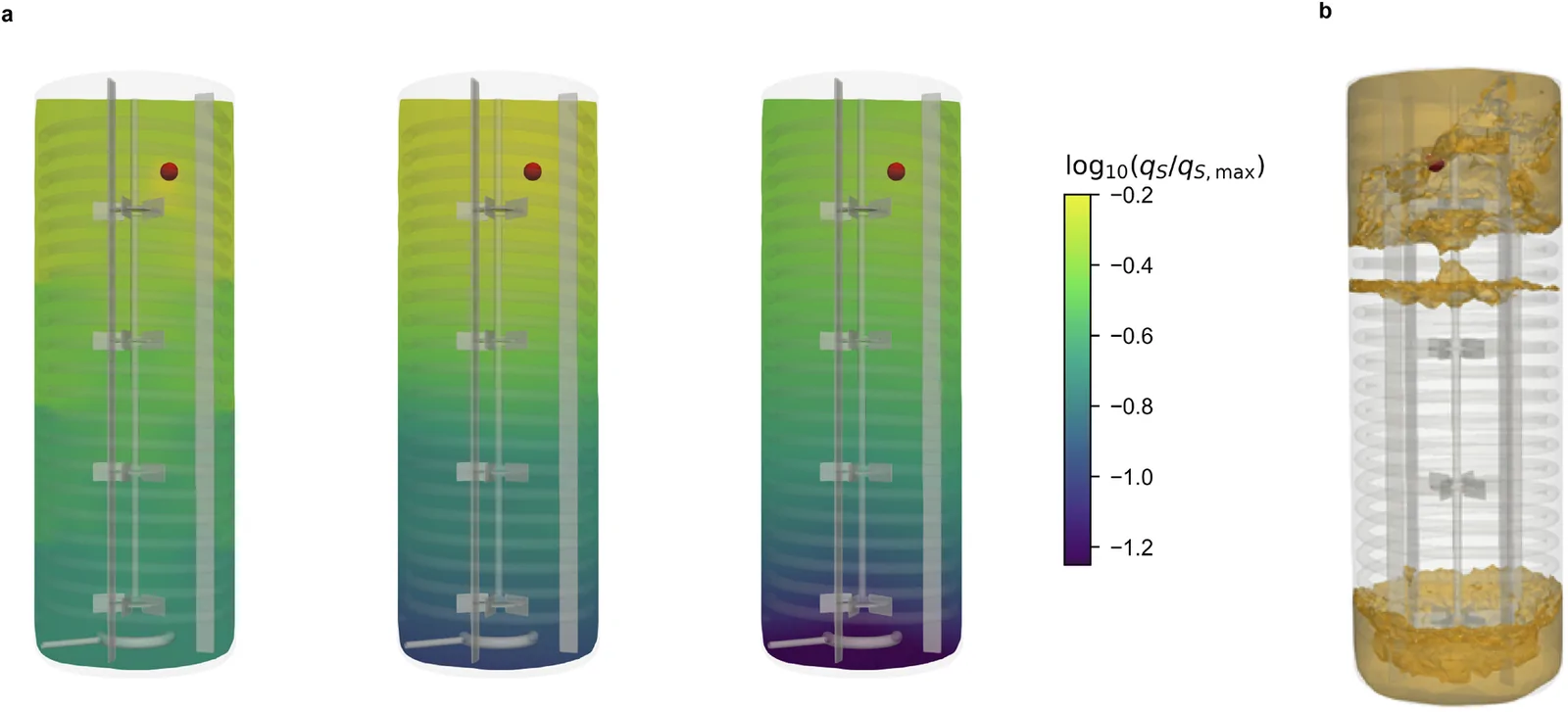
Three-dimensional reconstruction fidelity and spatial localization of prediction errors. **a** Logarithmic saturation ratio, ${{{\log }}}_{{{\boldsymbol{10}}}}\left({{{\boldsymbol{q}}}}_{{{\boldsymbol{S}}}}/{{{\boldsymbol{q}}}}_{{{\boldsymbol{S}}},{{\max }}}\right)$, for a representative held-out test case, shown from left to right for the CFD reference, the Focused-Attention NN prediction and the multiple linear regression (MLR) baseline. The red sphere indicates the feeding point. **b** Spatial localization of relative prediction errors for the Focused-Attention NN in a representative held-out test case. Brown mesh elements indicate regions with a relative error greater than ${{\boldsymbol{5}}}\,{{\boldsymbol{\%}}}$; errors are concentrated at both extremes of the reactor (close and far away from the feeding point) and at inter-impeller boundaries.

From an engineering perspective, this reveals a critical cost–accuracy trade-off. Roughly 200 diverse CFD runs are sufficient to saturate the linear baselines, whereas higher-capacity models continue to benefit from larger training sets. However, beyond a few hundred additional runs, the improvements in accuracy become increasingly marginal relative to the computational cost. This is consistent with the spatial error analysis, which showed that the remaining errors are concentrated in rare, localized regions (Fig. 4b). Additional randomly sampled simulations therefore provide limited extra information unless combined with more spatially expressive architectures like graph-NN or CNNs^33,34^, targeted sampling or loss reweighting. This trade-off is particularly acute in bioreactor CFD, where each additional case demands substantial time and resources. When minimizing error is critical, allocating extra simulations to the most expressive models can be justified, but the returns diminish quickly. Moreover, marginal error reduction depends not only on sample count but also on effective coverage of the operating space, underscoring the importance of efficient experimental designs such as LHS. Full learning curves, including log-scale views for the alternative target representations are provided in Supplementary Discussion 6 and Supplementary Figs. 32–36.

This result also informs us how the framework may scale when additional input variables are introduced. Adding new operating or geometric descriptors increases input dimensionality, but the flattening of the learning curves suggests that limited extensions may be feasible through targeted CFD sampling rather than exhaustive re-sampling of the full expanded space. The number of additional simulations required will depend on how strongly the new variable alters the flow or reaction regime.

### Spatial reconstruction fidelity and localized error analysis

To assess spatial reconstruction fidelity relative to the CFD reference, we performed full 3-D reconstruction for the 200 unseen test cases. Aggregate metrics (${{\rm{MSE}}}$) often mask localized deficiencies; however, visual inspection confirmed that neural architectures successfully captured the reactor’s compartmentalization (Fig. 4a).

The Focused-Attention NN reproduced substrate gradients with remarkable fidelity, accurately recovering the exponential decay from the feed and delineating the sharp interfaces between Rushton-impeller compartments. While the Modified-Attention NN achieved a marginally lower global ${{\rm{MSE}}}$, its spatial contours appeared slightly more diffuse compared to the Focused-Attention NN. This trade-off reflects architectural nuances: the Focused-Attention NN embeds and regularizes global features prior to reweighting, a process that seemingly favors clearer spatial localization over raw global error minimization (Methods, Eq. (16)).

In contrast, MLR provided a robust but coarse baseline. Unable to resolve non-linear compartmental structures, it produced smooth, diffusive fields reminiscent of pure Fickian transport. Nevertheless, its ability to evaluate rapidly with minimal training data (~200 runs) makes it a viable candidate for early-stage process screening where directional guidance suffices over high-fidelity resolution.

Spatial error mapping explicitly identified the dominant failure modes. Systematic deviations clustered in two distinct regimes: (i) saturation extremes, specifically overprediction near the concentrated feed inlet and underprediction in the depleted zones near the bottom wall; and (ii) high-shear mixing zones, particularly the inter-impeller planes where counter-rotating jets converge (Fig. 4b).

The error concentration in these regions highlights a fundamental challenge: the confluence of steep physical gradients and local data sparsity. The intense shear and intermittency at impeller junctions create sharp, rapidly evolving concentration filaments that surrogates—optimized on the bulk volume—struggle to resolve. While re-parametrizing the target (e.g., logit transformation) could up-weight these extremes, we retained the bounded [0, 1] scale to preserve physical interpretability. These findings suggest that targeted oversampling of high-gradient volumes or physics-informed loss reweighting could further suppress these edge biases.

Overall, the surrogate reproduces global gradient geometries well but remains less reliable for local extrema near the feed, depleted zones and high-shear impeller regions (where CFD can experience numerical difficulties). Its industrial role is therefore best framed as rapid screening to identify operating conditions that deserve high-resolution CFD or experimental follow-up.

### Computational efficiency and real-time inference

**Fig. 5:**
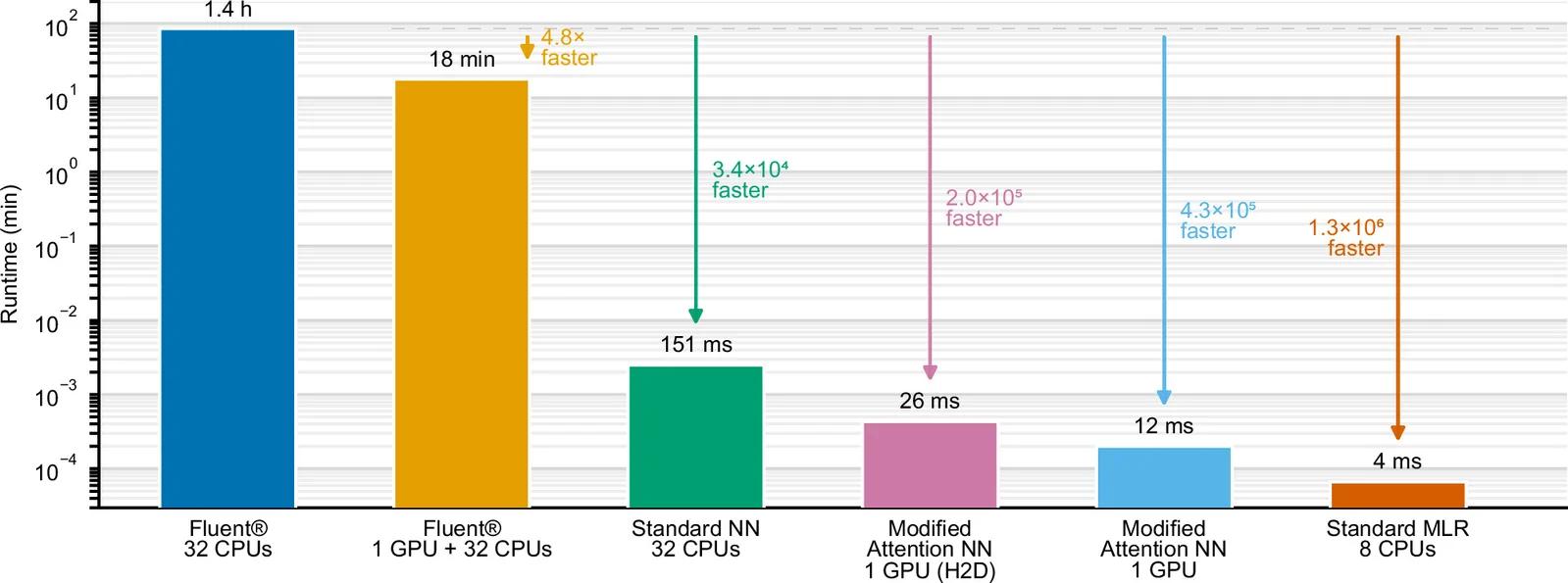
Computational speed-up of surrogate models relative to high-fidelity CFD. The bar chart compares the wall-clock time required to predict a single three-dimensional cell-performance field. Standard CFD simulations require minutes to hours, even with GPU acceleration, whereas the surrogate models reduce inference time to the millisecond scale. Vertical arrows indicate the corresponding reductions in runtime relative to the CPU-based CFD baseline. H2D denotes host-to-device memory-transfer latency. The ${{\boldsymbol{y}}}$ axis is logarithmic.

A defining advantage of the surrogate framework is the drastic reduction in evaluation time compared to full-physics simulations. As quantified in Fig. 5, a single CFD realization on 32 CPU cores (Xeon Gold 6226 R) required ~1.4 h. Even with GPU acceleration (GeForce RTX 3090), runtimes remained on the order of tens of minutes. In contrast, inference for the trained surrogates operated in the millisecond regime: the MLR baseline required $\approx 4\,{{\rm{ms}}}$ per evaluation on a standard CPU, while NNs predicted full 3-D fields in 10–30 ms on a single GPU. This translates to speed-ups ranging from ${10}^{4}$ to $1.3\times {10}^{6}$ relative to the CFD baseline, effectively transitioning complex reactor modeling from offline analysis to near real-time deployment.

However, this inference speed amortizes a substantial upfront computational investment. The dominant offline cost was the generation of the CFD database itself: the present surrogate models were developed from 760 CFD simulations, each requiring ~1–$3\,{{\rm{h}}}$ on 32 CPU cores under the standard workflow, corresponding to an average of $\sim \,2\,{{\rm{h}}}$ per case, or ~$1.5\times {10}^{3}$ simulation hours ($\sim \,4.9\times {10}^{4}\,{{\rm{CPU}}}\; {{\rm{core}}}\; {{\rm{h}}}$) for the full dataset. This cost is incurred once when constructing a domain-specific surrogate; repeating the approach for a substantially different reactor, fluid model, or operating domain would require either a new CFD data-generation campaign or access to a suitable pre-existing CFD database. Importantly, this upfront cost is expected to decrease as GPU-accelerated CFD, lattice-Boltzmann solvers and community CFD databases become more widely available^9,35^.

Model development added a second offline cost, especially for the NN models. NNs required extensive hyperparameter optimization, with cross-validation and architecture search, taking from several days to approximately two weeks, depending on the search space and hardware availability (at best GeForce RTX 3090); final training required up to two additional days. In contrast, linear baselines such as MLR and Poly-Lasso were trained within minutes and required no architecture search (Methods). Thus, model choice introduces an additional cost–accuracy trade-off: high-capacity models provide maximum fidelity at substantial offline optimization cost, whereas simpler regressors enable rapid, low-overhead deployment for initial screening. As CFD solvers and ML optimization workflows continue to accelerate, the practical cost of constructing domain-specific surrogates is expected to decrease.

Once models are trained, however, full-field predictions are achieved in the millisecond regime, enabling rapid interpolation across the sampled operating domain (Supplementary Discussion 5 and Supplementary Fig. 31), screening of hundreds of operating points, and computationally feasible integration into iterative optimization or operator-support workflows.

## Discussion

We present this study as a proof of concept for real-time prediction of 3-D reaction–transport fields. To our knowledge, it is among the first demonstrations of surrogate models that directly predict 3-D substrate gradients in stirred bioreactors while incorporating both hydrodynamic flow patterns and substrate uptake kinetics. Since Vinuesa and Brunton^13^ highlighted the potential of ML surrogates to accelerate CFD while retaining physical structure, substantial progress has been made in ML-enhanced fluid mechanics. However, existing hybrids in bioprocessing have largely targeted global hydrodynamic quantities (e.g., power number, mixing time) or low-dimensional outputs such as ${k}_{{{\rm{L}}}}a$ and bulk concentrations^36^. Other approaches have focused on 2-D flow field prediction^37^, mesh correction^21^, or dynamic compartment models which, while effective for large-scale heterogeneities, still rely on solving mass balances over average volumes^10^ and require previous hydrodynamic information for a single specific set of operating conditions. In contrast, our framework advances the level of spatial resolution by learning a direct mapping from global operating conditions to fully resolved 3-D concentration distributions. As a result, there are currently no directly comparable surrogates offering this level of volumetric granularity, and we strictly benchmarked against the underlying high-fidelity CFD simulations.

We deliberately restricted the scope of the current models. Training data was generated from single-phase CFD with a Multiple Reference Frame (MRF) treatment of the impellers (Methods)—currently the only practical route to assembling hundreds of large 3-D simulations, given available computational resources. No experimental validation was performed, and the study was restricted to a single vessel geometry and scale. The present surrogates should therefore not be interpreted as scale-invariant: application to geometrically similar vessels of different sizes would require additional CFD cases or retraining, because changes in scale can alter Reynolds number, power input per volume, turbulent length scales and compartment-to-compartment exchange. The surrogates also inherit the assumptions of the underlying CFD setup—including turbulence closure, MRF, Newtonian behavior, single-phase flow and simple Monod kinetics—and their errors are naturally concentrated in regions where those assumptions are most strained, such as high-shear impeller junctions. Accordingly, the present models should be interpreted as CFD-consistent emulators rather than experimentally validated reactor predictors; any systematic bias in the underlying CFD formulation would be propagated to the surrogate.

Methodologically, we position these surrogates as complementary to, rather than replacements for, classical reaction engineering, compartment modeling and mechanistic CFD. This complementarity is particularly relevant for CM-workflows: surrogate-predicted 3-D fields could be used to rapidly construct or update CMs across operating conditions, while CMs could provide dynamic cell-history simulations on top of the surrogate-derived spatial maps.

By learning a direct mapping to these 3-D gradients, our models provide CFD-consistent fields for the specified geometry at negligible computational cost. However, they do not replace detailed CFD or experiments when transient evolution, complex phase interactions, local extrema or explicit trajectory-wise cell histories are required^13^. The present framework also differs from PINNs-based CFD approaches. PINNs embed governing-equation residuals directly into training and can be valuable for data-sparse settings, inverse problems or local field reconstruction, including stirred-bioreactor applications^38^. In contrast, our objective was to emulate an existing CFD workflow over a large parametric operating space. This supervised strategy requires an upfront CFD database, but once trained, it enables millisecond-scale prediction without evaluating PDE residuals or rerunning CFD simulations. A key finding of this work is that successful emulation relies heavily on the representation of the target variable: direct modeling of raw concentration (${C}_{S}$) was hindered by its heavy right-skew, whereas the biologically bounded ratio ${q}_{S}/{q}_{S,\max }$ yielded stable training, proving that physics-informed data processing is as critical as model architecture.

Despite these restrictions, our findings hold substantial practical implications for bioprocess design. From a chemical engineering perspective, the surrogates—under pseudo-steady-state conditions—approximate the combined effects of turbulent mixing, convective–diffusive transport, and Monod kinetics as represented in the CFD training data. This capability enables rapid “virtual experimentation”, allowing engineers to perform systematic scans over different combinations of operating conditions in milliseconds. The observed inference speed ($\sim 10\,{{\rm{ms}}}$ per field on a single GPU)—representing a reduction of up to ${10}^{6}$-fold relative to the CFD baseline—places these models firmly in the real-time regime, unlocking their potential for integration into online set-point optimization loops and digital-twin frameworks^39^.

Looking ahead, we view this framework as a foundation that can be systematically extended by adding new input parameters and CFD simulations. As computational resources grow, larger databases^35^ incorporating sliding-mesh multiphase simulations^40^, parametrized geometries, non-Monod bio-kinetics, or non-Newtonian rheology would allow for the training of more generalizable models. For extensions to new kinetic regimes, transfer learning may reduce the required additional data by fine-tuning the present Monod-trained surrogate on a targeted set of CFD simulations rather than training a new model from scratch.

Furthermore, architectures with stronger spatial inductive biases (e.g., Graph NNs or Transformers) may reduce data requirements and improve extrapolation. Emerging work suggests that hybrid models, anchored in experimental measurements but expanded by synthetic data, offer a viable path toward reliable digital twins at scale^39,41^. In that context, we present this study as a critical first step: a demonstration that the coupling of hydrodynamics and biokinetics, as represented in the CFD training data, can be emulated in real-time at the level of fully resolved 3-D gradients, offering a realistic starting point for the next generation of spatially distributed data-driven bioprocess models.

## Methods

This study introduces a comprehensive methodology for developing various regression models—ranging from classical Multiple Linear Regression (MLR) to sophisticated NNs—specifically aimed at predicting spatial gradients in bioreactors. To illustrate the approach, we use a widely recognized bioreactor geometry: the Rushton-impeller stirred tank^1^. Nonetheless, the methodology is broadly applicable and can be adapted to other bioreactor configurations and geometries.

### Data generation: global and local variables, sampling methodology and simulation pipeline overview

Central to our approach is the careful distinction between global and local variables. Global variables describe the operating point of an entire fermentation run and therefore remain constant throughout a given simulation, whereas local variables capture the spatial state of every mesh element at that operating point.

#### Global variables

Each one of the ~760 simulations is characterized by a single vector of operating conditions, biokinetic parameters and fluid properties including the following variables: impeller speed, liquid height, feeding point coordinates (${x}_{{{\rm{feed}}}},\,{y}_{{{\rm{feed}}}},\,{z}_{{{\rm{feed}}}}$), volumetric substrate feed rate ${F}_{V}$, biomass concentration ${C}_{X}$, maximum substrate-uptake rate ${q}_{S,\max }$, half-saturation constant ${K}_{S}$, fluid density ρ and viscosity μ. These quantities define both the momentum and the reactive-transport problems solved by the CFD solver.

The complete case-level table containing the global input variables for all 760 CFD simulations is provided as Supplementary Data 1.

#### Local variables

Once the global set-point is fixed, the CFD solver returns a 3-D field over the computational mesh. For every cell, we record the centroid position ($x,{y},{z}$), its distance from the feeding point, the distance to the axis of rotation, and crucially, the substrate concentration ${C}_{S}$. The substrate concentration is the regression target for our surrogate models, while the other pointwise coordinates are predictors.

To properly sample the 11-D space of the global variables, we generated the combinatorial vectors with Latin Hypercube Sampling (LHS). LHS stratifies each factor into equally probable intervals and draws one value from every interval, thereby guaranteeing near-uniform coverage of every dimension with far fewer samples than simple random sampling^28,32^. For the feeding-point position, we used cylindrical coordinates for the sampling, to respect the vessel’s symmetry, and then converted the result to Cartesian coordinates before meshing (Supplementary Equation (1) in Supplementary Methods 1).

Each LHS sample is passed to an automated simulation pipeline that (i) builds the geometry, (ii) meshes the domain, (iii) launches a single-phase Reynolds-Averaged Navier-Stokes (RANS) calculation in the MRF formulation, and (iv) continues the calculation until a pseudo-steady state is reached. At that point, a snapshot of the substrate concentration field is exported and flattened: every mesh cell becomes one row in a dataframe whose first eleven columns repeat the global conditions and whose remaining columns hold the local predictors—spatial variables—plus the target ${C}_{S}$ or deterministic functions of it. A glimpse of the resulting structure makes the distinction unmistakable: for one simulation, the global columns are constant down the dataframe, while local columns vary from row to row.

Each LHS draw therefore represents a pseudo-steady state “frozen” instant along a possible fed-batch trajectory. By sampling many such process states across the design space (Table 1), the surrogate can learn to predict instantaneous 3-D substrate fields conditional on global state variables, without integrating a full transient CFD solution. In practical use, these global states could be supplied by measurements, soft sensors or macroscopic process models, as commonly done when coupling reactor-scale heterogeneity to reduced dynamic models such as compartment models^10,12^.

### Rushton-impeller tank geometry

**Fig. 6:**
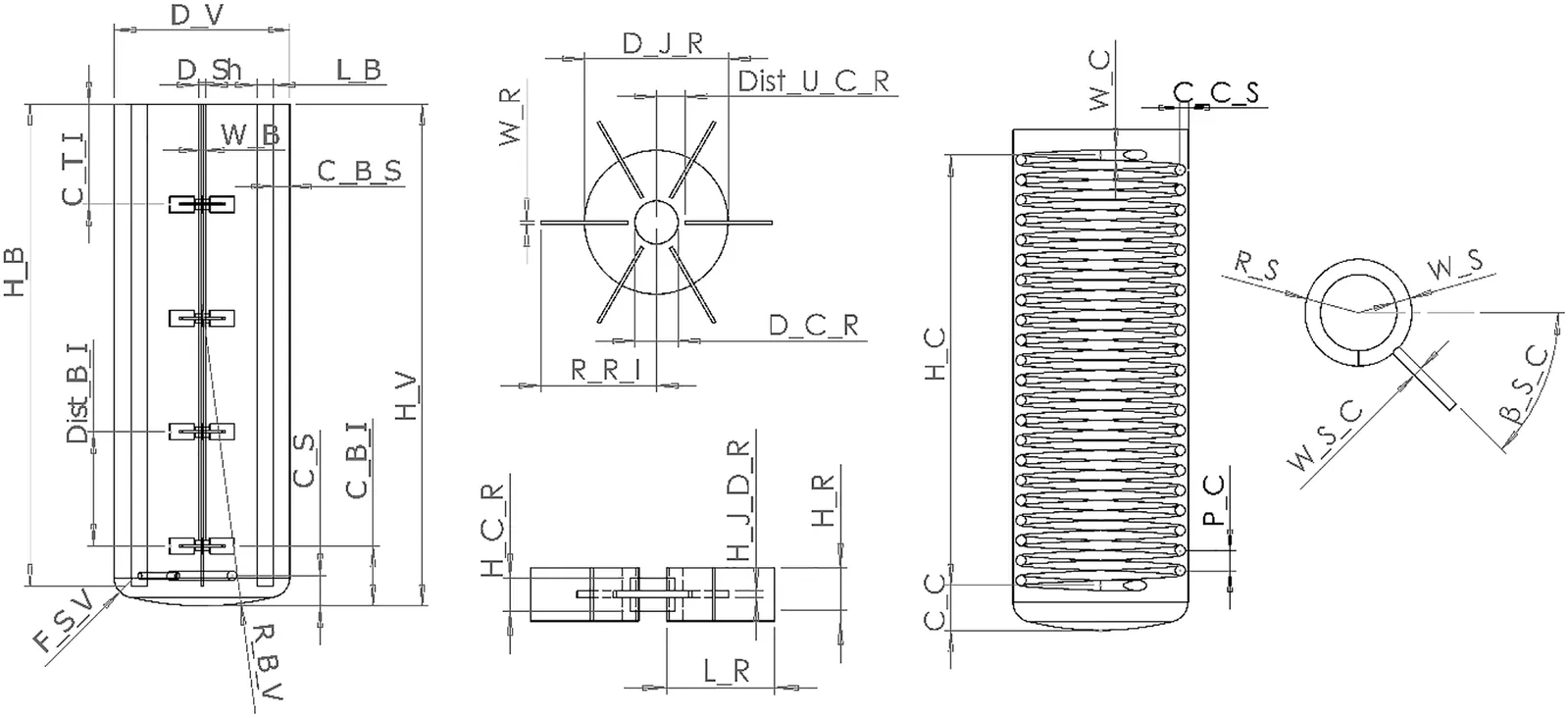
Schematic of the 170 m^3^ stirred tank with 4 Rushton impellers, ring sparger, 4 baffles and a helical cooling coil. The dimensional abbreviations correspond to the parameters reported in Supplementary Table 6.

The simulations employed a $170\,{{{\rm{m}}}}^{3}$ Rushton-tank geometry previously described in ref.^40^ and recreated from the original parametric SolidWorks® model for the present study (Fig. 6) with the following cylindrical coordinates:

1 $$\begin{array}{c}	0\le r\le 2.1875\,\left[{{\rm{m}}}\right]\\ 	0\le \vartheta \le 2\pi \\ 	0\le y\le 12.5\,\left[{{\rm{m}}}\right]\end{array}$$

The Rushton tank was designed with four Rushton impellers and baffles. Additionally, a helical cooling coil was placed around the impellers and baffles, and an O-ring sparger at the bottom of the tank. The sizes of the parametrized Rushton Impellers (disk, blades, clearance…) and the baffles were based on a standard bioreactor design^42^. The total volume of the tank is $170\,{{{\rm{m}}}}^{3}$.

The parametric tank dimensions, expressed relative to the vessel height where applicable, are provided in Supplementary Table 6.

### Meshing, fluid flow simulation and transport of diluted species

**Fig. 7:**
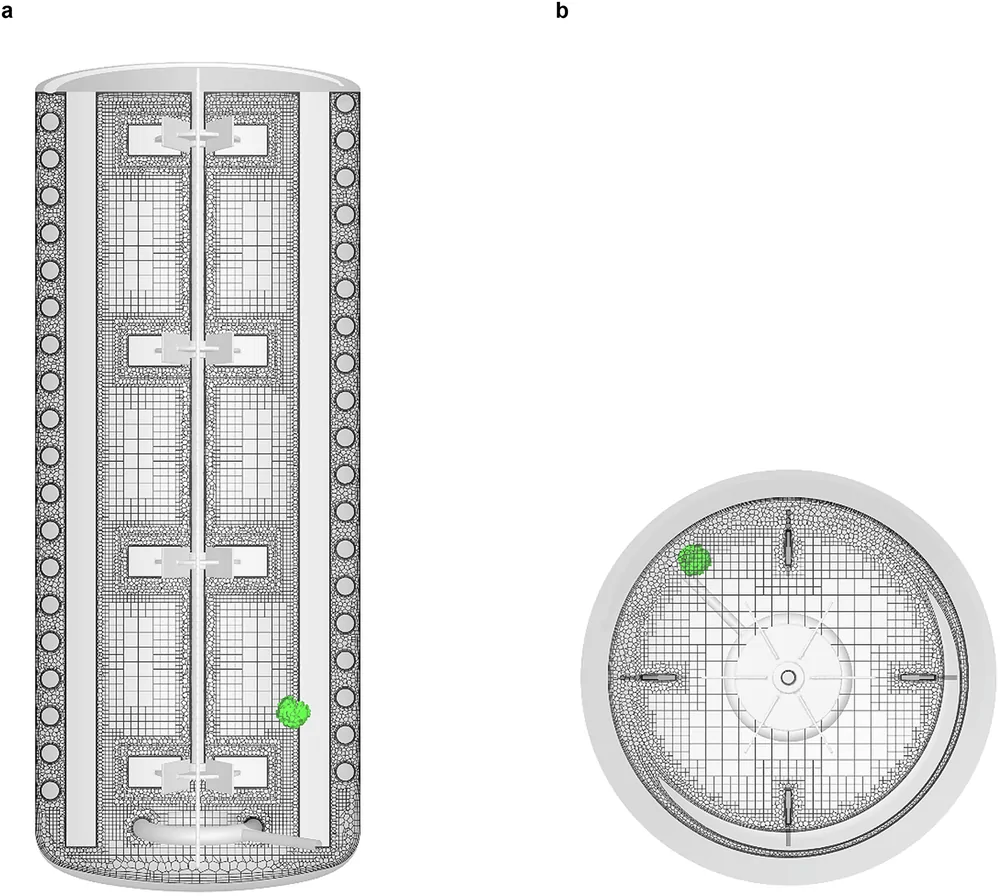
Representative poly-hexcore computational mesh (liquid height=10.65 m) and feeding-point location. **a** Front view of the computational mesh and internal reactor structures for a representative CFD case. **b** Top view of the same mesh. The green marker indicates the feeding-point location.

Each Latin-hypercube sample (e.g., ${{\rm{liquid\; height}}}$, feeding-point position) defined a distinct geometry; therefore, a fresh mesh was generated per case. We used a poly-hexcore mesh throughout, with a target resolution of $\sim 9\times {10}^{3}{{\rm{cells}}}\cdot {{{\rm{m}}}}^{-3}$ to keep cell counts comparable across cases with different liquid heights. This resolution was selected from a mesh-independence analysis performed on representative cases from the simulation campaign, which showed that the standard mesh reproduced the fine-mesh hydrodynamic diagnostics while substantially reducing computational cost (Supplementary Methods 3 and Supplementary Fig. 37). The entire pipeline, parametric meshing, solver setup, and post-processing, was automated via PyAnsys so that LHS parameters were injected directly into Ansys® Fluent prior to each run. Figure 7 shows the poly-hexcore mesh and feeding-point location for a representative CFD case (${{\rm{liquid\; height}}}=10.65{{\rm{m}}}$) in front and top views.

Ansys® Fluent was used to solve the continuity Eq. (2), momentum Eq. (3) and species-transport Eq. (4) for all simulations. The equations were solved in Reynolds-averaged form (RANS) with realizable k−ϵ turbulence model to solve the turbulent viscosity problem for the Reynolds stress tensor.

2 $$\nabla \cdot {{\bf{U}}}=0$$

3 $$\frac{\partial \left(\rho {{\bf{U}}}\right)}{\partial t}+\nabla \cdot \left(\rho {{\bf{U}}}{{\boldsymbol{\otimes }}}{{\bf{U}}}\right)=-\nabla P+\nabla \cdot \left(\left(\mu+{\mu }_{t}\right)\left(\nabla {{\bf{U}}}+{\left(\nabla {{\bf{U}}}\right)}^{{{\rm{T}}}}\right)\right)$$

The values of density (ρ) and viscosity (μ) were provided as input parameters in the multiple simulations designed with LHS within a DOE (Design of Experiment).

4 $$\left[\frac{\partial {C}_{S}}{\partial t}+\nabla \cdot \left({\mathbf{U}}{C}_{S}\right)=-\nabla \cdot \left[-\left({\mathscr D}+\frac{{\mu}_{t}}{{{\mathrm{Sc}}}_{t}}\right)\nabla {C}_{S}\right]+{S}_{S}\right]$$

The source term in Eq. (4) (general mass balance used in the CFD simulation) was computed using Monod (hyperbolic) kinetics, as defined in Eq. (5). The parameters, maximum uptake rate ${q}_{S,\max }$, affinity constant ${K}_{S}$ and biomass concentration ${C}_{X}$ were also input parameters in the LHS-DOE.

5 $${S}_{S}={-q}_{S,\max }\cdot \left(\frac{{C}_{S}}{{C}_{S}+{K}_{S}}\right)\cdot {C}_{X}$$

At the feeding point substrate was additionally provided via a source term. The volumetric feed source was one of the sampled global variables and was injected at the prescribed feed location.

The simulations used the well-validated multiple reference frame (MRF) with RANS approach^7^ to optimize CPU run-times. By using MRF, we assumed a pseudo-steady state at specific time points, which was valid for simulating fluid flow “snapshots” of the fermentation process. For transient simulations, however, macro-instabilities resulting from the turbulent interaction between impellers should be captured^43,44^. Using MRF, detailed interactions between the impeller and baffles were not fully captured, but the large separation minimized their impact compared to sliding mesh (SM) simulations^42^. Moreover, when solving for transport of species, the effects of the fluid flow became even further diluted, reducing the impact of the differences between using SM and MRF, especially in single-phase simulations^42^. All simulations used coupled velocity-pressure coupling and second-order discretization for all variables.

Similarly, we adopted a single-phase (liquid-only) formulation rather than an explicit two-phase model; this choice allowed us to reduce costs and enable large-scale data generation for training surrogates that reproduce the CFD results.

To verify that the automated CFD workflow produced physically plausible hydrodynamic behavior within the stated modeling assumptions, we performed an additional consistency check against classical stirred-tank power-number correlations. Specifically, CFD-derived effective impeller power numbers were compared with literature values for fully turbulent Rushton impellers in representative operating cases. This analysis was intended as a hydrodynamic sanity check of the numerical workflow, rather than as a complete experimental validation of the CFD model. We emphasize that the objective of this study was not to develop or validate a new high-fidelity CFD framework, but to evaluate whether machine-learning surrogates can accurately reproduce CFD-predicted 3-D substrate fields at greatly reduced computational cost. Accordingly, the surrogate models should be interpreted as fast emulators of the underlying RANS/MRF CFD simulations, and they inherit the assumptions and limitations of that numerical formulation. The hydrodynamic consistency check is reported in Supplementary Methods 4 and Supplementary Fig. 38.

When performing all simulations with the different parameters, sometimes a steady state was never reached, due to the accumulation of substrate (i.e., extremely low biomass concentration and very high feeding rates). In these cases, the simulations were repeated with new parameters to avoid reaching unrealistic substrate concentrations inside the reactor.

### Data scaling and transformation

Before training any model (MLR, other ML models, and NNs), data preprocessing was performed using feature scaling techniques to ensure that all variables contribute equally to the learning process. Specifically, the dataset, both local and global variables, was standardized so that each feature has mean zero and unit variance, Eq. (6):

6 $${x}_{{{\rm{std}}}}=\frac{x-\bar{x}}{\sigma }$$

Standardizing the data improves convergence of gradient-based optimization algorithms by ensuring that features are on a similar scale, leading to faster and more stable learning. It also prevents features with larger magnitudes from disproportionately influencing the model, thereby enhancing overall model performance. Algorithms that rely on distance calculations (Support Vector Regression, k-NN) particularly benefit from standardized features^45^.

For the original simulated response variable from the CFD simulations, ${C}_{S}$, multiple transformations were applied to facilitate prediction by de-skewing, normalizing, or scaling the data. We considered the following response variable transformations:

7 $${q}_{S}/{q}_{S,\max }=\frac{{C}_{S}}{{C}_{S}+{K}_{S}}$$

This maps ${C}_{S}$ to the dimensionless Monod saturation in $\left[{\mathrm{0,1}}\right]$, used as a proxy for “cell performance”.

8 $${{\rm{qt}}}\left({C}_{S}\right)={\Phi }^{-1}\left(\frac{{{\rm{rank}}}\left({C}_{S}\right)}{n+1}\right)$$

The quantile transformation of ${C}_{S}$ uses the inverse of the normal cumulative distribution function (CDF), ${\Phi }^{-1}$ to map the original values onto a normal distribution^46^. Finally, a logarithmic transformation (${\log }_{10}\left({C}_{S}+\epsilon \right)$) with a small offset ϵ>0 (to handle near-zero values) was used to address right skew typical of bioreactor concentrations.

### Dimensionality reduction study (PCA)

Principal Component Analysis (PCA) was performed on the standardized features to explore potential correlations among variables and reduce the problem dimensionality by identifying redundant variables. PCA transforms correlated variables into a smaller number of uncorrelated principal components. This process performs the eigen-decomposition of the covariance matrix of the centered data matrix. The first principal component (${{\rm{PC}}}1$) is the projection onto the leading eigenvector and captures the maximum variance, with subsequent components orthogonal to the previous ones^47^.

The variance explained by each principal component was analyzed to determine the necessary number of components. The loadings of each variable on these components were examined to identify the most significant contributors to data variation.

### Regression models

#### Multiple linear regression models

Multiple linear regression (MLR) models predict a dependent variable by combining various predictors. Specifically, they employ a linear equation, as shown in Eq. (9), which sums the products of coefficients and their respective features, adjusted by an intercept. Here, ${e}_{i}$ denotes the residual error (typically assumed zero-mean, normally distributed and homoscedastic). This model is constructed and refined using a training dataset.

9 $${y}_{i}={\beta }_{0}+{\beta }_{1}{x}_{i1}+\ldots+\,{\beta }_{p}{x}_{{ip}}+{e}_{i}$$

Table 2 summarizes six distinct multiple linear regression (MLR) techniques utilized in this study. Each technique employs a different loss function to address specific challenges within regression analysis. Robust regression techniques reduce sensitivity to outliers, regularization methods prevent overfitting when numerous variables are involved, and combined approaches of polynomial terms with regularization facilitate the modeling of non-linear relationships while curtailing the complexity induced by excessive features.

**Table 2:** Summary of MLR methods used in this paper to predict CFD data

Type of linear regression	Name	Description	Loss / Objective function	Hyperparameters tuned	Reference
Standard	Standard	General-purpose linear regression for predicting outcomes based on multiple predictors. Suitable for datasets without significant multicollinearity or outliers.	${\min }_{\beta }\left\{\frac{1}{2n}{\sum }_{i=1}^{n}{\left({y}_{i}-{\beta }_{0}-{\sum }_{j=1}^{p}{\beta }_{j}{x}_{{ij}}\right)}^{2}\right\}$	-	^55^
Regularization	Lasso	Shrinks some regression coefficients exactly to zero by applying an absolute value penalization term. Useful for feature selection and handling high-dimensional datasets.	${\min }_{\beta }\left\{\frac{1}{2n}{\sum }_{i=1}^{n}{\left({y}_{i}-{\beta }_{0}-{\sum }_{j=1}^{p}{\beta }_{j}{x}_{{ij}}\right)}^{2}+\lambda {\sum }_{j=1}^{p}\left|{\beta }_{j}\right|\right\}$	Regularization parameter: λ	^56^
	Ridge	Shrinks regression coefficients by applying a coefficient squared penalization term. Useful when predictors are highly correlated.	${\min }_{\beta }\left\{\frac{1}{2n}{\sum }_{i=1}^{n}{\left({y}_{i}-{\beta }_{0}-{\sum }_{j=1}^{p}{\beta }_{j}{x}_{{ij}}\right)}^{2}+\lambda {\sum }_{j=1}^{p}{{\beta }_{j}}^{2}\right\}$	Regularization parameter: λ	^57^
	Elastic net	Combines Lasso and Ridge benefits. Useful for datasets with correlated predictors and when feature selection is needed.	${\min }_{\beta }\left\{\frac{1}{2n}{\sum }_{i=1}^{n}{\left({y}_{i}-{\beta }_{0}-{\sum }_{j=1}^{p}{\beta }_{j}{x}_{{ij}}\right)}^{2}\right.$ $\left.\,+\lambda \left(\alpha {\sum }_{j=1}^{p}\left|{\beta }_{j}\right|+\frac{1-\alpha }{2}{\sum }_{j=1}^{p}{{\beta }_{j}}^{2}\right)\right\}$	Regularization parameter: λ Mixing parameter: α	^58^
Robust	Huber	Robust to outliers. Suitable for datasets with potential outliers that could impact standard linear regression coefficients.	${\min }_{\beta }\frac{1}{n}{\sum }_{i=1}^{n}{{{\mathcal{l}}}}_{\delta }\left({y}_{i}-{\beta }_{0}-{\sum }_{j=1}^{p}{\beta }_{j}{x}_{{ij}}\right)$ Where ${{{\mathcal{l}}}}_{\delta }$ is the Huber loss function defined by: $${\ell}_{\delta}(a)=\left\{\begin{array}{ll}\frac{1}{2}a^{2},\hfill& {\mathrm{for}}\ |a|\leq\delta\\ \delta\left(|a|-\frac{1}{2}\delta\right),& {\mathrm{for}}\ |a| > \delta\end{array}\right.$$	Threshold parameter: δ	^59^
Polynomial + Regularization	Polynomial + Lasso	Models complex relationships by fitting higher-degree polynomials. Ideal for reducing overfitting and selecting significant features in large datasets.	${\min }_{\beta }\left\{\frac{1}{2n}{\sum }_{i=1}^{n}{\left({y}_{i}-{\beta }_{0}-{\sum }_{j=1}^{p}{{\beta }_{j}{x}_{{ij}}}^{({{\rm{poly}}})}\right)}^{2}\,+\lambda {\sum }_{j=1}^{p}\left|{\beta }_{j}\right|\right\}$	Not tuned	^56,60^

Different models differ in the loss function and hyperparameters needed to be tuned during cross validation (CV) to find the optimum ones.

#### Best subset analysis for MLRs, ${{\rm{AIC}}}$ and ${{{\rm{R}}}}_{{{\rm{adj}}}}^{2}$

A best-subset search with MLR was performed to locate the smallest predictor set that still explains the response accurately. Models were ranked with the adjusted coefficient of determination ${{{\rm{R}}}}_{{{\rm{adj}}}}^{2}$ and the Akaike information criterion (${{\rm{AIC}}}$). Both metrics embody the same philosophy: they reward goodness-of-fit but penalize excess predictors, thereby discouraging overfitting and favoring parsimony (Supplementary Note 1). Plain mean squared error (${{\rm{MSE}}}$), which lacks any complexity penalty, was therefore not used as it would always select the most complex model.

The ${{\rm{AIC}}}$, which balances model complexity (number of estimated parameters, p) and fit (likelihood, L), is calculated as:

10 $${{\rm{AIC}}}=2{{\rm{p}}}-2{{\mathrm{ln}}}\left(\hat{L}\right)$$

The likelihood function for linear regression is described as:

11 $$\hat{L}={\left(2\pi {\sigma }^{2}\right)}^{-n/2}\cdot \exp \left(-\frac{{{\rm{RSS}}}}{2{\sigma }^{2}}\right),$$

where ${{\rm{RSS}}}$ represents the residual sum of squares or sum of squared differences of observed response minus predicted values.

Additionally, the adjusted ${{{\rm{R}}}}^{2}$, (${{{\rm{R}}}}_{{{\rm{adj}}}}^{2}$), which trades off reductions in the residual sum of squares against the number of predictors, was used for the best-subset analysis. This metric is calculated as:

12 $${{{\rm{R}}}}_{{{\rm{adj}}}}^{2}=1-\left(1-{{{\rm{R}}}}^{2}\right)\frac{n-1}{n-p-1},{{\rm{where}}}{{{\rm{R}}}}^{2}=1-\frac{{{\rm{RSS}}}}{\sum {\left({y}_{i}-\bar{y}\right)}^{2}}$$

As we are applying these metrics to MLR models (Table 2), the number of features equals the number of predictors.

Loosely speaking, ${{{\rm{R}}}}^{2}$ evaluates the proportion of variance in the dependent variable that is predictable from the independent variables. ${{{\rm{R}}}}_{{{\rm{adj}}}}^{2}$ is preferable for assessing model performance in regression analysis as it adjusts model complexity through the number of predictors, avoiding overfitting.

#### Other ML regression models

In our study, we went beyond traditional MLRs to include other ML regression models to better capture the complex patterns in the data. Well-known MLR limitations with non-linear relationships and interactions prompted us to explore a variety of non-linear models, including neighbor-based approaches, ensemble methods, decision trees, and support vector machines (SVMs).

Neighbor models are based on local data point proximity and similarity, ideal for our dataset’s spatial relationships. Ensemble methods, such as random forest (through bagging) and boosting, improve prediction stability and accuracy by pooling multiple models’ outputs, effectively reducing noise and outlier impacts. Decision trees provide interpretable means to segment data, capturing variable interactions, while SVM models can project data into higher-dimensional spaces to model complex non-linear relationships.

These techniques were instrumental in developing a robust framework capable of outperforming MLR, offering deeper insights and enhanced accuracy. Details and applications of these methods are summarized in Table 3.

**Table 3:** Summary of machine learning techniques used for predicting CFD data

ML-Regression Technique	Name (regressors)	Usage	Loss / Objective function/ Mathematical formulation	Hyperparameters tuned	Reference
Neighbors	k−Nearest neighbors (k-NN)	Uses proximity to predict values or categories, effective for datasets where close cases have similar outcomes.	Prediction: ${\hat{y}}_{i}=\frac{1}{k}{\sum }_{{j}\in {N}_{k}\left({x}_{i}\right)}{y}_{j}$	Number of neighbors: k	^61^
Trees	Decision trees (DT)	Splits data into homogeneous sets to base its predictions. Straightforward for interpretation and works well with mixed data types.	${\min }_{\Theta }{\sum }_{j=1}^{M}{\sum }_{{i}\in {R}_{j}}{\left({y}_{i}-{\hat{y}}_{{R}_{j}}\right)}^{2},$ Prediction: ${\hat{y}}_{{R}_{j}}=\frac{1}{\left|{R}_{j}\right|}{\sum }_{{i}\in {R}_{j}}{y}_{i}$	Maximum depth of trees Minimum samples split Minimum samples leaf	^62^
Ensemble	Random forest regression (RFR)	Averages multiple decision trees to improve prediction stability and accuracy, great for feature importance determination.	Objective (per tree): ${\min }_{{\theta }_{t}}\frac{1}{\left|{S}_{t}\right|}{\sum }_{{i}\in {S}_{t}}{\left({y}_{i}-{f}_{t}\left({x}_{i};{\theta }_{t}\right)\right)}^{2},$ Hyperparameter tuning: ${\min }_{\Theta }\frac{1}{n}{\sum }_{i=1}^{n}{\left({y}_{i}-{\hat{y}}_{i}\left(x,\Theta \right)\right)}^{2},$ Forest prediction: $\hat{y}\left(x\right)=\frac{1}{T}{\sum }_{t=1}^{T}{f}_{t}\left(x,\Theta \right)$	Number of trees: T Number of features Minimum samples split Minimum samples leaf	^63^
	Gradient boosting regression (GBR)	Builds models sequentially to correct previous errors, excels in accuracy and handling complex, non-linear patterns.	${\min }_{\Theta }{\sum }_{i=1}^{n}{{\mathcal{l}}}\left({y}_{i},{F}_{m-1}\left({x}_{i}\right)+{\Theta }_{m}{h}_{m}\left({x}_{i}\right)\right),$ With, ${{\mathcal{l}}}\left(y,\hat{y}\right)={\left(y-\hat{y}\right)}^{2}$ Learning recursive process with residuals: ${r}_{i.m}={y}_{i}-{F}_{m-1}\left({x}_{i}\right)$ Prediction: $\hat{y}\left(x\right)={F}_{T}\left(x\right)={F}_{0}\left(x\right)+{\sum }_{m=1}^{T}{\lambda }_{m}{h}_{m}(x)$	Number of trees: T Learning rate Maximum depth of trees	^64^
Support vector machine	Support vector regression (SVR)	Fits error within a certain threshold to predict continuous values, robust against outliers and highly effective in handling high-dimensional spaces.	${\min}_{w,b,\xi,{\xi }^{*}}\frac{1}{2}{\left|w\right|}^{2}+C{\sum }_{i=1}^{n}\left(\xi_i+{\xi }_{i}^{*}\right),$ Subject to constraints: ${y}_{i}-\left(w\cdot {x}_{i}+b\right)\le \zeta+{\xi }_{i}$ $\left(w\cdot {x}_{i}+b\right)-{y}_{i}\le \zeta+{\xi }_{i}^*$ ${\xi }_{i},\,{\xi }_{i}^{*}\ge 0$	Kernel type Regularization parameter: C Kernel coefficient: γ	^65^

These methods vary in their mechanisms, such as decision rules or ensemble strategies, and each requires specific hyperparameters to be optimized through cross-validation for best performance

The tested hyperparameter values are reported in Supplementary Tables 7 and 8, and the optimum configurations used for final training are reported in Supplementary Table 9.

#### Feed-forward neural networks

Feed-forward neural networks were employed to model complex non-linear relationships between inputs and outputs. For regression tasks, these networks utilized a single output neuron to predict continuous values^48^. They were trained using back-propagation with mean squared error (${{\rm{MSE}}}$) to minimize prediction errors, adjusting weights through gradient descent. Due to their adaptability, these neural networks proved effective in handling complex and high-dimensional regression challenges across various domains^49^ (Supplementary Fig. 43).

Given that the CFD simulations were conducted using various fermentation setups, each data point includes both local and global variables. Global variables remain constant throughout a dataset derived from the same simulation. We denote the local feature vector by $l\in {{\mathbb{R}}}^{{d}_{l}}$ and the global feature vector by $g\in {{\mathbb{R}}}^{{d}_{g}}$.

To account for the local and global variables in the data, we propose different input layer topologies for the NNs. First, we define a baseline—“**Standard-NN**”—feed-forward NN described as:

13 $${h}_{1}=\phi \left({{{\bf{W}}}}_{1}\left(l\oplus g\right)+{{\bf{b}}}\right),\,{h}_{n}=\phi \left({{{\bf{W}}}}_{n}\left({h}_{n-1}\right)+{{\bf{b}}}\right){{\rm{;\; with}}}:n=2,\ldots,{{\rm{L}}}$$

In the input layer, the local features (or local variables, l) are concatenated (⊕ operator) to the bioreactor global features (or global variables, g) of each data point, and then multiplied by a matrix of learnable weights, ${{\bf{W}}}$, and summed to corresponding learnable bias vectors, ${{\bf{b}}}$. A nonlinearity ϕ (e.g., ReLU) is applied to this result and forwarded through a number (hyperparameter) of fully-connected layers of the same nature. A final output layer produces the prediction.

A second input layer topology was considered that changed the input layer as defined below, called hereafter “**Attention-NN**”^50^.

14 $${l}_{1} 	=\phi \left(l{{\bf{L}}}{{\boldsymbol{+}}}{{{\bf{b}}}}_{{{\boldsymbol{l}}}}\right)\\ {g}_{1} 	=\phi \left(g{{\bf{G}}}{{\boldsymbol{+}}}{{{\bf{b}}}}_{{{\boldsymbol{g}}}}\right)\\ {s}_{1} 	={{\rm{softmax}}}\left(\tanh \left({{\bf{S}}}{g}_{1}+{{{\bf{b}}}}_{s}\right)\right)\\ {h}_{1} 	=\left[{l}_{1};\left({l}_{1}\odot {s}_{1}\right)\right]$$

Where, ${{\bf{G}}}$, ${{\bf{L}}}$ and ${{\bf{S}}}$ were learnable weight matrices, all with the same output dimension (hyperparameter of the NN). The ⊙ operator denotes the element-wise product. This way, a latent ${s}_{1}$ vector is learned that identifies the relation between the local features and global features, acting as attention weights.

A third topology for the input layer without the residual connection was also considered—“**Modified Attention-NN**”:

15 $${l}_{1} 	=\phi \left(l{{\bf{L}}}{{\boldsymbol{+}}}{{{\bf{b}}}}_{{{\boldsymbol{l}}}}\right)\\ {g}_{1} 	={{\rm{softmax}}}\left({{{\bf{G}}}}_{2}\tanh \left(g{{{\bf{G}}}}_{1}+{{{\bf{b}}}}_{{g}_{1}}\right)+{{{\bf{b}}}}_{{g}_{2}}\right)\\ {h}_{1} 	={l}_{1}\odot {g}_{1}$$

Finally, a fourth topology for the input layer was tested to avoid re-computing the global latent vector for all data points—“**Focused Attention-NN**”:

16 $${l}_{1} 	=\phi \left(l{{\bf{L}}}{{\boldsymbol{+}}}{{{\bf{b}}}}_{{{\boldsymbol{l}}}}\right)\\ {g}_{1} 	=\phi \left({{{\bf{G}}}}_{1}\dot{g}+{{{\bf{b}}}}_{\dot{g}}\right)\\ {s}_{1} 	={{\rm{softmax}}}\left({{{\bf{G}}}}_{3}\tanh \left({{{\bf{G}}}}_{2}{g}_{1}+{{{\bf{b}}}}_{{g}_{2}}\right)+{b}_{{g}_{3}}\right)\\ {h}_{1} 	={l}_{1}\odot {{\rm{Broadcast}}}\left({s}_{1}\right)$$

In this case a latent vector ${s}_{1}$ is learned from all the global features, but this time only once per batch, $\dot{g}$. The operator ${{\rm{Broadcast}}}\left(\cdot \right)$ expands ${s}_{1}$ across local feature dimension for each data point in the batch.

All the attention-based NNs are followed by a Standard-NN with subsequent layers using the architecture as one of the tuned hyperparameters.

All neural networks were implemented in PyTorch^30^. For regularization, ${{\rm{BatchNorm}}}$ layers^51^ were placed after each linear layer and before nonlinearity. Dropout layers^52^, with rates treated as hyperparameters during optimization, were applied after all linear layers, except for ${l}_{1}$ (and ${g}_{1}$ where applicable) in Eq. (15), where Dropout was omitted.

All the NNs targeted the variable ${q}_{S}/{q}_{S,\max }$, which always stays between 0 and 1, so a sigmoid output layer was used to keep predictions in-range.

The set of hyperparameters used for tuning the NNs is defined in Supplementary Table 8.

### Validation strategy and hyperparameter optimization

From the complete set of 760 CFD simulations, we randomly separated 200 simulations ($26\,\%$) as an independent test set, held aside exclusively to evaluate final model generalization capabilities. This set was not used for model fitting, hyperparameter optimization, early stopping, architecture selection or any other model-development decision, thus ensuring an unbiased evaluation of each model’s predictive performance under completely unseen operational conditions (global variables).

The remaining 560 simulations (74%) composed the development set, from which we derived all regression models and optimized hyperparameters. This development set underwent a two-stage process: first, a hyperparameter tuning phase—applicable to models featuring adjustable hyperparameters—followed by a final training step using the tuned models.

The hyperparameter search relied on a grouped (by simulation) 10-fold cross-validation (CV) applied to the 560-simulation development set. After a random shuffle at the simulation level, the simulations were partitioned into ten equally sized folds, each containing roughly 56 complete CFD runs (1$0\,\%$ of the development set). In every CV round, one fold served as the validation set while the remaining nine folds were merged into the training set. For each split, all per-run dataframes in the training (and validation) sets were concatenated into a single training (and validation) dataframe before model fitting and scoring. The procedure cycled until every fold had acted once as the validator, and the mean validation ${{\rm{MSE}}}$ across the ten rounds provided the performance score for each hyperparameter configuration.

Crucially, all mesh elements from the same given simulation were kept inside the same fold. Spatial fields within a single CFD run are strongly correlated; scattering those elements across training and validation subsets would leak information and underestimate error. Preserving whole simulations intact therefore prevents data leakage and mirrors the real-world scenario in which the surrogate is asked to predict an entirely new fermentation run rather than isolated fragments of one.

For ML models and regularized MLR variants—excluding polynomial-Lasso, which was not tuned—we adopted a grid search approach. Each combination of candidate hyperparameters was then assessed via the 10-fold CV procedure, and the configuration yielding the lowest mean ${{\rm{MSE}}}$ was selected as optimal.

Unlike classical ML and MLR-derived methods, NNs featured a significantly broader and continuous hyperparameter search space, demanding a more sophisticated approach. To address this, we utilized the tree-structured Parzen estimator (TPE), a Bayesian optimization algorithm implemented through the Optuna framework^53,54^, integrated within the PyTorch workflow^30^ (Supplementary Fig. 39). TPE iteratively constructs probabilistic models informed by past trials, categorizing hyperparameters into favorable or unfavorable regions based on their performance. This approach efficiently navigated the vast NN hyperparameter space—including the learning rate, network architecture, batch size, dropout rates and more—optimizing the hyperparameters to minimize the mean CV validation ${{\rm{MSE}}}$.

To justify data sub-sampling, we applied volume-weighted kernel density estimation (KDE), a non-parametric technique for estimating probability-density functions directly from data. Because each row in our dataframe represents a mesh element of differing physical size, every observation was weighted by its element volume ${V}_{i}$ so that the estimated density reflects the true spatial distribution, not the numerical density of cells. The weighted KDE is given by:

17 $${\hat{f}}_{H}\left(x\right)=\frac{1}{{\sum }_{i=1}^{n}{V}_{i}}{\sum }_{i=1}^{n}{V}_{i}{K}_{H}\left(x-{x}_{i}\right),$$

where ${K}_{H}$ denotes the kernel function (typically Gaussian) with bandwidth H, and ${x}_{i}$ represents individual observations from the dataset. By comparing KDE distributions for subsets of varying sizes against the original full dataset, we verified that small randomly drawn subsets maintained statistical fidelity to the original distributions (Supplementary Fig. 46). This verification gave confidence in our subsequent usage of significantly smaller datasets for hyperparameter tuning, thus enabling computational efficiency without sacrificing model accuracy.

Guided by the KDE analysis, we selected data fractions that balance computational cost and distributional fidelity for both hyperparameter tuning and the final fit: $5\,\%$ of datapoints for RFR, GBR and all NNs; $1\,\%$ for the computationally intensive SVR; and $10\,\%$ for DT and the MLR variants (Lasso, Ridge, Elastic-Net, Huber). Polynomial-Lasso, which was not tuned, used $10\,\%$ of the data at polynomial orders 2−3 and only $1\,\%$ at orders 4−5. After cross-validation and grid (or Optuna) search, the optimal hyper-parameters were frozen and each model was retrained on the same fraction, sampled from the full 560-simulation development set, so that the reported results reflect both predictive skill and the realistic computational budget of the algorithm. KDE plots in Supplementary Fig. 46 confirm that these sub-samples ($1-10\,\%$) preserve the marginal distributions of every variable, ensuring that no systematic bias is introduced by the reduction.

Finally, we gauged predictive accuracy and generalization on the held-out test set, an independent collection of simulations never seen during either hyperparameter tuning or final training. This unbiased evaluation serves as the common yardstick against which all surrogates are compared, using the mean squared error (${{\rm{MSE}}}$) and coefficient of determination (${{{\rm{R}}}}^{2}$) as primary metrics. The Akaike information criterion (${{\rm{AIC}}}$) requires an explicit likelihood and was therefore restricted to the best-subset MLR analysis. The adjusted coefficient of determination (${{{\rm{R}}}}_{{{\rm{adj}}}}^{2}$) was additionally reported in the best-subset and learning-curve analyses as a descriptive metric accounting for sample size and predictor count (see Supplementary Note 1).

Through this comprehensive validation and hyperparameter-optimization procedure, we ensured rigorous, unbiased and statistically sound model evaluation. Additional details on hyperparameter optimization, neural-network training dynamics and data subsampling are provided in Supplementary Methods 5–7; the statistical framework is described in the Supplementary Note 1.

## Supplementary information

Supplementary Information

Description of Additional Supplementary Files

Supplementary Data 1

Supplementary Software 1

Transparent Peer Review file

## Data Availability

The CFD simulation data generated and used in this study are publicly available through the BioBrAIn database under DOI 10.4121/27fd363b−1310-4a69-9635-aa50d9bb7823. The complete case-level table containing the global input variables and operating conditions for all 760 CFD simulations is provided as Supplementary Data 1. Numerical source data underlying all figures and tables in the main manuscript and Supplementary Information are provided with this paper in the accompanying Source Data file. No access restrictions apply to these data.

## Code availability

The scripts used to generate the data-based figures in the main manuscript and Supplementary Information from the processed Source Data are provided as Supplementary Software 1. The remaining custom code used for automated CFD data generation, data preprocessing, surrogate-model training, hyperparameter optimization and evaluation, together with the trained model files, is not deposited in a public repository so that reuse and redistribution can remain subject to appropriate institutional oversight. These materials are available to researchers affiliated with academic or non-profit research institutions for non-commercial research and reproducibility purposes. Requests should be directed to Víctor Puig-I-Laborda at viclab@dtu.dk and should include the requester’s institutional affiliation and intended research use. Access may be provided under a non-commercial research-use agreement. Researchers interested in reproducing, extending or building upon the methodology are also encouraged to contact the authors to discuss collaborative work.
