# Supplementary material for: Machine-learning surrogates enable rapid prediction of 3-D substrate gradients in industrial bioreactors
Source: Nat Commun. 2026 Oct 15;17:11636. doi: 10.1038/s41467-026-78273-7 (PMC13754804; doi:10.1038/s41467-026-78273-7)
Supplement: Supplementary file 1 [file 41467_2026_78273_MOESM1_ESM.pdf]

SUPPLEMENTARY INFORMATION

Machine-learning surrogates enable rapid prediction of 3-D substrate gradients in industrial bioreactors

Víctor Puig-I-Laborda[1,2,*], Jorge Carrasco Muriel[1], Andrea Castañeda Tena[1,2], Teddy Groves[1], Johan le Nepvou de Carfort[2], Ulrich Krühne[2], Krist V. Gernaey[1,2], Cees Haringa[3], Ignasi Puig-de-Dou[4], Lars Keld Nielsen[1,5,*]

[1] Novo Nordisk Foundation Center for Biosustainability, Technical University of Denmark, 2800 Kongens Lyngby, Denmark. The centre was succeeded at DTU by the Novo Nordisk Foundation Biotechnology Research Institute for the Green Transition (BRIGHT) in 2026.

[2] Department of Chemical and Biochemical Engineering, Technical University of Denmark, 2800 Kongens Lyngby, Denmark

[3] Department of Biotechnology, Faculty of Applied Sciences, Delft University of Technology, 2629HZ, Delft, The Netherlands.

[4] Escola Tècnica Superior d'Enginyeria Industrial, Departament d'Estadística i Investigació Operativa, Universitat Politècnica de Catalunya, Avinguda Diagonal, 647 08028 Barcelona, Spain

[5] Australian Institute for Bioengineering and Nanotechnology (AIBN), The University of Queensland, St.Lucia, QLD 4067, Australia

* Corresponding authors: viclab@dtu.dk, lars.nielsen@uq.edu.au

Contents

Supplementary Discussion 1. Target-variable representation and predictor-space structure

Supplementary Discussion 2. Best-subset analysis for multiple linear regression

Supplementary Discussion 3. Additional benchmarking across target representations

Supplementary Discussion 4. Residual diagnostics across surrogate-model families

Supplementary Discussion 5. Interpolation along one-dimensional operating-condition slices

Supplementary Discussion 6. Learning curve analysis for alternative target representations

Supplementary Methods 1. Cylindrical-to-Cartesian coordinate transformation

Supplementary Methods 2. Parametric geometry of the Rushton-impeller stirred tank

Supplementary Methods 3. Mesh-independence assessment

Supplementary Methods 4. Hydrodynamic consistency assessment against power-number correlations

Supplementary Methods 5. Cross-validation, hyperparameter search and model selection

Supplementary Methods 6. Neural-network training dynamics

Supplementary Methods 7. Data subsampling and distributional-fidelity assessment

Supplementary Note 1. Statistical foundations for model selection and generalization assessment

Supplementary References

This Supplementary Information provides additional analyses supporting target-variable selection, predictor-space characterization, surrogate benchmarking, residual behaviour, interpolation and data-efficiency assessment. It also provides expanded methodological details on reactor geometry, CFD verification, model selection, neural-network optimization, data subsampling and statistical assessment. Supplementary Discussions 1–6 complement the Results presented in the main article, whereas Supplementary Methods 1–7 and the Supplementary Note 1 expand the methodological descriptions provided in the main Methods.

Supplementary Discussions

Supplementary Discussion 1. Target-variable representation and predictor-space structure

To support selection of the primary response representation and assess redundancy in the predictor space, we compared the volume-weighted distributions of four target definitions and examined the pairwise correlation and principal-component structure of the standardized predictors.

The choice of target representation strongly affects the statistical structure of the surrogate-learning problem. The raw substrate concentration (C_S) exhibits a pronounced right-skewed distribution, reflecting the relatively small fraction of reactor volume exposed to high substrate concentrations compared with the larger low-concentration regions. We therefore compared four representations of the CFD-derived substrate field: raw C_S, $\log_{10} C_S$, the normalized uptake-based target $q_S/q_{S,\max}$, and the rank-based quantile transformation $\mathrm{qt}(C_S)$ (Supplementary Fig. 1). The logarithmic transformation reduces the long right tail, but its exponential inverse mapping can amplify prediction errors when transforming results back to concentration units. The quantile transformation produces an approximately Gaussian marginal distribution but similarly requires a strongly nonlinear inverse mapping. In contrast, $q_S/q_{S,\max}$ reduces the skewness of the response while remaining bounded and directly interpretable in biological terms, supporting its use as the primary target in the main analysis.

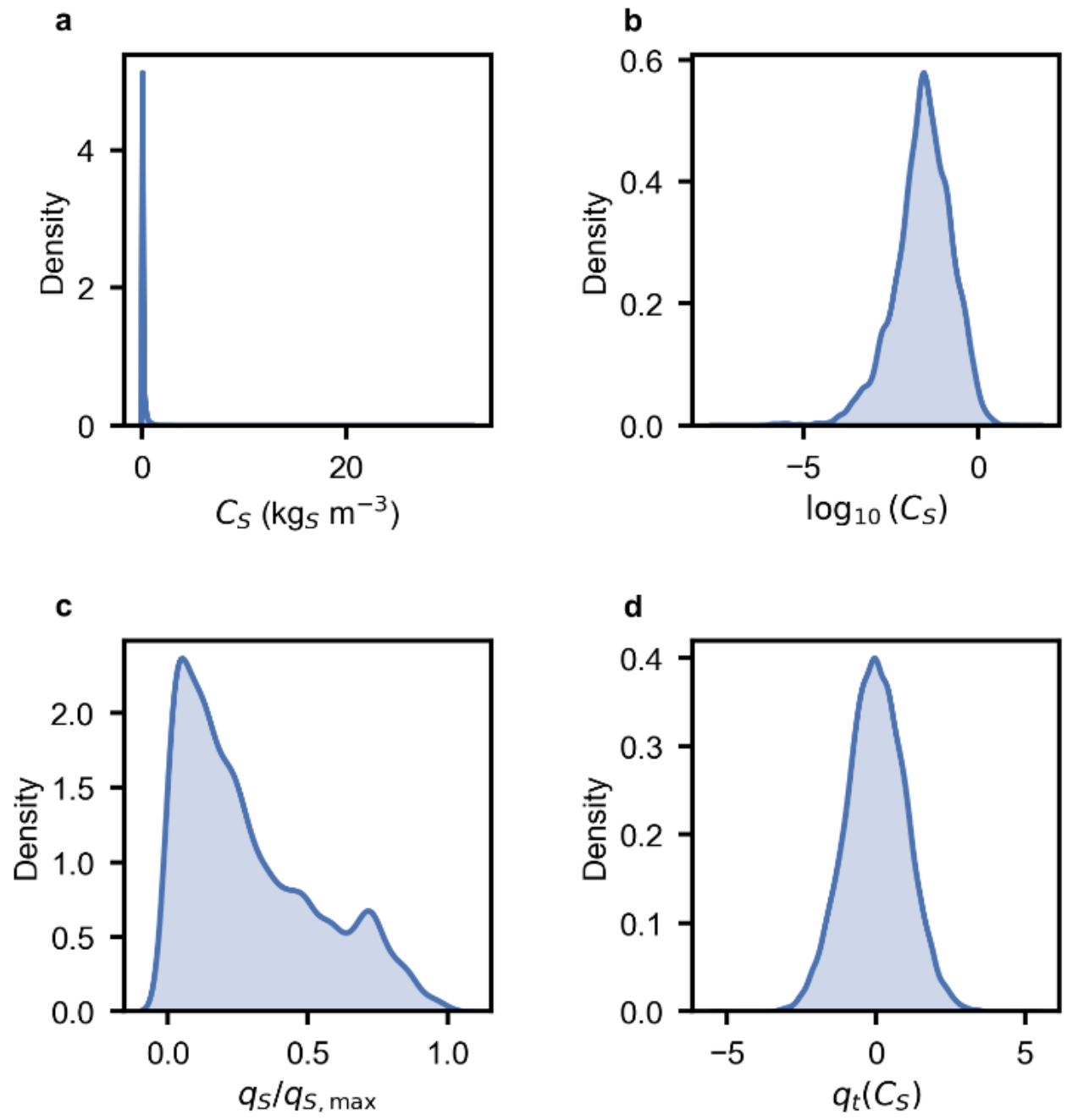

Supplementary Fig. 1. Volume-weighted distributions of alternative target representations. Kernel density estimates (KDEs) of the CFD-derived substrate field represented as raw substrate concentration C_S (a), $\log_{10} C_S$ (b), the normalized uptake-based target $q_S/q_{S,\max}$ (c) and the rank-based quantile transformation $\mathrm{qt}(C_S)$ (d). KDEs are weighted by CFD mesh-element volume according to equation (17) in the main Methods, so that the distributions represent physical reactor volume rather than numerical mesh density. The uptake-based target is bounded between 0 and 1, whereas $\mathrm{qt}(C_S)$ maps the original values onto a standard normal distribution.

Pairwise Pearson correlations were subsequently examined to characterize linear dependencies among the model inputs and the different target representations (Supplementary Fig. 2). Correlations among most input variables were weak, indicating limited linear multicollinearity across the sampled design space. The main structured dependency occurred among the axial variables and liquid height, consistent with the geometrical constraint imposed by the position of the free surface. As expected, the alternative target representations were more strongly correlated with one another because they are derived from the same underlying substrate-concentration field. Several gradient-related relationships with operating and spatial descriptors became more apparent after transformation of C_S, indicating that target transformation can expose linear structure that is less evident in the raw concentration. Conversely, variables entering the uptake relationship nonlinearly may exhibit relatively weak Pearson correlations even when they remain mechanistically relevant.

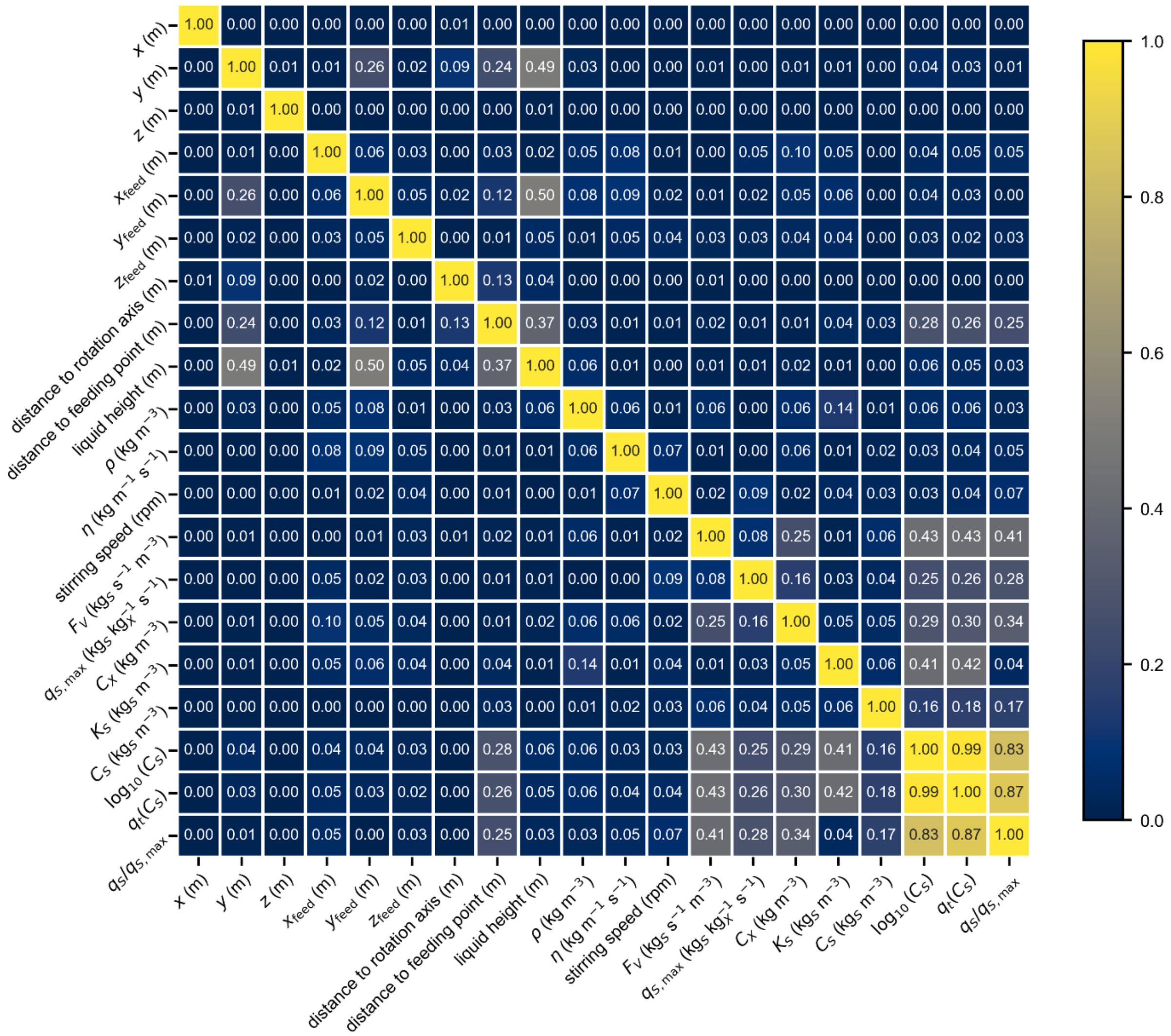

Supplementary Fig. 2. Correlation structure of model inputs and target representations. Pairwise Pearson correlation coefficients for the global operating variables, local spatial descriptors, and the alternative target representations C_S ,$\log_{10} C_S$,$q_S/q_{S,\max}$, $\mathrm{qt}(C_S)$.

Principal component analysis provided a complementary assessment of redundancy within the standardized predictor space. The cumulative explained variance increased approximately linearly with the number of retained components and showed no pronounced elbow, indicating that the variance was distributed across multiple dimensions rather than concentrated in a small number of principal components (Supplementary Fig. 3). Examination of the corresponding loadings showed that the first principal component was dominated by the axially constrained variables, consistent with the structured dependency identified in the correlation analysis (Supplementary Fig. 4). Subsequent components showed more broadly distributed loadings without a clear grouping of predictors. Together, these results indicate limited scope for substantial dimensionality reduction using PCA without discarding predictor information and are consistent with the broad coverage of the multidimensional operating space generated by the Latin hypercube sampling design.

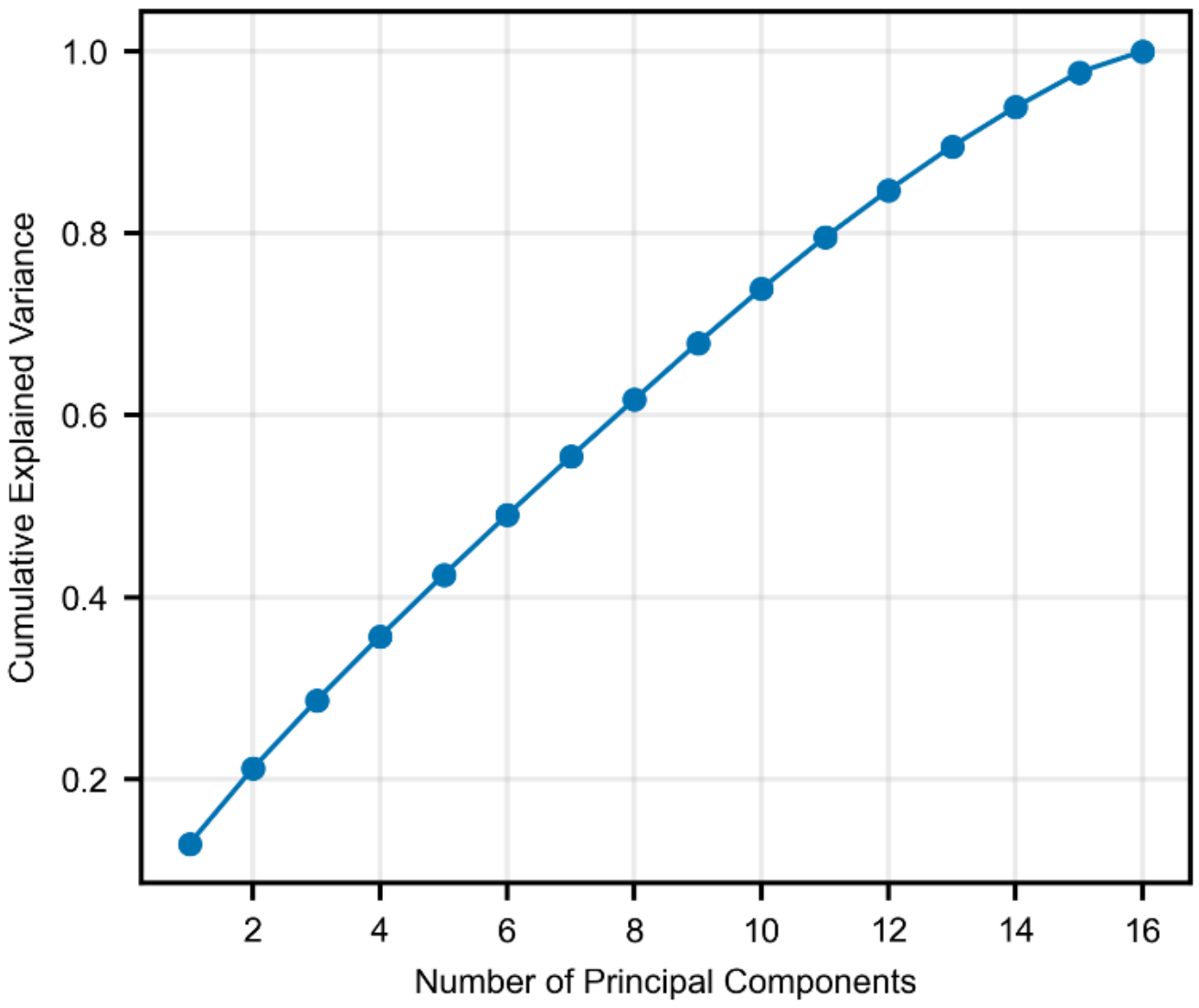

Supplementary Fig. 3. Cumulative variance explained by principal component analysis of the predictor space. Cumulative fraction of variance explained as a function of the number of retained principal components. Principal component analysis was performed on the standardized predictor variables.

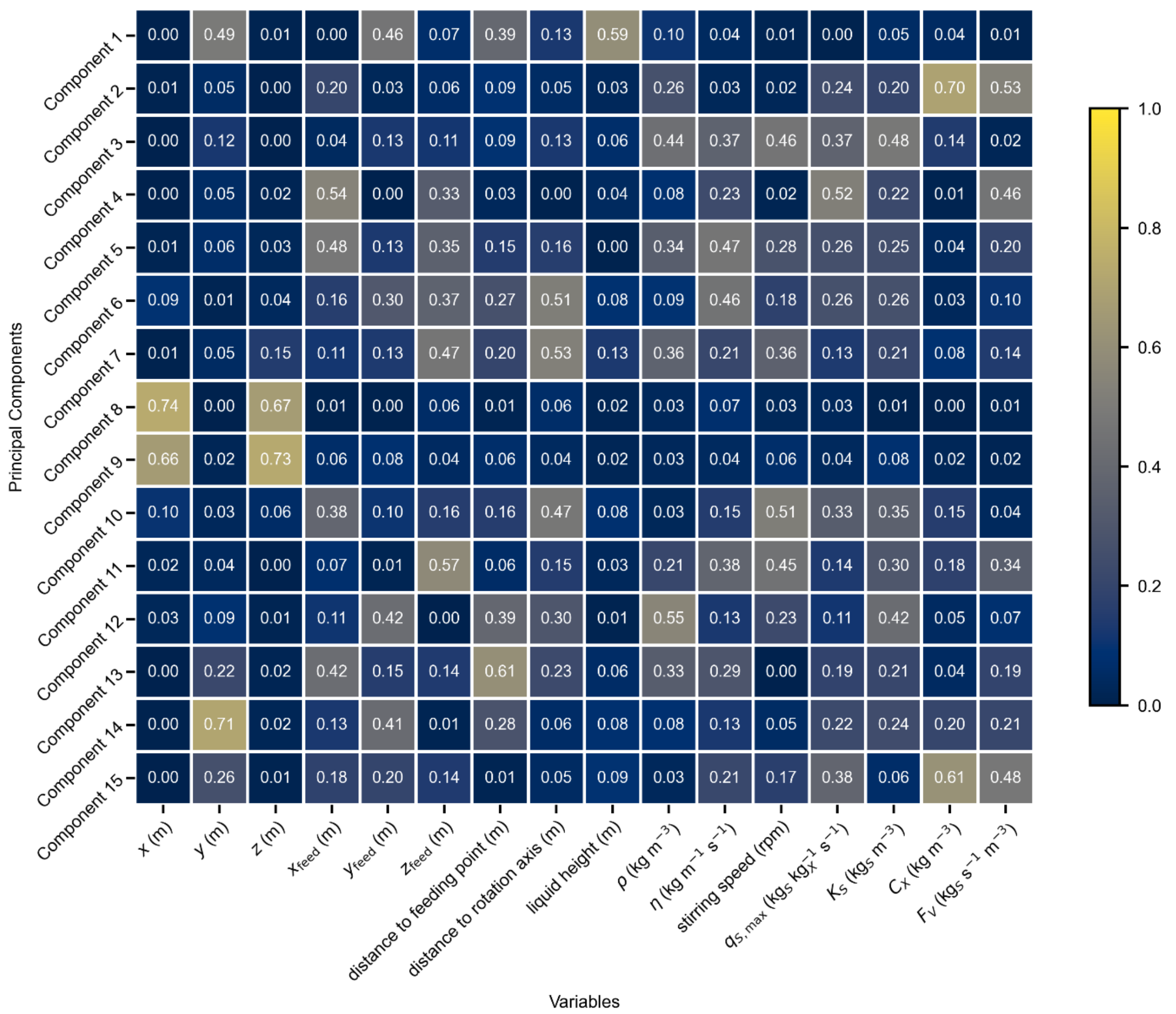

Supplementary Fig. 4. Predictor loadings across principal components. Absolute principal-component loadings of the standardized predictor variables. The heatmap shows the contribution of each predictor to each principal component, with larger absolute values indicating a stronger contribution to the corresponding component.

Supplementary Discussion 2. Best-subset analysis for multiple linear regression

Best-subset multiple linear regression (MLR) was applied separately to each target representation to identify compact predictor sets using the model-selection criteria described in the main Methods. Across target representations, the criteria generally converged on parsimonious subsets dominated by global operating conditions together with the local distance to the feeding point (Supplementary Tables 1–4). Raw spatial coordinates and feed coordinates appeared only sporadically among the highest-ranked subsets, indicating that the derived feed-distance descriptor captured much of the spatial information relevant to a linear representation of the response. For the log-transformed target, for example, the highest-ranked subset retained only a small number of predictors while maintaining a high adjusted R^2, further illustrating that comparable linear performance could be achieved with a compact predictor set.

For the normalized uptake-based target, the same general pattern was observed (Supplementary Table 1). Stirring speed was not retained in the highest-ranked subsets, despite its established

physical influence on mixing. This likely reflects the combination of response compression introduced by the normalized target and the limitations of a strictly linear model in representing non-linear interactions between stirring intensity, transport and uptake. Density and viscosity were also selected only sporadically, consistent with their relatively narrow sampled ranges and the highly turbulent regime represented in the CFD dataset. At the high impeller Reynolds numbers considered here, turbulent transport dominates molecular-viscosity effects, while the modest density variation has limited influence in the absence of buoyancy or compressibility effects. Within the explored operating space, their remaining linear contribution was therefore small compared with predictors directly describing feeding conditions and spatial proximity to the feed.

Supplementary Table 1. Best-subset screening of multiple linear regression for the uptake-based target $q_S/q_{S,\max}$. Predictor subset selected for the normalized uptake-based target using the model selection criteria described in the main Methods. Selected predictors are indicated in the corresponding columns.

Variables (features)	Best MSE	Best AIC	Best R^2_{adj}
x	–	–	–
y	X	–	X
z	X	–	–
x_{feed}		–	–
y_{feed}	X	X	X
z_{feed}	–	–	X
distance to feeding point	X	X	X
distance to rotation axis	X	X	–
liquid height		X	X
stirring speed	–	–	–
F_V	X	X	X
μ	–	–	X
ρ	–	–	–
C_X	X	X	X
K_S	–	–	–
$q_{S,\max}$	X	X	X

Supplementary Table 2. Best-subset screening of multiple linear regression for the target C_S. Predictor subsets selected for the untransformed substrate-concentration using the model-selection criteria described in the main Methods. Selected predictors are indicated in the corresponding columns.

Variables (features)	Best MSE	Best AIC	Best R^2_{adj}
x	X	–	–
y	–	–	X
z	–	–	–
x_{feed}	–	–	–
y_{feed}	X	X	X
z_{feed}	X	–	X
distance to feeding point	X	X	X
distance to rotation axis	X	X	X
liquid height	X	X	–
stirring speed	X	X	–

F_V	X	X	X
μ	X	X	–
ρ	–	–	–
C_X	X	X	X
K_S	X	X	X
$q_{S,\max}$	X	X	X

Supplementary Table 3. Best-subset screening of multiple linear regression for the target $\log_{10} C_S$. Predictor subsets selected for the log-transformed substrate-concentration using the model-selection criteria described in the main Methods. Selected predictors are indicated in the corresponding columns.

Variables (features)	Best MSE	Best AIC	Best R^2_{adj}
x	–	–	–
y	X	–	–
z	X	–	–
x_{feed}	–	–	–
y_{feed}	X	–	–
z_{feed}	–	X	X
distance to feeding point	X	X	X
distance to rotation axis	X	X	X
liquid height	X	X	X
stirring speed	X	X	–
F_V	X	X	X
μ	–	–	–
ρ	–	–	–
C_X	X	X	X
K_S	X	X	X
$q_{S,\max}$	X	X	X

Supplementary Table 4. Best-subset screening of multiple linear regression for the quantile-transformed target $\mathrm{qt}(C_S)$. Predictor subsets selected for the quantile-transformed substrate-concentration target using the model-selection criteria described in the main Methods. Selected predictors are indicated in the corresponding columns.

Variables (features)	Best MSE	Best AIC	Best R^2_{adj}
x	–	–	–
y	–	–	X
z	X	–	–
x_{feed}	–	–	–
y_{feed}	X	X	–
z_{feed}	X	X	–
distance to feeding point	X	X	X
distance to rotation axis	X	X	X
liquid height	X	X	X
stirring speed	X	–	–
F_V	X	X	X
μ	X	–	X
ρ	X	–	–
C_X	X	X	X

K_S	X	X	X
$q_{S,\max}$	X	X	X

Importantly, this analysis was intended to establish a parsimonious linear baseline rather than to identify a universally reduced predictor set. Variables with weak marginal or linear effects may still contribute through non-linear or higher-order interactions that cannot be represented by MLR. Consequently, the complete predictor set was retained for the non-linear surrogate models, which can down-weight weak or uninformative predictors through mechanisms such as regularization, tree-based selection or learned feature representations while still exploiting higher-order interactions among the operating and spatial variables.

Supplementary Discussion 3. Additional benchmarking across target representations

Model performance was evaluated primarily using the mean squared error (MSE), which was also used during model selection, while the coefficient of determination (R^2) provided a complementary assessment of the fraction of target variance captured by each surrogate. For the primary uptake-based target $\left({}^{q_S}/_{q_{S,\max}}\right)$, evaluation using R^2 produced a model ranking consistent with that obtained using MSE in the main benchmark (Fig. 2). The strongest-performing models retained high R^2 on the independent test set, whereas larger differences between development and test performance for several model classes indicated reduced generalization (Supplementary Fig. 5). The agreement between MSE and R^2 therefore indicates that the relative model ranking for the primary target is not specific to a single performance metric.

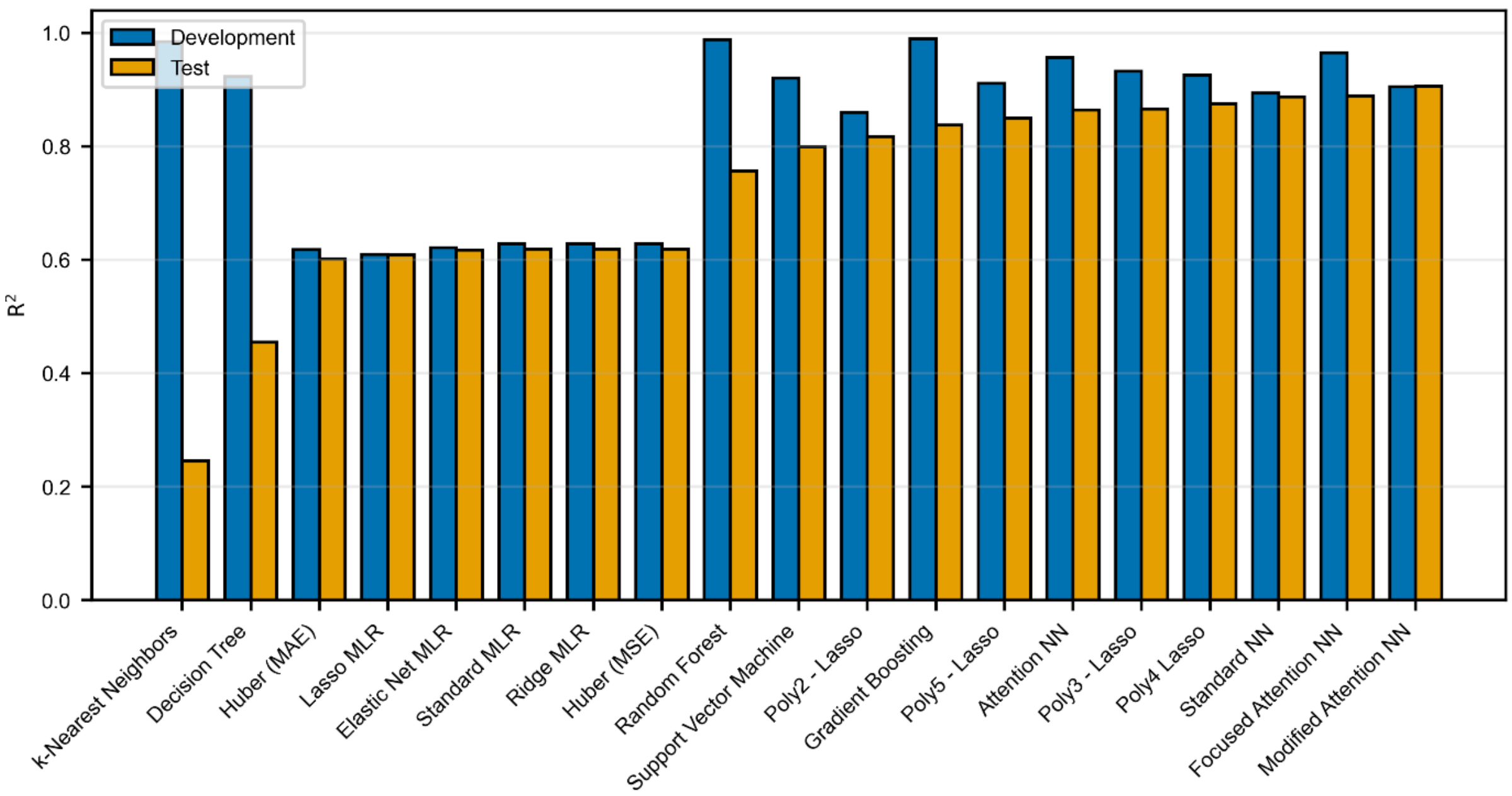

Supplementary Fig. 5. Complementary R^2 benchmarking for the uptake-based target $\left({}^{q_S}/_{q_{S,\max}}\right)$. Coefficient of determination ($\mathbf{R^2}$) for the benchmarked surrogate models evaluated on the development set and the independent test set comprising previously unseen CFD simulations. Models are ordered according to test-set performance. This analysis complements the **MSE**-based comparison presented in Fig. 2.

To assess the sensitivity of model performance to the choice of target representation, the same benchmarking framework was also applied to $\log_{10} C_S$, untransformed C_S, and the quantile-transformed target $\mathrm{qt}(C_S)$ (Supplementary Figs. 6–11).

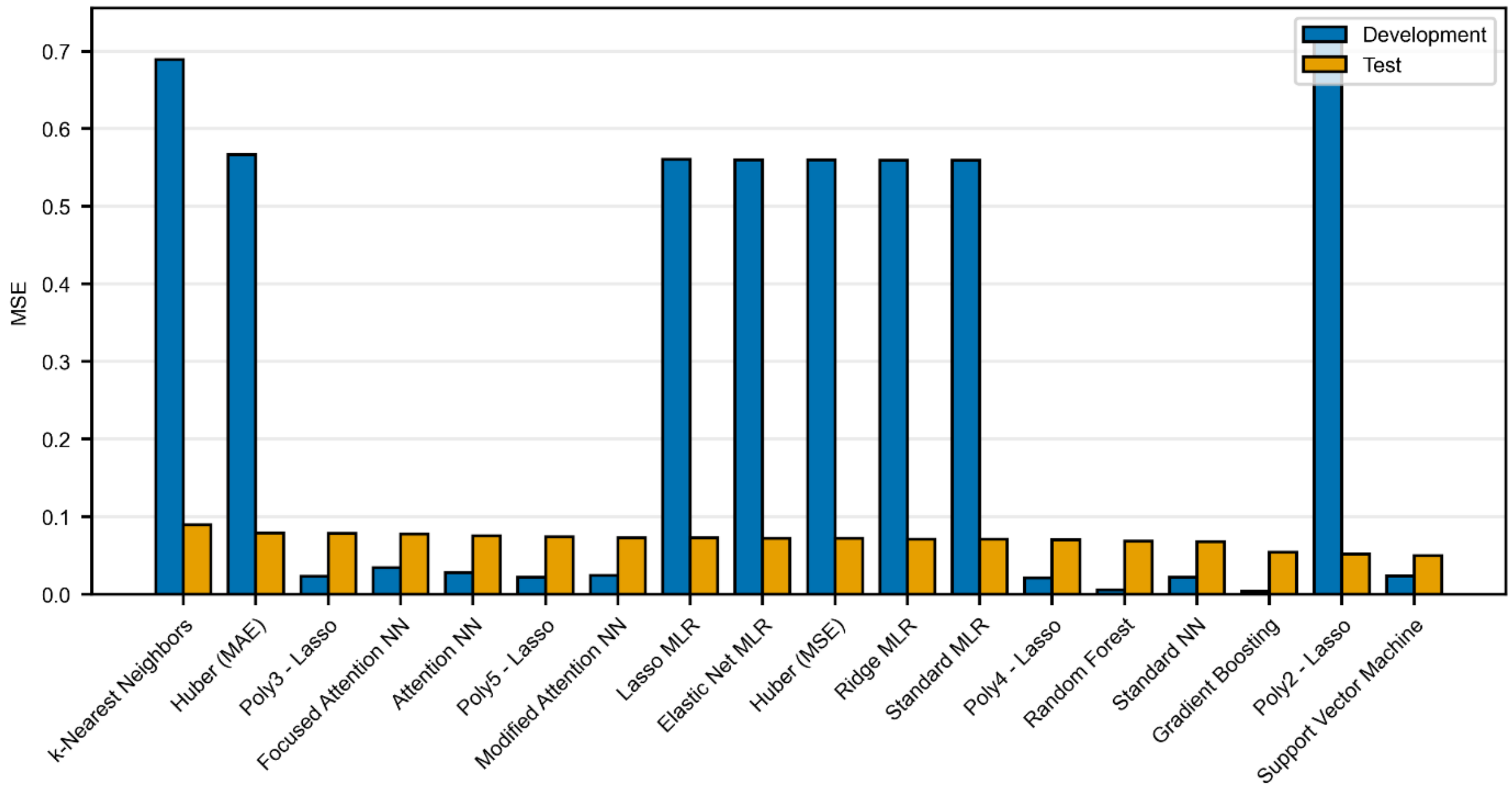

Supplementary Fig. 6. MSE benchmarking for log-transformed substrate concentration. Mean squared error (MSE) of the benchmarked surrogate models for the target $\log_{10} C_S$, evaluated on the development and independent test sets. Models are ordered according to test-set performance.

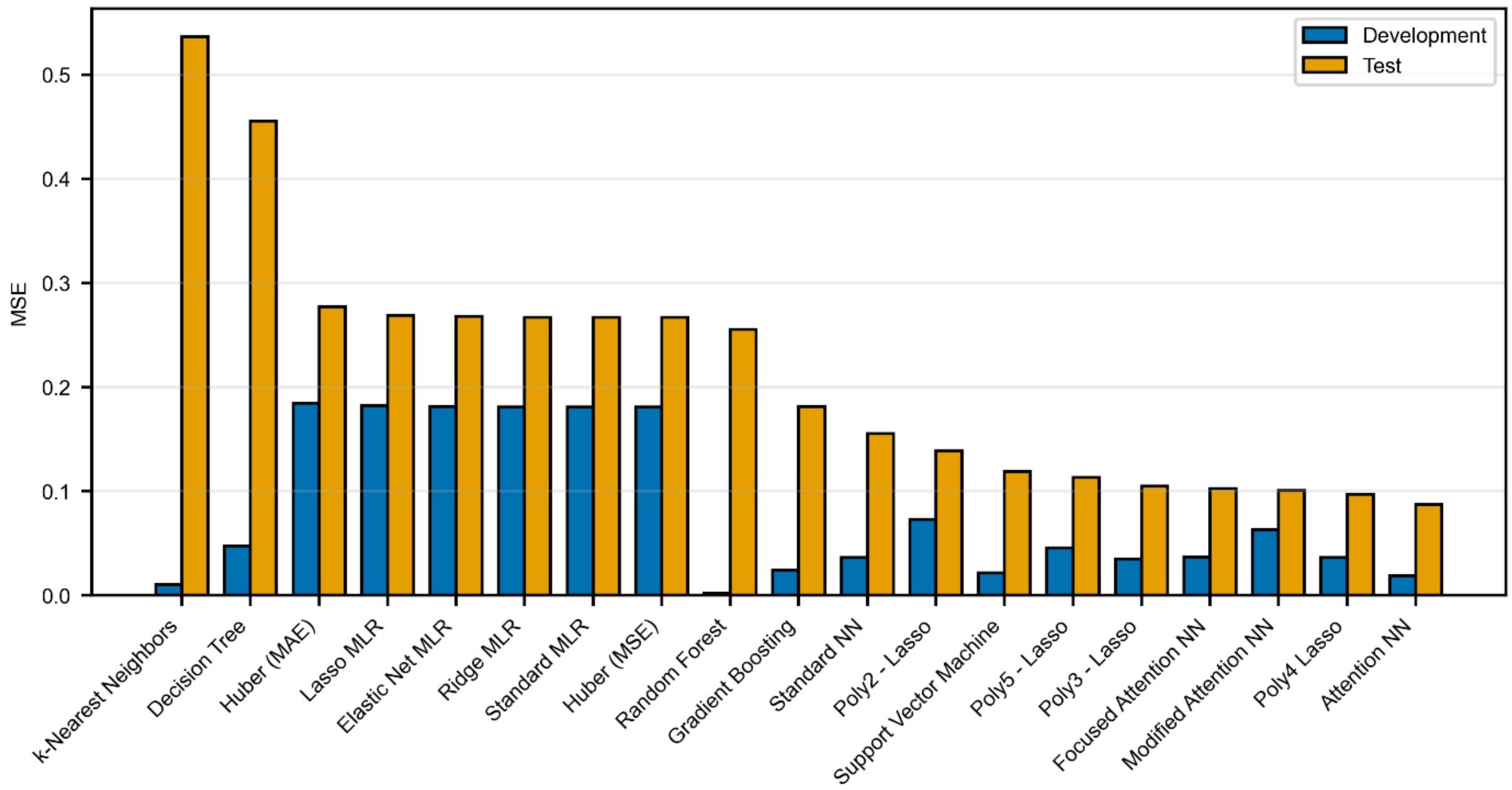

Supplementary Fig. 7. R^2 benchmarking for log-transformed substrate concentration. Coefficient of determination (R^2) of the benchmarked surrogate models for the target $\log_{10} C_S$, evaluated on the development and independent test sets. Models are ordered according to test-set performance.

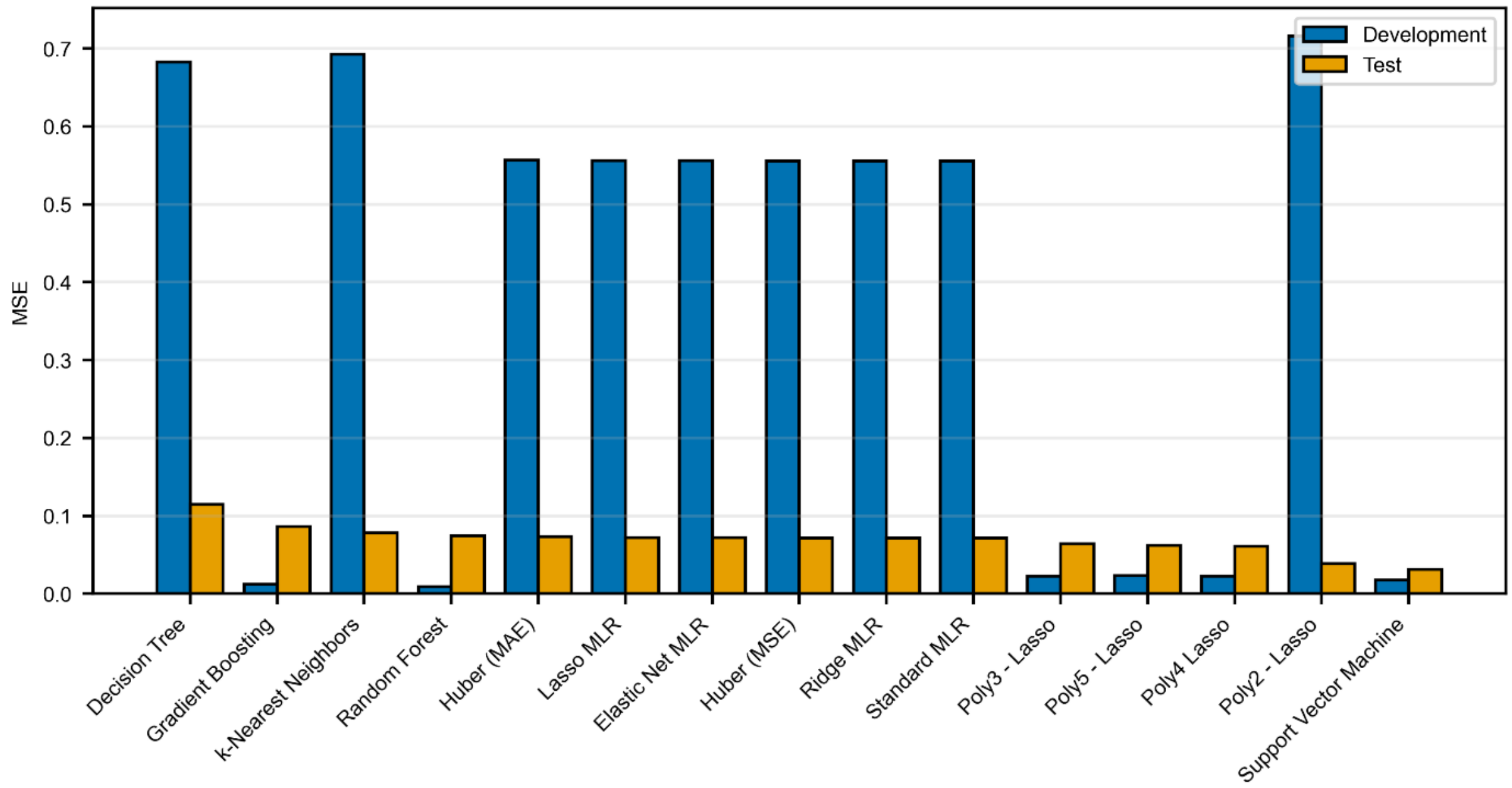

Supplementary Fig. 8. $\mathbf{MSE}$ benchmarking for substrate concentration. Mean squared error ($\mathbf{MSE}$) of the benchmarked surrogate models for the untransformed substrate-concentration target (C_S), evaluated on the development and independent test sets. Models are ordered according to test-set performance.

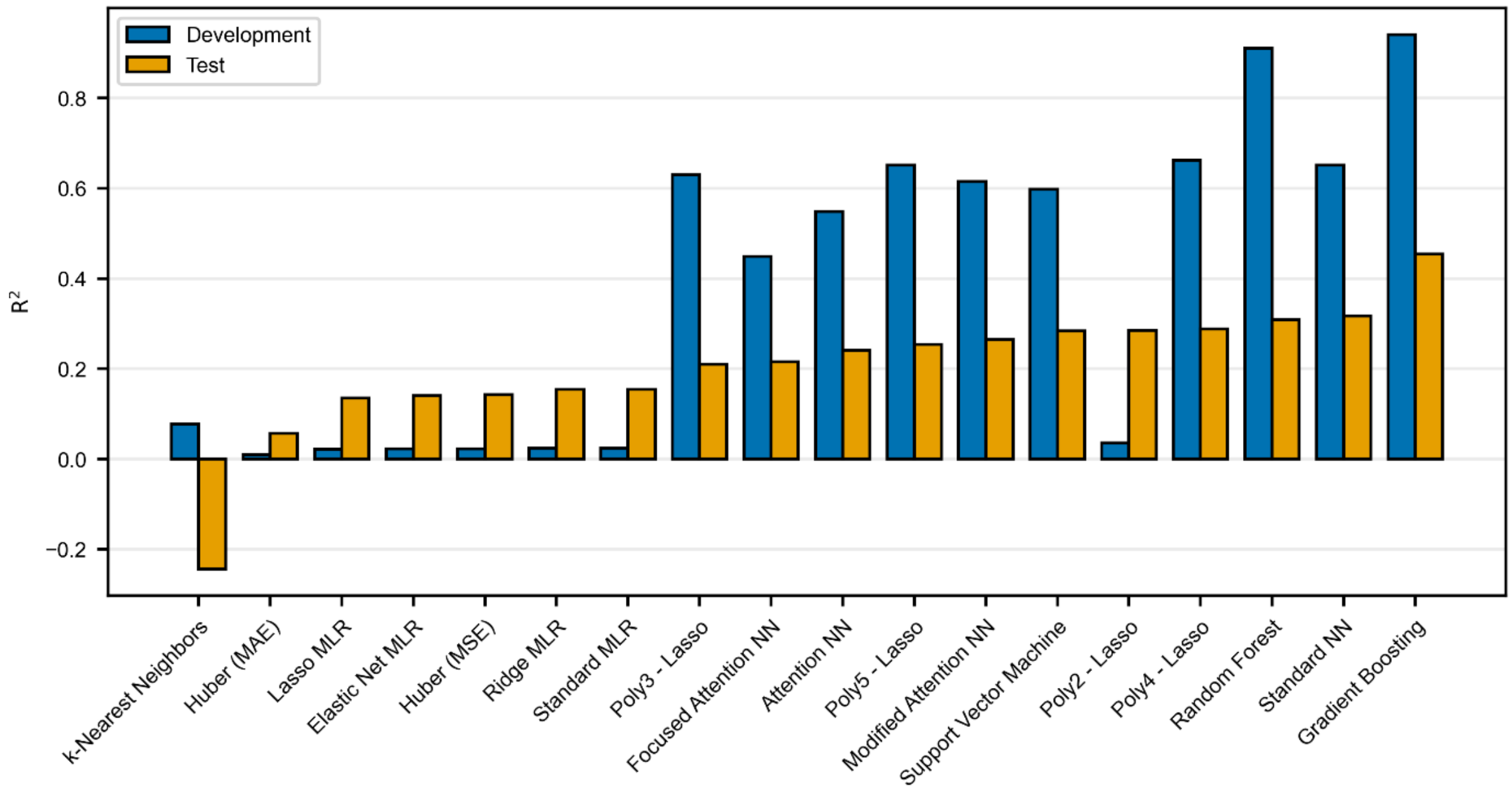

Supplementary Fig. 9. $\mathbf{R^2}$ benchmarking for substrate concentration. Coefficient of determination ($\mathbf{R^2}$) of the benchmarked surrogate models for the untransformed substrate-concentration target (C_S), evaluated on the development and independent test sets. Models are ordered according to test-set performance.

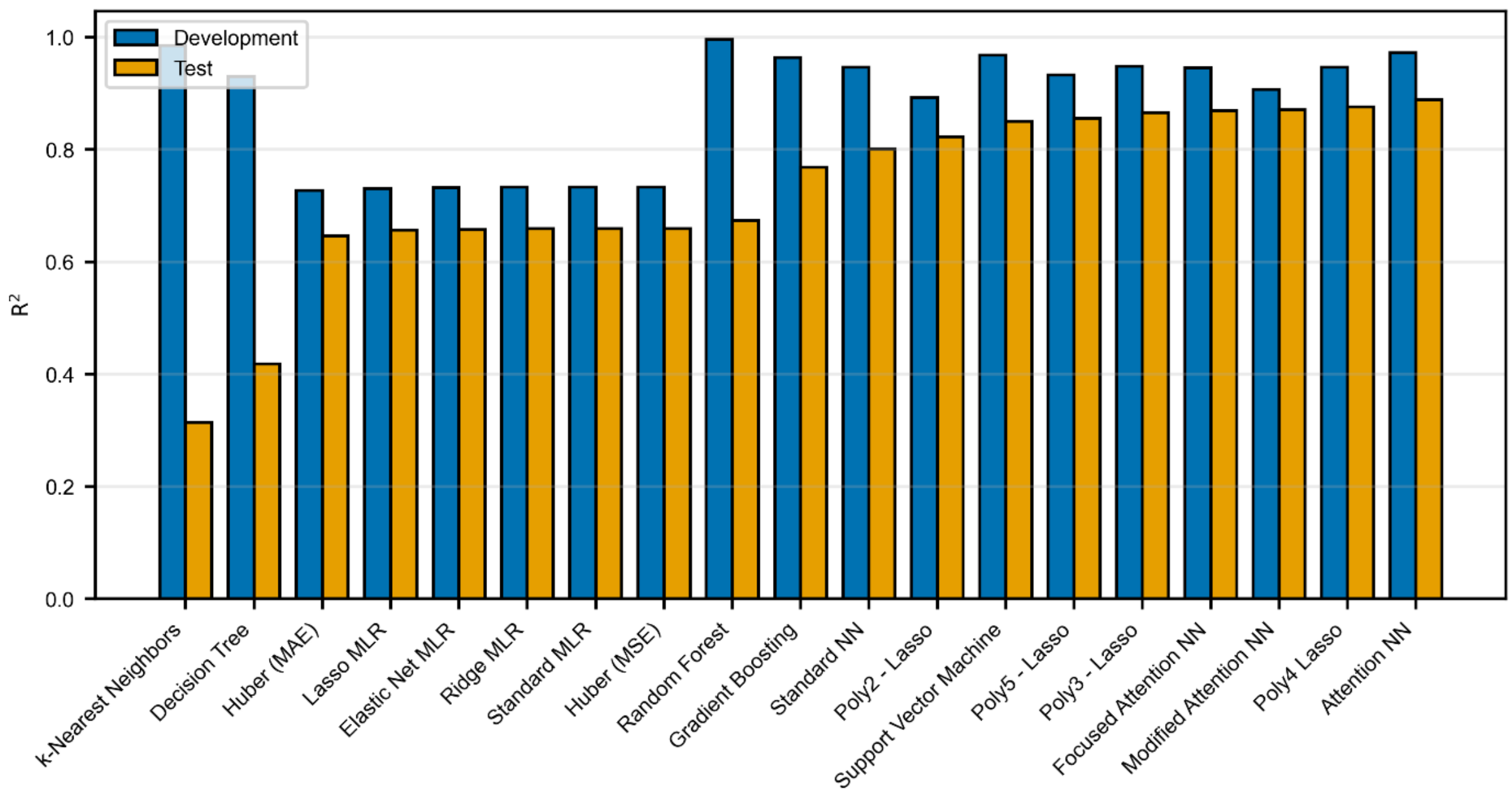

Supplementary Fig. 10. MSE benchmarking for the quantile transformed substrate concentration. Mean squared error () of the benchmarked surrogate models for the quantile transformed target ($\mathbf{qt}(C_S)$), evaluated on the development and independent test sets. Attention-based NN variants were not evaluated for this target. Models are ordered according to test-set performance.

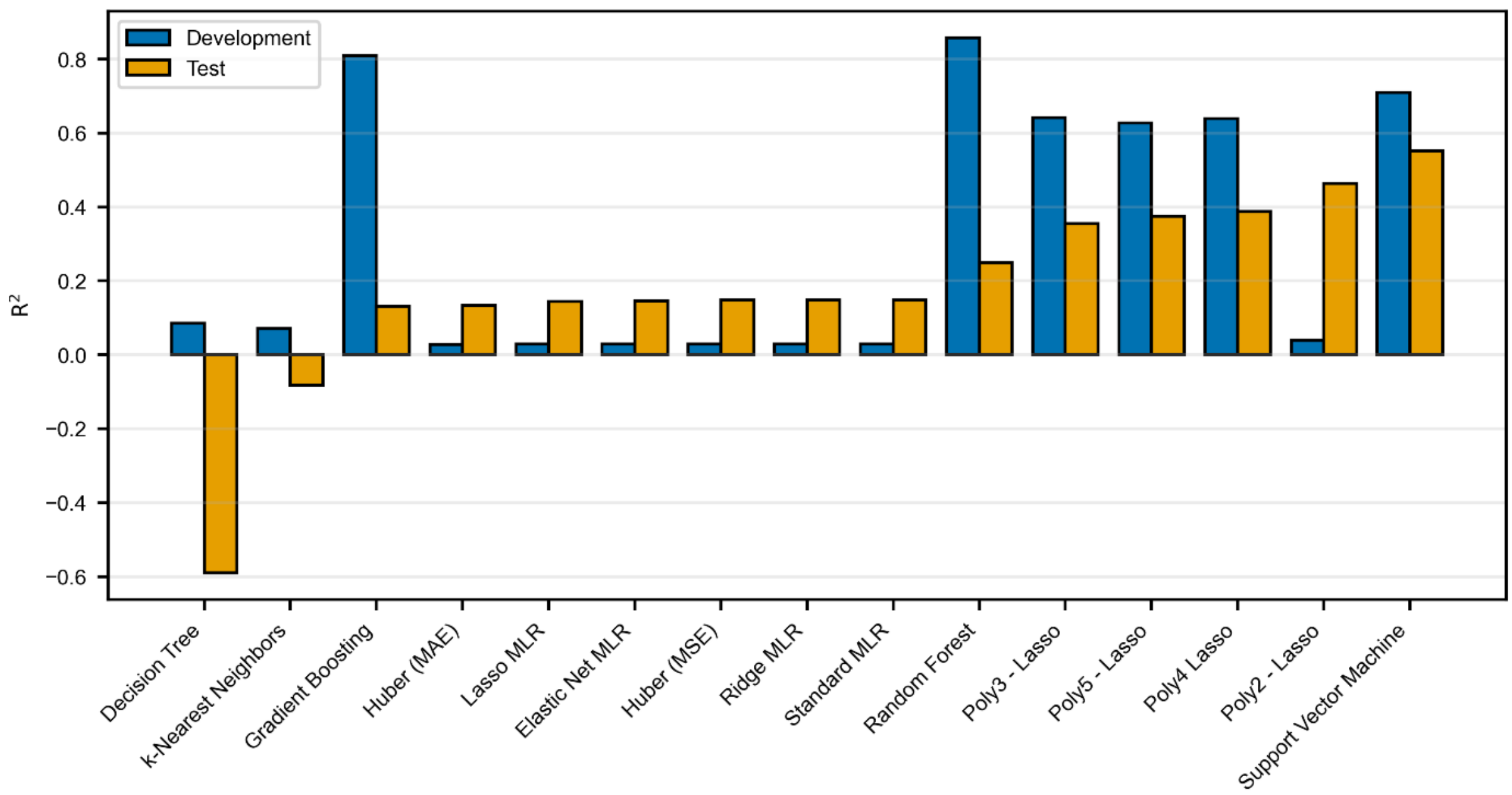

Supplementary Fig. 11. $\mathbf{R^2}$ benchmarking for the quantile-transformed substrate concentration. Coefficient of determination ($\mathbf{R^2}$) of the benchmarked surrogate models for the quantile-transformed target ($\mathbf{qt}(C_S)$), evaluated on the development and independent test sets. Attention-based NN variants were not evaluated for this target. Models are ordered according to test-set performance.

Supplementary Discussion 4. Residual diagnostics across surrogate-model families

Residual analysis provides a more detailed view of individual predictions, complementing aggregate metrics such as MSE and R^2 (Fig. 2 and Supplementary Figs. 5–11). Whereas global

scores summarize average prediction errors, residual diagnostics reveal where errors occur and whether systematic structure remains unmodelled. For each surrogate model, we evaluate: (i) predicted versus reference values, (ii) residuals, defined as $\varepsilon = y_{\mathrm{true}} - \hat{y}$, as a function of y_{true} to assess bias and heteroscedasticity, (iii) the residual-density distribution to identify heavy tails, and (iv) correlations between residuals and individual predictors to identify remaining error dependence. While strict linear inference assumes unbiased, homoscedastic and independent errors, here these patterns are used diagnostically to identify regions and conditions where predictive accuracy could still be improved. Residual diagnostics for the Modified-Attention NN and k-NN are shown in Supplementary Figs. 12 and 13, respectively, with the corresponding analyses for the remaining surrogate models shown in Supplementary Figs. 14–30.

Consistency between aggregate performance and residual structure was evident across model families. The best-performing Modified-Attention NN showed predictions concentrated close to the (1:1) line, with only a mild regression-to-the-mean tendency characterized by slight overprediction at low ${}^{q_S}/_{q_{S,\mathrm{max}}}$ and underprediction at high values (Supplementary Fig. 12). Its residual density was sharply concentrated around zero, although heavier tails indicated occasional larger errors, and correlations between residuals and individual predictors remained small. In contrast, the k-NN baseline exhibited substantially greater scatter, a pronounced fanning of the residual distribution with y_{true}, and stronger residual-predictor correlations (Supplementary Fig. 13).

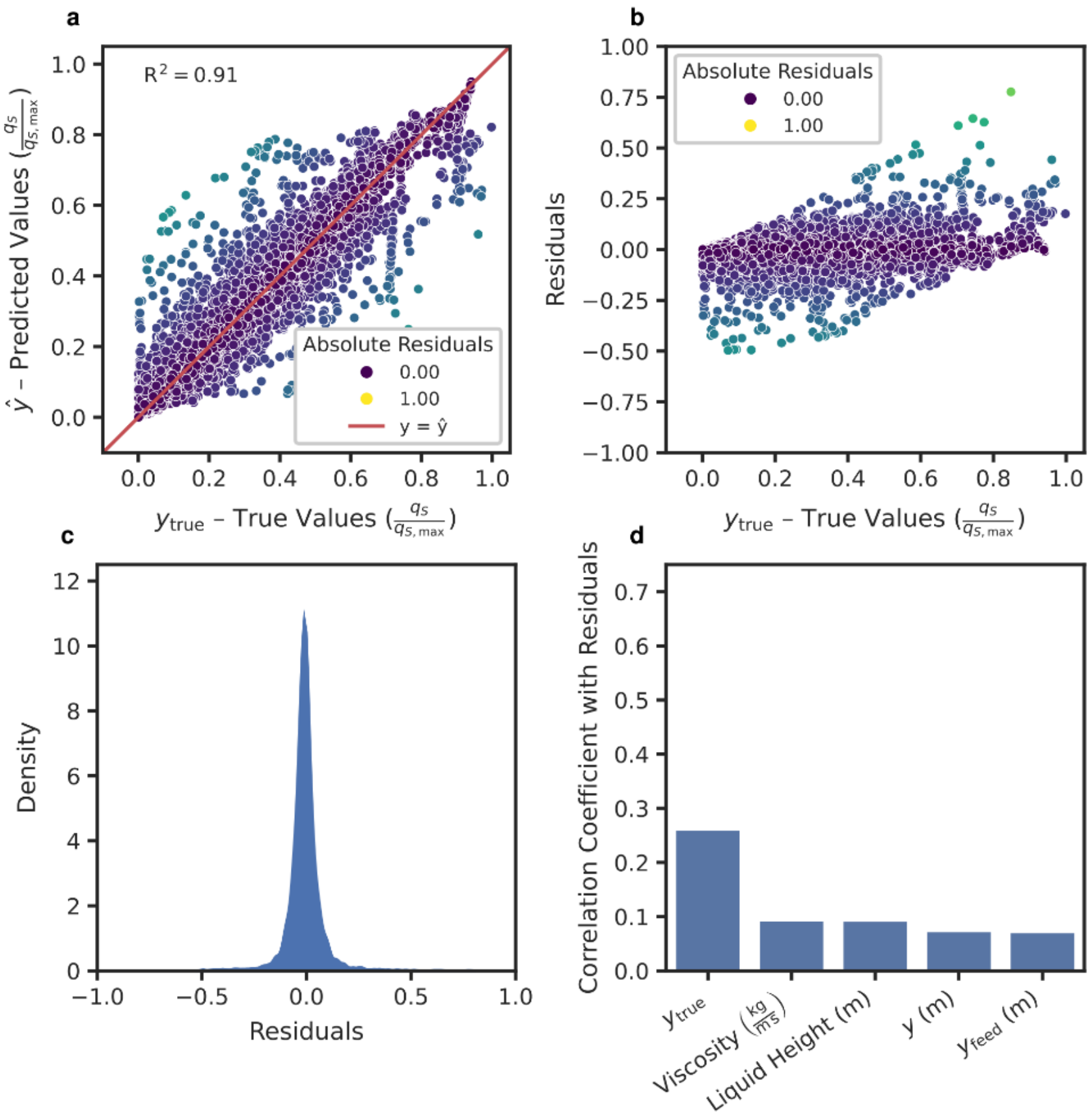

Supplementary Fig. 12. Residual diagnostics for the Modified-Attention NN on the uptake-based target, ${}^{q_S}/_{q_{S,\mathrm{max}}}$. Residual diagnostics on the independent test set. **a**, Predicted values ($\hat{y}$) versus CFD reference values (y_{true}); the red dashed line denotes the $\mathbf{1:1}$ reference and the annotated value reports

$\mathbf{R^2}$. **b**, Residuals, $\boldsymbol{\varepsilon = y_{true} - \hat{y}}$, as a function of $\boldsymbol{y_{true}}$, with points colored by residual magnitude. **c**, Residual-density distribution. **d**, Correlations between residuals and individual model predictors.

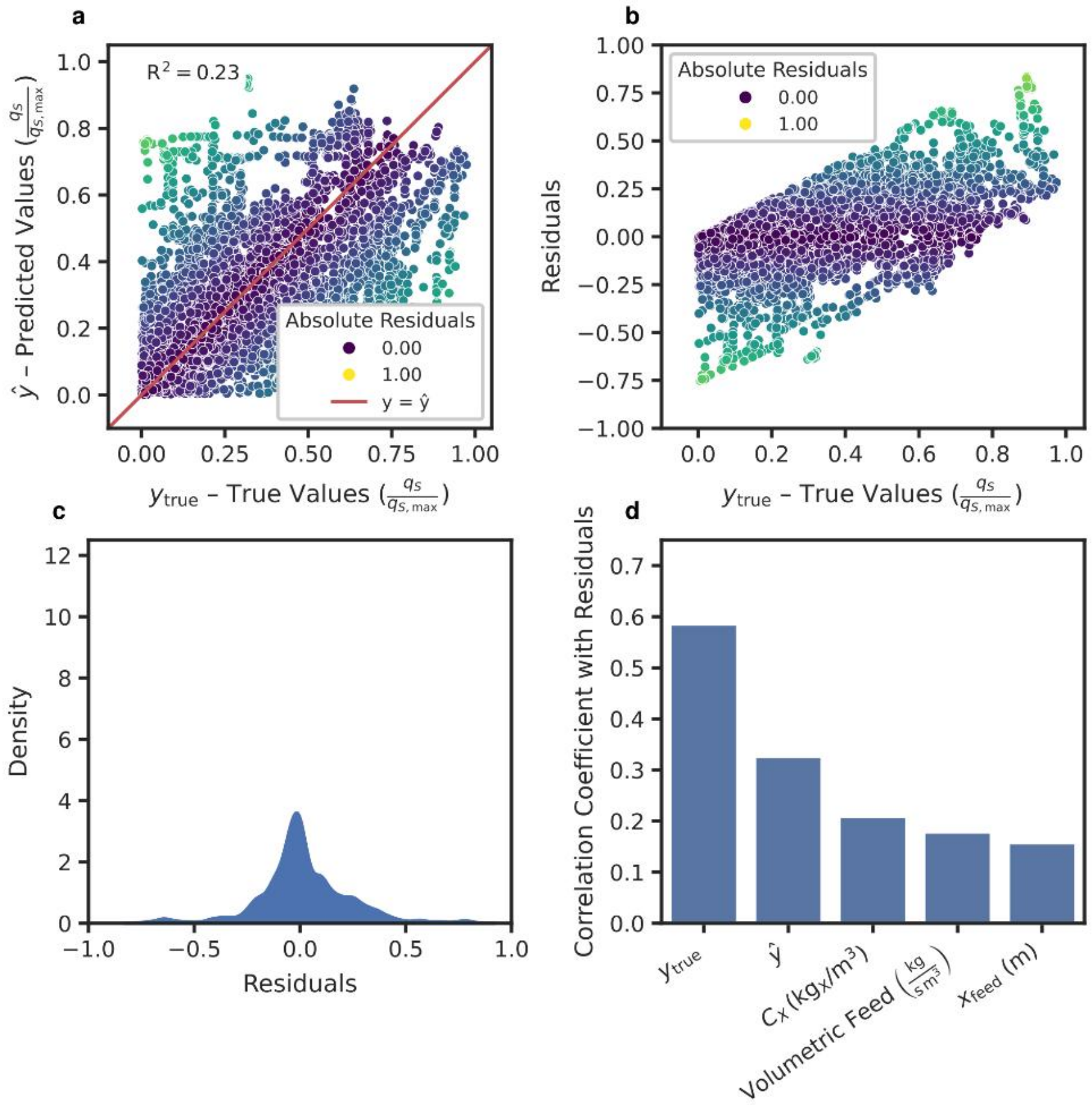

Supplementary Fig. 13. Residual diagnostics for the k-nearest-neighbors baseline on the uptake-based target, $^{q_S}/_{q_{S,\max}}$. Residual diagnostics on the independent test set. **a**, Predicted values $\hat{\boldsymbol{y}}$ versus CFD reference values $\boldsymbol{y_{true}}$; the red dashed line denotes the $\mathbf{1:1}$ reference and the annotated value reports $\mathbf{R^2}$. **b**, Residuals $\boldsymbol{\varepsilon = y_{true} - \hat{y}}$ as a function of $\boldsymbol{y_{true}}$, with points coloured by residual magnitude. **c**, Residual-density distribution. **d**, Correlations between residuals and individual model predictors.

More generally, models ranking best by low MSE and high R^2 exhibited tighter clouds around the $1{:}1$ line, near-zero residual means, weaker dependence of ε on y_{true}, and smaller residual–predictor correlations. Conversely, weaker learners showed stronger regression toward the mean, fanning residual variance and greater dependence of the errors on individual predictors (Supplementary Figs. 14–30). This agreement between aggregate performance and residual structure increases confidence that the benchmark ranking reflects genuine differences in generalization rather than idiosyncrasies of a single metric.

A notable case is the fourth-order Polynomial–Lasso regressor (Supplementary Fig. 22), which—despite being comparatively simple and linear in its fitted parameters—shows a particularly clean residual signature: residuals are symmetrically distributed with near-constant spread and negligible correlation with individual predictors. This indicates that a moderate polynomial basis, combined with L_1 shrinkage, captures a large fraction of the systematic variation in $^{q_S}/_{q_{S,\max}}$ while suppressing spurious interactions. Nevertheless, its global error remains slightly higher than that of the best NN models (Fig. 2), consistent with the fact that even a rich polynomial expansion cannot fully reproduce the more complex nonlinearities and spatial interactions captured by attention-based architectures. Practically, Poly4-Lasso therefore serves as a strong,

interpretable baseline: it explains much of the structured error while leaving a smaller residual component that benefits from higher-capacity learners.

Across architectures, the residual–response plots often exhibit a similar regression-to-the-mean structure—mild overprediction at low ${q_S}/{q_{S,\max}}$ and underprediction at high values—with the magnitude decreasing for more expressive learners. The broadly consistent direction of this bias suggests a data–physics interaction rather than a model-specific artifact. Steep, localized gradients near the feed and highly depleted regions occupy a small fraction of the reactor volume and are intrinsically harder to learn than the mid-range bulk. Three mechanisms can reinforce this behavior: (i) class imbalance, because extreme plume and starved regions are rare and MSE-weighted objectives are therefore dominated by bulk samples; (ii) bounded target behavior, because ${q_S}/{q_{S,\max}} \in [0,1]$ compresses variability near the bounds and reduces the effective signal for fitting extremes; and (iii) sampling geometry, because uniform spatial sampling under-represents small-volume regions in which gradients are steepest. Targeted oversampling near these extremes and/or loss reweighting that emphasizes the bounds could increase the learning signal for these physically important but underrepresented states and partially flatten the residual tilt.

Heavy-tailed residual densities reflect occasional large errors and are expected for highly non-uniform scalar fields; they do not by themselves invalidate predictive usefulness. In the strongest models, such as the Modified-Attention NN, bias is mild and largely confined to the target extremes, residual variance is approximately stable, and residual–predictor correlations remain small, indicating that most systematic structure is captured. Conversely, rigid or distance-based baselines such as DT and k-NN exhibit more pronounced bias, stronger heteroscedasticity and larger residual–predictor correlations, indicating that important nonlinear structure remains unmodelled. This behavior is consistent with the difficulty of defining informative neighborhoods in a high-dimensional and strongly nonlinear predictor space.

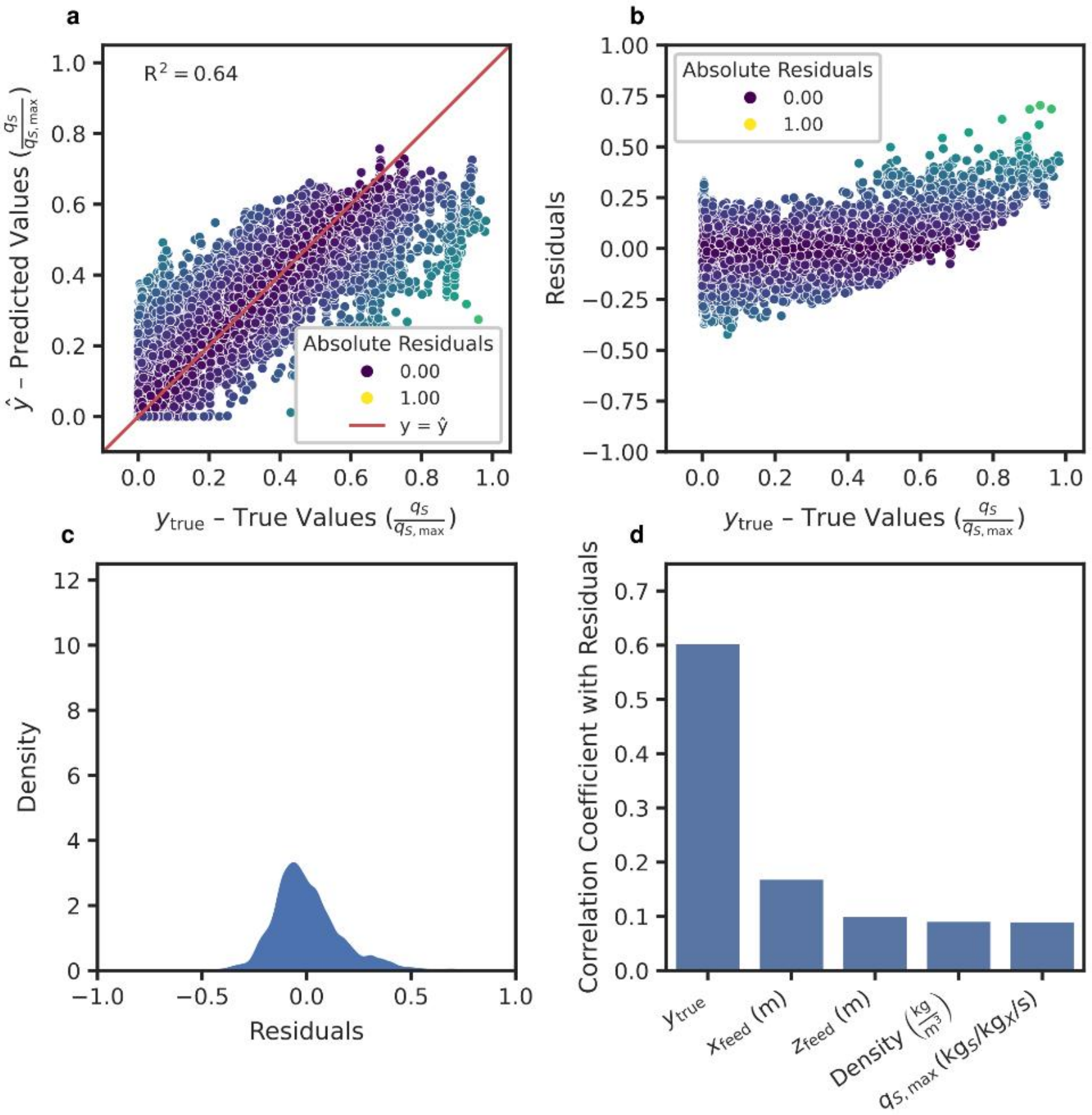

Supplementary Fig. 14. Residual diagnostics for multiple linear regression (MLR) on the uptake-based target $^{q_S}/_{q_{S,\max}}$. Residual diagnostics on the independent test set. **a**, Predicted values $\hat{y}$ versus CFD reference values y_{true}; the red dashed line denotes the $\mathbf{1}:\mathbf{1}$ reference and the annotated value reports $\mathbf{R}^2$. **b**, Residuals $\varepsilon = y_{\text{true}} - \hat{y}$ as a function of y_{true}, with points coloured by residual magnitude. **c**, Residual-density distribution. **d**, Correlations between residuals and individual model predictors.

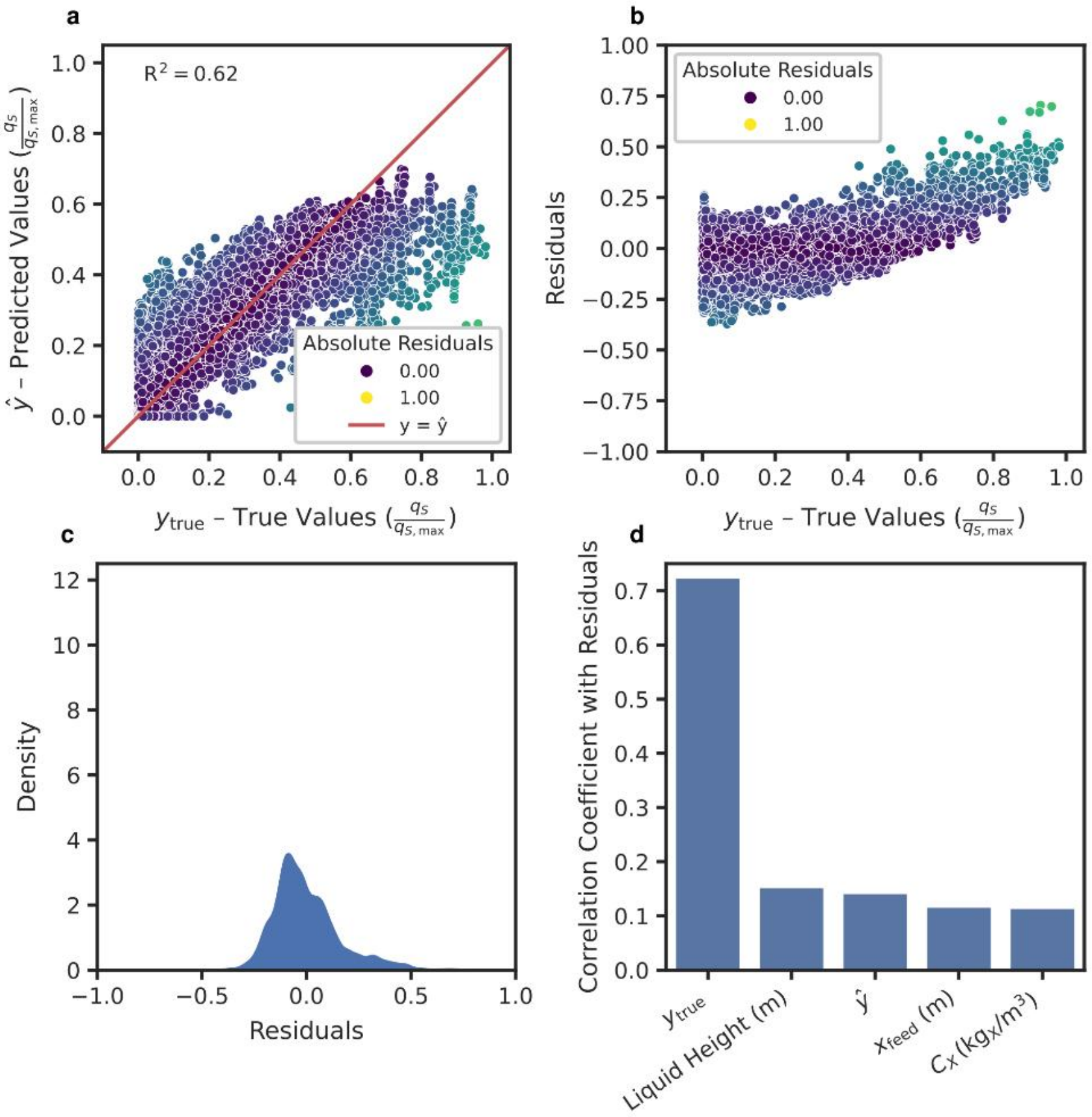

Supplementary Fig. 15. Residual diagnostics for Lasso regression on the uptake-based target $q_S/q_{S,\max}$. Residual diagnostics on the independent test set. **a**, Predicted values $\hat{y}$ versus CFD reference values y_{true}; the red dashed line denotes the $1{:}1$ reference and the annotated value reports R^2. **b**, Residuals $\varepsilon = y_{\text{true}} - \hat{y}$ as a function of y_{true}, with points coloured by residual magnitude. **c**, Residual-density distribution. **d**, Correlations between residuals and individual model predictors.

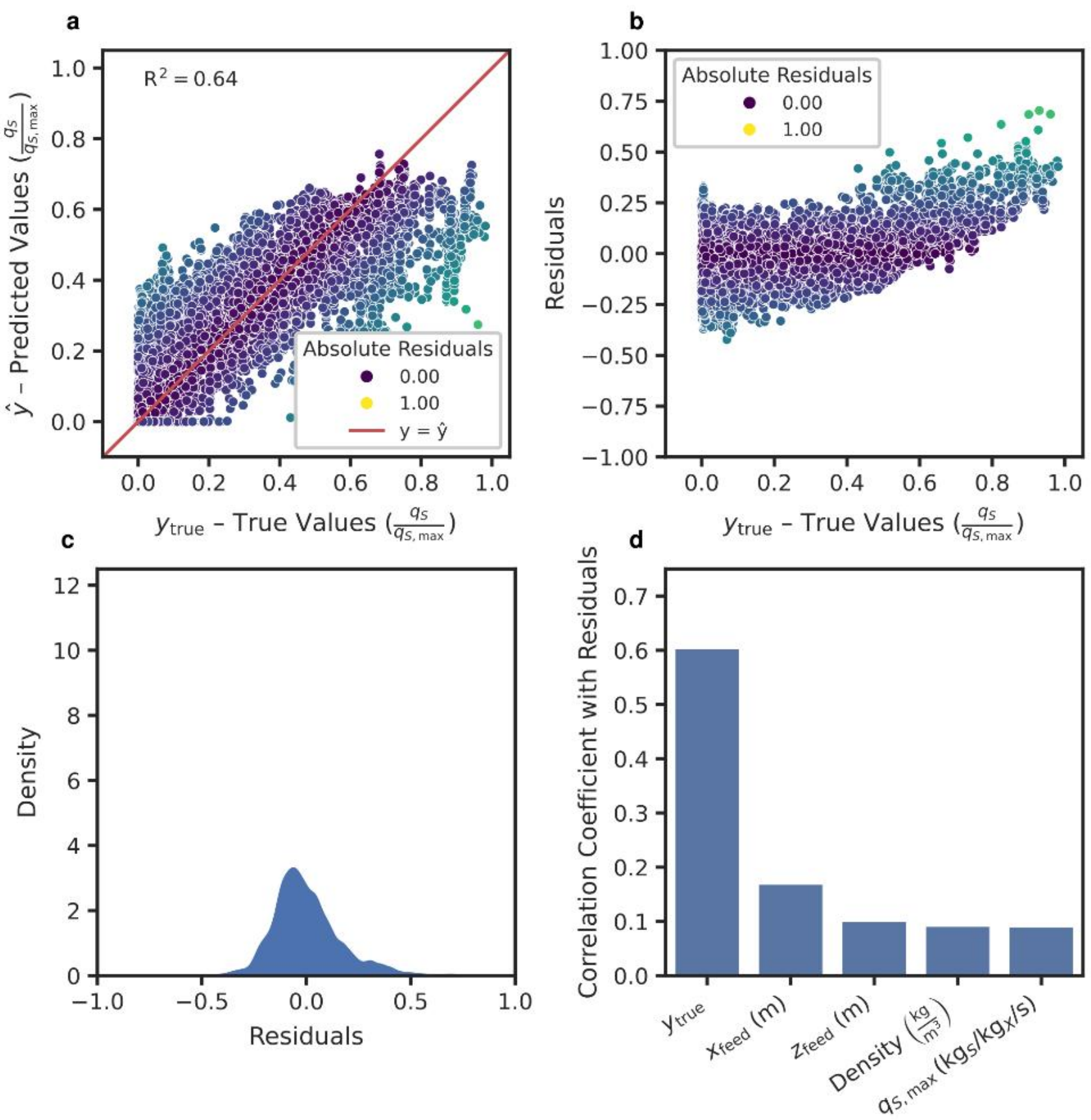

Supplementary Fig. 16. Residual diagnostics for Ridge regression on the uptake-based target $q_S/q_{S,\max}$. Residual diagnostics on the independent test set. **a**, Predicted values $\hat{y}$ versus CFD reference values y_{true}; the red dashed line denotes the $1{:}1$ reference and the annotated value reports R^2. **b**, Residuals $\varepsilon = y_{\text{true}} - \hat{y}$ as a function of y_{true}, with points coloured by residual magnitude. **c**, Residual-density distribution. **d**, Correlations between residuals and individual model predictors.

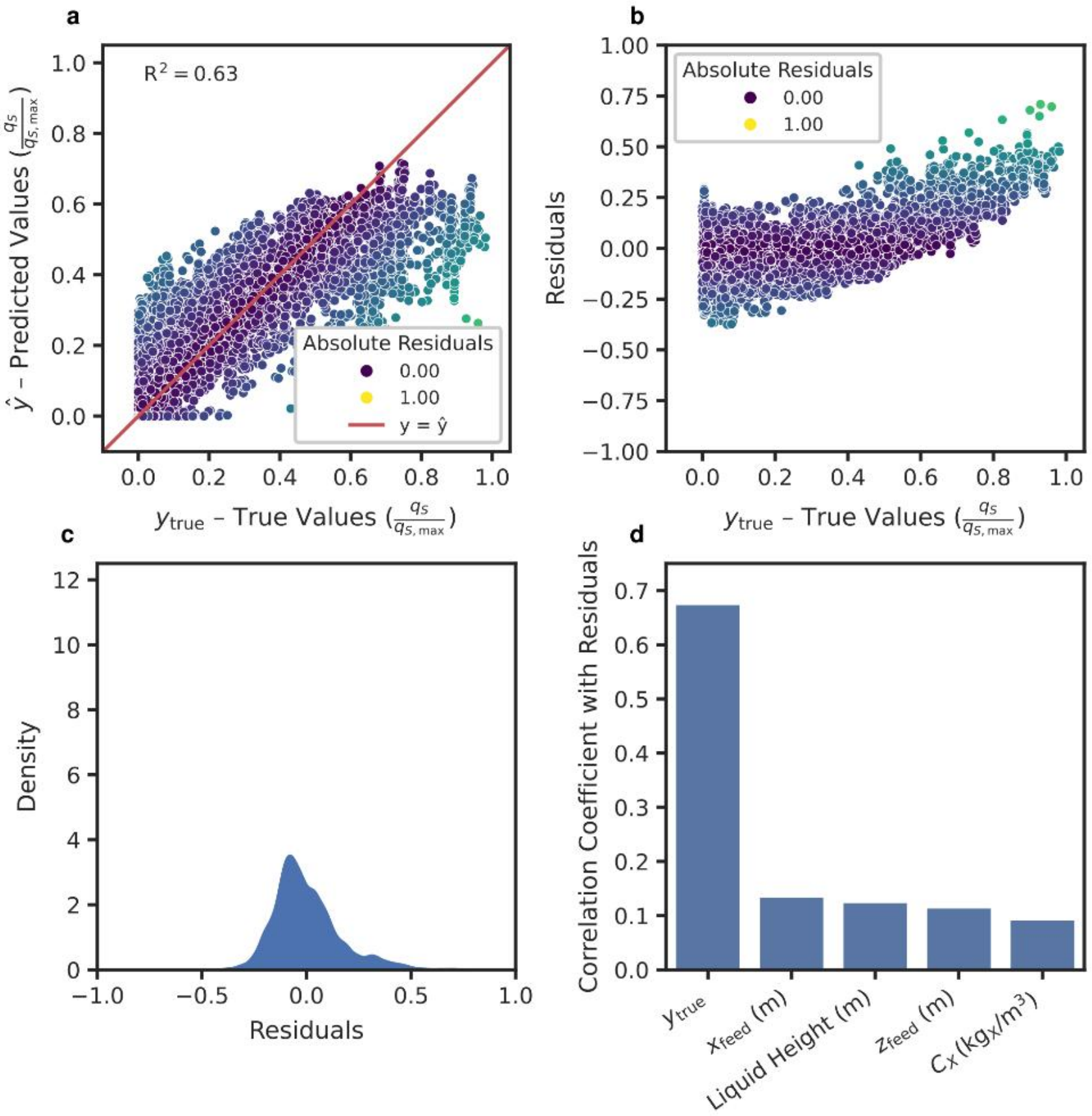

Supplementary Fig. 17. Residual diagnostics for Elastic Net regression on the uptake-based target $q_S/q_{S,\max}$. Residual diagnostics on the independent test set. **a**, Predicted values $\hat{y}$ versus CFD reference values y_{true}; the red dashed line denotes the $1{:}1$ reference and the annotated value reports R^2. **b**, Residuals $\varepsilon = y_{true} - \hat{y}$ as a function of y_{true}, with points coloured by residual magnitude. **c**, Residual-density distribution. **d**, Correlations between residuals and individual model predictors.

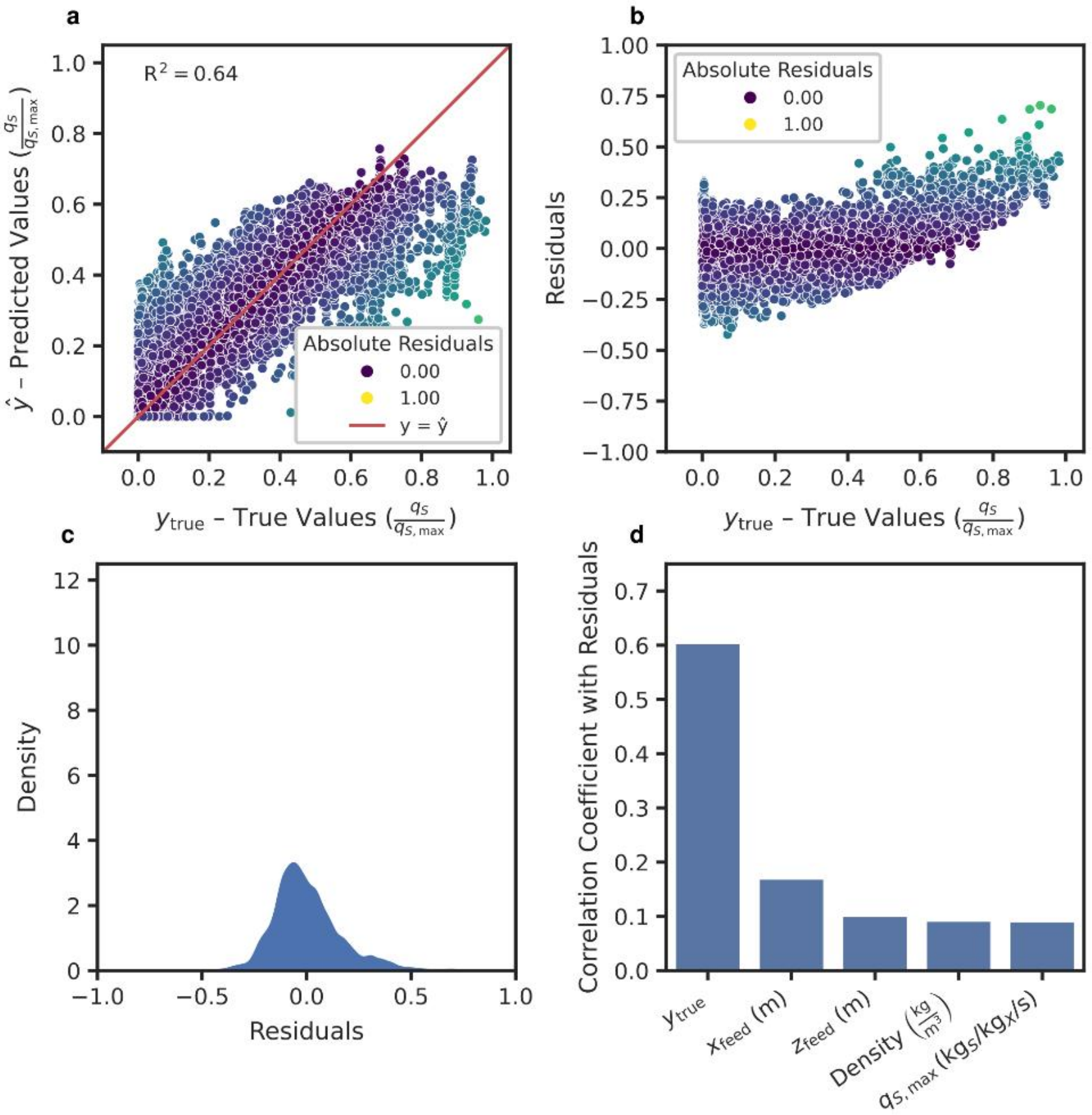

Supplementary Fig. 18. Residual diagnostics for Huber regression (best $\mathbf{MAE}$) on the uptake-based target $q_S/q_{S,\max}$. Residual diagnostics on the independent test set. **a**, Predicted values $\hat{y}$ versus CFD reference values y_{true}; the red dashed line denotes the $1:1$ reference and the annotated value reports $\mathbf{R^2}$. **b**, Residuals $\varepsilon = y_{\mathrm{true}} - \hat{y}$ as a function of y_{true}, with points coloured by residual magnitude. **c**, Residual-density distribution. **d**, Correlations between residuals and individual model predictors.

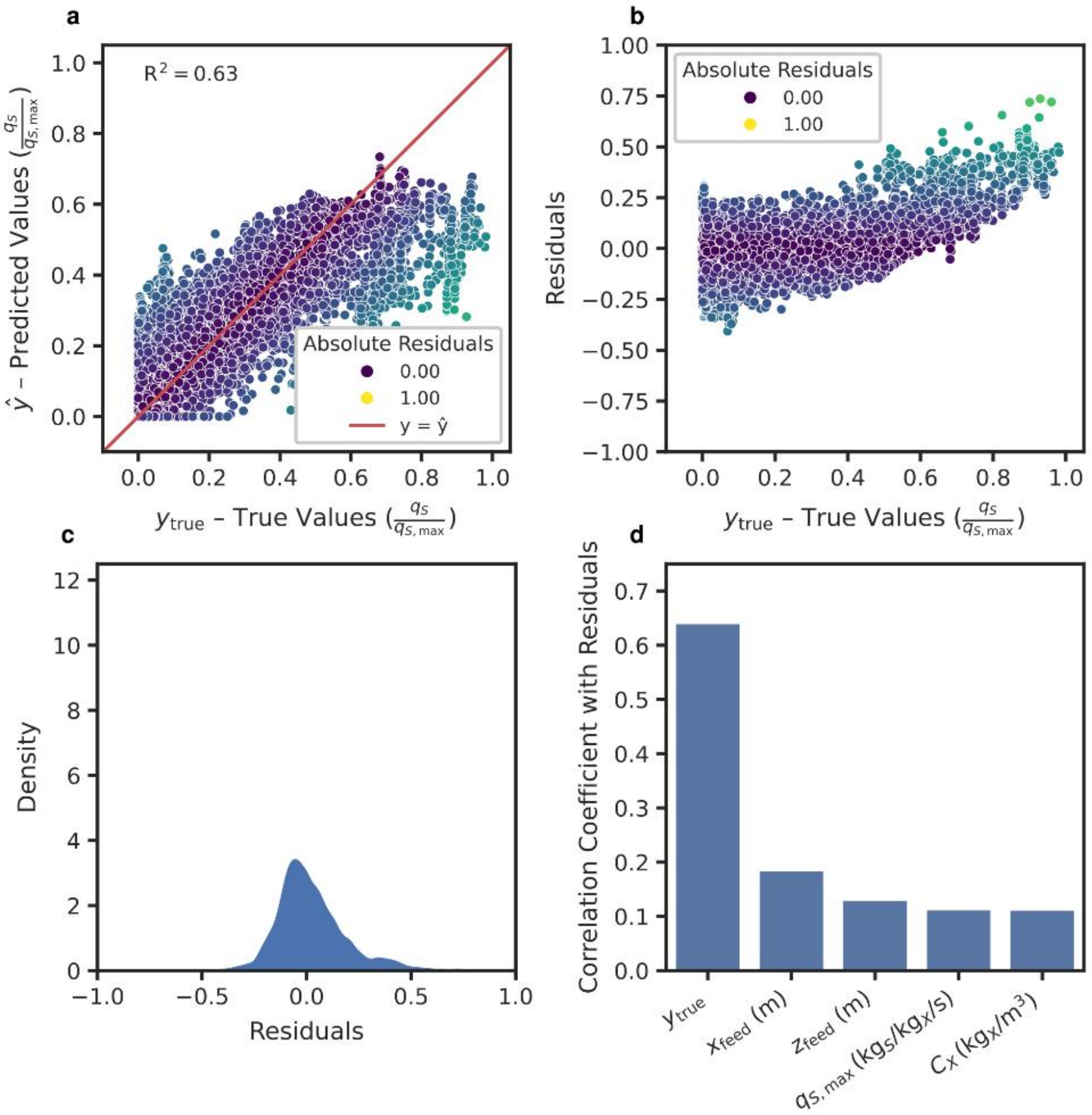

Supplementary Fig. 19. Residual diagnostics for Huber regression (best MSE) on the uptake-based target $^{q_S}/_{q_{S,\max}}$. Residual diagnostics on the independent test set. **a**, Predicted values $\hat{y}$ versus CFD reference values y_{true}; the red dashed line denotes the $1:1$ reference and the annotated value reports R^2. **b**, Residuals $\varepsilon = y_{\mathrm{true}} - \hat{y}$ as a function of y_{true}, with points coloured by residual magnitude. **c**, Residual-density distribution. **d**, Correlations between residuals and individual model predictors.

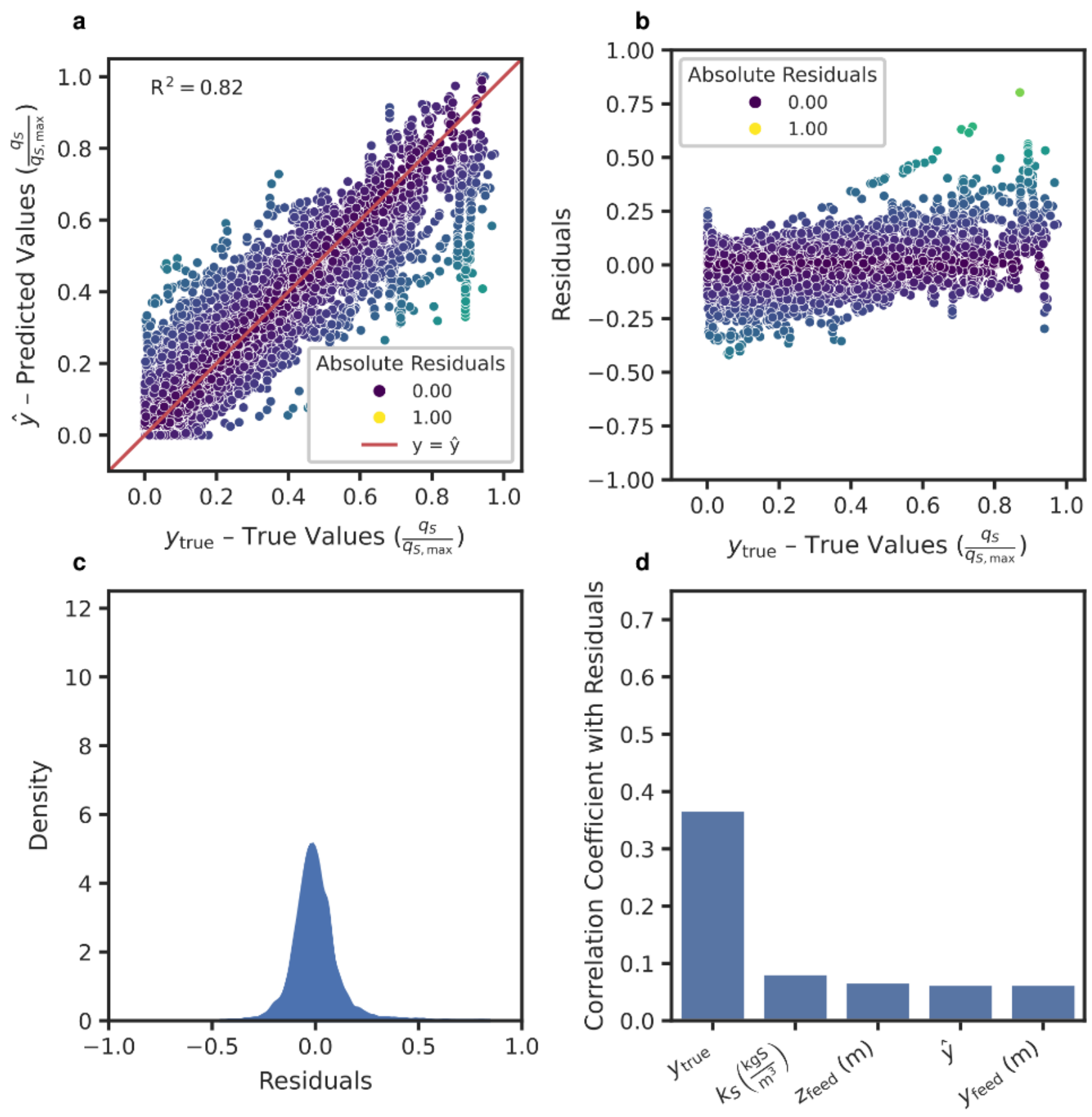

Supplementary Fig. 20. Residual diagnostics for Poly2-Lasso AIC on the uptake-based target $q_S/q_{S,\max}$. Residual diagnostics on the independent test set. **a**, Predicted values $\hat{y}$ versus CFD reference values y_{true}; the red dashed line denotes the $1:1$ reference and the annotated value reports R^2. **b**, Residuals $\varepsilon = y_{\text{true}} - \hat{y}$ as a function of y_{true}, with points coloured by residual magnitude. **c**, Residual-density distribution. **d**, Correlations between residuals and individual model predictors.

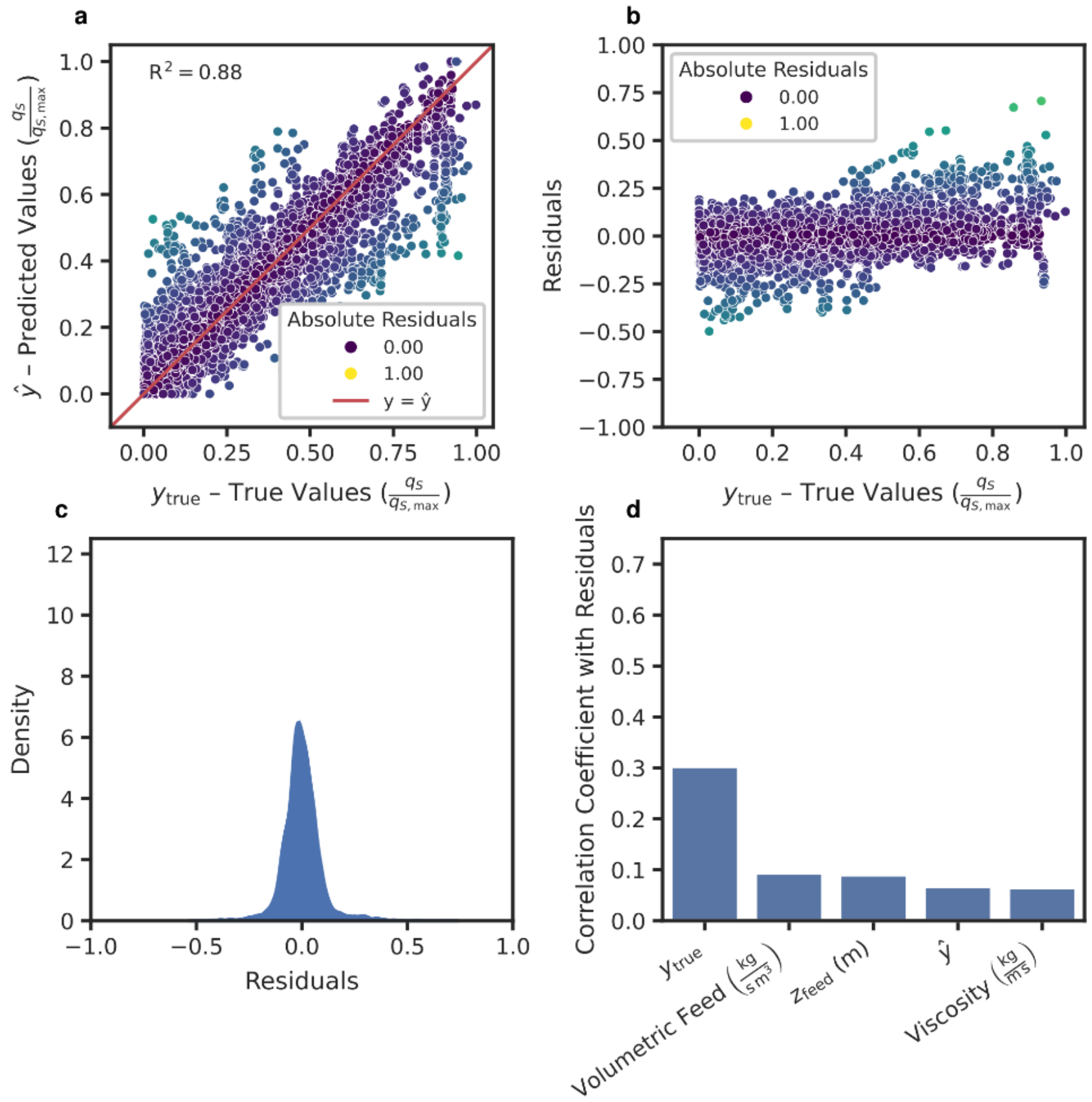

Supplementary Fig. 21. Residual diagnostics for Poly3-Lasso AIC on the uptake-based target $q_S/q_{S,\max}$. Residual diagnostics on the independent test set. **a**, Predicted values $\hat{y}$ versus CFD reference values y_{true}; the red dashed line denotes the $1{:}1$ reference and the annotated value reports R^2. **b**, Residuals $\varepsilon = y_{\text{true}} - \hat{y}$ as a function of y_{true}, with points coloured by residual magnitude. **c**, Residual-density distribution. **d**, Correlations between residuals and individual model predictors.

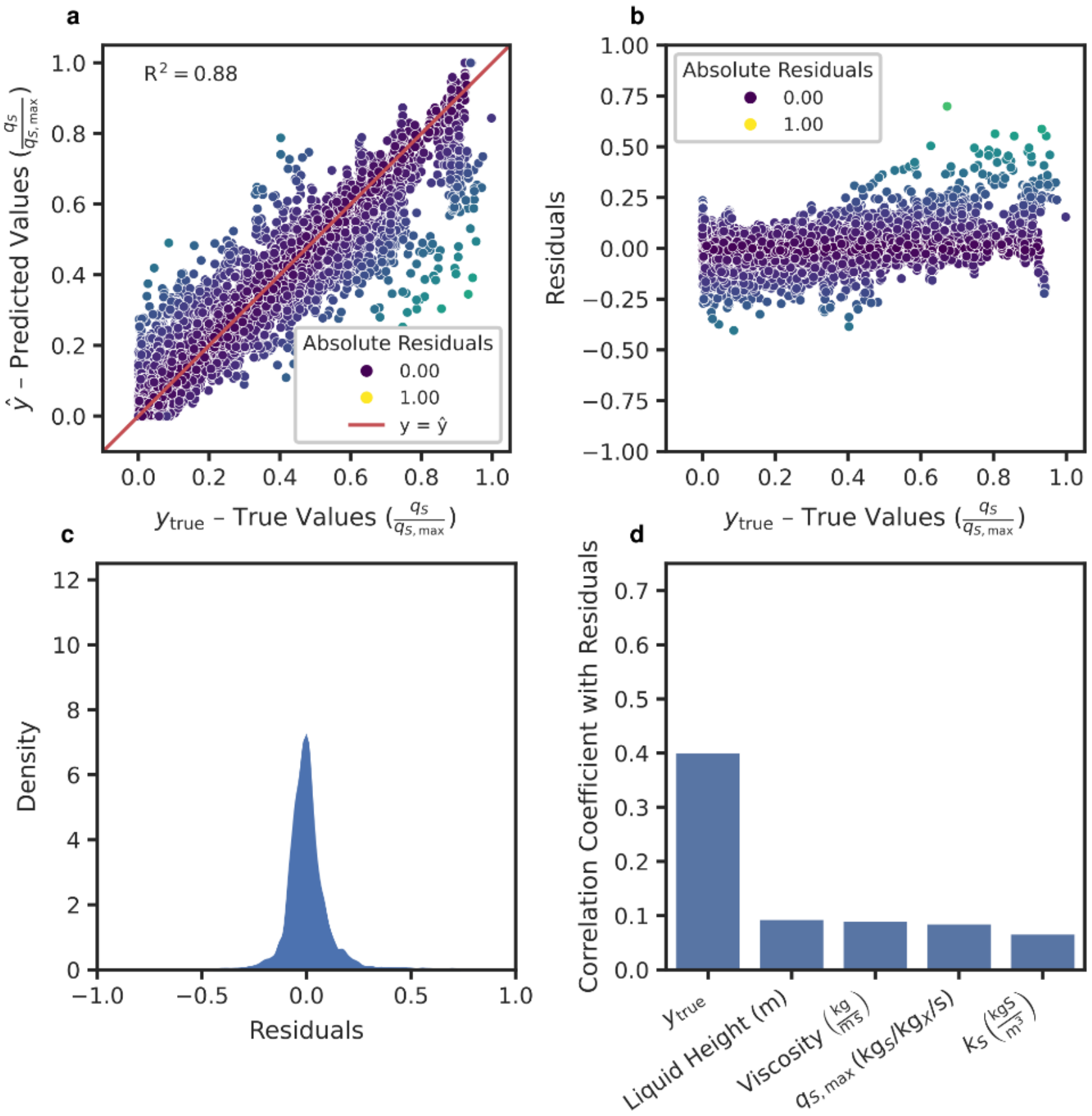

Supplementary Fig. 22. Residual diagnostics for Poly4-Lasso AIC on the uptake-based target $q_S/q_{S,\max}$. Residual diagnostics on the independent test set. **a**, Predicted values $\hat{y}$ versus CFD reference values y_{true}; the red dashed line denotes the $1{:}1$ reference and the annotated value reports R^2. **b**, Residuals $\varepsilon = y_{\text{true}} - \hat{y}$ as a function of y_{true}, with points coloured by residual magnitude. **c**, Residual-density distribution. **d**, Correlations between residuals and individual model predictors.

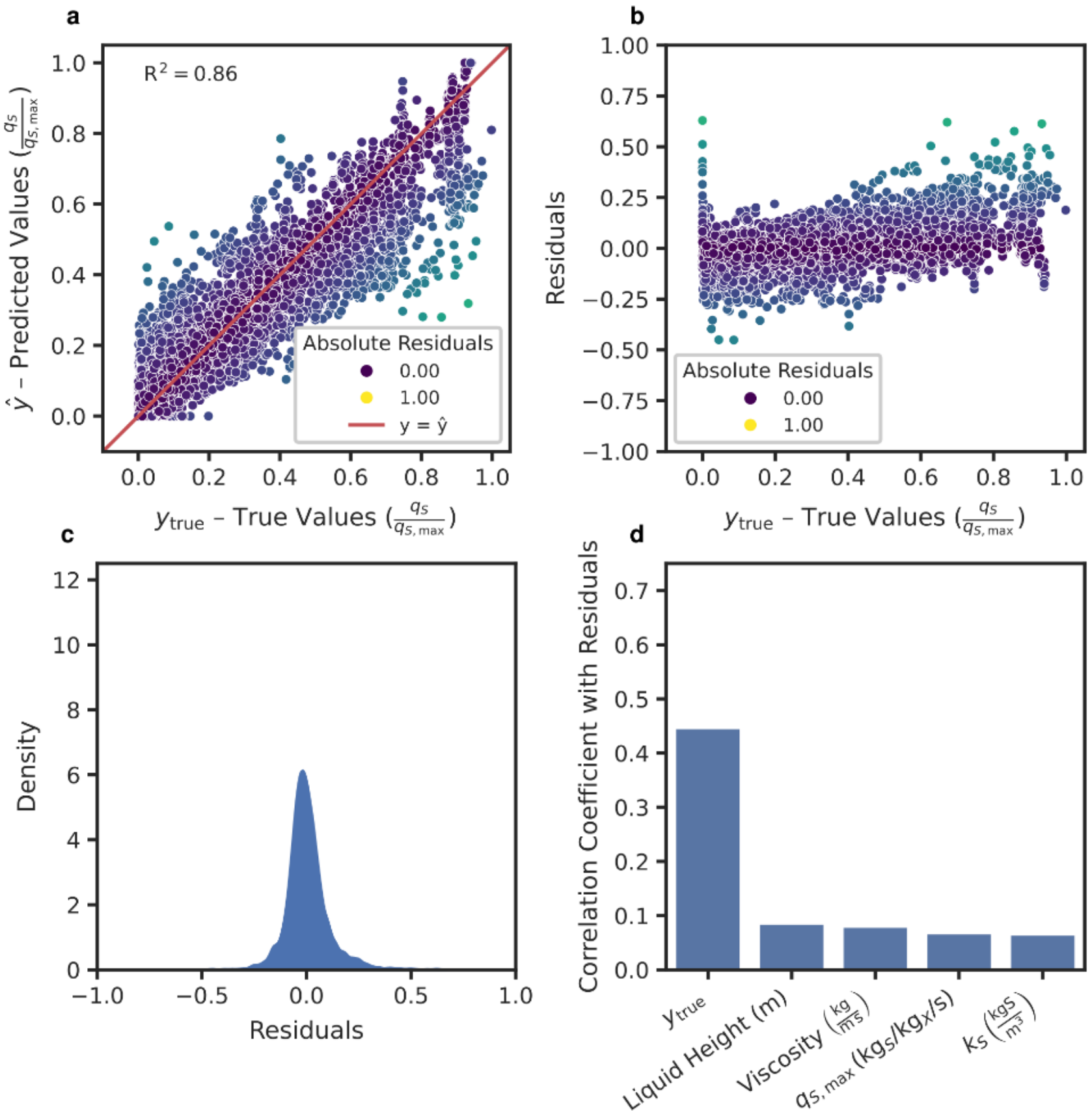

Supplementary Fig. 23. Residual diagnostics for Poly5-Lasso AIC on the uptake-based target $q_S/q_{S,\max}$. Residual diagnostics on the independent test set. **a**, Predicted values $\hat{y}$ versus CFD reference values y_{true}; the red dashed line denotes the $1:1$ reference and the annotated value reports R^2. **b**, Residuals $\varepsilon = y_{\text{true}} - \hat{y}$ as a function of y_{true}, with points coloured by residual magnitude. **c**, Residual-density distribution. **d**, Correlations between residuals and individual model predictors.

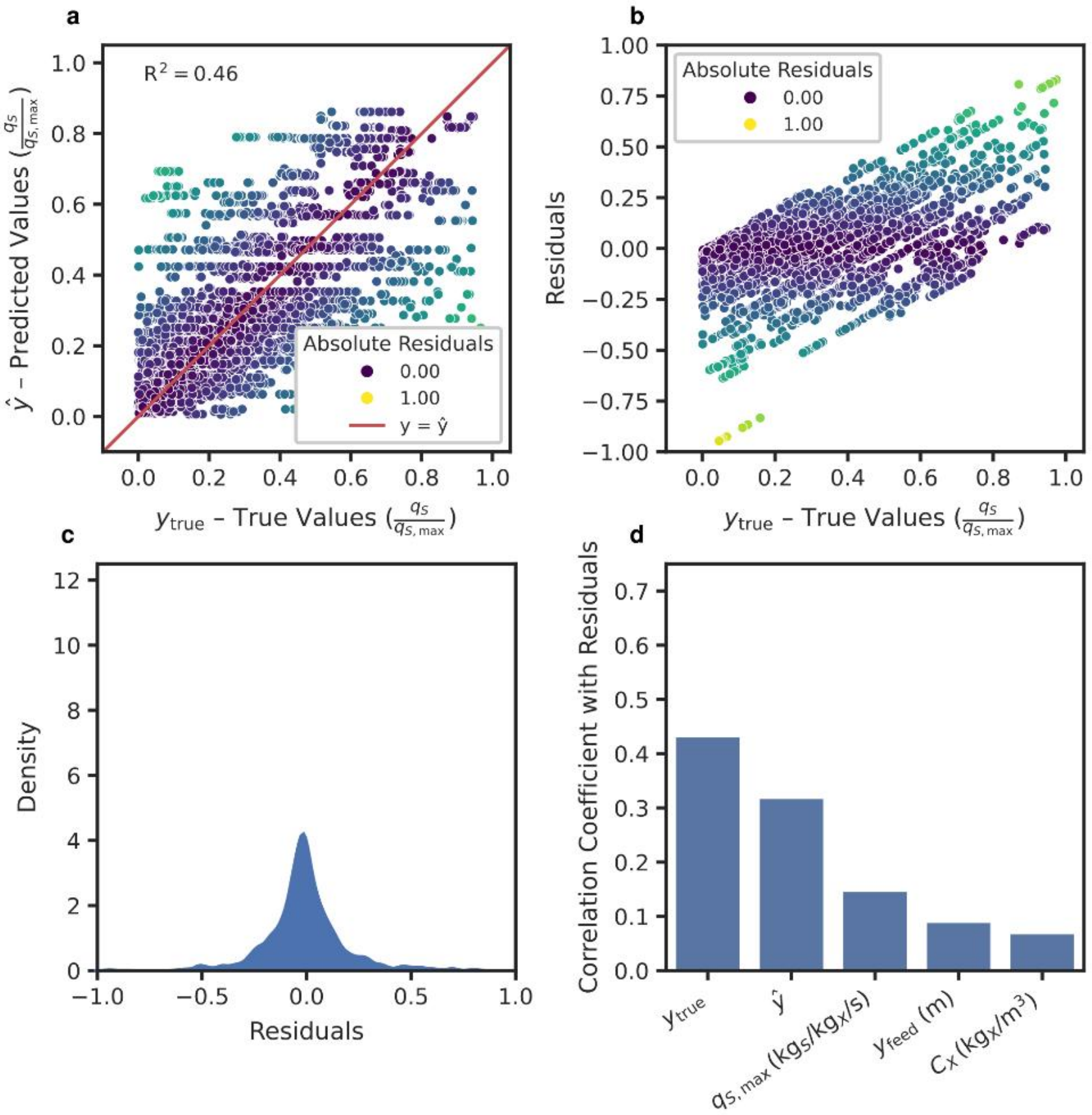

Supplementary Fig. 24. Residual diagnostics for decision trees (DT) on the uptake-based target $q_S/q_{S,\mathbf{max}}$. Residual diagnostics on the independent test set. **a**, Predicted values $\hat{y}$ versus CFD reference values $y_{\mathbf{true}}$; the red dashed line denotes the $\mathbf{1}:\mathbf{1}$ reference and the annotated value reports $\mathbf{R^2}$. **b**, Residuals $\varepsilon = y_{\mathbf{true}} - \hat{y}$ as a function of $y_{\mathbf{true}}$, with points coloured by residual magnitude. **c**, Residual-density distribution. **d**, Correlations between residuals and individual model predictors.

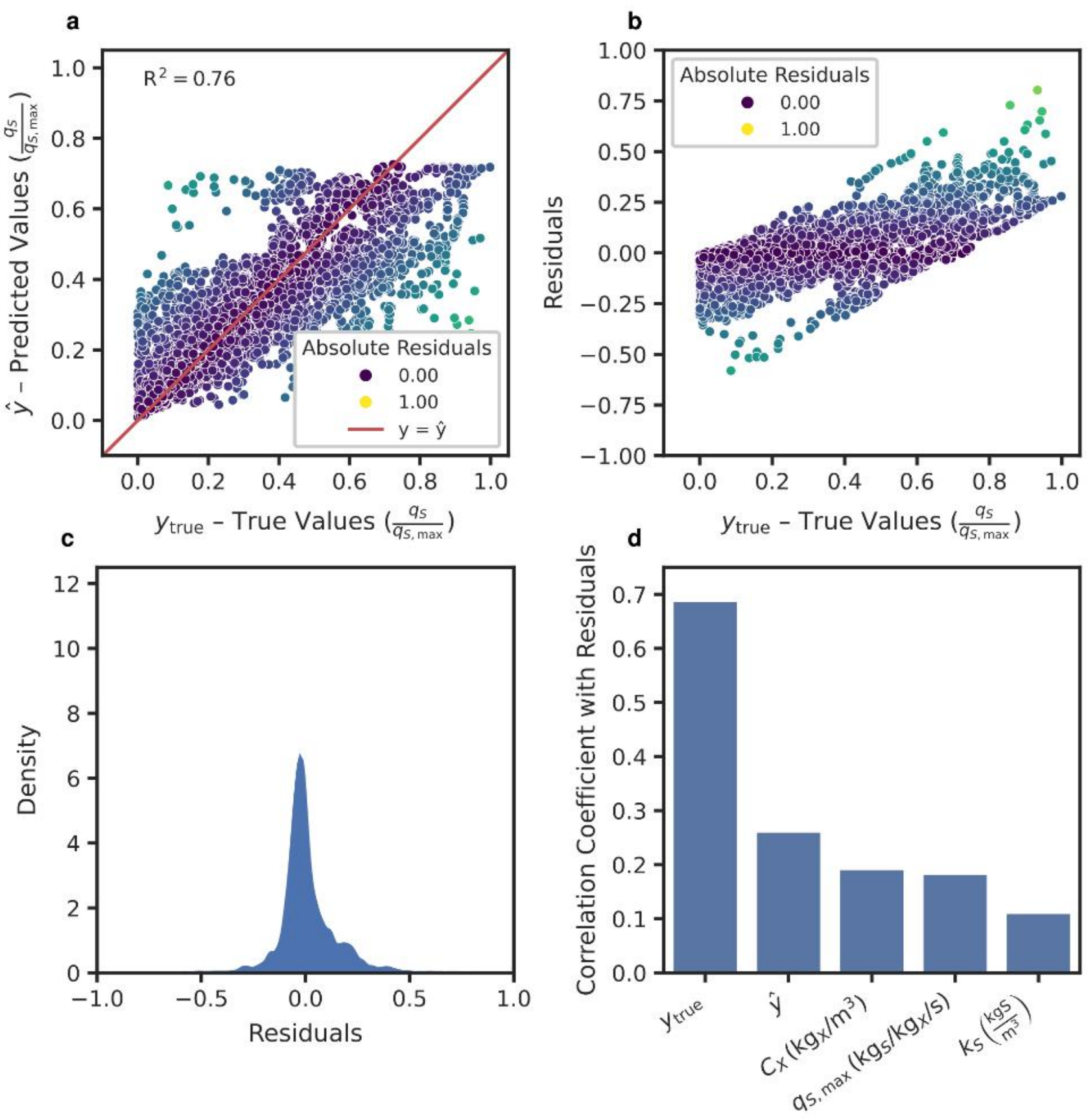

Supplementary Fig. 25. Residual diagnostics for random forest regression (RFR) on the uptake-based target $q_S/q_{S,\mathrm{max}}$. Residual diagnostics on the independent test set. **a**, Predicted values $\hat{y}$ versus CFD reference values y_{true}; the red dashed line denotes the $\mathbf{1}:\mathbf{1}$ reference and the annotated value reports $\mathbf{R^2}$. **b**, Residuals $\varepsilon = y_{\mathrm{true}} - \hat{y}$ as a function of y_{true}, with points coloured by residual magnitude. **c**, Residual-density distribution. **d**, Correlations between residuals and individual model predictors.

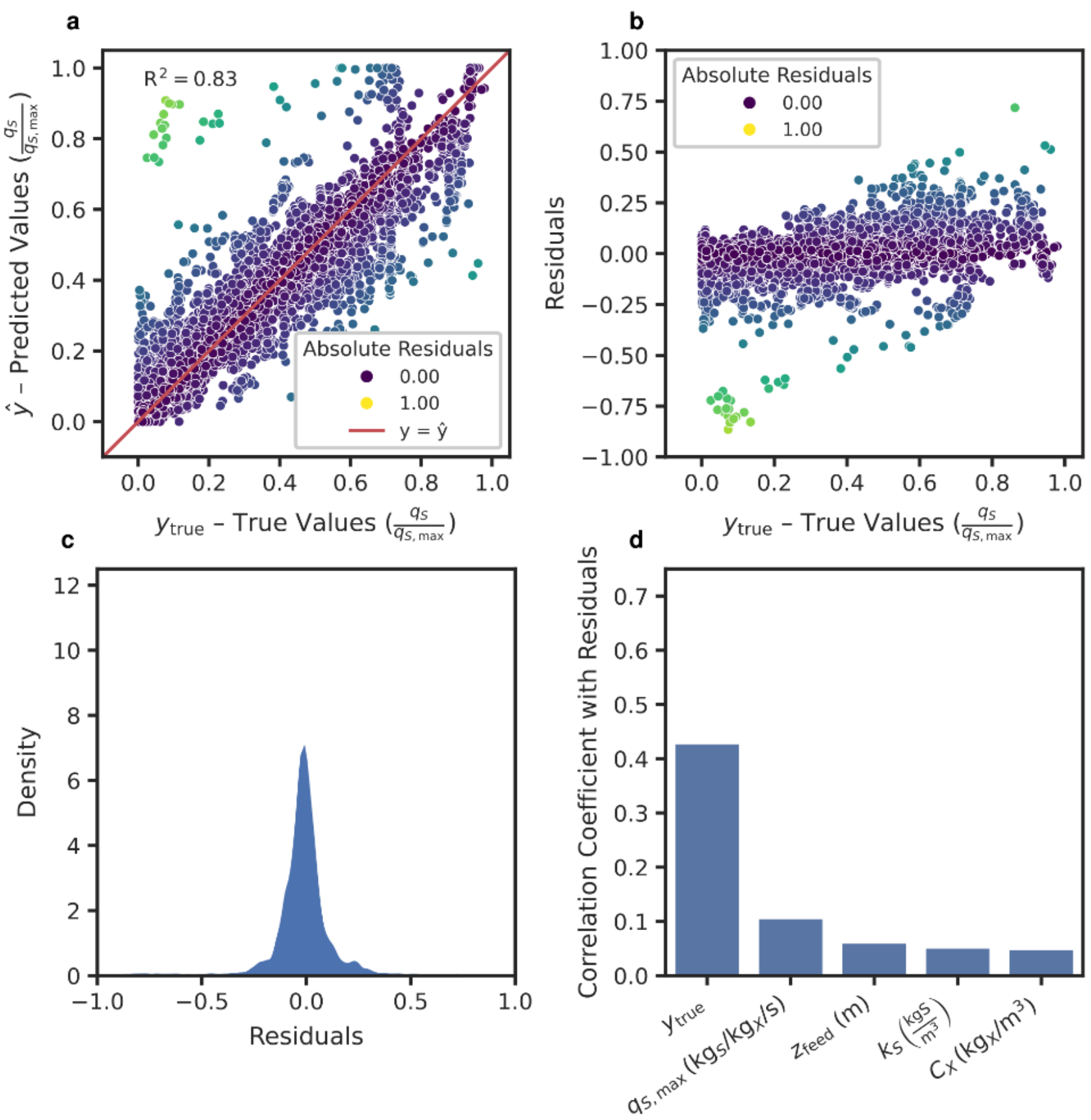

Supplementary Fig. 26. Residual diagnostics for gradient boosting regression (GBR) on the uptake-based target $q_S/q_{S,\max}$. Residual diagnostics on the independent test set. **a**, Predicted values $\hat{y}$ versus CFD reference values y_{true}; the red dashed line denotes the $1:1$ reference and the annotated value reports R^2. **b**, Residuals $\varepsilon = y_{\text{true}} - \hat{y}$ as a function of y_{true}, with points coloured by residual magnitude. **c**, Residual-density distribution. **d**, Correlations between residuals and individual model predictors.

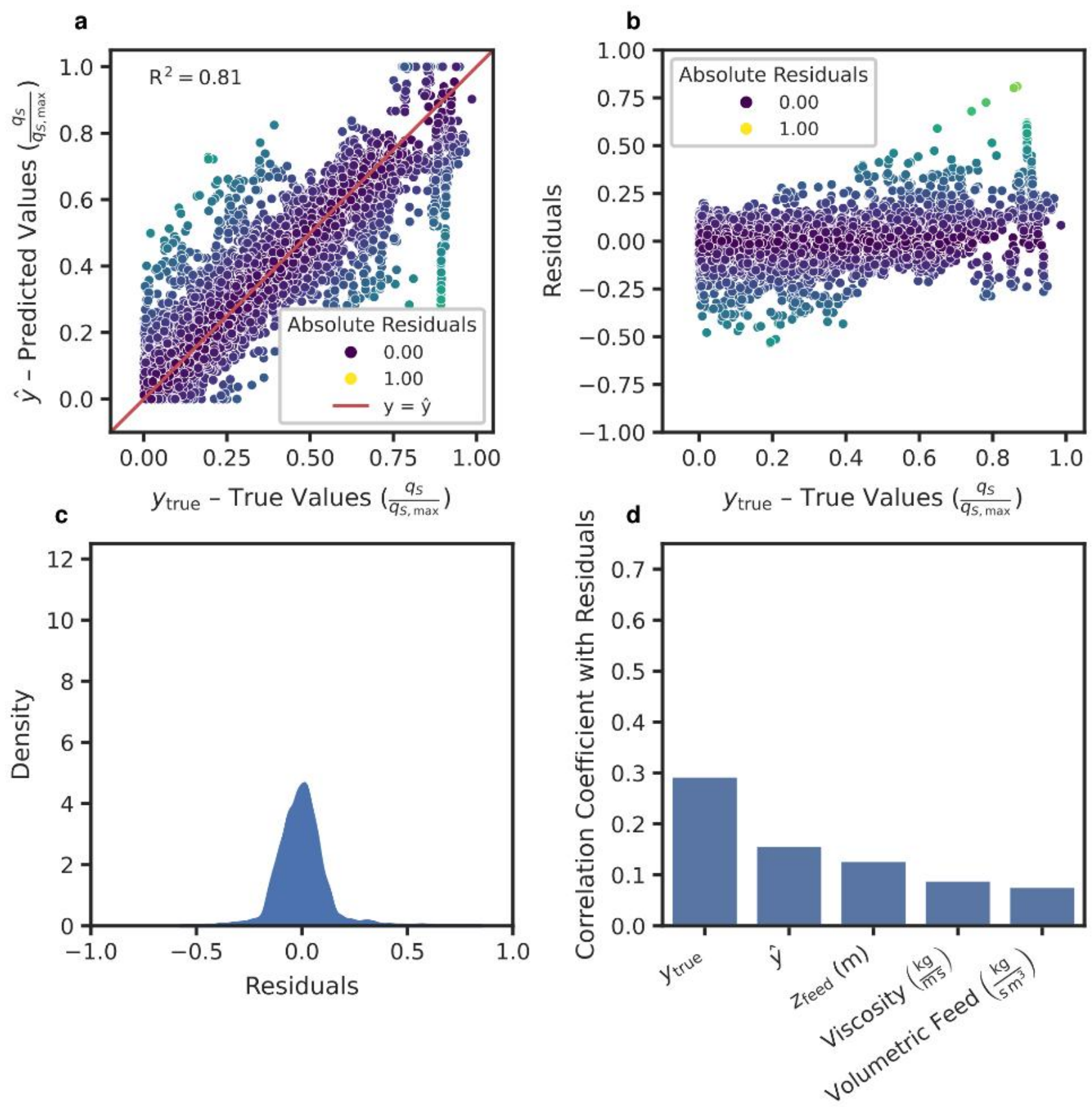

Supplementary Fig. 27. Residual diagnostics for support vector regression (SVR) on the uptake-based target $q_S/q_{S,\max}$. Residual diagnostics on the independent test set. **a**, Predicted values $\hat{y}$ versus CFD reference values y_{true}; the red dashed line denotes the $1:1$ reference and the annotated value reports R^2. **b**, Residuals $\varepsilon = y_{\text{true}} - \hat{y}$ as a function of y_{true}, with points coloured by residual magnitude. **c**, Residual-density distribution. **d**, Correlations between residuals and individual model predictors.

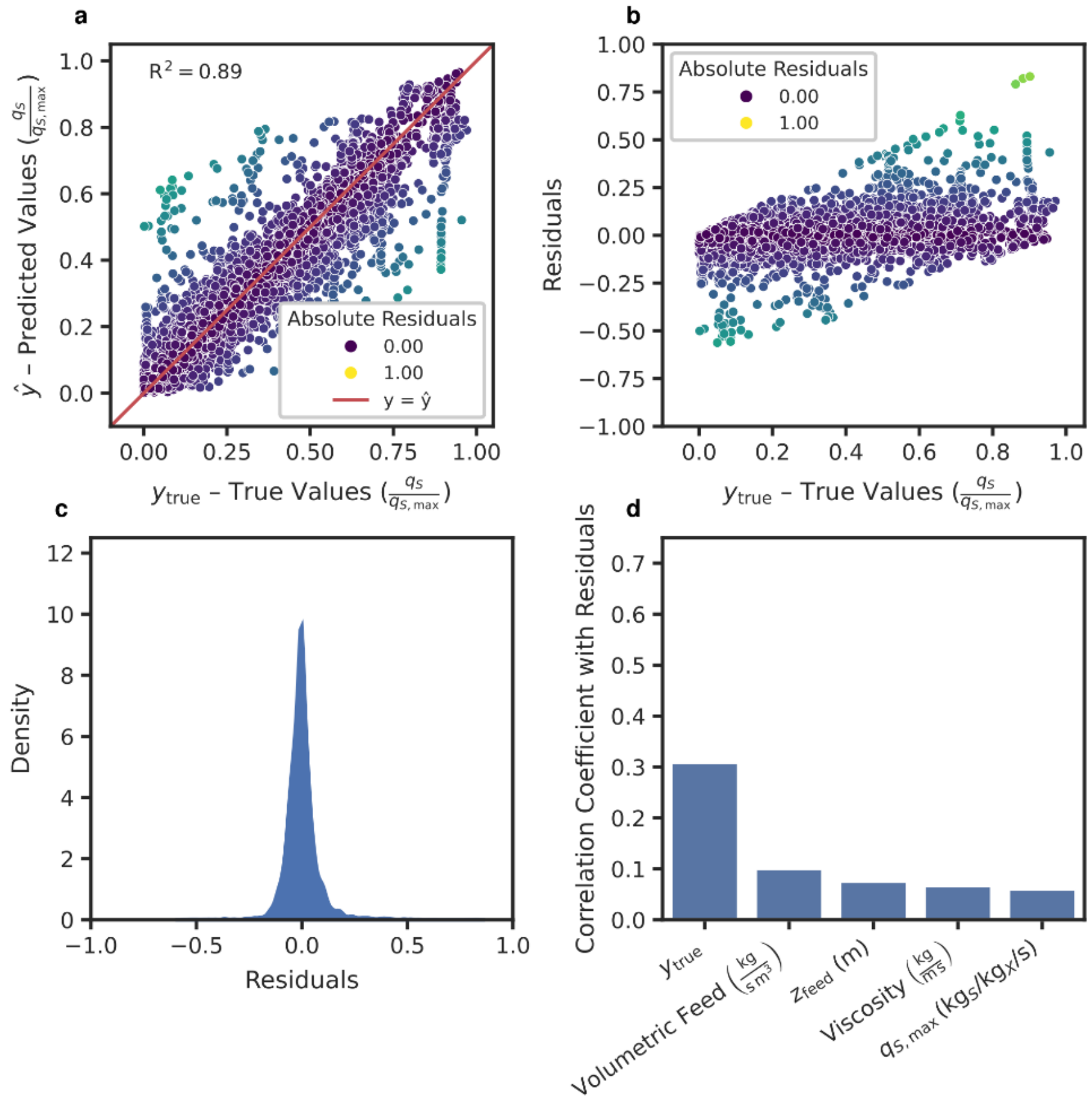

Supplementary Fig. 28. Residual diagnostics for Standard NN on the uptake-based target $q_S/q_{S,\max}$. Residual diagnostics on the independent test set. **a**, Predicted values $\hat{y}$ versus CFD reference values y_{true}; the red dashed line denotes the $1:1$ reference and the annotated value reports R^2. **b**, Residuals $\varepsilon = y_{\text{true}} - \hat{y}$ as a function of y_{true}, with points coloured by residual magnitude. **c**, Residual-density distribution. **d**, Correlations between residuals and individual model predictors.

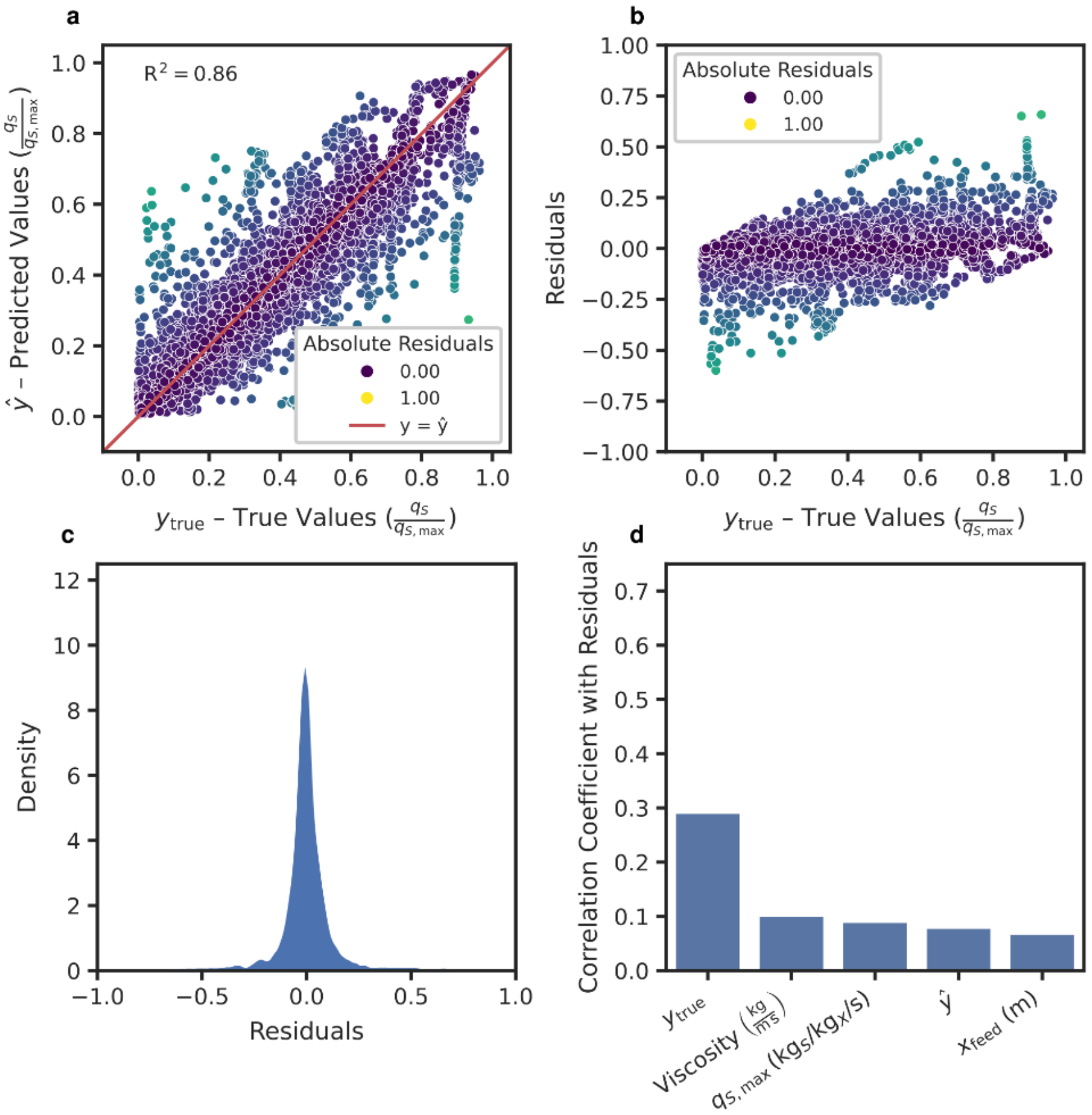

Supplementary Fig. 29. Residual diagnostics for Attention NN on the uptake-based target $^{q_S}/_{q_{S,max}}$. Residual diagnostics on the independent test set. **a**, Predicted values $\hat{\boldsymbol{y}}$ versus CFD reference values $\boldsymbol{y}_{\mathbf{true}}$; the red dashed line denotes the $\mathbf{1}:\mathbf{1}$ reference and the annotated value reports $\mathbf{R}^2$. **b**, Residuals $\boldsymbol{\varepsilon} = \boldsymbol{y}_{\mathbf{true}} - \hat{\boldsymbol{y}}$ as a function of $\boldsymbol{y}_{\mathbf{true}}$, with points coloured by residual magnitude. **c**, Residual-density distribution. **d**, Correlations between residuals and individual model predictors.

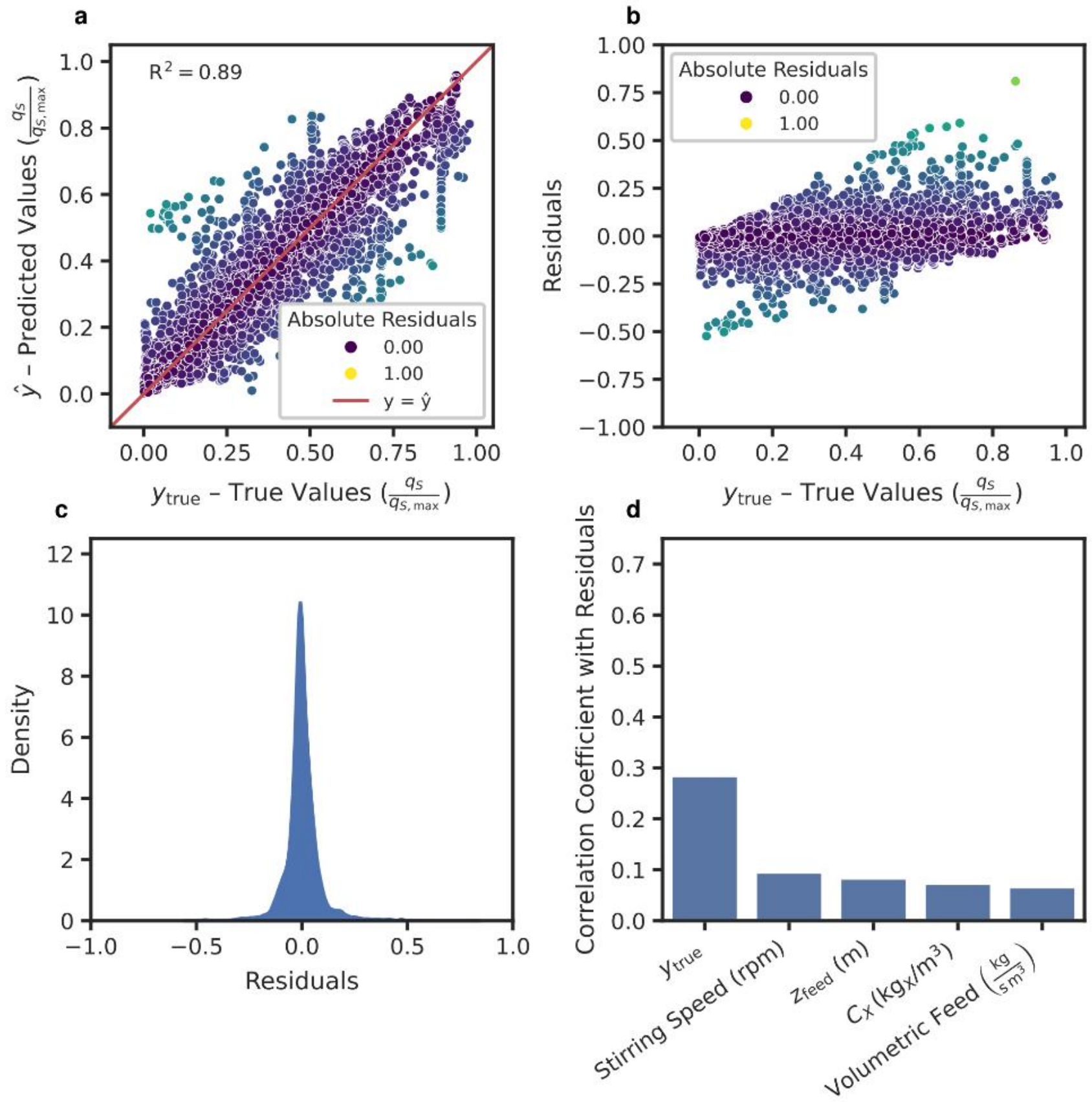

Supplementary Fig. 30. Residual diagnostics for Focused Attention NN on the uptake-based target $q_S/q_{S,\max}$. Residual diagnostics on the independent test set. **a**, Predicted values $\hat{y}$ versus CFD reference values y_{true}; the red dashed line denotes the $1:1$ reference and the annotated value reports $\mathbf{R^2}$. **b**, Residuals $\varepsilon = y_{\text{true}} - \hat{y}$ as a function of y_{true}, with points coloured by residual magnitude. **c**, Residual-density distribution. **d**, Correlations between residuals and individual model predictors.

Supplementary Discussion 5. Interpolation along one-dimensional operating-condition slices

To assess whether the surrogate models behave smoothly between sampled operating points, we performed one-dimensional interpolation analyses using representative CFD case setups. This analysis was not intended to exhaustively validate the full 11-dimensional input space, which would be computationally prohibitive, but rather to assess whether surrogate predictions remain well behaved along selected operating directions within the sampled domain.

The three interpolation sweeps were not generated from a single common base case. Instead, each sweep was constructed from an independent representative CFD setup, with its own operating conditions and local monitoring point (Supplementary Table 5). This design reduces dependence on any single favorable case and provides a broader assessment of interpolation behavior across distinct regions of the sampled operating domain.

Supplementary Table 5. Representative CFD case setups used for the one-dimensional interpolation sweeps. Each column corresponds to an independent base case used for one interpolation analysis. For each sweep, only the swept variable was varied; all other global inputs and the monitoring point were held fixed.

Global condition	$q_{S,\max}$ sweep	y_{feed} sweep	stirring speed sweep

**stirring speed** (rpm)	214	76	$5-700$
**liquid height** (m)	6.35	9.97	10.84
x_{feed} (m)	1.5	1.5	1.5
y_{feed} (m)	6.1	$1-10$	5.42
z_{feed} (m)	0.5	0.5	0.5
F_V $(\text{kg}_S\ \text{m}^{-3}\ \text{s}^{-1})$	2.1×10^{-3}	1×10^{-3}	4.6×10^{-3}
μ $(\text{kg m}^{-1}\ \text{s}^{-1})$	0.01	0.0104	0.0196
ρ (kg m^{-3})	1163	1199	1010
C_X $(\text{kg}_X\ \text{m}^{-3})$	35	37	42
K_S $(\text{kg}_S\ \text{kg}^{-1})$	1.38×10^{-4}	7.17×10^{-5}	1.40×10^{-4}
$q_{S,\max}$ $(\text{kg}_S\ \text{kg}_X^{-1}\ \text{s}^{-1})$	$7\times10^{-5} - 4.9 \times 10^{-4}$	1.7×10^{-4}	2×10^{-4}
Monitoring point coordinates (x, y, z)	$(0.56, 1.81, -0.3)$	$(0.53, 7.67, 0.82)$	$(0.13, 4.18, -1.96)$

Within each sweep, one global variable was varied while the remaining inputs were held fixed. We considered three operating directions: $q_{S,\max}$, which controls the maximum substrate-uptake capacity; y_{feed}, which changes the spatial relationship between the feed and the monitoring point; and stirring speed, which modifies mixing intensity. CFD reference values of $q_S/q_{S,\max}$ were compared with predictions from representative surrogate models, including MLR, Poly3-Lasso, Modified-Attention NN and Attention NN.

Across the tested slices, surrogate predictions varied smoothly without jagged or discontinuous behavior (Supplementary Fig. 31). In general, the NN-derived models reproduced the main CFD trends more closely than the linear MLR baseline, although some local pointwise deviations remained. These results support the use of the surrogates for within-domain screening across the sampled operating space while remaining limited to the selected one-dimensional directions examined here.

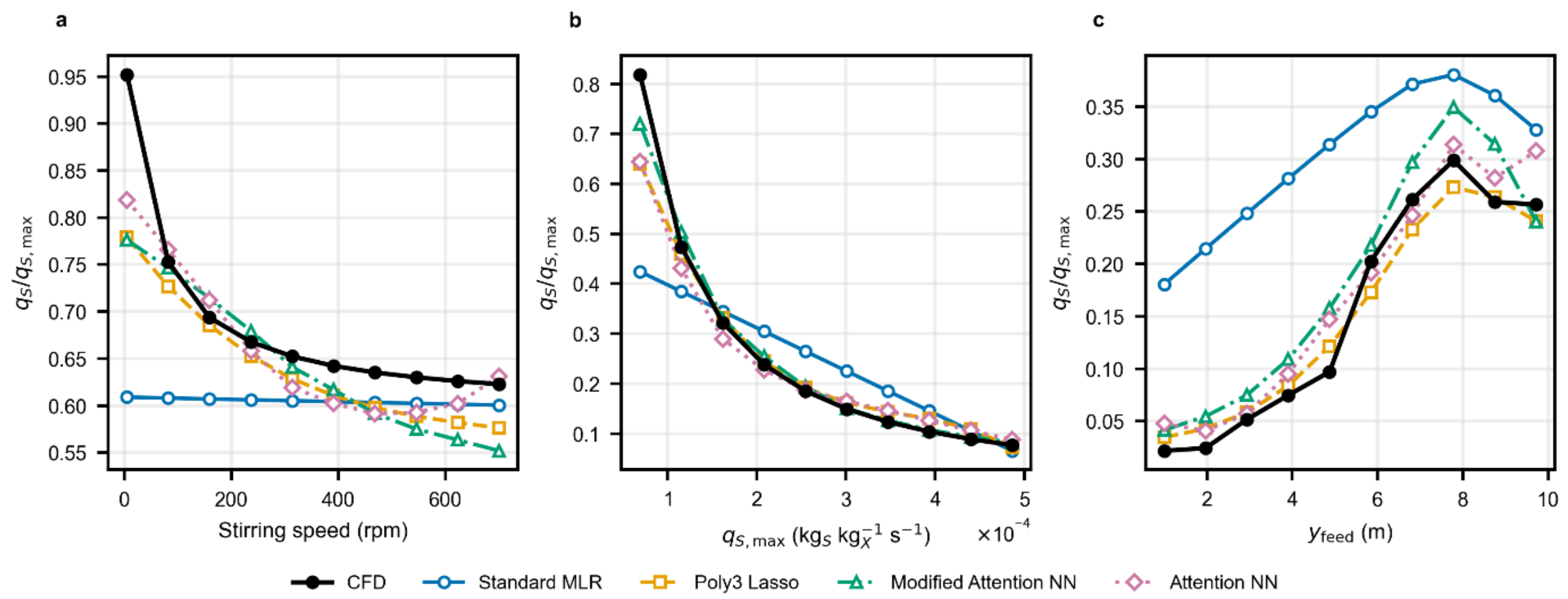

Supplementary Fig. 31. Interpolation along independent one-dimensional operating slices. CFD reference values and surrogate predictions along three independently selected operating-condition sweeps. Each panel corresponds to a different CFD base case and monitoring point defined in Supplementary Table 5, with one global variable varied while all remaining operating conditions and the monitoring point were held fixed. **a**, sweep in $q_{S,\max}$. **b**, sweep in y_{feed}. **c**, sweep in **stirring speed**. Predictions are shown for MLR, Poly3-Lasso, Modified-Attention NN and Attention NN.

Supplementary Discussion 6. Learning curve analysis for alternative target representations

Learning curves for the alternative target representations were generated using the same simulation-level subsampling strategy as the primary analysis (Fig. 3). For each target, model performance was evaluated as a function of the number of unique CFD simulations available for model development using the fixed optimized hyperparameters. Validation MSE and R^2 are reported in Supplementary Figs. 32–36, with four representative model architectures highlighted and the remaining benchmarked models shown in grey. These analyses complement the primary learning-curve analysis and indicate whether the bias- and variance-limited behaviours identified for the uptake-based target are retained under alternative response transformations.

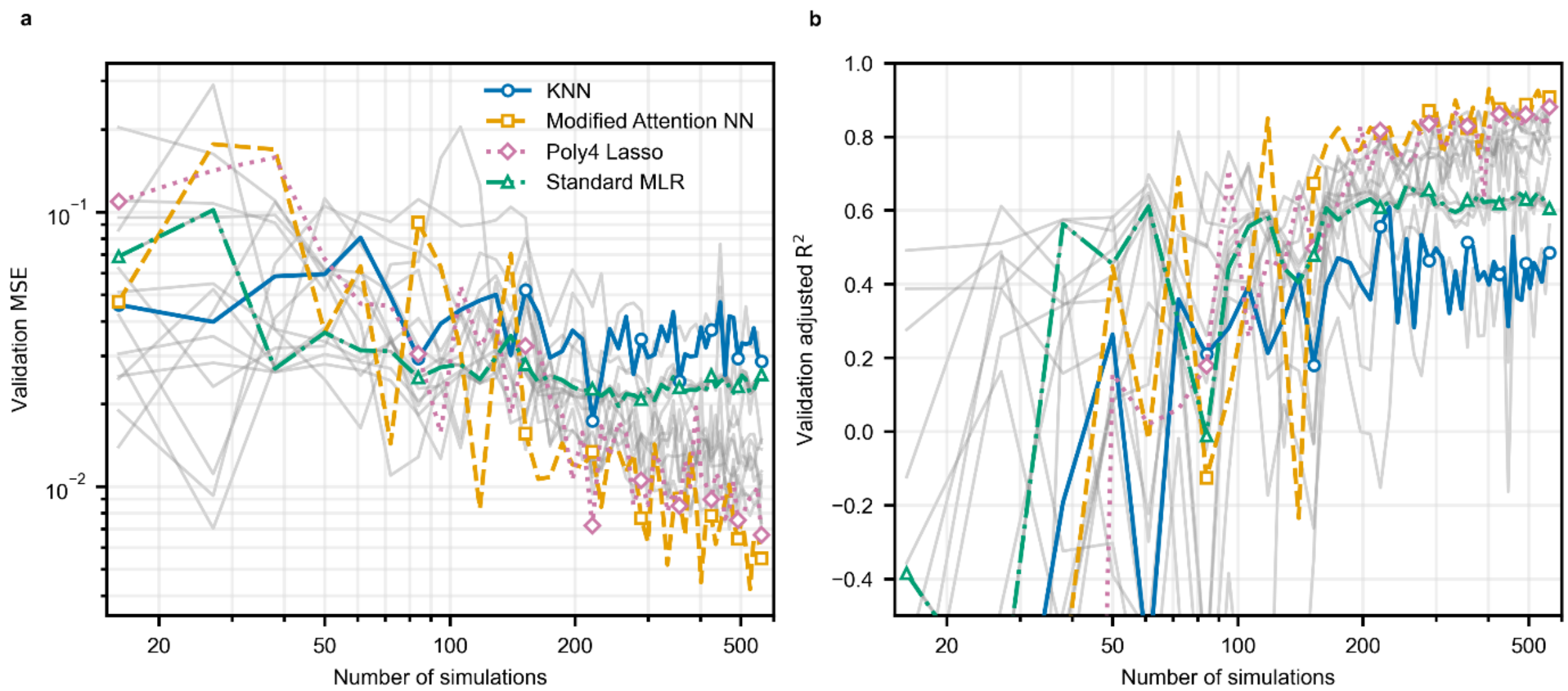

Supplementary Fig. 32. Learning curve analysis for $q_S/q_{S,\max}$. Validation performance as function of the number of CFD simulations used for model development. **a**, mean squared error (**MSE**). **b**, adjusted coefficient of determination ($\mathbf{R^2}$). Four representative models are highlighted, while the remaining benchmarked models are shown in grey. Performance is displayed on a logarithmic scale.

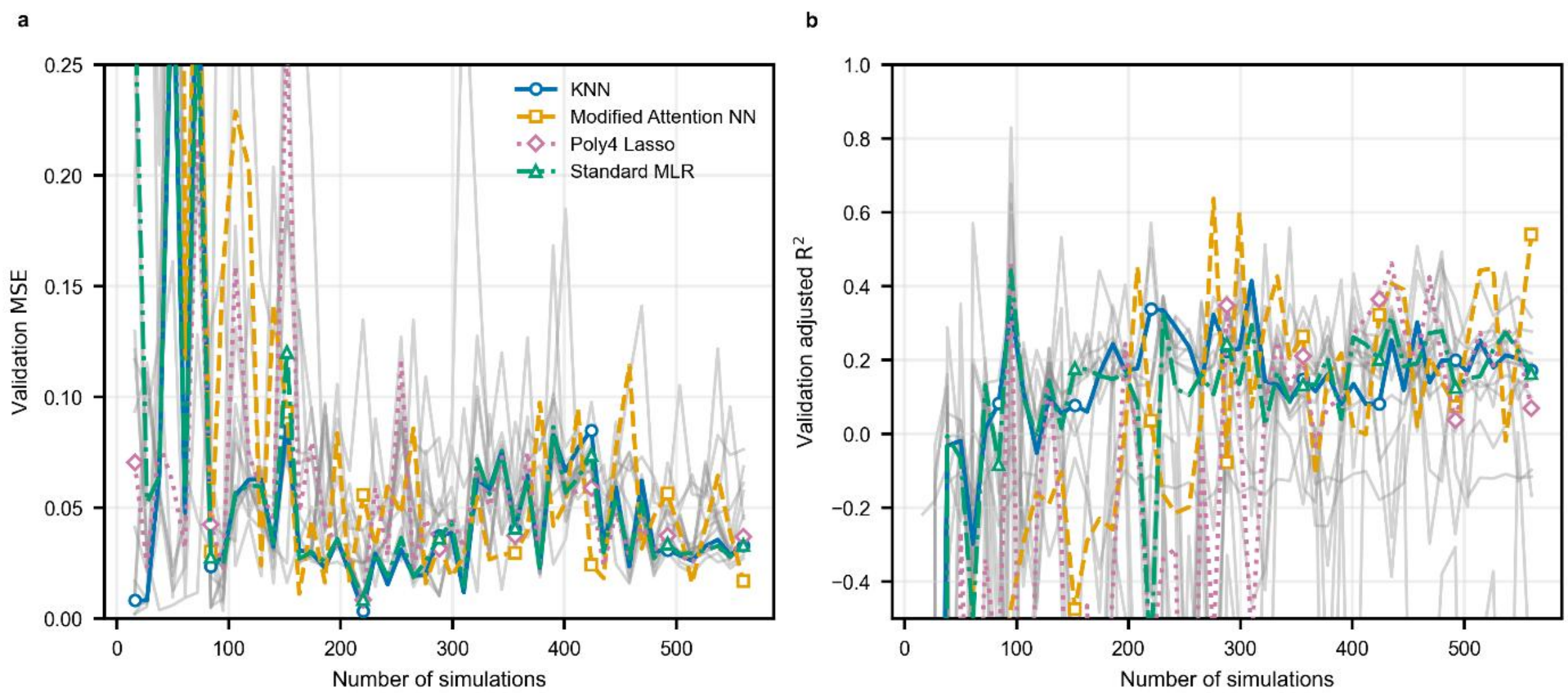

Supplementary Fig. 33. Learning curve analysis for C_S. Validation performance as function of the number of CFD simulations used for model development. **a**, mean squared error (**MSE**). **b**, adjusted

coefficient of determination ($\mathbf{R^2}$). Four representative models are highlighted, while the remaining benchmarked models are shown in grey.

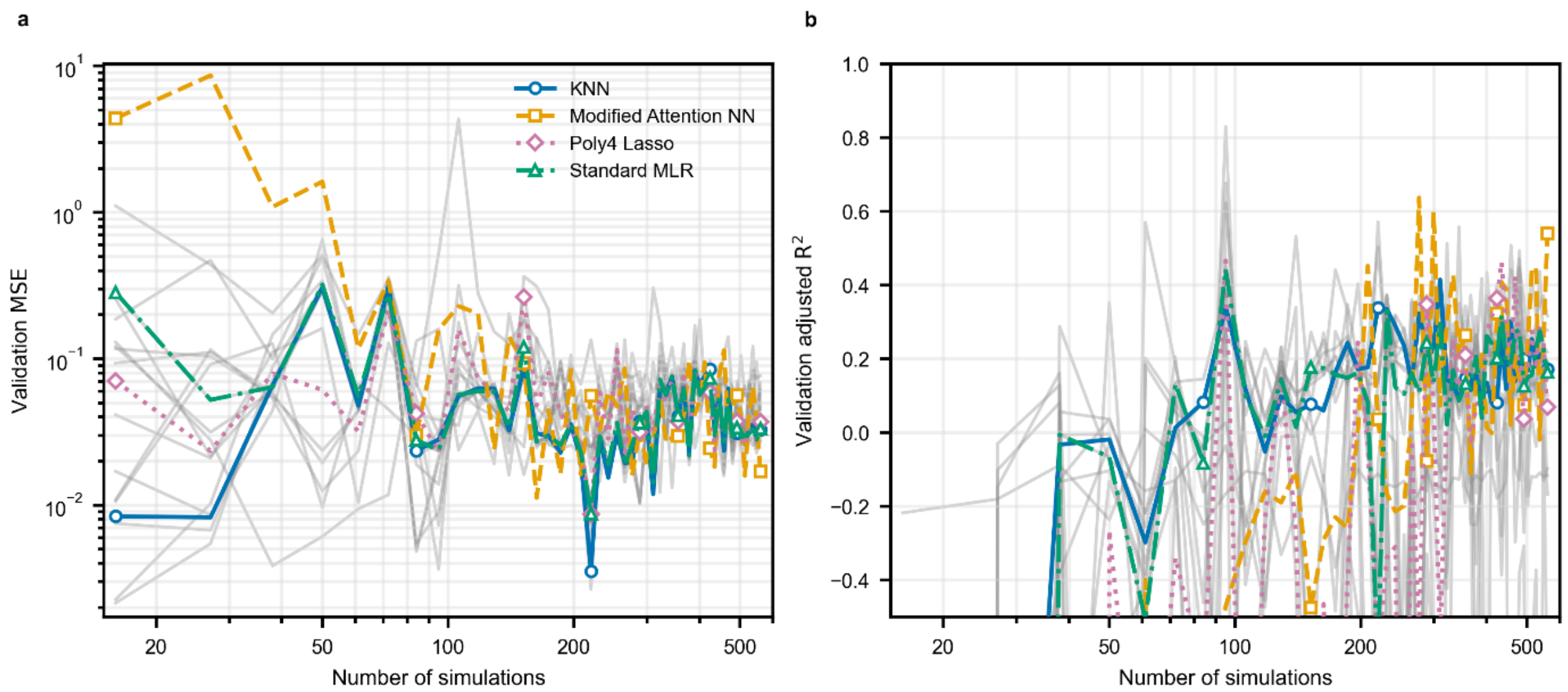

Supplementary Fig. 34. Learning curve analysis for C_S. Validation performance as function of the number of CFD simulations used for model development. **a**, mean squared error ($\mathbf{MSE}$). **b**, adjusted coefficient of determination ($\mathbf{R^2}$). Four representative models are highlighted, while the remaining benchmarked models are shown in grey. Performance is displayed on a logarithmic scale.

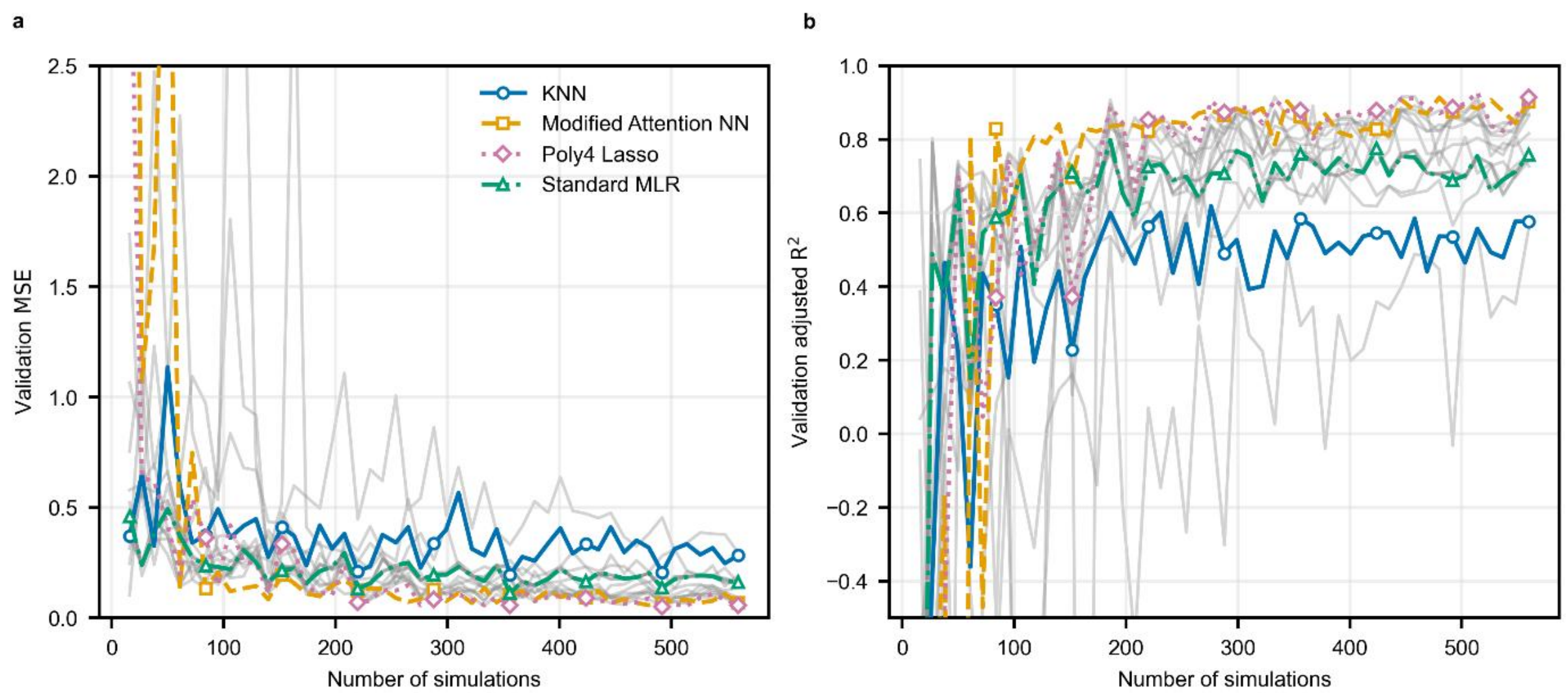

Supplementary Fig. 35. Learning curve analysis for $\log_{10} C_S$. Validation performance as function of the number of CFD simulations used for model development. **a**, mean squared error ($\mathbf{MSE}$). **b**, adjusted coefficient of determination ($\mathbf{R^2}$). Four representative models are highlighted, while the remaining benchmarked models are shown in grey.

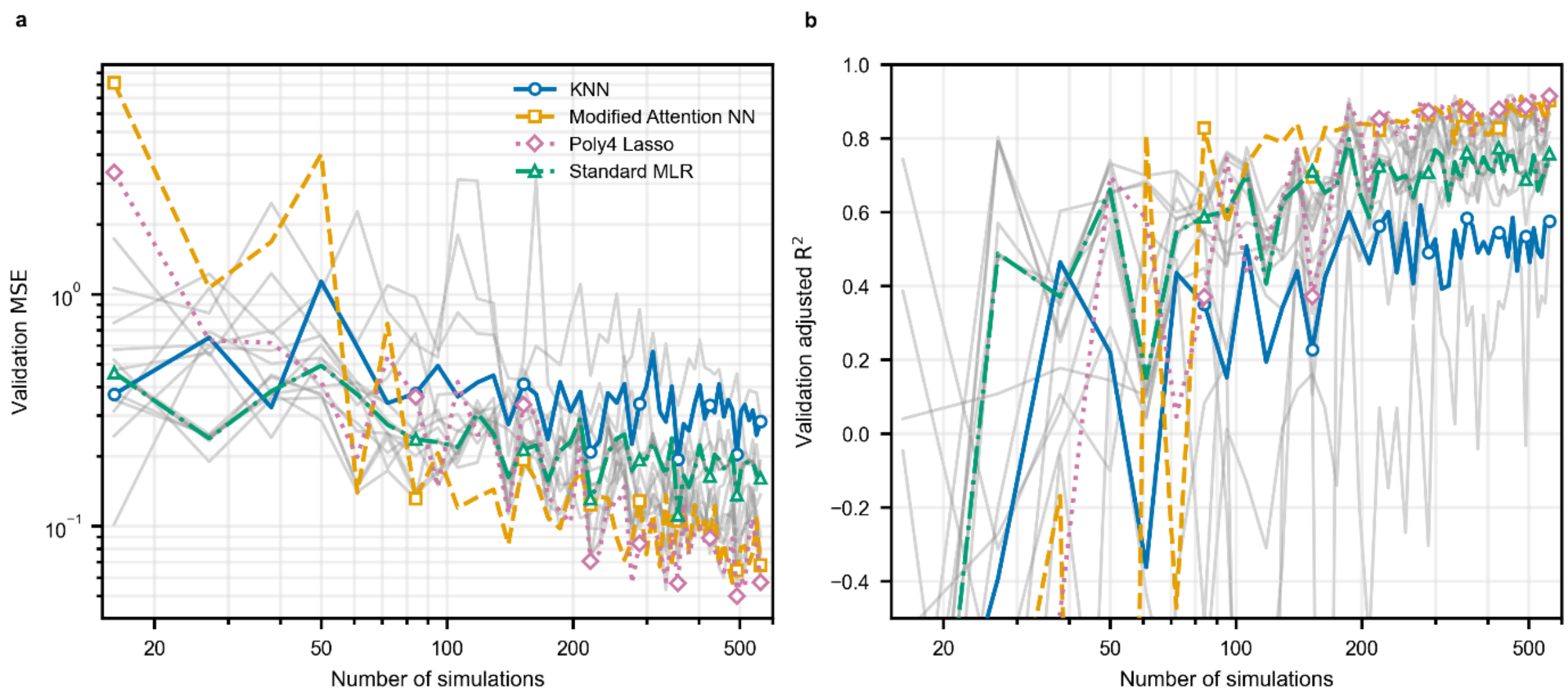

Supplementary Fig. 36. Learning curve analysis for $\log_{10} C_S$. Validation performance as function of the number of CFD simulations used for model development. **a**, mean squared error ($\mathbf{MSE}$). **b**, adjusted coefficient of determination ($\mathbf{R^2}$). Four representative models are highlighted, while the remaining benchmarked models are shown in grey. Performance is displayed on a logarithmic scale.

Supplementary Methods

Supplementary Methods 1. Cylindrical-to-Cartesian coordinate transformation

Feeding-point positions were sampled in cylindrical coordinates to preserve rotational symmetry in the design space and were subsequently transformed into Cartesian coordinates before geometry construction. For radial coordinate r, angular coordinate θ and axial coordinate y_{cyl}, the corresponding Cartesian coordinates were calculated as,

$$x = r \cdot \cos(\theta)$$
$$z = r \cdot \sin(\theta)$$
$$y = y_{\text{cyl}}$$

(1)

Supplementary Methods 2. Parametric geometry of the Rushton-impeller stirred tank

The stirred-tank geometry used for the CFD simulations was parametrically defined to maintain a consistent reactor configuration across the sampled operating conditions. The principal vessel, impeller, baffle, cooling-coil and sparger dimensions used to construct the geometry are summarized in Supplementary Table 6.

Supplementary Table 6. Parametric dimensions of the Rushton-impeller stirred-tank geometry.

Dimensions	Description	Value
$\boldsymbol{H}_{\mathbf{V}}$	Vessel height	12.5 m
$\boldsymbol{A}_{\mathbf{V}}$	Aspect ratio vessel	0.35

$\boldsymbol{D}_{\mathbf{V}}$	Vessel diameter	$H_V \cdot A_V$
$\boldsymbol{R}_{\mathbf{B,V}}$	Radius bottom vessel	$0.6 \cdot H_V$
$\boldsymbol{F}_{\mathbf{S,V}}$	Fillet sketch skeleton vessel	$0.08 \cdot H_V$
$\boldsymbol{C}_{\mathbf{B,I}}$	Bottom impeller clearance	$0.12 \cdot H_V$
$\boldsymbol{C}_{\mathbf{T,I}}$	Top impeller clearance	$0.2 \cdot H_V$
$\boldsymbol{N}_{\mathbf{I}}$	Number of impellers	4
$\mathbf{Dist}_{\mathbf{B,I}}$	Distance between impellers	$(H_V - C_{T,I} - C_{B,I}) / (N_I - 1)$
$\boldsymbol{D}_{\mathbf{shaft}}$	Shaft diameter	$0.016 \cdot H_V$
$\boldsymbol{H}_{\mathbf{C}}$	Height cooling coil	$0.86 \cdot H_V$
$\boldsymbol{W}_{\mathbf{C}}$	Width cooling coil	$0.02 \cdot H_V$
$\boldsymbol{C}_{\mathbf{C}}$	Bottom clearance cooling coil	$0.09 \cdot H_V$
$\boldsymbol{C}_{\mathbf{C,S}}$	Side wall tank–cooling coil clearance	$0.006 \cdot H_V$
$\boldsymbol{P}_{\mathbf{C}}$	Pitch helix cooling coil	$0.04 \cdot H_V$
$\boldsymbol{H}_{\mathbf{B}}$	Height baffle	$0.96 \cdot H_V$
$\boldsymbol{L}_{\mathbf{B}}$	Length baffle	$0.032 \cdot H_V$
$\boldsymbol{W}_{\mathbf{B}}$	Width baffle	$0.125 \cdot L_B$
$\boldsymbol{C}_{\mathbf{B,S}}$	Side tank wall–baffle clearance	$0.0332 \cdot H_V$
$\boldsymbol{N}_{\mathbf{B}}$	Number of baffles	4
$\boldsymbol{R}_{\mathbf{S}}$	Diameter sparger	$0.06 \cdot H_V$
$\boldsymbol{W}_{\mathbf{S}}$	Width sparger	$0.02 \cdot H_V$
$\boldsymbol{W}_{\mathbf{S,C}}$	Width sparger connector	$0.012 \cdot H_V$
$\boldsymbol{\beta}_{\mathbf{S,C}}$	Sparger connector plane angle	$45°$
$\boldsymbol{S}_{\mathbf{C}}$	Sparger clearance	$0.06 \cdot H_V$
$\boldsymbol{R}_{\mathbf{R,I}}$	Radius Rushton impeller	$0.93\ \mathrm{m}$
$\boldsymbol{L}_{\mathbf{R}}$	Length Rushton blade	$0.75 \cdot R_{R,I}$
$\boldsymbol{H}_{\mathbf{R}}$	Height Rushton blade	$0.43 \cdot R_{R,I}$
$\boldsymbol{W}_{\mathbf{R}}$	Width Rushton blade	$0.027 \cdot R_{R,I}$
–	Number of Rushton blades	6
$\boldsymbol{D}_{\mathbf{J,R}}$	Diameter joint disk	$1.24 \cdot R_{R,I}$
$\boldsymbol{H}_{\mathbf{J,R}}$	Height joint disk	$0.054 \cdot R_{R,I}$
$\mathbf{Dist}_{\mathbf{U,C,R}}$	Distance union Rushton blade–shaft connector disk	$0.2 \cdot D_{J,R}$
$\boldsymbol{D}_{\mathbf{C,R}}$	Diameter Rushton impeller–shaft connector disk	$0.3 \cdot D_{J,R}$
$\boldsymbol{H}_{\mathbf{C,R}}$	Height Rushton impeller–shaft connector disk	$0.27 \cdot R_{R,I}$

Supplementary Methods 3. Mesh-independence assessment

To assess mesh independence within the automated CFD workflow, we performed a mesh-density study on five randomly selected cases from the 760-simulation campaign. Repeating this analysis for the full dataset is computationally impractical; therefore, the selected cases were re-simulated using their original operating conditions, including liquid height, fluid properties and stirring speed. For each case, we evaluated four mesh densities: coarse mesh (2.5×10^3 cells m^{-3}), normal–coarse mesh (5.1×10^3 cells m^{-3}), standard mesh used throughout this study (8.8×10^3 cells m^{-3}), and fine mesh (2.5×10^4 cells m^{-3}).

Mesh convergence was assessed using both global and local flow diagnostics. Global integral quantities included torque given by the rotating domain, power input and volume-averaged flow quantities, whereas local convergence was evaluated by monitoring variables such as velocity magnitude and energy dissipation rate at fixed probe locations. These diagnostics were selected to capture both reactor-scale hydrodynamic behavior and local flow sensitivity. The complete diagnostic workflow was applied to all five mesh-independence cases. Because the same convergence trends were observed across cases, we report four representative diagnostics (Supplementary Fig. 37), each from a different case, to illustrate the behavior of the mesh sequence without overloading the Supplementary Information section.

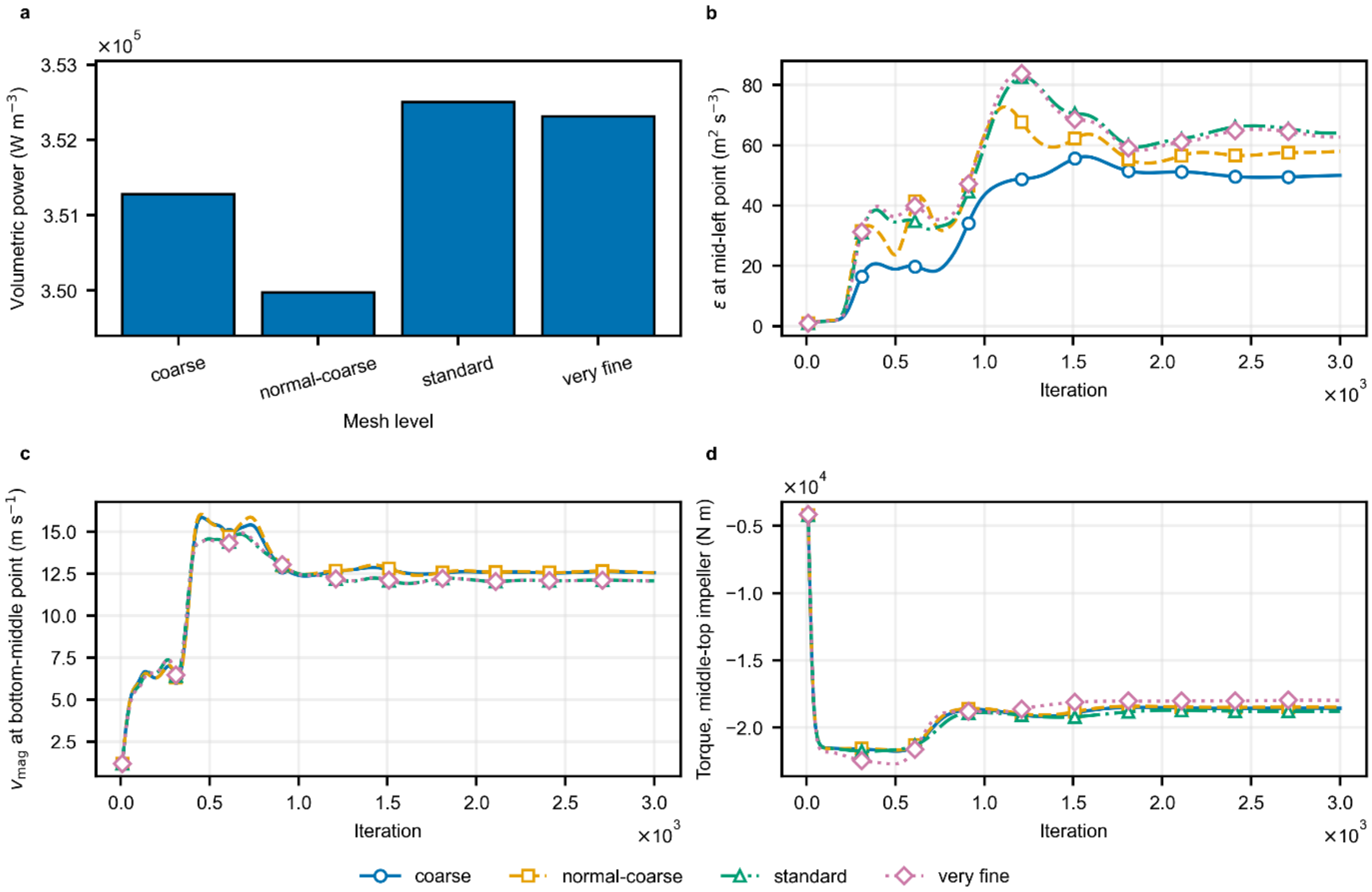

Supplementary Fig. 37. Mesh-density sensitivity analysis for representative CFD cases. Each panel compares four mesh densities, coarse ($\mathbf{2.5 \times 10^3}$ **cells** $\mathbf{m^{-3}}$), normal–coarse ($\mathbf{5.1 \times 10^3}$ **cells** $\mathbf{m^{-3}}$), standard ($\mathbf{8.8 \times 10^3}$ **cells** $\mathbf{m^{-3}}$), and fine ($\mathbf{2.5 \times 10^4}$ **cells** $\mathbf{m^{-3}}$). **a**, final power input for case with **5.06 m** liquid height, showing limited sensitivity of the global integral quantity to further mesh refinement. **b,** temporal evolution of the local energy dissipation rate at a fixed lower-middle/back probe for case with liquid height **4.06 m**. **c,** temporal evolution of the local velocity magnitude (v_{mag}) at a fixed lower/back probe location for the case with **7.91 m** liquid height. **d**, temporal evolution of the bottom-impeller torque for the case with liquid height of **9.57 m**, showing convergence of the global hydrodynamic response across mesh densities. The same diagnostics were generated for all five mesh-independence cases and showed similar convergence trends; only representative panels are shown here for conciseness.

The representative results show that the standard mesh provides a good compromise between numerical accuracy and computational cost. Global quantities, such as power input and bottom-impeller torque, changed only weakly across mesh densities, indicating that the large-scale hydrodynamics were already well captured. Local monitoring points were more sensitive to mesh density, as expected for turbulent stirred-tank simulations, but the standard and fine meshes generally converged to the same dynamic regime and showed very similar steady behavior. In contrast, the coarser meshes occasionally produced shifted local values or different transient responses. We therefore selected the standard mesh for the full simulation campaign.

Although a slightly coarser mesh may have been sufficient for most global quantities, the standard mesh was retained as a conservative choice as it is not possible to run a mesh study for all the simulations used in this study.

Supplementary Methods 4. Hydrodynamic consistency assessment against power-number correlations

To complement the mesh-independence analysis, we assessed whether the automated CFD workflow produced hydrodynamic quantities consistent with classical stirred-tank theory.

We selected 3 representative operating points with different liquid heights ($5.89, 9.23$ and $9.67\ \mathrm{m}$), broth rheological properties, stirring speeds and number of submerged impellers, because validating every case in the 760-simulation dataset against literature correlations was not feasible.

We focused on the impeller power number ($\mathrm{N_p}$), since it is a standard dimensionless quantity used to compare stirred-tank power draw across scales, fluid properties and operating conditions. For each representative case, the impeller Reynolds number was calculated as

$$\mathrm{Re}_i = \frac{\rho N D_i^2}{\mu},$$

(2)

where ρ is the liquid density, N is the impeller rotational frequency, D_i is the impeller diameter and μ is the dynamic viscosity. The mechanical power input was calculated from the shaft-axis torque as

$$P = 2\pi N M_z,$$

(3)

where M_z is the summed torque over the rotating impeller and shaft zones (rotating parts). We defined the corresponding total power number as [1,2]

$$\mathrm{N_{p,total}} = \frac{P}{\rho N^3 D_5^i}.$$

(4)

For multi-impeller configurations, we report an effective per-impeller power number [3],

$$\mathrm{N_{p,imp}} = \frac{\mathrm{N_{p,total}}}{n_{\mathrm{imp}}},$$

(5)

where n_{imp} is the number of submerged impellers.

All representative cases were in fully turbulent regime ($\mathrm{Re}_i > 10^4$), where the Rushton power number is expected to become approximately independent of the Reynolds number and mainly depend on impeller and vessel geometry [1–3]. In this regime, classical Rushton-impeller power-characteristic data and the Doran stirred-tank correlation give a typical turbulent value of approximately $\mathrm{N_p} = 5$-6 for baffled tanks [1–3]. The CFD-derived per impeller power numbers were $\mathrm{N_p} = 4.16$-4.28, with a mean of 4.21, a standard deviation of 0.06, and a coefficient of variation

of only 1.5 % (Supplementary Fig. 38). Although these values are slightly lower than the strict $N_p = 5\text{-}6$ textbook band, they are of the same order and remain nearly constant across cases with different liquid heights, stirring speeds, fluid properties and number of submerged impellers. This collapse is consistent with the expected turbulent-regime behavior of Rushton impellers.

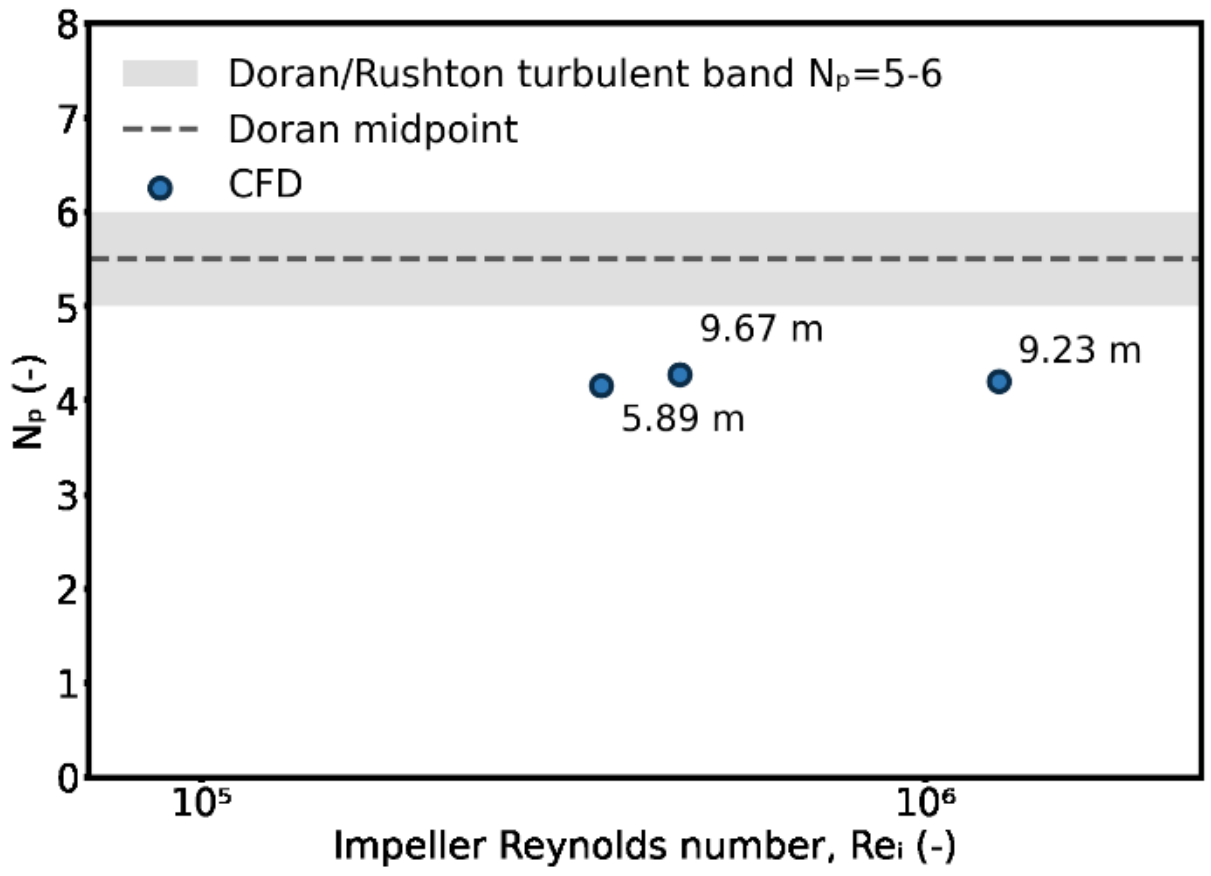

Supplementary Fig. 38. CFD power-number consistency assessment against classical Rushton-impeller references. Effective per-impeller power number, $\mathbf{N_{P,eff}}$, as a function of impeller Reynolds number, $\mathbf{Re}_i$, for the representative CFD cases. The grey band indicates the classical fully turbulent Rushton-impeller reference range reported by Doran[3]. Individual points correspond to the representative operating conditions described in Supplementary Methods 4.

The modest deviation from the ideal N_p reference should be interpreted cautiously. The Doran/Rushton value corresponds to the classical baffled-tank Rushton-impeller correlation, whereas the present simulations involve a tall multi-impeller reactor with internal structures, finite free-surface treatment, shaft contributions and possible inter-impeller interactions. In multi-impeller tanks, the total power draw is not necessarily obtained by simply multiplying a single-impeller reference value by the number of impellers, because axial spacing, hydrodynamic compartmentalization and impeller–impeller interactions can modify power consumption [4,5].

In addition, part of the deviation may originate from the CFD approximations used in this work. The simulations rely on single-phase RANS with MRF treatment of impeller motion and a k-ε-turbulence closure. This is a pragmatic approximation rather than the highest-fidelity description of stirred-tank hydrodynamics; sliding-mesh (SM) and large-eddy simulations (LES) approaches can better resolve blade–baffle interactions, macro-instabilities and transient inter-compartment exchange [6,7].

Nevertheless, RANS/MRF simulations remain widely used in stirred-tank and bioreactor CFD because they provide time-averaged hydrodynamic fields at computational costs compatible with engineering studies and large parametric campaigns. For example, Günyol and Mudde used RANS-based CFD to study hydrodynamics in both standard STR and large-scale multi-impeller fermenter [8], Bach et al. used RANS k-ε modelling to evaluate mixing and mass transfer in a pilot-scale bioreactor [9], and Haringa et al. explicitly assessed MRF-CFD for multi-impeller mixing in comparison with experiments and higher-fidelity transient simulations [6,7].

Together with the mesh-independence study, these hydrodynamic consistency checks support the use of the automated CFD workflow as physically plausible numerical data-generation

pipeline for surrogate model training. Nevertheless, the surrogate models inherit the assumptions and limitations of the underlying CFD formulation. We therefore interpret the CFD simulations as a consistent numerical reference within the stated modelling assumptions, rather than as a complete experimental validation of industrial-scale bioreactor hydrodynamics.

Supplementary Methods 5. Cross-validation, hyperparameter search and model selection

Hyperparameter optimization was performed exclusively within the development dataset using simulation-grouped ten-fold cross-validation (CV), with complete CFD simulations kept within individual folds to prevent leakage between spatially correlated mesh elements. Candidate hyperparameters for the regularized linear and non-neural machine-learning models were evaluated by grid search, whereas the broader neural-network search spaces were explored using the tree-structured Parzen estimator (TPE) [10] implemented in Optuna [11]. The evaluated search spaces and final selected configurations are summarized in Supplementary Tables 7–9, and the neural-network optimization trajectories are shown in Supplementary Figs. 39–42.

From the complete set of 760 CFD simulations, 200 simulations were reserved as an independent test set and were not used for model fitting, hyperparameter optimization, early stopping, architecture selection or any other model-development decision. The remaining 560 simulations constituted the development set used for model fitting and hyperparameter selection.

Hyperparameter optimization was performed using ten-fold cross-validation grouped by complete CFD simulation. After randomization at the simulation level, the development set was divided into ten folds of approximately 56 CFD simulations each. In each CV iteration, one fold was used for validation and the remaining nine folds for training. All mesh elements originating from the same CFD simulation were retained within the same fold. This grouping was necessary because spatial observations from a single CFD field are strongly correlated; distributing mesh elements from one simulation across training and validation folds would introduce information leakage and underestimate generalization error. The mean validation MSE across the ten folds was used as the optimization objective for each hyperparameter configuration.

For the regularized MLR-derived and non-neural machine-learning models, candidate hyperparameter combinations were evaluated using grid search within the simulation-grouped CV framework. The complete search spaces are reported in Supplementary Table 7. For each candidate configuration, the mean validation MSE across the ten CV folds was calculated, and the configuration yielding the lowest mean validation MSE was retained. Polynomial–Lasso was not subjected to hyperparameter optimization; polynomial order was instead evaluated as defined in the main Methods.

Supplementary Table 7. Hyperparameter search spaces for regularized linear and non-neural surrogate models. Candidate hyperparameter values evaluated using simulation-grouped $\mathbf{10}$-fold cross-validation. The mean validation $\mathbf{MSE}$ across the ten folds was used to select the optimal configuration for each model.

<table>
<tr><th>Model</th><th>Hyperparameter</th><th>Candidate values</th></tr>
<tr><td>Lasso</td><td>Regularization parameter (λ)</td><td rowspan="3">$\lambda = 10^{\log 0.01 + i\cdot\frac{\log 10^3 - \log 0.01}{n-1}}$,
for $i = 0, \ldots, n$ and $n = 10^3$</td></tr>
<tr><td>Ridge</td><td>Regularization parameter (λ)</td></tr>
<tr><td rowspan="2">Elastic net</td><td>Regularization parameter (λ)</td></tr>
<tr><td>Mixing parameter (L_1 ratio: α)</td><td>$0.1, 0.5, 0.7, 0.9, 0.95, 0.99$</td></tr>
</table>

**Huber**	Threshold parameter (δ)	$\delta = 10^{\log 1.1 + i \cdot \frac{\log 10^2 - \log 1.1}{n-1}}$, for $i = 0, \ldots, n$ and $n = 10^2$
***k*-Nearest neighbors**	Number of neighbors (k)	$k = i + 1$, for $i = 0, \ldots, 499$
**Decision trees**	Maximum depth trees	$10, 20, 30, 40$
	Minimum samples split	$6, 10, 25, 30$
	Minimum samples leaf	$3, 4, 6, 8$
**Random forest**	Number of trees (T)	$25, 50, 100, 250, 350, 500, 750$
	Maximum number of features (fraction)	$1.0, 0.33, \sqrt{n_f}, \log_2 n_f$
	Minimum samples split	$6, 10, 25, 30$
	Minimum samples leaf	$3, 4, 6, 8, 10$
**Gradient boosting**	Learning rate	$0.001, 0.0025, 0.005, 0.01, 0.05, 0.1$
	Max depth of trees	$5, 10, 20, 30, 40$
	Number of estimators	$50, 100, 250, 500, 10^3, 2.5 \times 10^3, 5 \times 10^3$
**Support vector**	Kernel type	Linear, RBF, poly, sigmoid
	Regularization parameter (C)	$0.1, 1, 10, 100, 1000$
	Kernel coefficient (γ)	$1/n_f, 1/(n_f \cdot \mathrm{var}(X)), 0.01, 0.1, 1.0$
	Loss tube size (ξ)	$0.01, 0.1, 0.2, 0.5, 1.0$

Neural networks required optimization over a broader search space comprising network architecture, learning rate, batch size, dropout rate, early stopping patience and weight decay (Supplementary Table 8). Rather than exhaustively evaluating all combinations, we used the tree-structured Parzen estimator (TPE), a Bayesian optimization algorithm implemented in Optuna[11], within the PyTorch training workflow[12]. Each Optuna trial sampled one hyperparameter configuration and evaluated it using the same simulation-grouped CV strategy, with mean validation MSE as the objective to be minimized.

The optimization trajectory for the Modified-Attention NN across the three target representations evaluated with this architecture is shown in Supplementary Fig. 39. The traces show early identification of high-performing regions followed by continued exploration of the search space. Corresponding optimization trajectories for the Standard NN, Attention NN and Focused-Attention NN are shown in Supplementary Figs. 40–42, respectively. The best-performing hyperparameter configuration identified for each model was subsequently retained for final model fitting.

Supplementary Table 8. Hyperparameter search space for the neural-network models. Candidate neural-network architectures and training hyperparameters explored using TPE-based Bayesian optimization within the simulation-grouped ten-fold cross-validation framework.

Hyperparameter tuned	Values
**Neural network architectures**	$[64, 64, 64] \longrightarrow$ Uniform layers
	$[16, 32, 64, 128] \longrightarrow$ Inverted funnel
	$[32, 32, 32, 32, 32] \longrightarrow$ Deep narrow network
	$[512, 256, 128, 256, 512] \longrightarrow$ Complex funnel
	$[256, 64, 256] \longrightarrow$ Bottleneck
	$[64, 128, 64, 128, 64] \longrightarrow$ Zig-zag pattern
	$[1024, 512] \longrightarrow$ Wide-shallow network

	[512, 256, 128, 64, 32, 16, 8] ⟶ Exponential decay
	[8, 16, 32, 64, 128, 256] ⟶ Exponential growth
	[128] ⟶ Single-layer network
	[8, 4] ⟶ Very simple network
	[128, 128, 128, 128] ⟶ Deep uniform network
	[1024, 512, 256, 128, 64] ⟶ Larger simple funnel
	[64, 64, 128] ⟶ Gradual expansion
	[64, 128, 256] ⟶ Steady growth
	[32,64, 16] ⟶ Narrow expansion + compression
	[16, 32] ⟶ Simple growth
	[8, 16, 32, 8] ⟶ Simple growth + compression
	[16, 32] ⟶ Simple decay
	[32, 128, 64, 32] ⟶ Expansion + compression cycle
**Objective variable**	$C_S, \log C_S$, $^{q_S}/_{q_{S,\max}}$, $\mathrm{qt}(C_S)$
**Batch size**	4096, 2048, 1024, 512, 128, 64, 32
**Dropout**	0, 0.1, 0.25, 0.5, 0.7
**Learning rate**	$2.5 \times 10^{-5}, 5 \times 10^{-5}, 10^{-4}, 0.001, 0.01, 0.1$
**Early stop patience**	$\mathrm{ES} = i + 1, \quad \text{for } i = 5, \ldots, 20$
**Weight decay (regularization)**	$0.1, 10^{-5}, 10^{-4}, 0.001, 0.005, 0.01, 0.05$

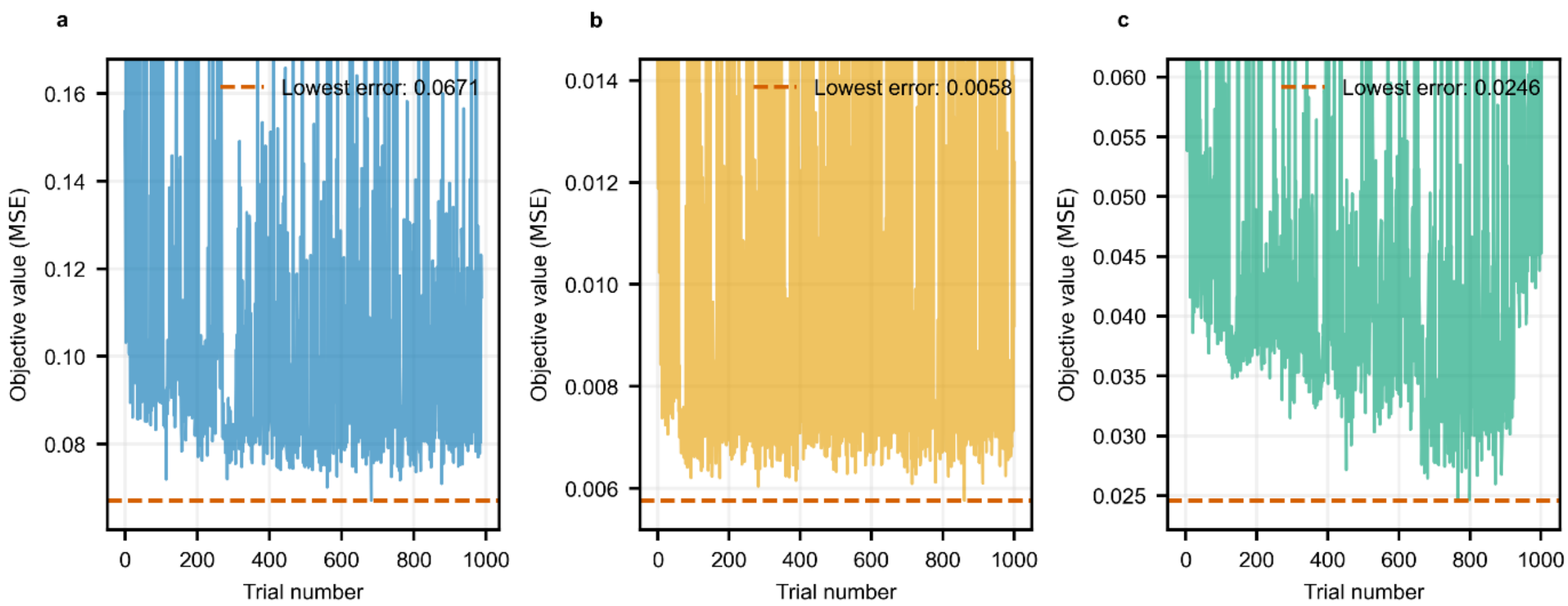

Supplementary Fig. 39. Bayesian hyperparameter optimization of the Modified-Attention NN across target representations. Mean cross-validated validation MSE obtained for each Optuna/TPE trial. **a–c**, Optimization trajectories for $\mathbf{log_{10}}\,\boldsymbol{C_S}$, $^{\boldsymbol{q_S}}/_{\boldsymbol{q_{S,\max}}}$ and $\boldsymbol{C_S}$, respectively. Each trial corresponds to one sampled hyperparameter configuration evaluated using simulation-grouped ten-fold cross-validation; the dashed horizontal line indicates the lowest objective value obtained for the corresponding target.

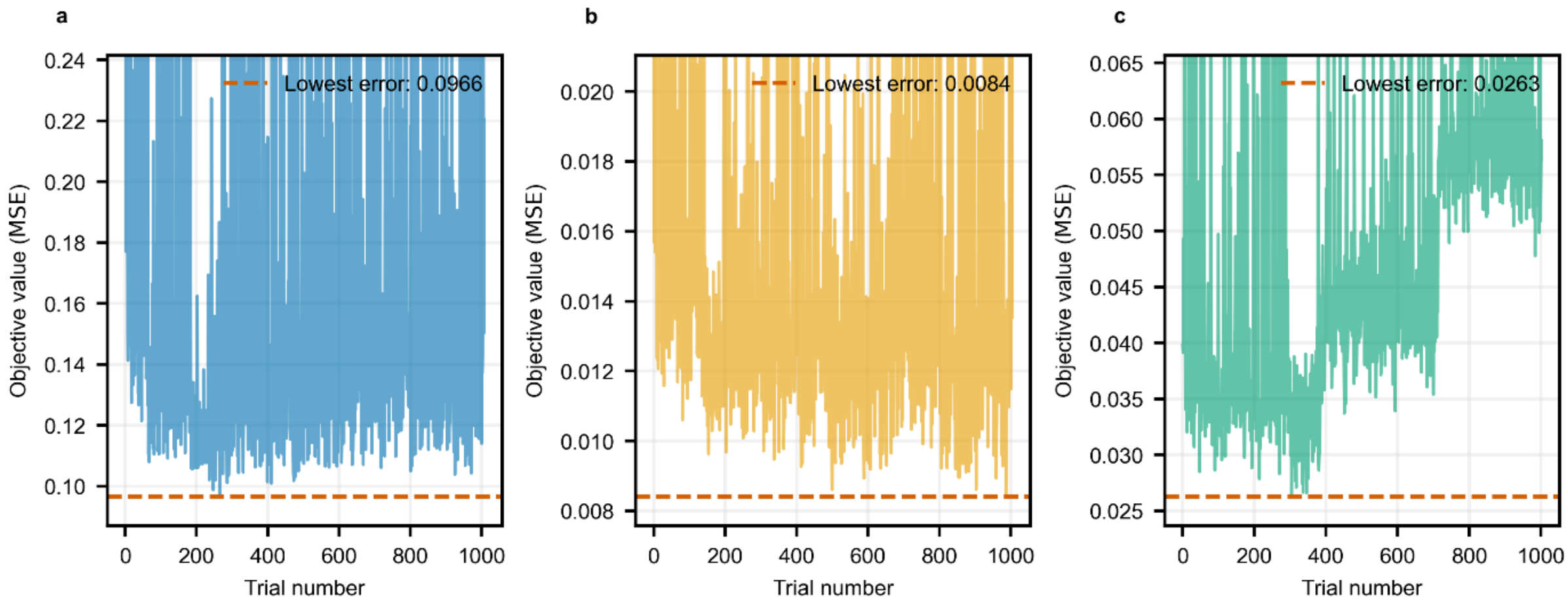

Supplementary Fig. 40. Bayesian hyperparameter optimization of the Standard NN across target representations. Mean cross-validated validation MSE obtained for each Optuna/TPE trial. **a–c**, Optimization trajectories for $\log_{10} C_S$, $q_S/q_{S,\max}$ and C_S, respectively. Each trial corresponds to one sampled hyperparameter configuration evaluated using simulation-grouped ten-fold cross-validation; the dashed horizontal line indicates the lowest objective value obtained for the corresponding target.

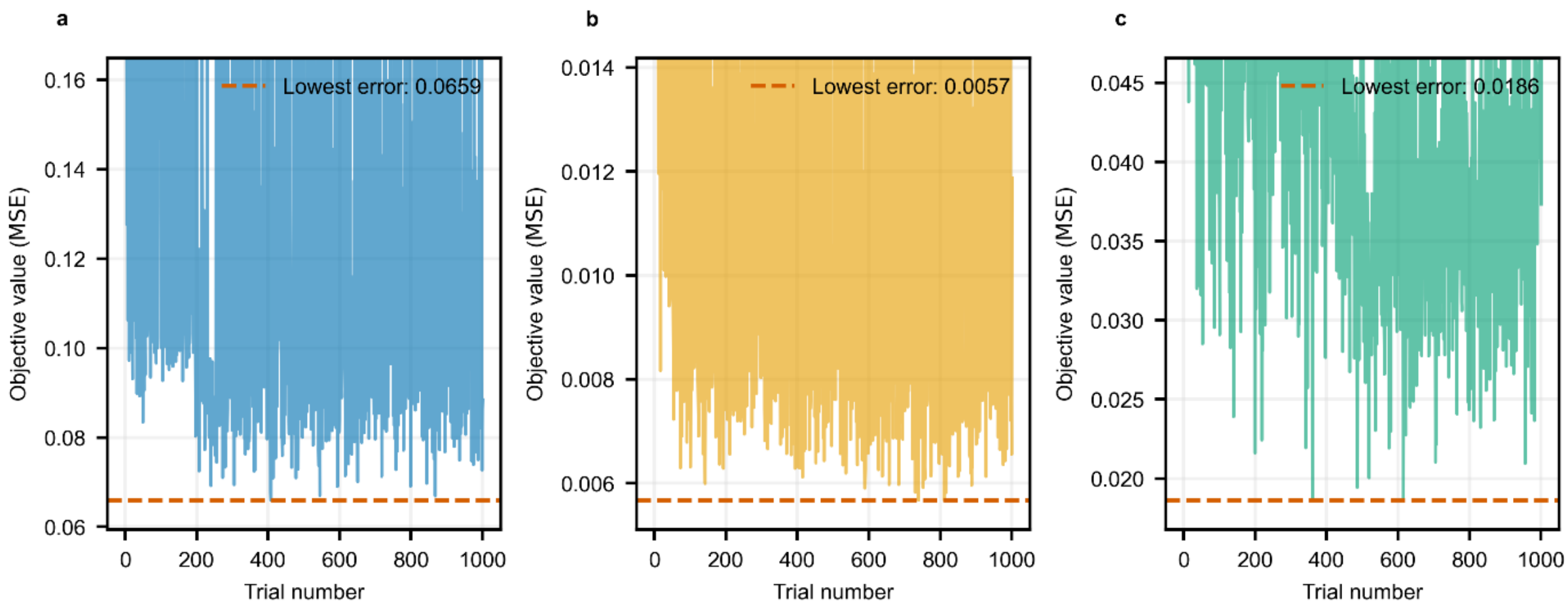

Supplementary Fig. 41. Bayesian hyperparameter optimization of the Attention NN across target representations. Mean cross-validated validation MSE obtained for each Optuna/TPE trial. **a–c**, Optimization trajectories for $\log_{10} C_S$, $q_S/q_{S,\max}$ and C_S, respectively. Each trial corresponds to one sampled hyperparameter configuration evaluated using simulation-grouped ten-fold cross-validation; the dashed horizontal line indicates the lowest objective value obtained for the corresponding target.

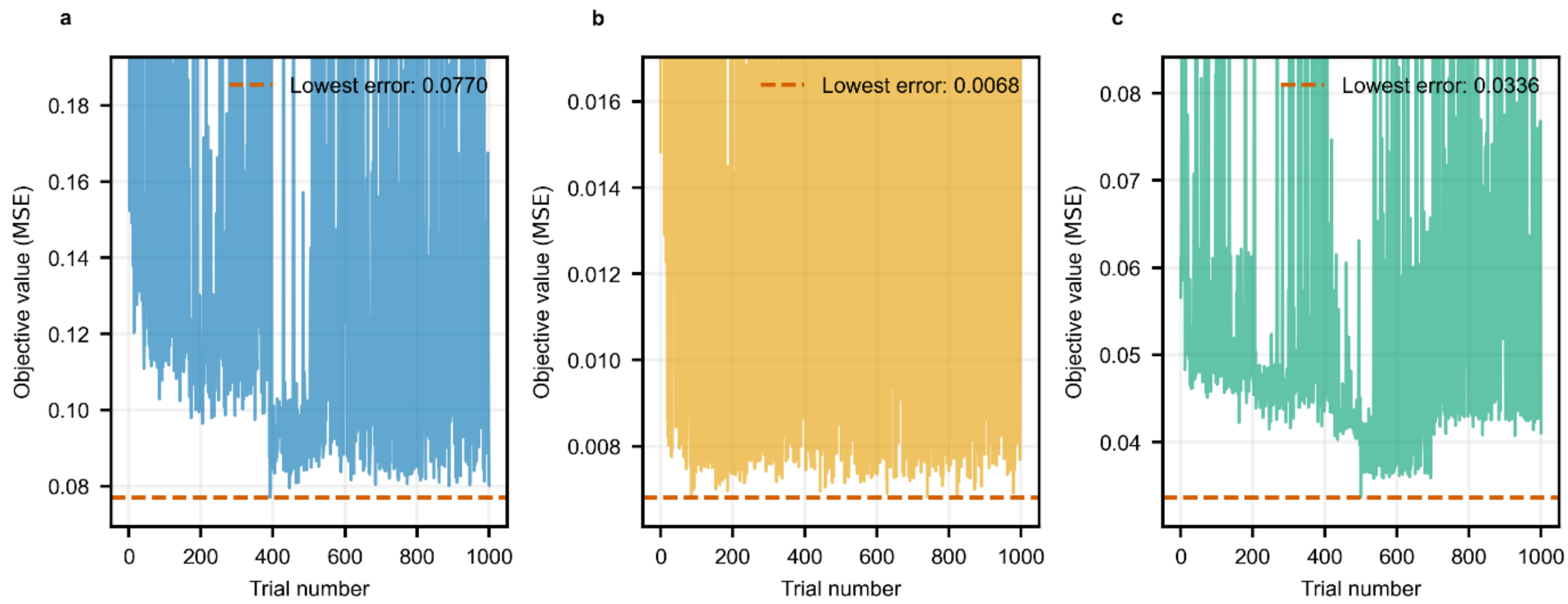

Supplementary Fig. 42. Bayesian hyperparameter optimization of the Focused-Attention NN across target representations. Mean cross-validated validation MSE obtained for each Optuna/TPE trial. **a–c**, Optimization trajectories for $\log_{10} C_S$, $q_S/q_{S,\max}$ and C_S, respectively. Each trial corresponds to one sampled hyperparameter configuration evaluated using simulation-grouped ten-fold cross-validation; the dashed horizontal line indicates the lowest objective value obtained for the corresponding target.

Following hyperparameter optimization, the configuration yielding the lowest mean cross-validated validation MSE was selected for each model. The resulting configurations are summarized in Supplementary Table 9. These hyperparameters were then fixed, and each model was retrained using the corresponding model-specific data fraction sampled from the complete development set. The subsampling fractions and their distributional-fidelity assessment are described separately in Supplementary Methods 7. Following final fitting, predictive performance was evaluated on the independent 200-simulation test set, which had remained completely excluded from hyperparameter selection and model development.

Supplementary Table 9. Selected hyperparameter configurations for the surrogate models. Hyperparameter values selected following simulation-grouped cross-validation and used for final model fitting on the development dataset. For models subjected to hyperparameter optimization, configurations were selected according to the lowest mean cross-validated validation MSE.

Model	Hyperparameter	Selected value
**Lasso**	Regularization parameter: λ	0.011
**Ridge**	Regularization parameter: λ	2.97
**Elastic net**	Regularization parameter: λ	0.02
	Mixing parameter: α	0.10
**Huber**	Threshold parameter: δ	16.2
**k-Nearest neighbors**	Number of neighbors (k)	174
**Decision trees**	Maximum depth trees	10
	Minimum samples split	30
	Minimum samples leaf	4
**Random forest**	Number of trees (T)	500
	Number of features	0.33
	Minimum samples split	6
	Minimum samples leaf	4
**Gradient boosting**	Learning rate	0.05
	Max depth of trees	5

	Number of estimators	2500
**Support vector**	Kernel type	RBF
	Regularization parameter (C)	100
	Kernel coefficient (γ)	0.01
	Loss tube size (ξ)	0.01
**Standard NN**	Architecture	$[128]$
	Learning rate	2.5×10^{-5}
	Dropout	0.5
	Batch size	512
	Early stop patience	18
	Weight decay	0
**Modified-Attention NN**	Architecture	$[16, 32]$
	Learning rate	2.5×10^{-5}
	Dropout	0.5
	Batch size	2048
	Early stop patience	14
	Weight decay	1×10^{-3}
**Attention NN**	Architecture	$[32, 64, 16]$
	Learning rate	2.5×10^{-5}
	Dropout	0
	Batch size	4096
	Early stop patience	20
	Weight decay	1×10^{-4}
**Focused-Attention NN**	Architecture	$[1024, 512]$
	Learning rate	2.5×10^{-5}
	Dropout	0
	Batch size	2048
	Early stop patience	19
	Weight decay	1×10^{-4}

Supplementary Methods 6. Neural-network training dynamics

Training dynamics were examined for the four neural-network architectures—Standard NN, Modified-Attention NN, Attention NN and Focused-Attention NN—across the target representations used for neural-network training. For each architecture, the evolution of the development and test MSE was monitored over the training epochs, together with the mean model prediction and the corresponding mean target value. Training trajectories for the primary uptake-based target ${q_S}/{q_{S,\max}}$ are shown in Supplementary Fig. 43, while the corresponding trajectories for raw substrate concentration C_S and log-transformed substrate concentration $\log_{10} C_S$ are shown in Supplementary Fig. 44 and Supplementary Fig. 45, respectively.

For the uptake-based target, all four neural-network architectures converged rapidly during the initial training period, after which further improvements in performance were comparatively small (Supplementary Fig. 43). Development and test losses followed similar trajectories, indicating limited divergence between model fitting and generalization under the selected

regularization. Early in training, deviations between the mean model prediction and the mean target value indicated an initial prediction bias that decreased as optimization progressed.

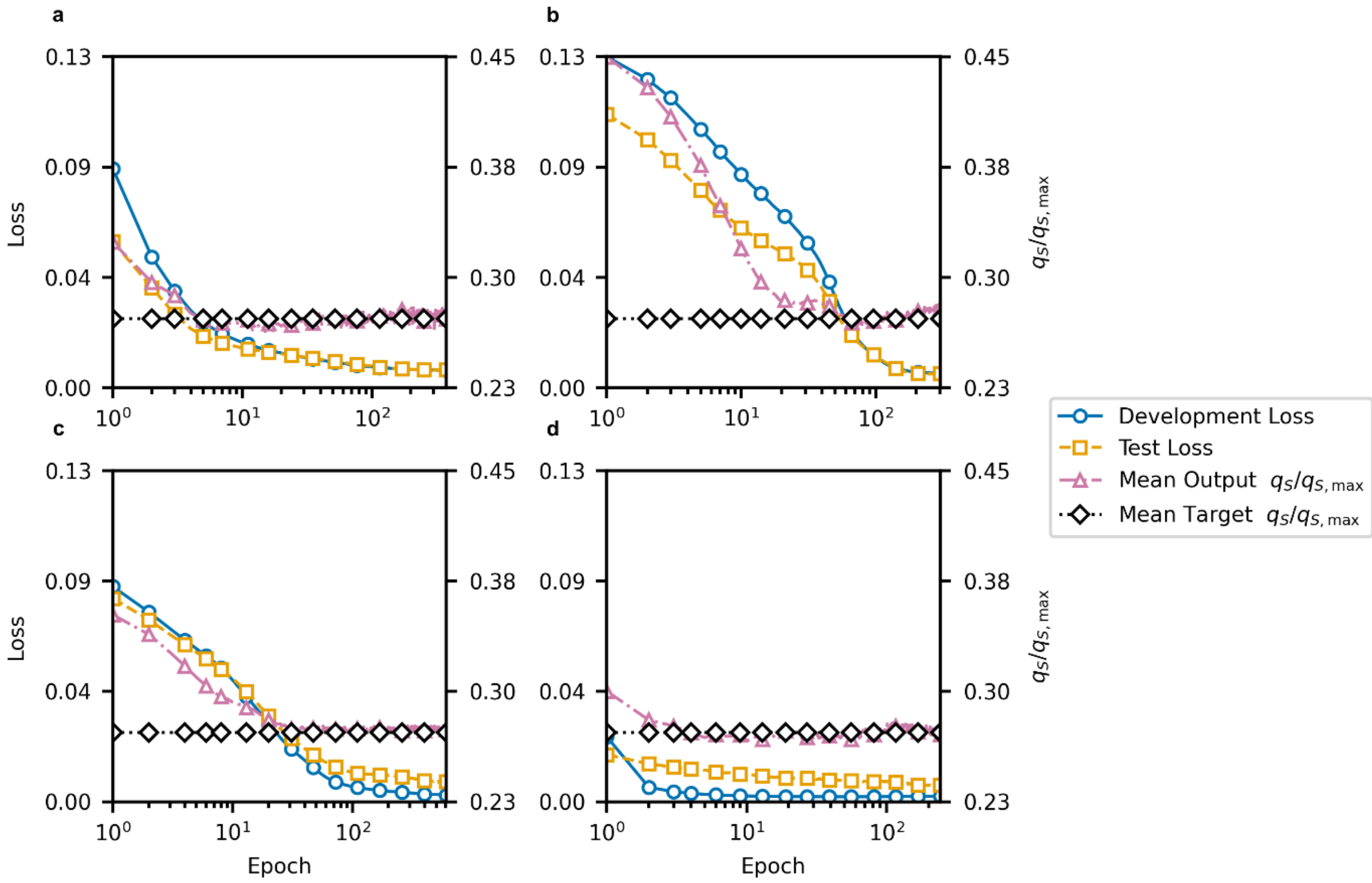

Supplementary Fig. 43. Training dynamics of the neural-network surrogates for the uptake-based target $q_S/q_{S,\mathrm{max}}$. Development and test mean squared error (**MSE**; left axis) and mean test-set prediction (right axis) as functions of training epoch. The epoch axis is logarithmic. The black diamond line indicates the mean target value. **a**, Standard NN. **b**, Modified-Attention NN. **c**, Attention NN. **d**, Focused-Attention NN.

The corresponding trajectories for C_S and $\log_{10} C_S$ are provided in Supplementary Fig. 44 and Supplementary Fig. 45 to document the training behavior under the alternative target representations. Although test-set trajectories are displayed as diagnostic information, all early stopping, architecture selection and hyperparameter-optimization decisions were performed using the development set, as described in the main Methods.

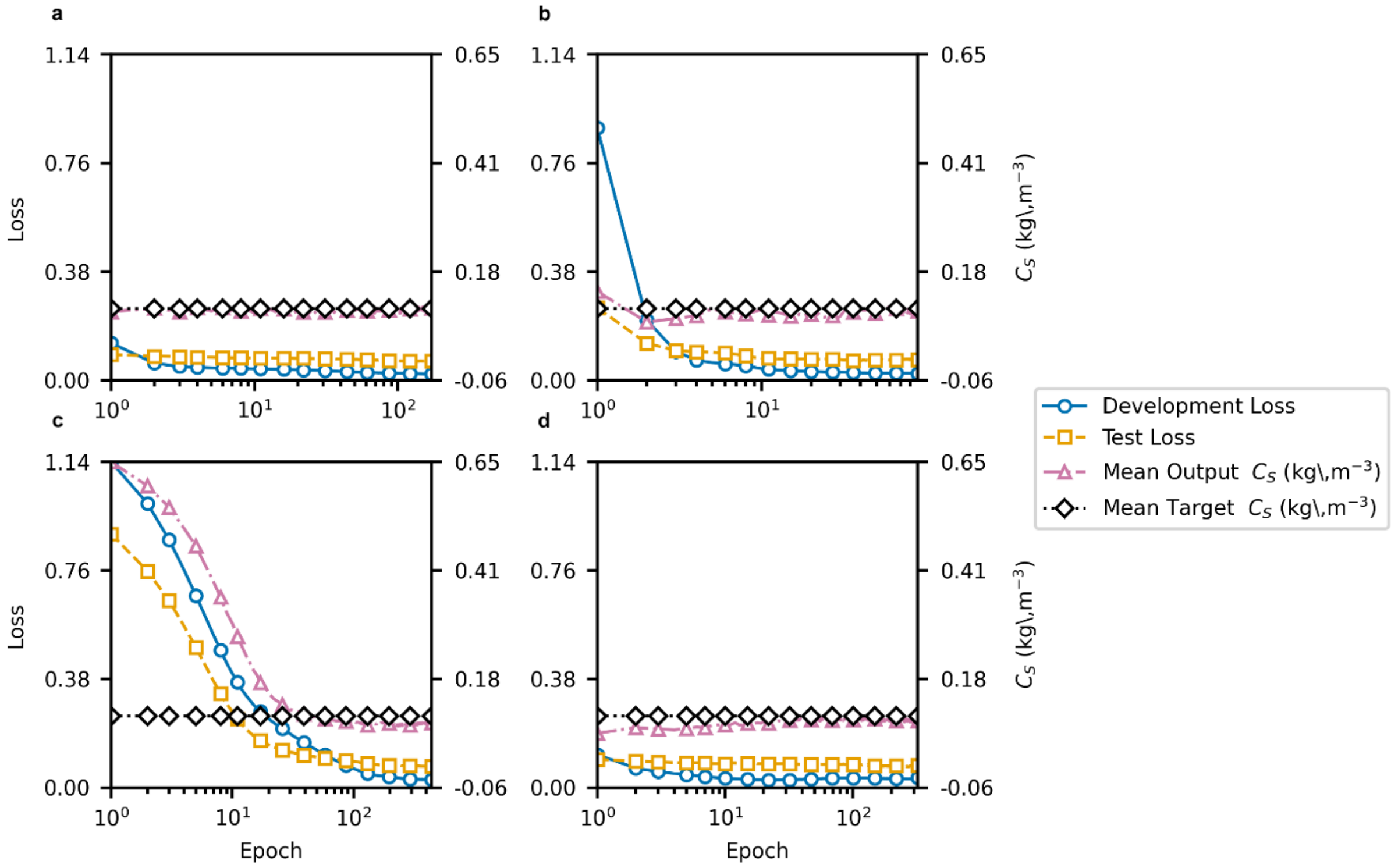

Supplementary Fig. 44. Training dynamics of the neural-network surrogates for substrate concentration C_S. Development and test mean squared error (**MSE**; left axis) and mean test-set prediction (right axis) as functions of training epoch. The epoch axis is logarithmic. The black diamond line indicates the mean target value. **a**, Standard NN. **b**, Modified-Attention NN. **c**, Attention NN. **d**, Focused-Attention NN.

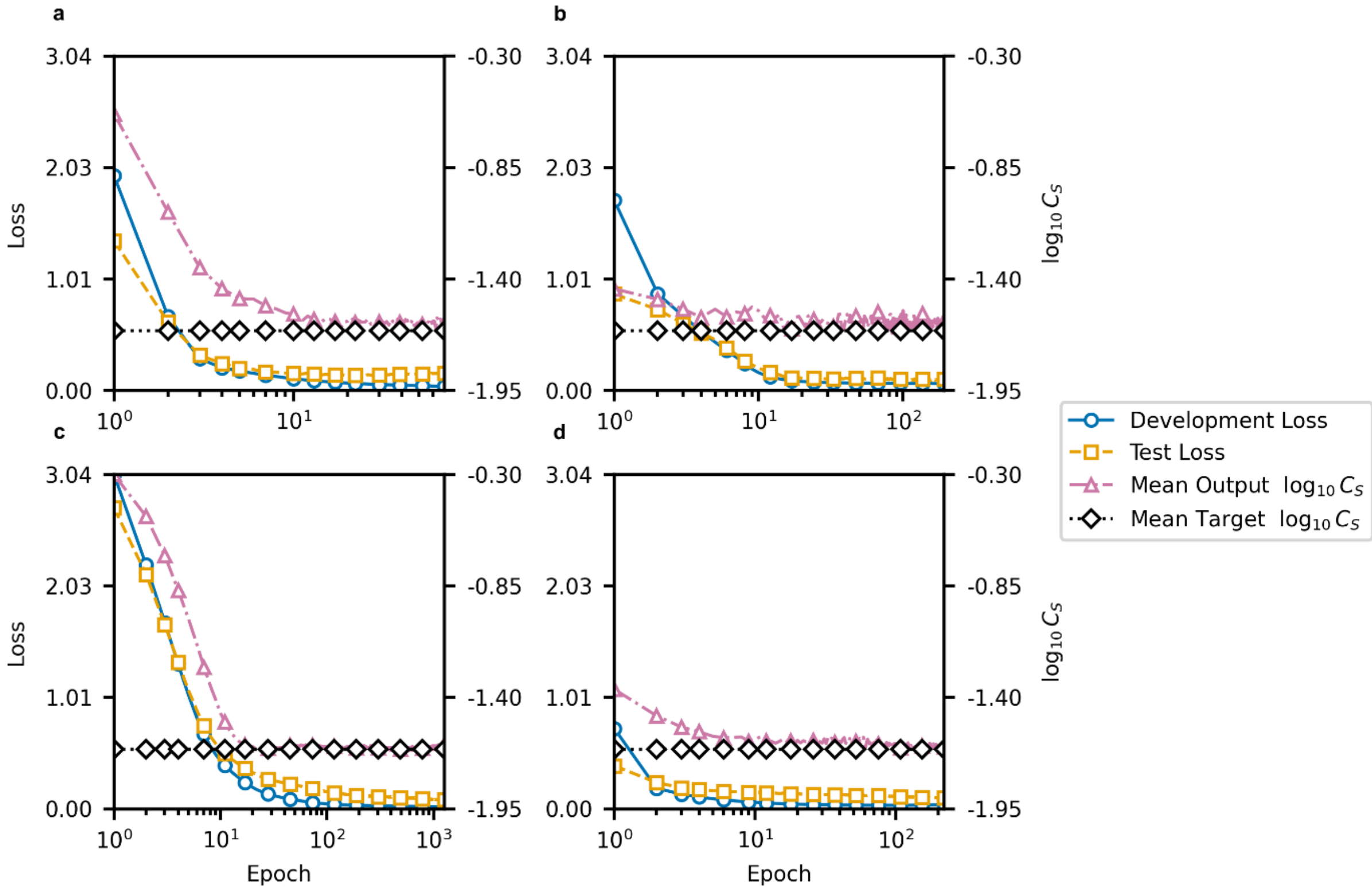

Supplementary Fig. 45. Training dynamics of the neural-network surrogates for substrate concentration $\log_{10} C_S$. Development and test mean squared error (**MSE**; left axis) and mean test-set

prediction (right axis) as functions of training epoch. The epoch axis is logarithmic. The black diamond line indicates the mean target value. **a**, Standard NN. **b**, Modified-Attention NN. **c**, Attention NN. **d**, Focused-Attention NN.

Supplementary Methods 7. Data subsampling and distributional-fidelity assessment

To reduce the computational cost of hyperparameter optimization and model fitting, random row-level subsamples were drawn from the development dataset after the simulation-level development/test split had been fixed. Because CFD mesh elements differ in physical volume, distributional fidelity was assessed using the volume-weighted kernel density estimation (KDE) procedure defined in equation (17) in the main Methods rather than unweighted row counts. Across the predictor variables and target-related quantities, the marginal distributions obtained using the investigated subsampling fractions closely followed those of the complete development dataset (Supplementary Fig. 46), supporting the use of reduced data fractions for computationally intensive model-development steps.

The largest deviations occurred for the smallest subsamples, particularly in rare distribution tails and variables constrained by reactor geometry, such as axial coordinates and liquid height. These differences are consistent with the lower probability of retaining rare or extreme

observations when increasingly small random fractions are sampled and motivated the use of larger data fractions where greater distributional fidelity was required for model fitting.

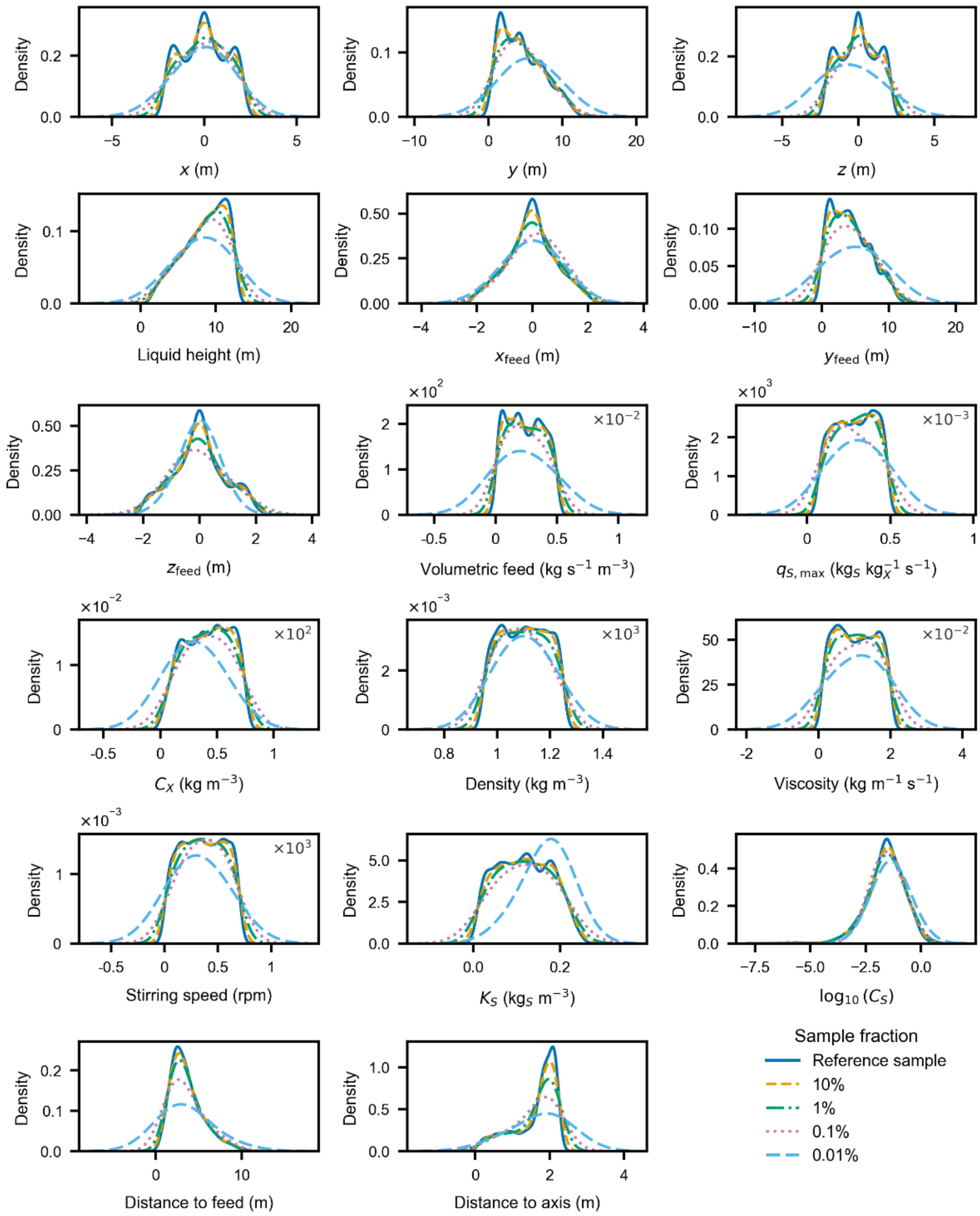

Supplementary Fig. 46. Distributional fidelity of data subsampling used for model development. Volume-weighted kernel density estimates (KDEs) of the predictor variables, including operating conditions and local spatial descriptors, and target-related quantities for the complete development dataset and progressively smaller random subsamples. KDEs are weighted by CFD mesh-element volume according to equation (17) in the main Methods.

Supplementary Note 1. Statistical foundations for model selection and generalization assessment: "From analytic criteria to empirical validation: Classical statistics vs. Modern machine learning"

Concurrently, significant advances in artificial intelligence and machine learning (AI/ML) [13–15] have expanded the spectrum of data-analytic tools far beyond the remit of classical statistics. AI denotes any computational artifact that imitates aspects of cognition, ML is the AI subfield that infers patterns from data, and deep learning encompasses those machine learning models based on multilayer neural networks [13]. Finally, classical statistics supplies the probabilistic grammar for modeling noise and hypothesis testing [16]. The rigor and clarity of classical statistics provide a foundation that, when combined with modern computational power, allows raw data to be processed by intelligent algorithms. Ordinary least-squares (OLS) regression lies at this intersection, simultaneously serving as a rigorous statistical estimator and as one of the earliest machine-learning algorithms, whose foundational principles and error metrics underpin today's computationally intensive AI/ML methods.

At the heart of every data-analytic workflow lies a model, formalized as a parameterized mapping:

$$M_\theta : \mathcal{X} \longrightarrow \mathcal{Y}, \tag{6}$$

where $\mathcal{X}$ denotes the input space (the domain of predictors or features) and $\mathcal{Y}$ represents the output space (the domain of responses, targets, or inferred latent structures). The parameter vector $\theta \in \Theta \subseteq \mathbb{R}^p$ comprises numerical quantities that determine the specific instance of the mapping M_θ. Here, $\mathbb{R}^p$ denotes the ambient Euclidean space, and the parameter space $\Theta \subseteq \mathbb{R}^p$ encodes constraints or structures imposed upon parameters—such as non-negativity, sparsity, or simplex conditions—to reflect prior knowledge or control model complexity.

Within this framework, learning, or model training, denotes the process of estimating the parameter vector θ that best aligns the chosen model M_θ with the information encoded in a dataset. Formally, learning is cast as minimizing an objective (loss) function $J(\theta)$ to obtain the optimal parameter vector $\hat{\theta}$:

$$\hat{\theta} = \arg\min_{\theta \in \Theta} J(\theta), \tag{7}$$

where the choice of objective function depends fundamentally on the learning paradigm (supervised or unsupervised) and the nature of available data. Hyperparameters control the behavior of the learning algorithm rather than representing degrees of freedom of the model itself. Unlike the entries of θ, which are updated directly from data during training, hyperparameters (e.g. architecture, regularization strengths, optimizer settings) are fixed for a given training run and are usually chosen by the practitioner or by a separate hyperparameter search procedure on a validation set. It is the interplay between these hyperparameters and the learned parameters that sets the bias–variance trade-off, governs convergence and computational cost, and ultimately determines how well the model generalizes to unseen data.

Classical statistics

Classical (frequentist) statistics is fundamentally grounded in likelihood theory, which provides a rigorous and analytical framework for parameter estimation and model evaluation. Central to likelihood theory is the concept of the likelihood function, defined as the joint probability (or probability density) of observing a specific dataset given a particular statistical model and chosen parameter values [17]. Formally, given a set of observations $\{(x_i, y_i)\}_{i=1}^{n}$, its likelihood function indicates how plausible different parameter values (θ) are, based on the observed data, and is expressed as:

$$L(\theta) = p(y_1, \dots, y_n | x_1, \dots, x_n; \theta)$$

(8)

Assuming (conditional) independence across observations given their covariates, this joint probability factorizes into the product of individual probabilities (or probability densities). As dealing directly with products of probabilities can become numerically unstable and analytically cumbersome, especially for large datasets, it is common practice to convert this product into a sum by taking the natural logarithm, yielding the log-likelihood function:

$$L(\theta) = \prod_{i=1}^{n} p(y_i | x_i; \theta) \longrightarrow \log L(\theta) = \sum_{i=1}^{n} \log p(y_i | x_i; \theta)$$

(9)

The goal of frequentist statistics is estimating the set of parameters that maximize the likelihood function (or the log-likelihood) given the observed data [17].

Classical statistical methods employ probabilistic models (e.g., linear regression, mixed models) whose likelihood functions can be optimized. By setting the gradient of the log-likelihood to zero or via numerical optimization, explicit solutions (maximum likelihood estimators) can often be found. The probabilistic grounding of these statistical models facilitates the estimation of model parameters and model-evaluation metrics if the model hypotheses are reasonable reflections of the actual system behavior. Model evaluation metrics, such as the Akaike Information Criterion (AIC) [18] or Bayesian information criterion (BIC) [19], incorporate the log-likelihood and the number of model parameters to quantify the balance between model fit and complexity.

As long as the assumed probabilistic model plausibly approximates the data-generating process, it yields principled measures of fit and parameter uncertainty (e.g., standard errors, confidence intervals, likelihood criteria) that support significance assessment and help guard against overfitting.

For instance, the multiple linear regression (MLR) model postulates a linear mean relationship with Gaussian noise,

$$y_i = \beta_0 + \sum_{j=1}^{p} \beta_j x_{ij} + \varepsilon_i, \quad \varepsilon_i \sim \mathcal{N}(0, \sigma^2),$$

(10)

and estimates the coefficients by ordinary least squares (OLS) [20],

$$\hat{\beta} = \arg\min_{\beta} \sum_{i=1}^{n} \left(y_i - \beta_0 - \sum_{j=1}^{p} \beta_j x_{ij} \right)^2 = (\mathbf{X}^\top \mathbf{X})^{-1} \mathbf{X}^\top y, \tag{11}$$

assuming $\mathbf{X}$ has full column rank. Building on this baseline, analysts can:

- add polynomial terms to $\mathbf{x}$ to capture curvature while staying linear in the parameters [21];
- shrink coefficients with Ridge (ℓ_2) or Lasso (ℓ_1) penalties, or blend the two in Elastic Net, tuning two hyperparameters (overall penalty and mixing) [22–24];
- damp the influence of outliers via the Huber loss [25], which is quadratic for small residuals and linear for large ones, retaining efficiency while gaining robustness.

All of these variants remain convex in the coefficients, so their optimization is feasible and their fitted log-likelihoods feed directly into information criteria.

Because the OLS loss is quadratic and convex, the normal equations yield a closed-form maximum-likelihood estimator.

ML models

In contrast to classical statistical models with explicit likelihoods, many modern ML models (e.g., neural networks and tree ensembles) are trained in a purely discriminative manner without specifying a parametric data-generating model; consequently, closed-form likelihoods and classical significance tests are generally unavailable. Instead, training proceeds by minimizing an empirical objective (loss) function J_θ, which can take different losses for different applications. Parameter updates that minimize J_θ are computed numerically, e.g., via gradient descent [26]:

$$\theta_{t+1} = \theta_t - \alpha \nabla_\theta J(x, y, \theta), \tag{12}$$

where $\alpha > 0$ is the learning rate, which limits the size of the steps toward function minimization. A fully stochastic variant, stochastic gradient descent (SGD), replaces the full-batch gradient with a mini-batch gradient computed on a randomly chosen subset $\mathcal{B}_t$ of the training indices [27]:

$$\theta_{t+1} = \theta_t - \alpha \nabla_\theta J_{\mathcal{B}_t}(\theta) \tag{13}$$

where $\mathcal{B}_t$ is resampled at each step. Adaptive optimizers such as Adam further accelerate convergence by normalizing these updates with running moment estimates [28].

The lack of an explicit generative model precludes likelihood-based inference (e.g., standard errors, p-values, $\mathrm{AIC/BIC}$), so model assessment relies on the empirical evaluation of generalization (the ability to predict unseen data). The data are partitioned into a training set (drives parameter updates), a validation set (monitors overfitting, tunes hyperparameters and triggers early stopping), and a test set (held back for a single, final evaluation of generalization error). When n is small, k-fold cross-validation (CV) rotates the validation role through the data; its loss estimator is:

$$\hat{J}_{\text{CV}} = \frac{1}{k}\sum_{j=1}^{k} J^{(j)}, \tag{14}$$

where $J^{(j)}$ is the loss on fold j. Hyperparameter searches—grid, random or Bayesian—wrap these training loops, compounding the computational cost and motivating the widespread use of GPUs, TPUs, and distributed training infrastructure in modern ML pipelines.

Depending on the availability of labels, ML training can be divided into supervised and unsupervised learning.

Supervised learning

Supervised learning problems typically fall into two families. (i) In classification, the target is categorical and the goal is to assign well-calibrated class probabilities and make reliable decisions between discrete labels. (ii) In regression, the target is continuous and the aim is to predict numeric responses with minimal error and well-characterized uncertainty. In what follows we focus on regression, as the outputs used in this study are continuous-valued process variables (e.g., substrate concentration or velocity magnitudes).

In supervised learning, the dataset ($\mathcal{D}$) consists of observed input-output pairs $\mathcal{D} = \{(x_i, y_i)\}_{i=1}^{n}$, providing explicit labels or targets $y_i \in \mathcal{Y}$ for each input $x_i \in \mathcal{X}$. The objective function for supervised learning, often referred to as empirical risk, is expressed as the average of a loss function $\mathcal{L}$:

$$J(\theta)_{\text{supervised}} = \frac{1}{n}\sum_{i=1}^{n} \mathcal{L}\big(y_i, M_\theta(x_i)\big) + \Omega(\theta; \lambda), \tag{15}$$

where the loss function $\mathcal{L} : \mathcal{Y} \times \mathcal{Y} \to \mathbb{R}_{\geq 0}$ quantifies discrepancies between predicted responses $M_\theta(x_i)$ and observed responses y_i. Examples of supervised learning losses include the mean-squared error (MSE, $\|y_i - M_\theta(x_i)\|_2^2$), absolute error (MAE, $|y_i - M_\theta(x_i)|$), or cross-entropy for classification tasks [13]. These losses encode different assumptions about the underlying data distribution or the desired predictive behavior. The term $\Omega(\theta; \lambda)$ is an optional regularizer, introduced to penalize complexity or enforce desired structural properties on the parameters θ. It prevents overfitting by restricting model flexibility, typically controlled by the scalar hyperparameter $\lambda > 0$, which determines the strength of regularization.

Decision trees

Beyond the classical loss-regularizer formulations, supervised learning embraces model classes whose complexity and expressive power far exceed that of linear predictors. The simplest nonlinear family is the decision tree (DT), which recursively partitions the predictor space by selecting a feature and split value that maximizes an impurity reduction, measured, for example, by the residual sum of squares in regression or by the Gini impurity in classification [29]. Although trees are easy to interpret, their stepwise, region-constant predictions make them high-variance estimators prone to overfitting.

Random forest

The bootstrap is a resampling scheme in which one draws samples of size n from the training set with replacement, thereby producing multiple "bootstrap samples" that contain duplicates [30]. Bagging (bootstrap aggregating) leverages this mechanism by training an identical base learner (e.g., a tree) on many such bootstrap samples and then aggregating their predictions—averaging for regression, majority vote for classification—to reduce variance and stabilize performance [31]. Because each bootstrap sample leaves out a subset of training points, one can form out-of-bag (OOB) predictions for any training observation by averaging only the models that did not include it; the OOB error provides an internal estimate of generalization performance without a separate validation set and is commonly used for model selection.

Random forests extend bagging by injecting additional randomness during tree construction: at every split, only a random subset of features is considered. This feature subsampling decorrelates the trees, making the aggregated predictor more effective than plain bagging when strong predictors dominate [32]. In practice, random forests train many bootstrapped trees with feature subsampling at each split and aggregate them as in bagging; hyperparameters such as the number of trees, number of features tried at each split, maximum depth, and minimum samples per leaf are typically tuned using OOB error or CV.

Gradient boosting

A more aggressive strategy, boosting, fits trees sequentially so that each new tree focuses on the residual errors of the current ensemble [33]. Modern implementations such as XGBoost and LightGBM cast boosting as steepest descent in function space, iteratively minimizing a specified loss while controlling complexity via learning rate, tree depth, and regularization [34,35]. These methods often deliver state-of-the-art accuracy on structured data with comparatively few hyperparameters to tune.

Kernel-based methods

Another classical nonlinear approach arises from kernel methods, which let us handle complex patterns by working in a higher-dimensional space. Instead of transforming the input x into that space directly, we use a kernel function:

$$K(x, x') = \langle \phi(x), \phi(x') \rangle, \tag{16}$$

which computes how similar two points are as if they had been mapped into that space—without doing the mapping. This shortcut, called the kernel trick, allows models to stay efficient while learning nonlinear patterns. It is used in support vector regression (SVR), where the goal is to find a simple function that fits the data well, with only a few data points—support vectors—influencing the final model [36].

Neural networks

When the target relationship is too intricate for kernel methods or tree ensembles, practitioners turn to neural networks (NNs). The lineage begins with the single-neuron perceptron [37]. A perceptron performs a forward pass: it takes a linear combination of its inputs and applies a step-like activation to produce an output. Extending this idea, Rumelhart et al. [38] stacked many such units in parallel and in series to obtain the multilayer perceptron (MLP) and, most importantly,

introduced backpropagation—an algorithm that computes gradients of the loss with respect to every weight by the chain rule, allowing all layers to learn simultaneously.

Each neuron, the fundamental computational unit of NNs, first applies a linear transformation to its input (weighted sum plus bias) and then a non-linear activation to the result—such as the rectified linear unit $\mathrm{ReLU}(z) = \max(0, z)$, the logistic sigmoid, or the hyperbolic tangent. In a fully connected feed-forward network with $\ell = 1, \dots, L$ layers, the forward pass (prediction from input) is:

$$h^{(0)} = x, \quad h^{(\ell)} = \sigma\big(\mathbf{W}^{(\ell)} h^{(\ell-1)} + \mathbf{b}^{(\ell)}\big); \quad \ell = 1, \dots, L; \quad \hat{y} = g\big(h^{(L)}\big), \tag{17}$$

where $\{\mathbf{W}^{(\ell)}, \mathbf{b}^{(\ell)}\}$ are layer-specific weights and biases, σ is the hidden-layer activation, and g is the output activation (linear for regression, softmax for multiclass classification). Backpropagation propagates the loss gradient through this composite map, yielding derivatives for every weight, which are then updated by stochastic-gradient methods, enabling modern deep networks to fit highly nonlinear functions.

Other specialized neural-network architectures capture distinct data structures more efficiently. Convolutional Neural Networks (CNNs) slide learned filters over grid-like inputs (e.g., images), drastically cutting parameter count while detecting local patterns such as edges or textures [39]. For sequential data, such as language, Recurrent Neural Networks (RNNs) weave information through time by feeding each output back as input at the next step, granting them some form of memory for sequence modeling [38]. To overcome RNNs' vanishing-gradient issues, gated variants like long short-term memory (LSTM) [40] or gated recurrent unit (GRU) [41] introduce learnable gates that regulate how much past information is retained or forgotten.

Transformers

The predominant architecture for sequence data is now the Transformer, whose hallmark is self-attention [15]. Given matrices of queries $\mathbf{Q}$, keys $\mathbf{K}$, and values $\mathbf{V}$, produced by learned linear projections of the input tokens (discrete splits of the input data), one attention head computes:

$$\mathrm{Attention}(\mathbf{Q}, \mathbf{K}, \mathbf{V}) = \mathrm{softmax}\left(\frac{\mathbf{Q}\mathbf{K}^{\top}}{\sqrt{d_k}}\right)\mathbf{V}, \tag{18}$$

where d_k is the dimensionality of the key vectors used in the scaling factor. The factor $\sqrt{d_k}$ prevents the dot products in $\mathbf{Q}\mathbf{K}^{\top}$ from growing too large in high dimensions. This formulation allows each token to assimilate information from every other token in the sequence with the attention weights controlling the strength of these interactions.

In essence, supervised learning today covers everything from simple, transparent linear regressions—augmented with penalties to control complexity—to powerful, nonlinear methods like tree ensembles, kernel machines, and a spectrum of neural network designs (CNNs for images; RNNs and transformers for sequences). All these methods share a common goal—minimizing empirical risk—but differ wildly in how flexible their models are and how much computational effort is needed to find good solutions.

Generative artificial intelligence

Generative artificial intelligence (AI) refers to models that learn the underlying patterns of data and can subsequently generate new, realistic samples that resemble the original distribution. Unlike predictive models, which estimate outcomes for given inputs, generative models create entirely new instances, extending beyond the conditions observed during training [13]. In bioprocessing, such models can augment sparse datasets, fill sensor gaps, propose plausible operating scenarios, or serve as fast surrogates for computationally expensive simulations—provided the generated outputs are constrained and validated for physical plausibility.

Unsupervised learning

In contrast to supervised learning, unsupervised learning employs datasets that contain only inputs $\mathcal{D} = \{x_i\}_{i=1}^{n} \subseteq \mathcal{X}$, without corresponding explicit labels. Here, the objective is not directly predictive accuracy against observed targets, but rather the detection or encoding of meaningful patterns or latent structures within the data itself. Thus, the objective function typically takes the form of a structural cost $c : \mathcal{X} \times \mathcal{H} \to \mathbb{R}_{\geq 0}$, such as reconstruction error (PCA, autoencoders), cluster compactness (clustering), or negative log-likelihood (Gaussian mixture models) under a latent density model (with $M_\theta : \mathcal{X} \to \mathcal{H}$ a representation or generative model):

$$J_{\text{unsup}}(\theta) = \frac{1}{n}\sum_{i=1}^{n} c\big(x_i, M_\theta(x_i)\big) + \Omega(\theta; \lambda) \tag{19}$$

Typical unsupervised tasks fall into two broad families depending on the objective: structure discovery (clustering) and representation learning (dimensionality reduction).

Structure discovery (clustering)[42–44]

At one end of the spectrum, clustering algorithms such as k-means iteratively assign each point to its closest prototype, refining those prototypes until the within-cluster scatter (inertia) can no longer be reduced [42–44]. A probabilistic reinterpretation of the same idea leads to Gaussian mixture models (GMMs), which view the data as arising from several overlapping normal distributions; the expectation-maximization (EM) algorithm alternates between estimating the probability that each point belongs to each component and re-estimating the component parameters, gradually revealing coherent latent subpopulations [45].

Representation learning (dimensionality reduction)

At the other end lie dimensionality reduction techniques. Classical principal component analysis (PCA) rotates the predictor coordinate system so that the first few axes capture the bulk of the variance, yielding a compact orthogonal basis that often exposes hidden linear structure [46,47]. When relationships are curved rather than linear, neural autoencoders take over: an encoder network compresses each input into a low-dimensional code, and a decoder tries to reconstruct the original signal from that code [38,48]. Successful reconstruction forces the code to act as a distilled summary, much like a nonlinear generalization of PCA.

Between these extremes, a variety of algorithms—spectral clustering, manifold learning, density-based methods—pursue the same unsupervised goal: to minimize some structural cost so that what remains in the model parameters is an intelligible portrait of the data's underlying organization, all achieved without ever seeing a label.

In the end, supervised and unsupervised learning share the same mathematical backbone: both define an objective (loss) on the observed data as a function of the model parameters, and then minimize it. What distinguishes them is whether that objective is anchored to external labels, as in supervised learning, or to patterns that the data reveal on their own, as in the unsupervised case. Modern ML methods do not necessarily require explicit generative probabilistic assumptions and typically rely on numerical optimization rather than closed-form derivations, marking a departure from classical statistics, which often yields analytic solutions based on probabilistic modeling and hypothesis testing. Where frequentist classical methods lean on likelihood theory, information criteria, and test statistics, modern machine learning turns instead to flexible function approximators, empirical risk minimization, and validation through resampling. The shift is not in the goal—learning from data—but in assumptions and tools: from probabilistically motivated, analytically tractable objectives to data-driven, compute-intensive algorithms with fewer explicit distributional assumptions. This transition has been greatly accelerated by the rise of open-source machine-learning libraries such as scikit-learn [49], TensorFlow [50], PyTorch [12], and XGBoost [34], which provide unified, efficient, and accessible implementations of both classical and cutting-edge models, making powerful methods available to researchers and practitioners across disciplines.

Supplementary References

1. Rushton, J. H., Costich, E. W. & Everett, H. J. Power characteristics of mixing impellers. Part I. *Chem. Eng. Prog.* **46**, 395–404 (1950).
2. Rushton, J. H., Costich, E. W. & Everett, H. J. Power characteristics of mixing impellers. Part II. *Chem. Eng. Prog.* **46**, 467–476 (1950).
3. Doran, P. M., Morrissey, K. & Carlson, R. P. *Bioprocess Engineering Principles, Third Edition* (Academic Press, 2025). doi:10.1016/C2017-0-01824-4.
4. Magelli, F., Montante, G., Pinelli, D. & Paglianti, A. Mixing time in high aspect ratio vessels stirred with multiple impellers. *Chem. Eng. Sci.* **101**, 712–720 (2013).
5. Armenante, P. M. & Chang, G.-M. Power Consumption in Agitated Vessels Provided with Multiple-Disk Turbines. *Ind. Eng. Chem. Res.* **37**, 284–291 (1998).
6. Haringa, C., Vandewijer, R. & Mudde, R. F. Inter-compartment interaction in multi-impeller mixing: Part I. Experiments and multiple reference frame CFD. *Chemical Engineering Research and Design* **136**, 870–885 (2018).
7. Haringa, C., Vandewijer, R. & Mudde, R. F. Inter-compartment interaction in multi-impeller mixing. Part II. Experiments, sliding mesh and large Eddy simulations. *Chemical Engineering Research and Design* **136**, 886–899 (2018).
8. Günyol, Ö. & Mudde, R. F. Computational Study of Hydrodynamics of a Standard Stirred Tank Reactor and a Large-Scale Multi-Impeller Fermenter. *Int. J. Multiscale Comput. Eng.* **7**, 559–576 (2009).
9. Bach, C. *et al.* Evaluation of mixing and mass transfer in a stirred pilot scale bioreactor utilizing CFD. *Chem. Eng. Sci.* **171**, 19–26 (2017).
10. Bergstra, J., Bardenet, R., Bengio, Y. & Kégl, B. Algorithms for Hyper-Parameter Optimization. *Adv. Neural Inf. Process. Syst.* **24**, (2011).
11. Akiba, T., Sano, S., Yanase, T., Ohta, T. & Koyama, M. Optuna: A Next-generation Hyperparameter Optimization Framework. *Proceedings of the ACM SIGKDD International Conference on Knowledge Discovery and Data Mining* 2623–2631 (2019) doi:10.1145/3292500.3330701.
12. Paszke, A. *et al.* PyTorch: An Imperative Style, High-Performance Deep Learning Library. *Adv. Neural Inf. Process. Syst.* **32**, (2019).
13. Bishop, C. M. *Pattern Recognition and Machine Learning*. (Springer, New York, 2006).
14. Gurney, K. *An Introduction to Neural Networks*. (CRC Press, London, UK, 1997).
15. Vaswani, A. *et al.* Attention is All you Need. *Adv. Neural Inf. Process. Syst.* **30**, (2017).
16. Hastie, T., Tibshirani, R. & Friedman, J. H. *The Elements of Statistical Learning: Data Mining, Inference, and Prediction*. (Springer Series in Statistics, New York, NY, 2009). doi:10.1007/978-0-387-84858-7.
17. Breiman, L. Statistical modeling: The two cultures (with comments and a rejoinder by the author). *Statistical science* **16**, 199–231 (2001).

18. Akaike, H. Factor Analysis and AIC. *Psychometrika* **52**, 317–332 (1987).

19. Schwarz, G. Estimating the Dimension of a Model. *The Annals of Statistics* **6**, 461–464 (1978).

20. Kutner, M. H., Nachtsheim, C. J., Neter, J. & Li, W. *Applied Linear Statistical Models*. (McGraw-Hill/Irwin, Boston, 2005).

21. Theil, H. A Rank-Invariant Method of Linear and Polynomial Regression Analysis. in *Henri Theil's Contributions to Economics and Econometrics: Econometric Theory and Methodology* (eds. Raj, B. & Koerts, J.) 345–381 (Springer Netherlands, Dordrecht, 1992). doi:10.1007/978-94-011-2546-8_20.

22. Tibshirani, R. Regression Shrinkage and Selection Via the Lasso. *J. R. Stat. Soc. Series B Stat. Methodol.* **58**, 267–288 (1996).

23. Hoerl, A. E. & Kennard, R. W. Ridge Regression: Biased Estimation for Nonorthogonal Problems. *Technometrics* **12**, 55–67 (1970).

24. Zou, H. & Hastie, T. Regularization and Variable Selection Via the Elastic Net. *J. R. Stat. Soc. Series B Stat. Methodol.* **67**, 301–320 (2005).

25. Huber, P. J. Robust Estimation of a Location Parameter. *The Annals of Mathematical Statistics* **35**, 73–101 (1964).

26. Boyd, S. & Vandenberghe, L. *Convex Optimization*. (Cambridge University Press, Cambridge, 2004). doi:10.1017/CBO9780511804441.

27. Robbins, H. & Monro, S. A stochastic approximation method. *The annals of mathematical statistics* **22**, 400–407 (1951).

28. Kingma, D. P. & Ba, J. L. Adam: A Method for Stochastic Optimization. *3rd International Conference on Learning Representations, ICLR 2015 - Conference Track Proceedings* (2015).

29. Quinlan, J. R. Induction of decision trees. *Mach. Learn.* **1**, 81–106 (1986).

30. Efron, B. Bootstrap Methods: Another Look at the Jackknife. in *Breakthroughs in Statistics: Methodology and Distribution* (eds. Kotz, S. & Johnson, N. L.) 569–593 (Springer New York, New York, NY, 1992). doi:10.1007/978-1-4612-4380-9_41.

31. Breiman, L. Bagging predictors. *Mach. Learn.* **24**, 123–140 (1996).

32. Breiman, L. Random forests. *Mach. Learn.* **45**, 5–32 (2001).

33. Friedman, J. H. Greedy function approximation: A gradient boosting machine. *Ann. Stat.* **29**, 1189–1232 (2001).

34. Chen, T. & Guestrin, C. XGBoost: A scalable tree boosting system. *Proceedings of the 22nd ACM SIGKDD International Conference on Knowledge Discovery and Data Mining* 785–794 (2016) doi:10.1145/2939672.2939785.

35. Ke, G. *et al.* LightGBM: A Highly Efficient Gradient Boosting Decision Tree. *Adv. Neural Inf. Process. Syst.* **30**, (2017).

36. Drucker, H., Burges, C. J., Kaufman, L., Smola, A. & Vapnik, V. Support Vector Regression Machines. *Adv. Neural Inf. Process. Syst.* **9**, (1996).

37. Rosenblatt, F. The perceptron: A probabilistic model for information storage and organization in the brain. *Psychol. Rev.* **65**, 386–408 (1958).

38. Rumelhart, D. E., Hinton, G. E. & Williams, R. J. Learning representations by back-propagating errors. *Nature* **323**, 533–536 (1986).

39. LeCun, Y. *et al.* Backpropagation Applied to Handwritten Zip Code Recognition. *Neural Comput.* **1**, 541–551 (1989).

40. Hochreiter, S. & Schmidhuber, J. Long Short-Term Memory. *Neural Comput.* **9**, 1735–1780 (1997).

41. Cho, K. *et al.* Learning Phrase Representations using RNN Encoder-Decoder for Statistical Machine Translation. *EMNLP 2014 - 2014 Conference on Empirical Methods in Natural Language Processing, Proceedings of the Conference* 1724–1734 (2014) doi:10.3115/v1/d14-1179.

42. Steinhaus, H. Sur la division des corps matériels en parties. *Bull. Acad. Polon. Sci* **1**, 801 (1956).

43. MacQueen, J. B. Some Methods for Classification and Analysis of Multivariate Observations. in *Proceedings of the Fifth Berkeley Symposium on Mathematical Statistics and Probability* (eds. Le Cam, L. M. & Neyman, J.) 281–297 (University of California Press, Berkeley, CA, USA, 1967).

44. Lloyd, S. P. Least Squares Quantization in PCM. *IEEE Trans. Inf. Theory* **28**, 129–137 (1982).

45. Dempster, A. P., Laird, N. M. & Rubin, D. B. Maximum Likelihood from Incomplete Data Via the EM Algorithm. *J. R. Stat. Soc. Series B Stat. Methodol.* **39**, 1–22 (1977).

46. Pearson, K. LIII. On lines and planes of closest fit to systems of points in space. *The London, Edinburgh, and Dublin Philosophical Magazine and Journal of Science* **2**, 559–572 (1901).

47. Hotelling, H. Analysis of a complex of statistical variables into principal components. *J. Educ. Psychol.* **24**, 417–441 (1933).

48. Baldi, P. & Hornik, K. Neural networks and principal component analysis: Learning from examples without local minima. *Neural Networks* **2**, 53–58 (1989).

49. Pedregosa, F. *et al.* Scikit-learn: Machine Learning in Python. *Journal of Machine Learning Research* **12**, 2825–2830 (2011).

50. Abadi, M. *et al.* TensorFlow: A system for large-scale machine learning. *Proceedings of the 12th USENIX Symposium on Operating Systems Design and Implementation, OSDI 2016* 265–283 (2016).
