# Supplementary material for: Machine-learning surrogates enable rapid prediction of 3-D substrate gradients in industrial bioreactors
Source: Nat Commun. 2026 Oct 15;17:11636. doi: 10.1038/s41467-026-78273-7 (PMC13754804; doi:10.1038/s41467-026-78273-7)
Supplement: Supplementary file 2 [file 41467_2026_78273_MOESM2_ESM.pdf]

Description of Additional Supplementary Files

Supplementary Data 1. Global input variables and operating conditions for the CFD simulation campaign. Case-level table containing the global operating variables, feeding-point coordinates, fluid properties and biokinetic parameters for all 760 CFD simulations used in this study.

Supplementary Software 1. Figure-generation scripts. Scripts used to reproduce the main and Supplementary Figures from the numerical results supplied in the Source Data file. The package contains plotting and data-processing routines required to regenerate the published graphical outputs and does not contain the restricted CFD-generation, surrogate-training or trained-model software.
