# Supplementary material for: Machine-learning surrogates enable rapid prediction of 3-D substrate gradients in industrial bioreactors
Source: Nat Commun. 2026 Oct 15;17:11636. doi: 10.1038/s41467-026-78273-7 (PMC13754804; doi:10.1038/s41467-026-78273-7)
Supplement: Supplementary file 3 [file 41467_2026_78273_MOESM5_ESM.pdf]

nature portfolio

Peer Review File

Machine-learning surrogates enable rapid prediction of 3-D substrate gradients in industrial bioreactors

Corresponding Author: Dr Víctor Puig-I-Laborda

This file contains all reviewer reports in order by version, followed by all author rebuttals in order by version.

Version 0:

Reviewer comments:

Reviewer #1

(Remarks to the Author)
This manuscript comprehensively evaluated CFD surrogate models using different machine learning algorithms. The evaluation work was extensive, covering a wide range of machine learning algorithms and fluid flow conditions. The work presents a good modeling practice but needs greater emphasis on its specific usefulness in real bioreactor applications. Many of the model parameters, such as density and viscosity, were held constant, which do not reflect real bioreactor conditions. The authors need to discuss these limitations and explain how specific applications may benefit from this work for real-life adoption.
In ML modeling, the CFD modeling parameters for the training and validation datasets need to be specified to allow readers to reproduce the proposed work. The grid-independent study and CFD model validation are missing.

Reviewer #2

(Remarks to the Author)
The paper by Víctor Puig-I-Laborda develops a methodology for automatic development of CFD surrogate models to describe 3D substrate gradients in large stirred-tank bioreactors. A key innovation of the study is the ability to simulate these 3D substrate gradients within milliseconds, representing a significant improvement in computational efficiency compared to typical CFD simulations. As the authors rightly note, integrating CFD with biological reaction mechanisms and their associated kinetics remains rare in the literature. In this regard, the work constitutes a clear advancement over the current state of the art.

In terms of practical applicability, this study opens the possibility for model-basted process monitoring and control in real-time that explicitly account for substrate heterogeneities in large-scale vessels. This represents a highly relevant area and an essential direction for future research.

Different machine learning (ML) approaches were systematically benchmarked. The best-performing model was a standard neural network (NN) incorporating an attention mechanism. However, the results are not readily generalizable to other problem domains. More importantly, the strength of the study lies in its methodology, which is well-reasoned and correctly implemented, constituting the paper’s primary contribution. The discussion and conclusions are well supported by the results.

I have however some doubts in some parts of the paper and I would like some additional clarifications.

Q1: Physics-informed neural networks were applied several times for CFD including 3D flows.
Cai, S., Mao, Z., Wang, Z. et al. Physics-informed neural networks (PINNs) for fluid mechanics: a review. Acta Mech. Sin. 37, 1727–1738 (2021). https://doi.org/10.1007/s10409-021-01148-1
In fact, the first paper that introduced PINNs addressed a CFD case study.
M. Raissi, P. Perdikaris, G.E. Karniadakis, Physics-informed neural networks: A deep learning framework for solving forward and inverse problems involving nonlinear partial differential equations, Journal of Computational Physics, Volume 378, 2019, Pages 686-707, https://doi.org/10.1016/j.jcp.2018.10.045.
Moreover, PINNs were applied for CFD of stirred tank bioreactors, e.g.:

Horiguchi I, Shima K, Okano Y. Physics-informed neural networks (PINNs) for high-resolutional prediction of shear stress on cells in suspension culture. AIChE J. 2025;71(7):e18853. doi:10.1002/aic.18853
In the case of PINNs, the neural network can be trained directly on the Navier Stokes equations. It is a different concept with the same purpose. The main section of the paper would benefit from a reference to the PINN methodology for CFD. Even if it has never been applied for 3D substrate gradients. The discussion would also benefit with a reference to potential advantages/disadvantages of the surrogate approach in relation to the PINN approach.

Q2: Figure 1 organization is very clear. I would however recommend to replace the box “test on Dev. Set” by “Validate on Validation Set”. I am assuming that any decision on hyperparameters was taken on the basis of the validation error. Could the authors please confirm?

Q3: I imagine a very significant computational cost to run this pipeline (the 760 CFD simulations + ML surrogate models development and benchmarking). This information is neglected in the paper. Could the Authors add this information to the paper? Since the results are not generalizable to different domains, it is important to known the cost of repeating the pipeline for a different domain.

Q4: Regarding the data generation step, the 760 CFD simulations assume fed-batch operation and are run dynamically until a pseudo-steady state is reached. Even if the Monod substrate uptake cannot the generalized to all cases, it is very interesting that qsmax and KS are global input variables. This allows the surrogate model to infer a different kinetic parameters set without the need to retain it. However, I have some concerns about the biomass concentration. The biomass concentration (another global variable) is assumed to be constant over time (please confirm). This has the advantage that 3D substrate steady-state obtained by CFD does not depend on the initial condition.
dS/dt = qsmax S/(KS+S)*X – D(S -Sin) = 0 => S = f(parameters=(qsmax, KS, X, D, Sin))
Indeed, initial conditions are never inputs to the surrogate models. However, the 3D steady-state snapshot obtained in this way is not a good representation of a typical in fed-batch operation. Could this be a practical limitation? Why didn’t the authors assume a CSTR instead?

Q5: The CFD simulations assumes a standard stirred tank configuration with 4 Rushton-impellers and fixed reactor size. Could the surrogate models be applied to a different tank size while keeping the same geometry and configuration?

Q6: The standard feedforward neural network with Modified-Attention achieved the lowest test MSE (0.0054) and a test R2 of 0.91 (Figure 2). Moreover, the test MSE is comparable (even a bit lower) than the train MSE, which is an excellent result. The authors state that “the Polynomial–Lasso regressors at intermediate degree (3rd-4th order) emerged as the next best class”. However, the train loss is roughly 50% of the test loss, this is not so positive and could suggest overfitting. The closest match is the standard ANN without the attention mechanism. Are the differences significant between the NN with and without attention? Why does attention matter in this case, given the low dimension and uncorrelated inputs (Extended Figs. 3-5)?

Q7: The effect of training data size on the test loss shows a saturation. Again the NN with modified attention mechanism stands out as the best approach. The authors conclude that “beyond a few hundred additional runs, the improvements in accuracy become increasingly marginal relative to the computational cost”. The test R2 was 0.91. What about the train R2? I am assuming it was close to 0.91 (given the comparable MSE). Why does the pipeline saturate at 0.91 test R2? Could this be related to the ANN topology and/or training algorithm? Maybe underfitting?

Q8: The 10-folds CV in the supplementary information delivered a validation loss mean and dispersion coefficient, which are not given in the paper. If the authors compare the validation loss across the different ML methods (the ones with hyperparameter challenges), would the same conclusion be reached as the one in Figure 2?

Q9: Please include in the captions of Supplementary Information Figs. 12-28 that the residual analysis refers to the test data only. Was the residuals distribution better for the Development Data set?

Reviewer #3

(Remarks to the Author)
In their manuscript the authors present in detail a surrogate modelling methodology aimed at using data-driven methods to recover the three-dimensional profiles of substrate concentrations predicted by CFD simulations in the context of fermentation operations. The document is well-written and the framework proposed therein is highly relevant for the improvement of model-based analysis and optimisation of bioprocessing applications. The discussed methodology is sound and the best-performing surrogate model appears to offer satisfactory predictive power. However, in my opinion the article does not fully demonstrate the stated advantages of the prosed framework, i.e. the ability to (partially) replace and/or complement 3D CFD predictions. For these reasons, I recommend the manuscript to be published after the authors have addressed the following points.

1.It is not completely clear to what extend the surrogate models can interpolate along the explored 10D hyper-surface. The authors discuss on page 11 that the developed model can be used to screen multiple operating points without the need of conducting additional CFD simulations. For this to be realised, model predictions need to be well-behaved when some or all of the inputs are in-between the points in the grid used for training. Although the text reports similar MSEs between development and test sets for the best model, the presented residual analysis does not distinguish between the sets (I presume the plots show both). This makes it difficult to assess if the discussed patterns regarding model errors are different

for the holdout data. Furthermore, the text should show that model predictions behave correctly. For example, if qs/qs_max at a given point is expected (or observed from simulations) to change smoothly with an increasing stirring speed, surrogate model predictions for a dense array of speeds should be approximately smooth as opposed to jagged or discontinuous.

2.Similarly, it is unclear if the developed models have any extrapolation power. It is acknowledged that the presented work aims to show a proof of concept. However, the value of this work would be greatly enhanced if the authors can prove that the surrogate models can offer some degree of predictive power when one or more of the inputs are outside the intervals showed on table 1. More importantly, the article emphasises the advantages derived from fast inference times after an initial and substantial computational investment, which corresponds to the original CFD simulations in addition to model training. Consequently, the relevance of the presented approach would be significantly degraded if too many new CFD simulations are needed to allow the surrogate model to account for relatively small changes in operational and/or geometric parameters. Can the authors estimate where this threshold lies? Is it possible for any of the tested models to cope with any changes in the reactor geometry? If not, would this imply that the proposed method cannot be used to conduct geometric optimisations?

3.As discussed on page 9, prediction errors concentrate on high-shear zones and in saturated and depleted zones. These are the regions where the usage of high-resolution CFD simulations has the highest value. Please discuss further the industrial implications of this limitation. Moreover, please assess how does the presented surrogate model compare with other computationally efficient alternatives, such as tank-in-series or other possible types of compartmental models.

4.Figure 4 and the parity plots in the supplementary materials show that the best surrogate model can recover the target to a high degree. However, the residual error can climb to be around 20-30% or higher. Is this satisfactory enough for the intended industrial application? Following the previous remark, does this error level allows for the surrogate model to be more informative than, for instance, a simpler compartmental approach, which would not require the elevated computational expenses of the surrogate model?

5.The authors assert that the developed model can potentially integrate process dynamics by using a snapshot strategy. Please show an example of such predictions, preferably accompanied by the results of transient CFD simulations (or experimental data, if available).

6.How was mesh-independence ensured within the automated workflow used to generate the dataset?

7.Did the authors conduct a feature importance analysis on the non-MLR models?

8.Please provide some discussion justifying the absence of experimental validation of the CFD models. What is the expected effect of the surrogate model inheriting the assumptions of the CFD setup?

9.How do the authors expect the modelling framework to behave in the presence of non-Monod kinetics?

10.How were the sets of tunned hyperparameters chosen? How were the neural network architectures defined?

Minor comments:

11.Page 12: ‘can be emulated’
12.There are some “Error! Reference source not found’’ instances in the supplementary materials file.

Version 1:

Reviewer comments:

Reviewer #1

(Remarks to the Author)
The authors addressed the reviewer's comments.

Reviewer #2

(Remarks to the Author)
All questions raised were properly addressed and I am happy with this revised version of the manuscript.

Reviewer #3

(Remarks to the Author)
In my opinion, the authors have appropriately addressed the previous remarks made by the reviewers.

Open Access This Peer Review File is licensed under a Creative Commons Attribution 4.0 International License, which permits use, sharing, adaptation, distribution and reproduction in any medium or format, as long as you give appropriate credit to the original author(s) and the source, provide a link to the Creative Commons license, and indicate if changes were made.
In cases where reviewers are anonymous, credit should be given to 'Anonymous Referee' and the source.
The images or other third party material in this Peer Review File are included in the article's Creative Commons license, unless indicated otherwise in a credit line to the material. If material is not included in the article's Creative Commons license and your intended use is not permitted by statutory regulation or exceeds the permitted use, you will need to obtain permission directly from the copyright holder.
To view a copy of this license, visit https://creativecommons.org/licenses/by/4.0/

Answer to reviewers

Reviewer #1 (Remarks to the Author):

This manuscript comprehensively evaluated CFD surrogate models using different machine learning algorithms. The evaluation work was extensive, covering a wide range of machine learning algorithms and fluid flow conditions. The work presents a good modeling practice but needs greater emphasis on its specific usefulness in real bioreactor applications. Many of the model parameters, such as density and viscosity, were held constant, which do not reflect real bioreactor conditions. The authors need to discuss these limitations and explain how specific applications may benefit from this work for real-life adoption.

In ML modeling, the CFD modeling parameters for the training and validation datasets need to be specified to allow readers to reproduce the proposed work. The grid-independent study and CFD model validation are missing.

We thank the reviewer for the constructive assessment of our manuscript. We are pleased that the reviewer found the machine-learning evaluation extensive and recognized the modeling practice presented in the study. We also agree that the original version required a clearer discussion of practical applications, model limitations and mesh independence. We have revised the manuscript and Supplementary Information accordingly, and we address each point in more detail below.

A) Comment on density and viscosity parameters

Density and viscosity were not held fixed across the full dataset; they were included as global input variables and varied from simulation to simulation as part of the LHS design, alongside other parameters such as bio-kinetics, stirring speed, liquid height, feeding point position, feeding rate, biomass concentration... (Methods). However, within each individual CFD simulation, the liquid was treated as a Newtonian fluid with constant density and viscosity.

Current and future work focuses on adding power-law non-Newtonian behavior in the ML method. Thus, assuming constant viscosity inside the same simulation limits the scope to Newtonian fluids, but from simulation to simulation these parameters were modified.

Modified paper:

- Mostly clarified through existing Methods text: density/viscosity are listed as global inputs in **Methods, lines 452–457.**

- Limitations are discussed in **Discussion, lines 385–395**, where the surrogate is framed as inheriting CFD assumptions. Future rheology/non-Newtonian extension: **Discussion, lines 424–430**.

B) Comment on usefulness in real bioreactor applications and limitations

The present study is intended as a proof of concept showing that ML surrogates can reproduce CFD-predicted 3-D substrate gradients in millisecond-scale inference times. We do not claim that the current model is already a fully general industrial deployment tool; rather, it demonstrates a new route towards fast spatially resolved bioreactor modeling.

Within the sampled operating domain and reactor configuration, the framework can support practical bioprocess-development tasks such as rapid screening of feeding conditions, stirring speeds, biomass concentrations and kinetic regimes; identification of operating points likely to generate strong substrate heterogeneities; and generation of 3-D substrate fields for future optimization or digital-twin workflows. This is valuable because such analyses would normally require repeated CFD simulations, whereas the trained surrogate enables extensive operating-window exploration at negligible runtime cost.

Modified paper:

- Expanded mainly in **Discussion, lines 385–414**,
- **Results, lines 305–308**, where the surrogate is framed as a rapid screening tool, not a replacement for CFD/experiments in local-extrema cases.

C) Reproducibility of CFD and ML dataset generation

All ML/AI parameters are included in the supplementary information, including the best ones resulting from the hyperparameter tuning (E.g., Supplementary Information Tables 2-4).

Regarding the CFD data, the full simulation dataset has already been deposited in an online repository, including the CFD cases and associated simulation data:

Maldonado de León, H., Puig I Laborda, V., Naderibeni, M. & Haringa, C. *BioBrAIn: A CFD database of bioreactors*. Preprint at https://doi.org/10.4121/27fd363b-1310-4a69-9635-aa50d9bb7823 (2025).

To further facilitate reproducibility, we Supplementary Data 1 file with the revised submission summarizing the operating conditions and input parameters (operating conditions) used for the 760 CFD simulations.

Modified paper:

- Added sentence in **Methods, lines 458–459**: complete case-level table of global input variables for all 760 CFD simulations is provided as supplementary .csv to the editor.

D) Mesh-Independence Study:

We agree with the reviewer that the mesh independence study should be explicitly documented, since the surrogate models are trained to reproduce the CFD-generated fields. In the original study, a preliminary mesh-sensitivity check had been performed for an individual case during model development, but these results were not included in the submitted manuscript. We have now expanded this analysis and added a dedicated mesh-independence section to the Supplementary Information.

Specifically, we performed a mesh-density study on five randomly selected cases from the 760 CFD simulations. Repeating a full mesh study for every simulation would be computationally prohibitive; therefore, we selected representative cases and re-simulated them using their operating conditions (liquid height, viscosity, stirring speed…). For each case, four mesh densities were evaluated: coarse mesh, coarse-medium mesh, the standard mesh used throughout the study, and a very fine mesh.

Mesh convergence was assessed using both global and local diagnostics. Global integral quantities included torque, power input and volume-averaged flow variables, while local dynamic diagnostics included the convergence evolution of flow variables (e.g., velocity magnitude or turbulent energy dissipation rate) at fixed monitoring points. Although some global quantities were already reasonably stable at coarser resolutions, we retained the standard mesh as a conservative choice to reduce local discretization sensitivity. This was particularly important because performing a mesh-independence study for all 760 simulations was computationally infeasible; therefore, we selected a slightly finer mesh to ensure robust results across the full automated simulation campaign while maintaining a manageable computational cost.

Modified paper:

- Added Methods reference in **Methods, lines 518–521**.
- Full SI section added: **B.3. Mesh independence study, lines 984–1023**.

E) Validation analysis:

We also agree that the original manuscript did not sufficiently document the plausibility of the CFD-generated hydrodynamic fields. We therefore added a new Supplementary Information section comparing CFD-derived effective power numbers against classical fully turbulent Rushton-impeller correlations.

For three representative cases spanning different liquid heights, stirring speeds, fluid properties and numbers of submerged impellers, we calculated the impeller Re number

and the effective per-impeller power number from the CFD-predicted torque. The resulting power numbers were nearly constant across cases and were like the classical turbulent Rushton-impeller values reported in the literature. This supports the hydrodynamic consistency of the automated CFD workflow within the assumptions of the RANS/MRF formulation.

We emphasize, however, that the objective of this manuscript is not to develop or validate a new high-fidelity CFD framework. Rather, the objective is to evaluate whether ML surrogates can accurately emulate CFD-predicted 3-D substrate fields at greatly reduced computational costs. Accordingly, we now state more clearly that the surrogate models inherit the assumptions and limitations of the underlying CFD formulation. The added power-number comparison should therefore be interpreted as a hydrodynamic sanity check for the numerical workflow, rather than as a complete experimental validation of industrial-scale stirred-tank hydrodynamics.

We have added this analysis as a new section in the Supplementary Information **"B.4. Hydrodynamic validation against literature correlations"** and have included new text referring to it in the Methods section.

Modified paper

- Added Methods text in **Methods, lines 557–569**.
- Full SI section added: **B.4. Hydrodynamic validation against literature correlations, lines 1024–1092**.

Reviewer #2 (Remarks to the Author):

The paper by Víctor Puig-I-Laborda develops a methodology for automatic development of CFD surrogate models to describe 3D substrate gradients in large stirred-tank bioreactors. A key innovation of the study is the ability to simulate these 3D substrate gradients within milliseconds, representing a significant improvement in computational efficiency compared to typical CFD simulations. As the authors rightly note, integrating CFD with biological reaction mechanisms and their associated kinetics remains rare in the literature. In this regard, the work constitutes a clear advancement over the current state of the art.

In terms of practical applicability, this study opens the possibility for model-basted process monitoring and control in real-time that explicitly account for substrate heterogeneities in large-scale vessels. This represents a highly relevant area and an essential direction for future research.

Different machine learning (ML) approaches were systematically benchmarked. The best-performing model was a standard neural network (NN) incorporating an attention mechanism. However, the results are not readily generalizable to other problem

domains. More importantly, the strength of the study lies in its methodology, which is well-reasoned and correctly implemented, constituting the paper's primary contribution. The discussion and conclusions are well supported by the results.

I have however some doubts in some parts of the paper and I would like some additional clarifications.

Q1: Physics-informed neural networks were applied several times for CFD including 3D flows.
Cai, S., Mao, Z., Wang, Z. et al. Physics-informed neural networks (PINNs) for fluid mechanics: a review. Acta Mech. Sin. 37, 1727–1738 (2021). https://doi.org/10.1007/s10409-021-01148-1

In fact, the first paper that introduced PINNs addressed a CFD case study. M. Raissi, P. Perdikaris, G.E. Karniadakis, Physics-informed neural networks: A deep learning framework for solving forward and inverse problems involving nonlinear partial differential equations, Journal of Computational Physics, Volume 378, 2019, Pages 686-707, https://doi.org/10.1016/j.jcp.2018.10.045.
Moreover, PINNs were applied for CFD of stirred tank bioreactors, e.g.: Horiguchi I, Shima K, Okano Y. Physics-informed neural networks (PINNs) for high-resolutional prediction of shear stress on cells in suspension culture. AIChE J. 2025;71(7):e18853.doi:10.1002/aic.18853
In the case of PINNs, the neural network can be trained directly on the Navier Stokes equations. It is a different concept with the same purpose. The main section of the paper would benefit from a reference to the PINN methodology for CFD. Even if it has never been applied for 3D substrate gradients. The discussion would also benefit with a reference to potential advantages/disadvantages of the surrogate approach in relation to the PINN approach.

Nice point, we agree that PINNs represent a highly relevant and complementary scientific-ML approach for CFD. We have therefore added a new discussion comparing the current approach with PINNs.

Conceptually, the two approaches are related but target different use cases. PINNs are particularly attractive for data-sparse or inverse problems, where physical residuals can compensate for limited labelled data. In contrast, our goal was to emulate an existing CFD workflow over a large operating space using a supervised database of CFD-generated 3-D substrate fields. This requires substantial upfront CFD data generation, but once trained, the surrogate provides millisecond-scale inference without solving PDE residuals or rerunning CFD. We now clarify that PINNs are a promising future direction, especially for reducing data requirements or enforcing physical constraints, while the present work focuses more on rapid interpolation within a CFD-derived design space.

PINNs have now been introduced in the intro section and compared with our current approach in the discussion.

- Introduction: PINNs paragraph, **lines 62–71**.
- Discussion: PINNs comparison, **lines 404–410**.

Q2: Figure 1 organization is very clear. I would however recommend to replace the box "test on Dev. Set" by "Validate on Validation Set". I am assuming that any decision on hyperparameters was taken on the basis of the validation error. Could the authors please confirm?

Nice catch! Indeed, we used the validation set to select the hyperparameters! That has been corrected now.

- Figure-only correction; no line-numbered text change.

Q3: I imagine a very significant computational cost to run this pipeline (the 760 CFD simulations + ML surrogate models development and benchmarking). This information is neglected in the paper. Could the Authors add this information to the paper? Since the results are not generalizable to different domains, it is important to known the cost of repeating the pipeline for a different domain.

Good point! Indeed, the original manuscript focused mainly on the inference speed of the trained surrogates and did not sufficiently report the upfront computational cost required to generate the CFD database and develop the ML models. This info becomes more important as this is not a generalizable pipeline.

We have therefore revised the **Computational Efficiency and Real-Time Inference** section to explicitly distinguish between: (i) the one-time offline cost of CFD data generation and surrogate-model development, and (ii) the online cost of using the trained surrogates. We now report the approximate computational cost, hardware used, additional cost associated with ML and NN tuning/fine-tuning.

Importantly, this upfront cost is only required when constructing a new domain-specific surrogate model. Once the CFD database has been generated and the surrogate trained, full 3-D substrate fields can be predicted in milliseconds, enabling rapid operating-window exploration. We have clarified this trade-off in the revised manuscript.

- Results: offline CFD/model-development cost, **lines 335–354**.
- Final inference/screening statement, **lines 360–363**.

Q4: Regarding the data generation step, the 760 CFD simulations assume fed-batch operation and are run dynamically until a pseudo-steady state is reached. Even if the Monod substrate uptake cannot the generalized to all cases, it is very interesting that qsmax and KS are global input variables. This allows the surrogate model to infer a different kinetic parameters set without the need to retain it. However, I have some

concerns about the biomass concentration. The biomass concentration (another global variable) is assumed to be constant over time (please confirm). This has the advantage that 3D substrate steady-state obtained by CFD does not depend on the initial condition. dS/dt = qsmax S/(KS+S)*X – D(S -Sin) = 0 => S = f(parameters=(qsmax, KS, X, D, Sin)) Indeed, initial conditions are never inputs to the surrogate models. However, the 3D steady-state snapshot obtained in this way is not a good representation of a typical in fed-batch operation. Could this be a practical limitation? Why didn't the authors assume a CSTR instead?

We thank the reviewer for this very insightful comment. We confirm that, within each individual CFD simulation, biomass concentration was treated as constant. However, biomass concentration was varied between simulations as a global input variable in the LHS design, similarly to $q_{S,\max}$, K_S, feed rate and operating conditions. Thus, each CFD case should be interpreted as a pseudo-steady snapshot of a particular process state, rather than as a fully dynamic fed-batch trajectory. In this formulation, biomass is not assumed to be constant over the entire fermentation; rather, its value must be supplied to the surrogate at the process state of interest, for example from in-line measurements, soft sensors or a separate process-dependent growth model.

We agree that this is a practical limitation if the objective is to predict the complete fed-batch evolution directly from initial conditions. We deliberately did not impose a universal biomass-growth model, because growth dynamics depend strongly on organism, strain, medium, product formation and local substrate exposure. Instead, biomass concentration was treated as a conditional input to the gradient-prediction problem. The same logic applies to other slowly varying process properties, such as density and viscosity, which may either be measured, estimated or predicted by a separate macroscopic model before being passed on to the surrogate.

A CSTR formulation could also be implemented within the same framework, but it would correspond to a different physical problem, with explicit outflow/dilution terms and a different residence-time structure. We selected the fed-batch snapshot formulation because fed-batch operation is highly relevant in industrial bioprocessing and provides a more challenging setting for demonstrating spatial substrate-gradient prediction.

- Methods: revised snapshot strategy, **lines 489–495**.

Q5: The CFD simulations assumes a standard stirred tank configuration with 4 Rushton-impellers and fixed reactor size. Could the surrogate models be applied to a different tank size while keeping the same geometry and configuration?

Interesting observation. The current surrogate models were trained for a fixed stirred-tank configuration and reactor-size; therefore, they should not be directly applied to a different tank size or geometry without additional CFD (or real) data. Even if the same geometric configuration is retained, changing scale can alter the relevant hydrodynamic regime

(e.g., Re number), power input per volume, turbulent length scales, mixing time and compartment-to-compartment exchange. As a result, the underlying substrate-gradient patterns may differ from those learned by the present model.

The current model should therefore be interpreted as valid for interpolation within the sampled operating domain of the studied reactor configuration, not as a scale-invariant predictor. However, the framework itself is general. A natural next step is to extend the training database to parametrized geometries, for example including vessel dimensions, impeller diameter, spacing, type as additional model inputs. The current dataset could serve as one part of a larger training database, supplemented with new CFD simulations spanning additional scales and geometrical configurations.

We have clarified this limitation in the revised discussion. In its present form, the surrogate does not enable direct geometric optimization, but it provides a foundation for future geometry-aware surrogates trained across multiple reactor sizes and configurations.

- Discussion: single geometry/scale limitation, **lines 385–395**.
- Future parametrized geometries, **lines 424–430**.

Q6: The standard feedforward neural network with Modified-Attention achieved the lowest test MSE (0.0054) and a test R2 of 0.91 (Figure 2). Moreover, the test MSE is comparable (even a bit lower) than the train MSE, which is an excellent result. The authors state that "the Polynomial–Lasso regressors at intermediate degree (3rd-4th order) emerged as the next best class". However, the train loss is roughly 50% of the test loss, this is not so positive and could suggest overfitting. The closest match is the standard ANN without the attention mechanism. Are the differences significant between the NN with and without attention? Why does attention matter in this case, given the low dimension and uncorrelated inputs (Extended Figs. 3-5)?

Insightful observation. We agree that the 3rd–4th order Poly-Lasso regressors show a larger development–test gap than the NN models, indicating some degree of overfitting. However, we do not consider this gap alone sufficient to discard these models, because model quality depends on both bias and generalization behavior. For example, simple MLR-derived models show very small development–test gaps, but their test errors remain high, indicating low variance but substantial bias. In contrast, intermediate-degree Poly-Lasso models reduce this bias considerably and achieve low test errors, although at the cost of a larger generalization gap.

Regarding the attention mechanisms. Good point with the Extended Data Figs. 3-5, however, these analyses only assess pairwise linear correlations or linear combinations of standardized inputs. They do not evaluate non-linear interactions between global operating conditions and local spatial descriptors. The attention-based architecture was designed precisely to model such interactions by allowing global process variables to

modulate the local spatial representation. This is relevant for the present problem, because the effect of local coordinate or distance to feed point to the gradient, depends on global conditions.

- Results: attention mechanism and Poly-Lasso generalization gap, **lines 169–180**

Q7: The effect of training data size on the test loss shows a saturation. Again the NN with modified attention mechanism stands out as the best approach. The authors conclude that “beyond a few hundred additional runs, the improvements in accuracy become increasingly marginal relative to the computational cost”. The test R2 was 0.91. What about the train R2? I am assuming it was close to 0.91 (given the comparable MSE). Why does the pipeline saturate at 0.91 test R2? Could this be related to the ANN topology and/or training algorithm? Maybe underfitting?

The development-set R2 for all the best performing models (remember development is used as the training set in the last training step) was comparable to the held-out test-set R2 reported in the manuscript (Extended Data Fig. 8). Thus, learning-curve saturation is unlikely to be caused primarily by overfitting; so indeed it suggests that the remaining error is dominated by model bias, target representation and localized flow features that are difficult to learn from the current input representation (so, in general underfitting).

Therefore, the best NNs seem to be limited not by the amount of data but by the model type. Additional training data alone therefore provides diminishing returns, because the remaining errors are associated with localized hydrodynamic structures and rare extreme regions rather than insufficient coverage of the bulk operating space. More expressive architectures with stronger spatial inductive bias, such as graph neural networks, neural operators or transformer-based models, may better exploit larger datasets and reduce this residual error, together with a better sampling strategy to sample high-error regions.

- Results: learning-curve interpretation and diminishing returns, **lines 239–243**; extra-input-variable paragraph, **lines 251–256**.

Q8: The 10-folds CV in the supplementary information delivered a validation loss mean and dispersion coefficient, which are not given in the paper. If the authors compare the validation loss across the different ML methods (the ones with hyperparameter challenges), would the same conclusion be reached as the one in Figure 2?

The cross-validation reported in the Supplementary Information was used for hyperparameter selection within the development set, not for the final model comparison on the test set. For each model and hyperparameter configuration, the validation loss was averaged across folds and used to select the optimal configuration. The final comparison in Figure 2 was then performed on the independent held-out test set of 200 CFD simulations, which was not used during hyperparameter tuning (as seen in Figure 1).

Comparing the best CV validation losses across model classes leads to the same overall conclusion as Figure 2: The NNs, particularly the attention-based architectures, achieved the strongest performance, whereas simpler linear models were stable but more bias-limited. Minor differences in exact ordering are expected because CV losses are computed within the development set during model selection, while Figure 2 reports final generalization on unseen simulations. (CV in this work should only be seen as a means to optimize the hyperparameters in the models and not as a final comparison between models).

- Mostly existing Methods: validation and hyperparameter optimization, **lines 736–812**.

Q9: Please include in the captions of Supplementary Information Figs. 12-28 that the residual analysis refers to the test data only. Was the residuals distribution better for the Development Data set?

Captions revised for the supplementary information to explicitly state that all residual analyses were performed on the independent held-out test set only.

The residual distributions on the development set were generally similar or slightly narrower, as expected, particularly for more flexible models. However, we chose to report the test-set residuals because they provide the most relevant and conservative assessment of generalization to unseen CFD simulations. Importantly, the key conclusions from the residual analysis were unchanged: the best-performing NNs showed residuals concentrated around zero within limited residual–predictor correlation.

- SI residual figures now state “on the test set”: **lines 1227–1268.**

Reviewer #3 (Remarks to the Author):

In their manuscript the authors present in detail a surrogate modelling methodology aimed at using data-driven methods to recover the three-dimensional profiles of substrate concentrations predicted by CFD simulations in the context of fermentation operations. The document is well-written and the framework proposed therein is highly relevant for the improvement of model-based analysis and optimisation of bioprocessing applications. The discussed methodology is sound and the best-performing surrogate model appears to offer satisfactory predictive power. However, in my opinion the article does not fully demonstrate the stated advantages of the prosed framework, i.e. the ability to (partially) replace and/or complement 3D CFD predictions. For these reasons, I recommend the manuscript to be published after the authors have addressed the following points.

1.It is not completely clear to what extend the surrogate models can interpolate along the explored 10D hyper-surface. The authors discuss on page 11 that the developed model can be used to screen multiple operating points without the need of conducting additional CFD simulations. For this to be realised, model predictions need to be well-behaved when some or all of the inputs are in-between the points in the grid used for training. Although the text reports similar MSEs between development and test sets for the best model, the presented residual analysis does not distinguish between the sets (I presume the plots show both). This makes it difficult to assess if the discussed patterns regarding model errors are different for the holdout data. Furthermore, the text should show that model predictions behave correctly. For example, if qs/qs_max at a given point is expected (or observed from simulations) to change smoothly with an increasing stirring speed, surrogate model predictions for a dense array of speeds should be approximately smooth as opposed to jagged or discontinuous.

Nice point, we agree that low test-set error on held-out LHS cases demonstrates generalization to unseen simulations but does not by itself prove that the surrogate behaves smoothly along continuous interpolation paths within the sampled operating space. We also clarify that the residual analyses shown in the Supplementary Information were performed on the independent held-out test set only, not on combined development and test data. Development-set residuals showed similar quantitative trends and were generally slightly narrower, as expected; however, we focused on the test set because it provides the most conservative assessment of generalization to unseen CFD simulations. We have revised the residual-analysis captions to state this explicitly.

To directly address the interpolation concern, we added a new smoothness/interpolation analysis. Because exhaustive interpolation validation across the full 11-dimensional input space would be computationally prohibitive, we evaluated representative one-dimensional slices through the sampled domain. Starting from representative operating points, we varied one global variable at a time while holding all other inputs fixed. Specifically, we analyzed sweeps in $q_{S,\max}$, y_{feed} position and stirring speed, which probe kinetic intensity, feed placement and mixing intensity, respectively.

For each sweep, additional CFD reference points were compared with predictions from the trained surrogates at fixed local monitoring points. The resulting curves showed smooth, non-jagged surrogate responses across the tested interpolation paths. The best-performing NNs generally followed the CFD trends more closely than the linear baseline.

- Results: interpolation sentence, **lines 212–214**.
- New SI section **B.12**, **lines 1271–1305**, including Table 8 and Fig. 31.

2.Similarly, it is unclear if the developed models have any extrapolation power. It is acknowledged that the presented work aims to show a proof of concept. However, the value of this work would be greatly enhanced if the authors can prove that the surrogate

models can offer some degree of predictive power when one or more of the inputs are outside the intervals showed on table 1. More importantly, the article emphasises the advantages derived from fast inference times after an initial and substantial computational investment, which corresponds to the original CFD simulations in addition to model training. Consequently, the relevance of the presented approach would be significantly degraded if too many new CFD simulations are needed to allow the surrogate model to account for relatively small changes in operational and/or geometric parameters. Can the authors estimate where this threshold lies? Is it possible for any of the tested models to cope with any changes in the reactor geometry? If not, would this imply that the proposed method cannot be used to conduct geometric optimisations?

We thank the reviewer for this important point. We did not explicitly test extrapolation outside the ranges in Table 1 and therefore do not claim validated extrapolation power beyond the sampled domain. The design space was deliberately chosen to be broad so that practical use would rely mainly on interpolation rather than extrapolation.

Limited extrapolation may be possible when the dominant regime remains unchanged, for example within the same turbulent-flow, Newtonian, single-geometry setting. However, changes that alter the governing physics, such as different rheology, flow regime or reactor geometry, would require additional CFD data and retraining or fine-tuning.

The current model should therefore not be used directly for geometric optimization. A geometry-aware surrogate would require an expanded CFD database including geometric descriptors such as impeller type, aspect ratio, impeller spacing or scale parameters. However, the number of required simulations increases with input dimensionality, so this must be done through targeted experimental design rather than exhaustive sampling. Based on the flattening of the learning curves, limited extensions may be feasible through targeted CFD sampling rather than exhaustive resampling of the expanded design space. However, the number of additional cases required for each new variable cannot be inferred from the present study and will depend on how strongly that variable alters the flow or reaction regime.

- Results: extra input variables paragraph, **lines 251–256**.
- Discussion: scale/geometric limitation, **lines 381–395**; future geometry-aware extensions, **lines 424–430**.

3.As discussed on page 9, prediction errors concentrate on high-shear zones and in saturated and depleted zones. These are the regions where the usage of high-resolution CFD simulations has the highest value. Please discuss further the industrial implications of this limitation. Moreover, please assess how does the presented surrogate model compare with other computationally efficient alternatives, such as tank-in-series or other possible types of compartmental models.

We agree that the regions with the highest surrogate errors are also regions where high-resolution CFD has the greatest value. We have already discussed that these errors likely reflect local data sparsity and that future work should include targeted sampling of high-gradient/high-error regions and loss reweighting. We have now expanded this discussion to make the industrial implications clearer.

In its present form, the surrogate should primarily be used for rapid screening, sensitivity analysis and operating-window exploration within the sampled domain. It can identify operating conditions likely to generate strong substrate heterogeneities at negligible cost. However, when the exact magnitude of local extremes is critical, the surrogate should not be considered a full replacement for high-resolution CFD or experimental validation. Rather, it can serve as a fast pre-screening layer to identify cases that merit detailed CFD follow-up.

A small comparison with compartment models was already included in the Discussion, but we have now expanded it to clarify the relationship between the two approaches. Compartment and tank-in-series models are highly useful for dynamic process simulations and cell-history modelling, but they typically rely on a hydrodynamic reduction of the reactor into average zones and exchange terms, often derived from CFD, tracer experiments or empirical mixing assumptions. They are therefore not directly equivalent to the present framework, which learns a direct mapping to CFD-predicted local 3-D substrate fields.

We have also clarified that the two approaches address different modelling needs. Compartment models are particularly useful when detailed biological models or trajectory-wise cellular exposure histories are required, but they depend on a hydrodynamic reduction of the reactor into compartments and exchange terms. If operating conditions or reactor configurations substantially change, this hydrodynamic description may need to be recomputed or reparametrized. The present surrogate, in contrast, is designed to rapidly explore how changes in operating conditions affect CFD-predicted 3-D substrate fields within the sampled domain. However, in its current form, the biological description is simpler and tied to the uptake kinetics represented in the CFD training data. We have added text to the Discussion to clarify this complementarity and the different positions these approaches occupy in the cost–resolution–dynamics trade-off.

- Results: local-error screening role, **lines 295–308**.
- Discussion: CMs and spatial-resolution comparison, **lines 375–376** and **396–400**.

4.Figure 4 and the parity plots in the supplementary materials show that the best surrogate model can recover the target to a high degree. However, the residual error can climb to be around 20-30% or higher. Is this satisfactory enough for the intended industrial application? Following the previous remark, does this error level allows for the

surrogate model to be more informative than, for instance, a simpler compartmental approach, which would not require the elevated computational expenses of the surrogate model?

Indeed, local residual errors of about 20–30% would not be satisfactory if the objective were to replace CFD for the final quantification of local extremes (e.g., peak concentrations near the feeding point or severe depletion zones). However, these larger errors occur only in localized regions of the reactor, whereas the global error and the average field reconstruction error are substantially lower. We have clarified this point in the previous answer.

In this context, the surrogate can still be highly informative, allows many operating conditions to be explored and problematic cases to be identified before selecting a smaller number for high-resolution CFD or experimental follow-up. Thus, the relevant comparison is not only pointwise error, but the trade-off between spatial resolution, computational cost and intended decision.

We also agree that CMs are valuable computationally efficient alternatives, but they are not directly equivalent to the present surrogate. CMs are well suited for dynamic process simulations, cell-history analysis and coupling with detailed biological models, but they represent the reactor through averaged zones and exchange terms rather than local 3-D fields. The present surrogate requires an upfront CFD campaign, but once trained it enables rapid reconstruction of CFD-consistent 3-D substrate gradients across many operating conditions within the sampled domain rather than derived from an initial CFD simulation.

- Results **lines 305–308**
- Discussion **lines 396–400**.

5.The authors assert that the developed model can potentially integrate process dynamics by using a snapshot strategy. Please show an example of such predictions, preferably accompanied by the results of transient CFD simulations (or experimental data, if available).

By "snapshot strategy", we refer to pseudo-st.st. CFD simulations representing different process states along a fed-batch trajectory. In practice, global state variables such as biomass concentration, feed rate, feed position, density or viscosity parameters could be obtained from measurements, soft sensors or a macroscopic process model, and the surrogate would then predict the corresponding instantaneous 3-D substrate field.

Such industrial-scale 3-D spatio-temporal CFD or experimental datasets are not currently available for validation at the scale considered here (probably proprietary for some industries). Therefore, we cannot provide transient CFD or experimental comparisons in the present study.

We have clarified the wording in the revised manuscript to avoid implying that the current surrogate directly integrates process dynamics. Instead, it predicts pseudo-steady 3-D fields conditional on supplied process-state variables, which may represent successive snapshots along a fed-batch trajectory.

- Methods: revised pseudo-steady snapshot wording, **lines 481–495**.

6.How was mesh-independence ensured within the automated workflow used to generate the dataset?

We thank the reviewer for raising this important point. We fully agree that mesh-independence should be explicitly documented, as the surrogate models are trained directly on CFD-generated fields and therefore inherit the numerical properties of the CFD solutions.

During development of the automated workflow, an initial mesh-sensitivity analysis was performed to select a mesh resolution that provided stable hydrodynamic predictions while remaining computationally feasible for large-scale data generation. In the revised manuscript, we have expanded this analysis and added a dedicated mesh-independence section to the Supplementary Information (**B.3. Mesh independence study**). Specifically, 5 representative cases were selected from the 760-simulation campaign and re-simulated using four mesh densities: coarse, coarse-medium, standard and fine. Each case was simulated using its original operating conditions, including liquid height, fluid properties, stirring speed…

Mesh convergence was evaluated using both global and local diagnostics. Global quantities included torque given by each impeller, power input, volume-averaged flow variables, while local diagnostics included convergence evolution of fluid variables (e.g., velocity magnitude or turbulent energy dissipation rate) at fixed monitoring points. Across tested cases, the standard mesh used in the full simulation campaign closely reproduces the very fine-mesh behavior for both global quantities and representative local flow dynamics. Coarser meshes were already adequate for some integral quantities but showed larger deviations in selected local diagnostics.

We therefore retained the standard mesh as a conservative compromise between numerical accuracy and computational feasibility. This choice was particularly important because performing a complete mesh-independence study for all 760 simulations is computationally infeasible. Once selected, the same meshing strategy and target mesh density were applied consistently within the automated workflow for all generated cases.

- Methods **lines 518–521**
- Section **B.3. Mesh independence study** was added in the Supplementary Information.

7.Did the authors conduct a feature importance analysis on the non-MLR models?

Nice question! We did not conduct a systematic feature-importance analysis for the non-MLR models. Feature importance was assessed explicitly for the linear baseline through best-subset analysis, and more generally through predictor correlations and residual–predictor correlations. For non-linear models, however, feature importance is model-dependent and not directly comparable across architectures: tree-based impurity measures, permutation importance, SHAP-type analyses and neural-network attribution methods can lead to different interpretations, especially when inputs interact non-linearly.

Because the main objective of this study was predictive benchmarking and 3-D field reconstruction, we focused on generalization performance and residual diagnostics rather than model-specific attribution. This paper is already extensive, but if future work focuses on a smaller subset of models, for example only neural networks, it would be valuable to perform dedicated attribution analyses using model-agnostic methods such as permutation importance or SHAP, or architecture-specific methods such as gradient-based sensitivity analysis.

- No new text added. Existing support:
- Results best-subset discussion, **lines 151–163**;
- Extended Data Table 1, **lines 894–909**;
- SI best-subset discussion, **lines 1103–1125**.

8. Please provide some discussion justifying the absence of experimental validation of the CFD models. What is the expected effect of the surrogate model inheriting the assumptions of the CFD setup?

We agree that the absence of experimental validation should be stated clearly. The objective of this study was not to develop or validate a new CFD model, but to test whether machine-learning models can emulate a consistent CFD workflow at greatly reduced computational cost. For this reason, we benchmarked the surrogates against the CFD simulations used as the numerical reference, and we added mesh-independence and hydrodynamic consistency checks to verify that the automated CFD pipeline produced physically plausible results.

The consequence is that the surrogate should be interpreted as a fast emulator of the underlying CFD setup, not as an experimentally validated representation of the real reactor. Therefore, it inherits the assumptions and biases of the CFD formulation, including RANS turbulence closure, MRF treatment of impeller motion, single-phase flow and Monod kinetics. Any systematic CFD error would be propagated into the surrogate predictions.

We have clarified this in the revised Discussion and Supplementary Information. Future extensions should combine CFD-generated data with experimental measurements where available, or use hybrid training strategies in which experimental data anchor the

surrogate while CFD expands the accessible operating space. We also note that direct validation of industrial-scale 3-D CFD fields in bioreactors is rarely available, because spatially resolved measurements are highly constrained by stainless-steel vessel design, limited optical access, sterility requirements, intrusive probe effects and the difficulty of measuring full 3-D concentration or velocity fields under production-relevant conditions.

- Discussion: CFD-consistent emulator and inherited assumptions, **lines 382–395**.
- Methods: hydrodynamic sanity check and inherited RANS/MRF limitations, **lines 557–569**.
- Supplementary Information: B.4 Hydrodynamic validation against literature correlations, **lines 1024–1092 (new section included)**.

9.How do the authors expect the modelling framework to behave in the presence of non-Monod kinetics?

This is a relevant point, and the current model should not be expected to generalize directly to fundamentally different non-Monod kinetics without additional training data. However, the framework itself is not restricted to Monod kinetics. More complex biological rate laws could be incorporated by generating additional CFD simulations with the corresponding source terms and by including the relevant kinetic parameters as model inputs.

Importantly, this would not necessarily require rebuilding the framework from scratch. A transfer-learning strategy could be used, where the current Monod-trained surrogate provides an initial model that is then fine-tuned on additional CFD cases covering the new kinetic regimes. This could reduce the amount of new data required compared with training a new surrogate entirely from zero.

- Discussion: future non-Monod bio-kinetics extension, **lines 424–430**.

10.How were the sets of tunned hyperparameters chosen? How were the neural network architectures defined?

The hyperparameter selection procedure is described in Figure 1, the Methods section “Validation strategy and hyperparameter optimization”, and Supplementary Information Tables 2–4.

Briefly, all tunable models were optimized only on the development set using grouped cross-validation at the simulation level, so that all mesh cells from the same CFD simulation remained in the same fold. For classical ML and regularized regression models, we used grid search over the candidate hyperparameter values reported in Supplementary Information Table 2. The configuration with the lowest mean cross-validation MSE was selected, then retrained on the development set and finally evaluated once on the independent held-out test set.

For neural networks, the hyperparameter space was larger and included network architecture, learning rate, batch size, dropout, weight decay and early-stopping patience. We therefore used Optuna/TPE Bayesian optimization to minimize the mean cross-validation MSE. The candidate NN architectures and hyperparameter ranges are listed in Supplementary Information Table 3, while the optimum configuration selected for each NN architecture is now reported in Supplementary Information Table 4.

- NN architectures: **lines 677–735**.
- Validation/hyperparameter optimization: **lines 736–812**.
- Supplementary Information hyperparameter tables: **lines 1093–1102**.

Minor comments

11.Page 12: ‘can be emulated’

Corrected

12.There are some “Error! Reference source not found’’ instances in the supplementary materials file.

Corrected
