# Supplementary material for: Machine-learning surrogates enable rapid prediction of 3-D substrate gradients in industrial bioreactors
Source: Nat Commun. 2026 Oct 15;17:11636. doi: 10.1038/s41467-026-78273-7 (PMC13754804; doi:10.1038/s41467-026-78273-7)
Supplement: Supplementary file 4 [file 41467_2026_78273_MOESM4_ESM.zip › supplementary_software_1/figure_05_runtime_speedup/figure_05_runtime_speedup.pdf]

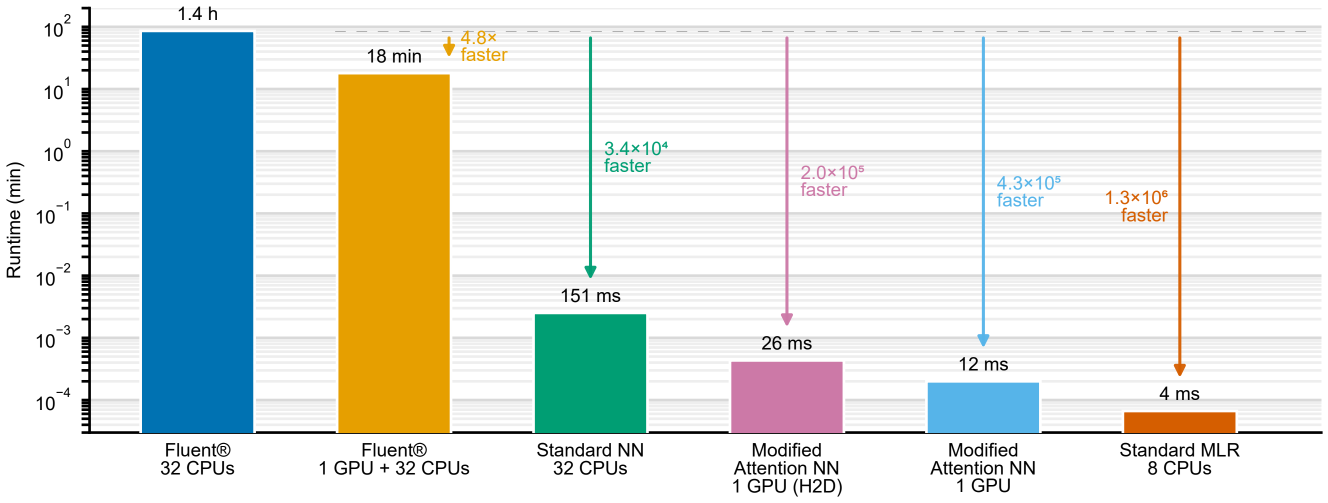

Runtime (min)
10^2
10^1
10^0
10^-1
10^-2
10^-3
10^-4
1.4 h
18 min
4.8× faster
151 ms
3.4×10⁴ faster
26 ms
2.0×10⁵ faster
12 ms
4.3×10⁵ faster
4 ms
1.3×10⁶ faster
Fluent® 32 CPUs
Fluent® 1 GPU + 32 CPUs
Standard NN 32 CPUs
Modified Attention NN 1 GPU (H2D)
Modified Attention NN 1 GPU
Standard MLR 8 CPUs
