# Supplementary material for: Machine-learning surrogates enable rapid prediction of 3-D substrate gradients in industrial bioreactors
Source: Nat Commun. 2026 Oct 15;17:11636. doi: 10.1038/s41467-026-78273-7 (PMC13754804; doi:10.1038/s41467-026-78273-7)
Supplement: Supplementary file 4 [file 41467_2026_78273_MOESM4_ESM.zip › supplementary_software_1/figure_05_runtime_speedup/reference/source_outputs/Runtime_Figure.pdf]

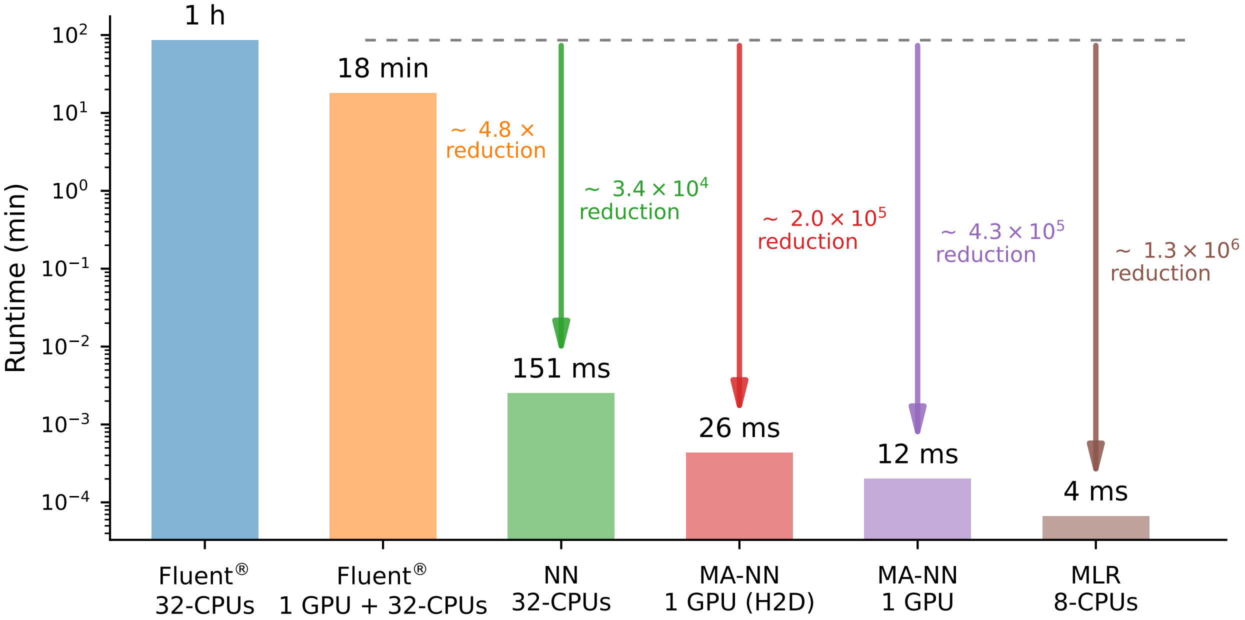

Runtime (min)
10^2
10^1
10^0
10^{-1}
10^{-2}
10^{-3}
10^{-4}
1 h
18 min
151 ms
26 ms
12 ms
4 ms
$\sim$ 4.8 $\times$ reduction
$\sim$ 3.4×10^4 reduction
$\sim$ 2.0×10^5 reduction
$\sim$ 4.3×10^5 reduction
$\sim$ 1.3×10^6 reduction
Fluent® 32-CPUs
Fluent® 1 GPU + 32-CPUs
NN 32-CPUs
MA-NN 1 GPU (H2D)
MA-NN 1 GPU
MLR 8-CPUs
