# Supplementary material for: Machine-learning surrogates enable rapid prediction of 3-D substrate gradients in industrial bioreactors
Source: Nat Commun. 2026 Oct 15;17:11636. doi: 10.1038/s41467-026-78273-7 (PMC13754804; doi:10.1038/s41467-026-78273-7)
Supplement: Supplementary file 4 [file 41467_2026_78273_MOESM4_ESM.zip › supplementary_software_1/figure_06_stirred_tank_schematic/figure_06_stirred_tank_schematic.pdf]

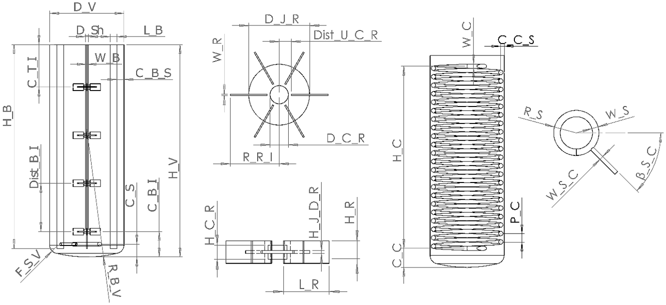
D_V
D_Sh
L_B
W_B
C_B_S
C_T_I
H_B
Dist_B_I
C_S
C_B_I
H_V
F_S_V
R_B_V
D_J_R
Dist_U_C_R
W_R
D_C_R
R_R_I
H_C_R
H_J_D_R
H_R
L_R
W_C
C_C_S
H_C
P_C
C_C
R_S
W_S
W_S_C
β_S_C
