# Supplementary material for: Machine-learning surrogates enable rapid prediction of 3-D substrate gradients in industrial bioreactors
Source: Nat Commun. 2026 Oct 15;17:11636. doi: 10.1038/s41467-026-78273-7 (PMC13754804; doi:10.1038/s41467-026-78273-7)
Supplement: Supplementary file 4 [file 41467_2026_78273_MOESM4_ESM.zip › supplementary_software_1/figure_01_workflow/figure_01_workflow.pdf]

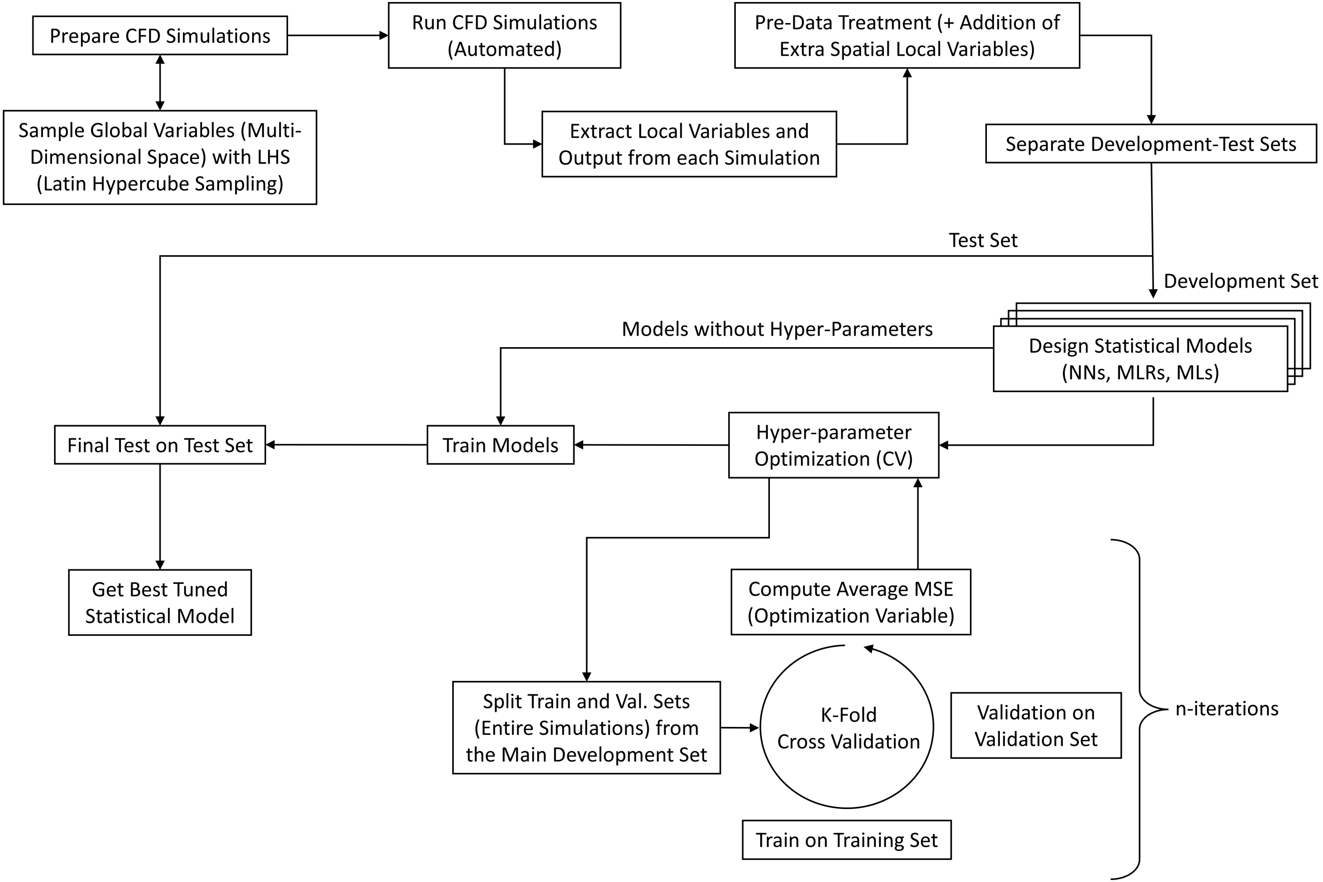

Prepare CFD Simulations
Run CFD Simulations (Automated)
Pre-Data Treatment (+ Addition of Extra Spatial Local Variables)
Sample Global Variables (Multi-Dimensional Space) with LHS (Latin Hypercube Sampling)
Extract Local Variables and Output from each Simulation
Separate Development-Test Sets
Test Set
Development Set
Models without Hyper-Parameters
Design Statistical Models (NNs, MLRs, MLs)
Final Test on Test Set
Train Models
Hyper-parameter Optimization (CV)
Get Best Tuned Statistical Model
Compute Average MSE (Optimization Variable)
Split Train and Val. Sets (Entire Simulations) from the Main Development Set
K-Fold Cross Validation
Validation on Validation Set
n-iterations
Train on Training Set
