# Supplementary material for: Machine-learning surrogates enable rapid prediction of 3-D substrate gradients in industrial bioreactors
Source: Nat Commun. 2026 Oct 15;17:11636. doi: 10.1038/s41467-026-78273-7 (PMC13754804; doi:10.1038/s41467-026-78273-7)
Supplement: Supplementary file 4 [file 41467_2026_78273_MOESM4_ESM.zip › supplementary_software_1/figure_04_spatial_gradients_and_error/figure_04_spatial_gradients_and_error.pdf]

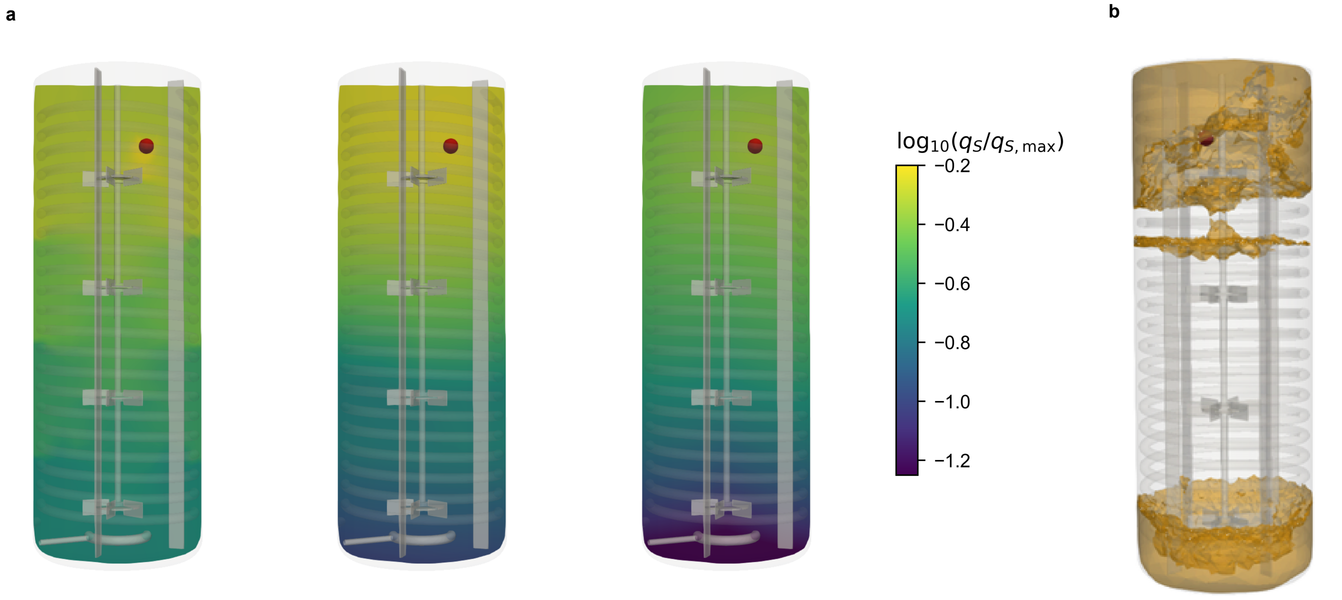
a
b
$\log_{10}(q_S/q_{S,\max})$
−0.2
−0.4
−0.6
−0.8
−1.0
−1.2
