# Supplementary material for: Machine-learning surrogates enable rapid prediction of 3-D substrate gradients in industrial bioreactors
Source: Nat Commun. 2026 Oct 15;17:11636. doi: 10.1038/s41467-026-78273-7 (PMC13754804; doi:10.1038/s41467-026-78273-7)
Supplement: Supplementary file 4 [file 41467_2026_78273_MOESM4_ESM.zip › supplementary_software_1/figure_03_learning_curve_qS_qS_max/reference/source_outputs/Simulations_vs_Error_label_qS_qS_max.pdf]

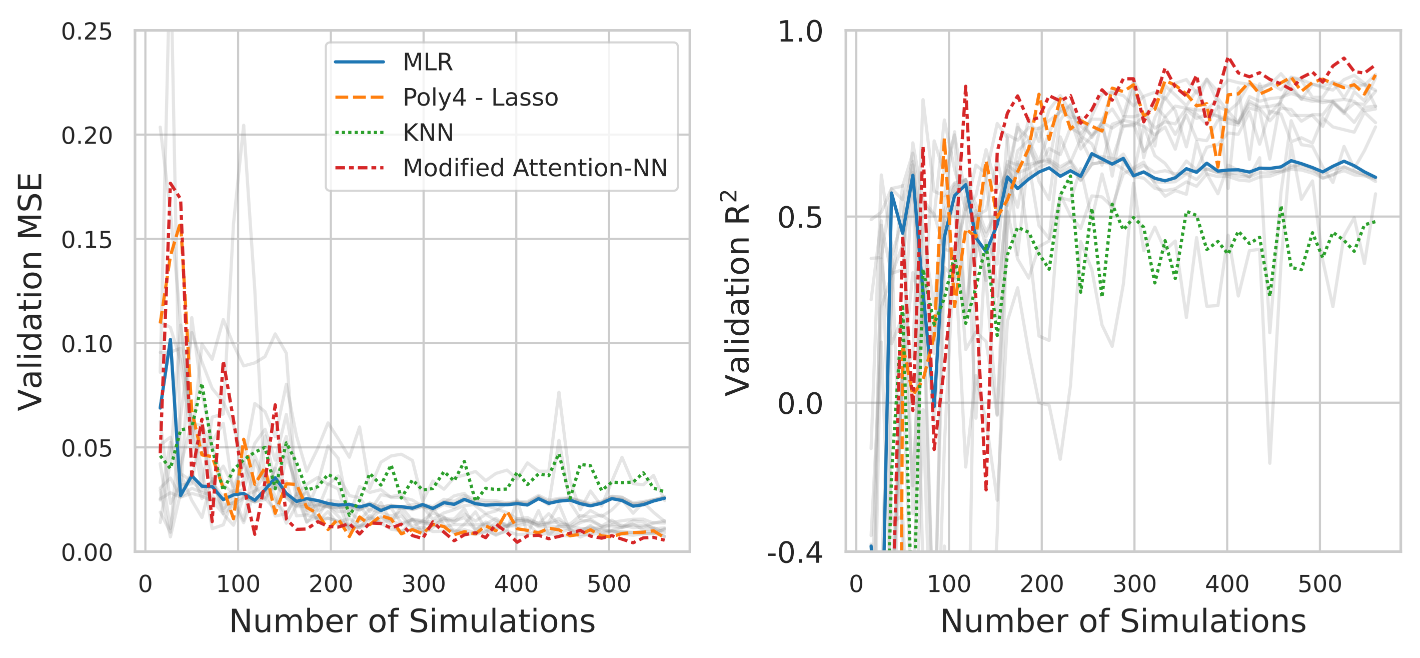

Validation MSE
0.25
0.20
0.15
0.10
0.05
0.00
0
100
200
300
400
500
Number of Simulations
MLR
Poly4 - Lasso
KNN
Modified Attention-NN
Validation R2
1.0
0.5
0.0
-0.4
0
100
200
300
400
500
Number of Simulations
