# Supplementary material for: Machine-learning surrogates enable rapid prediction of 3-D substrate gradients in industrial bioreactors
Source: Nat Commun. 2026 Oct 15;17:11636. doi: 10.1038/s41467-026-78273-7 (PMC13754804; doi:10.1038/s41467-026-78273-7)
Supplement: Supplementary file 4 [file 41467_2026_78273_MOESM4_ESM.zip › supplementary_software_1/figure_03_learning_curve_qS_qS_max/figure_03_learning_curves.pdf]

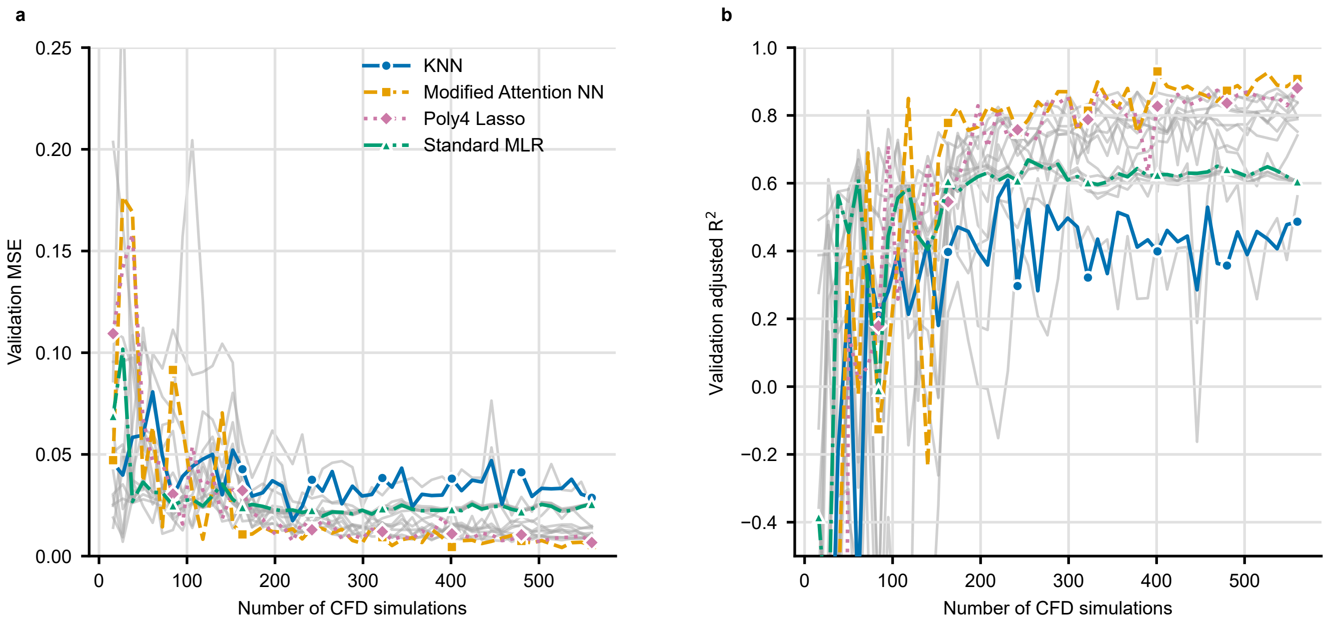

a
b
0.25
0.20
0.15
0.10
0.05
0.00
Validation MSE
0
100
200
300
400
500
Number of CFD simulations
KNN
Modified Attention NN
Poly4 Lasso
Standard MLR
1.0
0.8
0.6
0.4
0.2
0.0
−0.2
−0.4
Validation adjusted R2
0
100
200
300
400
500
Number of CFD simulations
