# Supplementary material for: Machine-learning surrogates enable rapid prediction of 3-D substrate gradients in industrial bioreactors
Source: Nat Commun. 2026 Oct 15;17:11636. doi: 10.1038/s41467-026-78273-7 (PMC13754804; doi:10.1038/s41467-026-78273-7)
Supplement: Supplementary file 4 [file 41467_2026_78273_MOESM4_ESM.zip › supplementary_software_1/figure_02_test_set_mse_qS_qS_max/reference/source_outputs/test_set_MSE_qS_qS_max.pdf]

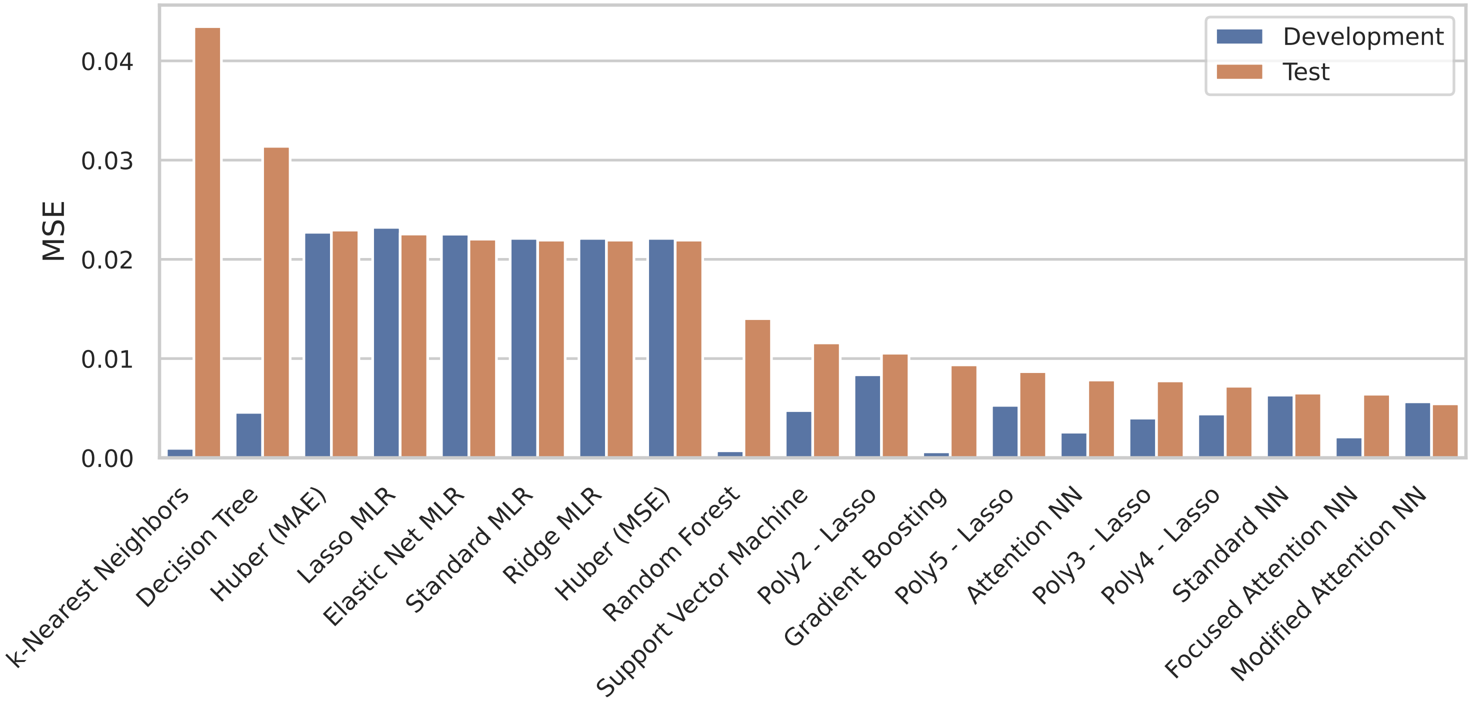

Development
Test
MSE
0.00
0.01
0.02
0.03
0.04
k-Nearest Neighbors
Decision Tree
Huber (MAE)
Lasso MLR
Elastic Net MLR
Standard MLR
Ridge MLR
Huber (MSE)
Random Forest
Support Vector Machine
Poly2 - Lasso
Gradient Boosting
Poly5 - Lasso
Attention NN
Poly3 - Lasso
Poly4 - Lasso
Standard NN
Focused Attention NN
Modified Attention NN
